# The relationship between voluntary product (re) formulation commitments and changes in the nutritional quality of products offered by the top packaged food and beverage companies in Canada from 2013 to 2017

**DOI:** 10.1186/s12889-022-12683-2

**Published:** 2022-02-10

**Authors:** Laura Vergeer, Mavra Ahmed, Lana Vanderlee, Christine Mulligan, Madyson Weippert, Beatriz Franco-Arellano, Kacie Dickinson, Jodi T. Bernstein, Marie-Ève Labonté, Mary R. L’Abbé

**Affiliations:** 1 Department of Nutritional Sciences, Temerty Faculty of Medicine, University of Toronto, 1 King’s College Circle, Toronto, Ontario M5S 1A8 Canada; 2 Centre nutrition, santé et société (NUTRISS), Institut sur la nutrition et les aliments fonctionnels, École de nutrition, Université Laval, Pavillon des Services, 2440 boul. Hochelaga, Québec, Québec G1V 0A6 Canada; 3 Faculty of Health Science, Ontario Tech University, 2000 Simcoe Street North, Oshawa, ON L1G 0C5 Canada; 4 Nutrition and Dietetics, College of Nursing and Health Sciences, Flinders University, G.P.O. Box 2100, Adelaide, SA 5001 Australia

**Keywords:** Food company, Reformulation, Nutritional quality, Nutrient profile, Commitment

## Abstract

**Background:**

Food companies shape Canada’s food supply through voluntary actions and commitments concerning product (re)formulation; however, the extent that these initiatives translate into actual improvements in nutritional quality is unclear. This study examined changes in the nutritional quality of products offered by the top 22 packaged food and beverage companies in Canada from 2013 to 2017, in relation to the strength of their product (re) formulation actions and commitments.

**Methods:**

The Food Company Reformulation (FCR) scoring tool was used to quantify the strength of companies’ reported recent actions and commitments to reduce energy and nutrients of concern in their products, with higher scores signifying stronger voluntary actions/commitments. Nutritional information for products was sourced from the University of Toronto FLIP 2013 (*n* = 6490) and 2017 (*n* = 8277) databases (*n* = 4074 matched products). Changes in product healthfulness were assessed using the Health Star Rating (HSR) system (with higher HSRs denoting healthier products) and calories, sodium, saturated fat, *trans* fat, and total and free sugar levels per 100 g/mL. Generalized estimating equations examined changes in nutritional quality in relation to FCR scores.

**Results:**

Overall, mean HSRs increased significantly for 5 companies’ product portfolios and were reduced in 1 company’s product portfolio. There were significant reductions in calories, sodium, saturated fat in 2 companies’ portfolios and increases in 4, 3, and 8 companies’ portfolios, respectively. *Trans* fats increased significantly in 2 companies’ portfolios. Total and free sugars decreased significantly in 4 and 5 companies’ portfolios, respectively, and increased in 1 company’s portfolio. There was little change in the healthfulness of matched products. Higher FCR scores were not associated with greater increases in HSRs, or reductions in calories or nutrient amounts. FCR scores were negatively associated with HSRs and positively associated with total and free sugars. No relationship was observed between FCR scores and calories, sodium, saturated fat or *trans* fat.

**Conclusions:**

Reporting stronger voluntary product (re) formulation actions and commitments was not associated with greater improvements in the healthfulness of products offered by Canada’s leading packaged food and beverage companies from 2013 to 2017, suggesting a need for stronger industry initiatives or mandatory government interventions to improve the healthfulness of the food supply.

**Supplementary Information:**

The online version contains supplementary material available at 10.1186/s12889-022-12683-2.

## Background

Packaged food supplies dominated by energy-dense products containing excessive amounts of saturated fat, sodium and sugars promote poor diet quality and increase consumers’ risk of developing obesity and non-communicable diseases (NCDs) [1]. In Canada, approximately 50% of average daily energy intakes come from ultra-processed foods, many of which are energy-dense and high in sodium, saturated fat and/or sugars [2–4]. Consequently, Canadians with the highest intakes of ultra- processed foods are more likely to have obesity, and excessive intakes of saturated fats, sodium, added sugars and energy [5–7]. For example, Canadians consume approximately 2760 mg of sodium per day, which is well above the Chronic Disease Risk Reduction Intake of 2300 mg/day [8]. Given the strong influence that food companies have on the nutritional quality of the packaged food supply, the World Health Organization (WHO) recommends that food companies contribute to reducing the prevalence of obesity and diet-related NCDs by reformulating their existing products to improve their nutritional composition and/or introducing healthier products [9, 10].

Reformulation of products in the packaged food supply to improve their nutritional quality is a cost-effective policy measure for addressing the obesity and NCD burden [11–13]. Notably, reformulation is likely to have more impact on diet quality than interventions relying on behaviour changes from consumers [14–16]. Reformulation has been shown by systematic reviews of empirical and modeling studies to improve the healthfulness of food purchases, nutrient intakes and health outcomes, particularly for sodium and trans fat [12, 17]. According to the Organisation for Economic Cooperation and Development, a 20% calorie reduction in energy-dense foods high in sugars, sodium and saturated fats in Canada could prevent 387,000 NCDs between 2019 and 2050, save CAD $339 million in annual healthcare expenses, and “increase employment and productivity by the equivalent of 15 thousand full-time workers per year” [18]. Reformulation could also prevent 1.1 million annual global cases of CVDs, diabetes and cancer [19].

Previous studies in Canada and elsewhere have demonstrated that many top packaged food and beverage companies have made voluntary commitments to improve the nutritional quality of their products [20–26]. However, the timelines, breadth of coverage across their product portfolio, geographic applicability, transparency and significance of these commitments in the context of public health recommendations vary widely between companies. Many major food and beverage companies in Canada have not established SMART (specific, measurable, attainable, realistic and time-sensitive) reduction targets for sodium, saturated fat, *trans* fat, sugars and/or calories/portion sizes as of 2017 [26]. Research has also shown that many of the products offered by major food companies are of relatively poor nutritional quality [24, 25, 27–30]. In Canada, two-thirds of products offered by the leading packaged food and beverage companies were energy-dense and high in sodium, saturated fat and/or sugars in 2017 [3]. These findings are not surprising, given the evidence to suggest the limited impact of government-led voluntary product (re) formulation policies on the healthfulness of the packaged food supply [31, 32]. However, with many companies establishing their own voluntary product (re) formulation commitments – extending beyond government reformulation programs – there is a need to examine whether stronger commitments reported by individual companies translate into greater improvements in the healthfulness of their products [33]. Companies’ public reporting of their reformulation progress is often ambiguous, incomplete and measured according to companies’ own nutrient profile models or criteria, suggesting a need for independent assessments of companies’ performance concerning product (re) formulation [26]. Although the Access to Nutrition Initiative has assessed both the product (re) formulation commitments and the nutritional quality of the product portfolios of several multinational food companies [24, 25, 27], these reports have not tracked longitudinal improvements in companies’ product portfolios in relation to their reformulation targets and this work does not include data from the Canadian market. It is also not known whether some companies have reformulated or improved the nutritional quality of their product portfolios without publicly disclosing this information in their corporate reports and websites.

Thus, the purpose of this study was to investigate changes in the nutritional quality of the product portfolios of the top packaged food and beverage companies in Canada between 2013 and 2017, in relation to their voluntary product (re) formulation actions and commitments reported over a similar time period.

## Methods

### Selection of companies

Canada’s top 22 packaged food and beverage manufacturers were chosen according to their national market shares in these sectors (sourced from Euromonitor International). The study sample included companies with at least 1% of the national market share in 2016, representing a total of 51.8 and 69.8% of Canadian packaged food and beverage sales, respectively [34, 35]. Only the parent company (e.g., Coca-Cola) was analyzed when both the parent company and one of its subsidiaries had market shares ≥1% (e.g., Coca-Cola and Minute Maid). The sample consisted of 12 multinational companies based in other countries, 8 Canadian manufacturers or subsidiaries, and 2 national retailers with private-label products. The Canadian packaged food and/or beverage market shares, geographic areas served, location of corporate headquarters and types of food and beverage products offered by companies in this sample are summarized elsewhere [26].

### Assessment of product (re) formulation actions and commitments

Data on the voluntary product (re) formulation actions and commitments of the 22 packaged food and beverage companies was gathered as part of the BIA-Obesity Canada 2017 project, which is reported elsewhere [20, 36]. Briefly, information about companies’ recent actions and commitments to reduce the sodium, saturated fat, *trans* fat, sugars and energy content or portion sizes of products reported as of December 31, 2017 was collected from publicly available sources (e.g., company websites, annual and corporate social responsibility reports) and verified by representatives of the sampled companies, where possible (*n* = 11/22, 50% of companies participated).

The Food Company Reformulation (FCR) scoring tool was applied to assess and quantify the strength of the product (re) formulation actions and commitments reported by companies concerning reductions in energy/portion sizes, sodium, saturated fat, *trans* fat and (total, added or free) sugars [26]. The FCR tool assesses companies’ recent actions and commitments in terms of their breadth of application across their product portfolio, achieved or targeted magnitudes of nutrient/component reduction and their measurability and meaningfulness in relation to public health recommendations, applicability on national and global levels, and transparency in monitoring and reporting of the company’s reformulation progress. Companies only or mainly offering beverages were not evaluated on commitments or recent actions concerning sodium, saturated fat or *trans* fat, given that amounts of these nutrients/components are typically very low in such products (A. Lassonde, Canada Dry Mott’s, Coca-Cola, and Ocean Spray Cranberries). Scores for all companies were weighted, with equal weights assigned to each of the 6 domains constituting the nutrient/component scores (e.g., breadth across product portfolio, magnitude of reduction, national/global applicability) and to each nutrient/component in generating the total scores, resulting in scores out of 100% in total and for each nutrient/component. A detailed description of the FCR scoring tool is provided elsewhere [26] and the weighted scores used in this analysis are shown in **Supplementary Table 1 (**Additional File 1**)**. A summary of the validity, internal consistency and inter-rater reliability of the FCR tool is provided in Additional File 2**.**

### Food composition data

The University of Toronto Food Label Information Program (FLIP) database (described elsewhere [37, 38]) was used to examine changes in the nutritional quality of the products offered by the sampled companies in Canada between 2013 and 2017. FLIP is a database of packaged food and beverage product labels collected from four top Canadian grocery chains (including one retail outlet per chain) from May to September of 2013 (Loblaws, Metro, Safeway, Sobeys; *n* = 15,285) and three grocery chains between June and September of 2017 (Loblaws, Metro, Sobeys; Safeway was acquired by Sobeys in Fall 2013; *n* = 17,671). FLIP contains information including the name, brand, company, ingredients list, and Nutrition Facts table (NFt) displayed on a product. Products were classified based on the food categories outlined in Health Canada’s Table of Reference Amounts for Food (TRA) [39]. A comparison of the healthfulness of the products offered by these 22 companies in 2017 has been published elsewhere [3] and was compared to product healthfulness data from 2013 in this study.

### Product matching between 2013 and 2017

Company names provided on food and beverage packages were searched online to determine if they were offered by any of the sampled companies in 2013 and/or 2017. Individual products offered by the sampled companies in both 2013 and 2017 were matched by barcode or manually by identifying products with identical brand and product names, and the same or similar NFts, ingredients lists and/or product packaging. If a 2013 product was relaunched as a new product in 2017 (e.g., with a different name, brand, flavour, etc.), they were not matched.

### Products included in the sample

Products in FLIP 2013 (*n* = 8756) and 2017 (*n* = 9200) that were not offered by any of the sampled companies in either of these years were excluded from the analyses (**Supplementary Fig. 1,** Additional File 1). Infant/toddler foods (*n* = 2 in 2013; *n* = 134 in 2017), meal replacements and nutritional supplements (*n* = 34 in 2013; *n* = 36 in 2017), non-alcoholic drink mixers (*n =* 3 in 2013; *n =* 14 in 2017), sugar substitutes (*n* = 7 in 2017) and products without fibre labelling (which is required for calculating a Health Star Rating (HSR); *n =* 3 in 2017) were also excluded. The final analytic sample included a total of 14,767 unique products from FLIP 2013 (*n* = 6490) and 2017 (*n* = 8277), of which 4074 products were matched between cycles (**Supplementary Fig. 1,** Additional File 1).

### Assessment of the nutritional quality of products

The nutritional quality of products offered by the companies in this sample in 2013 and 2017 were evaluated in terms of the HSR system [40] and levels of calories, sodium, saturated fat, *trans* fat, total sugars and free sugars per 100 g (or mL). Products necessitating the addition of water or other ingredients prior to consumption were assessed according to their nutritional composition “as prepared” (based on directions for preparation provided on the product packaging) to enable comparisons with prepared products within that food category, such as: condensed soups, combination dishes needing preparation; and baking, beverage, sauce, gravy, or dessert powders and mixes. All other products in the sample were assessed based on their nutritional composition “as sold”.

#### HSR system

The HSR system is a nutrient profile (NP) model that underlies the interpretative front-of-package nutrition labelling system, developed for implementation in Australia and New Zealand [40]. This system was selected for use as a NP model in the present study as a means of summarizing the nutritional quality of foods and beverages because it encompasses both positive and negative nutrients/components. The HSR system also provides a range of scores, which is more useful for comparing the healthfulness of products offered by different companies than binary nutrient profile models that classify products as simply “healthy” or “unhealthy”. Additionally, the HSR system is a derivation of previously validated and globally-applied NP models [40, 41], and has been used in similar studies evaluating the healthfulness of products offered by packaged food and beverage manufacturers in other countries [24, 25, 27–30].

Methodology for applying the HSR system to packaged foods and beverages is described in detail elsewhere [40]. Briefly, the HSR system evaluates products based on their energy, saturated fat, sodium, total sugars content, fibre, protein, and fruits, vegetables, nuts and legumes (FVNL) contents per 100 g or 100 mL (depending on whether the NFt is listed per g or mL), with nutrient-specific thresholds and algorithms for calculating points that differ by HSR food category. Given that quantitative ingredient declarations are not required on Canadian foods and beverages, a method for estimating the FVNL contents of products using a non-quantitative ingredients list was derived by our group and applied in this study [42]. A final HSR was derived, ranging from 0.5 to 5 stars in 0.5-star increments. An HSR ≥3.5 was deemed “healthy”, based on research by the New South Wales Ministry of Health in Australia, which showed that foods with an HSR ≥3.5 are generally in agreement with Australian dietary guidelines [43]. The ≥3.5 threshold is also consistent with other studies that used the HSR system [24, 25, 27–29, 44, 45].

#### Calorie and nutrient amounts

The nutritional quality of products offered by the companies in this sample was also examined in terms of calories, sodium, saturated fat, *trans* fat, total sugars and free sugars per 100 g or 100 mL (depending on whether the relevant TRA minor food category used g or mL as the unit). Nutrients/components were examined individually to enable comparisons with nutrient/component sub-scores from the FCR scoring tool, and because they are recommended to limit in the diet and the food supply by the WHO, Health Canada and other public health authorities [46, 47]. Free sugar contents in 2013 and 2017 were approximated using an algorithm derived by Bernstein et al. [37]. Both total and free sugars contents were used since it was not possible to estimate free sugars for some Loblaw and Sobeys private-label products that included an NFt but did not display an ingredients list (e.g., desserts baked in-store; *n* = 276).

### Statistical analyses

The number and percentage of products that each company offered in 2013 but not 2017 or vice versa, and the number and percentage of the products of each company that were matched between cycles, were calculated. Absolute and percentage changes in mean HSRs and median calories, sodium, saturated fat, *trans* fat, and total and free sugars per 100 g (or mL) were evaluated in each company’s total product portfolio, by TRA food category and for matched products specifically. Changes in the HSRs of matched products were compared to those of unmatched products to examine whether observed changes in the nutritional quality of companies’ product portfolios were more likely due to reformulation (i.e., matched) versus product development or discontinuation, or acquisitions and sales of products between companies (i.e., unmatched) from 2013 to 2017.

For each company, Mann-Whitney U tests and Pearson chi-squared tests compared HSRs and the proportion of products with an HSR ≥3.5, respectively, between 2013 and 2017. Fisher’s exact test was used to compare proportions when < 5 products had an HSR ≥3.5 in 2013 or 2017. Mann-Whitney U tests were used to compare amounts of calories and nutrients per 100 g (or mL) in companies’ product portfolios between 2013 and 2017, while Wilcoxon signed-rank tests compared changes in HSRs and calorie/nutrient contents of matched products.

Generalized estimating equations (GEEs) examined the relationship between changes in the HSRs, and calorie, sodium, saturated fat, *trans* fat, and total and free sugars contents (per 100 g or mL) of companies’ products from 2013 to 2017, and their FCR tool scores. Separate models were constructed with HSR, calories, sodium, saturated fat, *trans* fat, total sugars and free sugars as outcome variables, with year (2013 or 2017) and FCR tool score (total score for the HSR model or the relevant nutrient/component sub-score for the other models) as predictors, adjusted for TRA major food category. FCR scores were standardized and entered into the models as a continuous predictor. An interaction term between year and FCR score was used to examine whether the change in HSRs or calorie/nutrient density over time differed between products offered by companies with higher versus lower FCR tool scores. Products matched between 2013 and 2017 were included in the same clusters and an exchangeable working correlation matrix was specified for all models [48]. Model types were selected based on the distribution of the data and the Quasi Likelihood under Independence Model Criterion and Corrected Quasi Likelihood under Independence Model Criterion goodness-of-fit information criteria [48]. For GEE models that used a logarithm link function (i.e., those with calories, sodium, saturated fat or free sugars as the dependent variable), a 1-unit change in a covariate (i.e., year or FCR score) multiplies the mean value of the dependent variable (e.g., calories) by exp.(β). For models with an identity link (i.e., those with HSR, trans fat or total sugars as the dependent variable), exp.(β) is the expected change in the mean of the dependent variable for each change in a covariate. For all analyses, *p*-values < 0.05 were considered statistically significant. Analyses were completed using RStudio (version 1.2.5019, RStudio Inc., Boston, MA, USA) and IBM SPSS (version 26.0, IBM Corp., Chicago, IL, USA).

## Results

### Matched, new and discontinued products

Among the total sample, 49.2% (*n* = 4074) of products in 2017 were matched to 2013, ranging from 28.0 to 72.2% of the products offered by a given company in 2017 (Table 1). Newly introduced and acquired products (i.e., collected in FLIP 2017 but not 2013) represented 50.8% (*n* = 4203) of the 2017 sample, ranging from 34.2 to 72.0% of a company’s product portfolio. Products that were discontinued or sold to another company (outside the sample) between 2013 and 2017 (i.e., included in FLIP 2013 but not 2017) constituted 37.2% of the 2013 sample (*n* = 2416), ranging from 16.9 to 57.4% between companies.

**Table 1:** The number of products offered by top packaged food and beverage companies in Canada in 2013 and 2017

Company	Total products (n)^**a**^		Matched products^**b,c**^		Products discontinued or sold to other companies^**d**^		New or acquired products^**e**^	
	2013	2017	n	%	n	%	n	%
OVERALL	6490	8277	4074	49.2	2416	37.2	4203	50.8
A. Lassonde	55	77	35	45.5	20	36.4	42	54.5
Agropur	99	123	53	43.1	46	46.5	70	56.9
Campbell Soup	197	222	116	52.3	81	41.1	106	47.7
Canada Bread	101	96	48	50.0	58	57.4	42	43.8
Canada Dry Mott’s	50	64	40	62.5	10	20.0	24	37.5
Coca-Cola	83	138	68	49.3	14	16.9	70	50.7
Danone	63	132	37	28.0	26	41.3	95	72.0
General Mills	300	382	194	50.8	106	35.3	188	49.2
George Weston	104	169	65	38.5	39	37.5	104	61.5
Kellogg	129	138	84	60.9	45	34.9	54	39.1
Kraft Heinz^f^	612	460	332	72.2	240	39.2	168	36.5
Loblaw	2260	3099	1479	47.7	781	34.6	1620	52.3
Maple Leaf Foods	178	158	90	57.0	88	49.4	68	43.0
Mondelēz	45	227	68	30.0	17	37.8	119	52.4
Nestlé	277	313	158	50.5	120	43.3	155	49.5
Ocean Spray	32	38	25	65.8	7	21.9	13	34.2
Parmalat	72	120	41	34.2	31	43.1	79	65.8
PepsiCo	267	340	199	58.5	68	25.5	141	41.5
Saputo	78	95	36	37.9	37	47.4	65	68.4
Sobeys	1248	1633	754	46.2	494	39.6	879	53.8
Sun-Rype	40	36	19	52.8	21	52.5	17	47.2
Unilever	200	217	133	61.3	67	33.5	84	38.7

^a^Data from a subset of FLIP 2017 database that includes products offered by the 22 top packaged food and beverage companies in Canada (as of 2016). ^b^Refers to products that were matched between the FLIP 2013 and 2017 datasets (based on barcode, product name, brand, company, Nutrition Facts table, ingredients list, product packaging); products may or may not have been reformulated. ^c^The percentage of products in FLIP 2017 that were matched to FLIP 2013. ^d^The number and percentage of products offered in 2013 but not 2017, such as due to discontinuation or acquisition of the product by another company; alternatively, products may have not been captured in FLIP 2017 despite being offered by one of the sampled companies at the time of data collection. ^e^The number and percentage of products in 2017 but not 2013, such as due to the introduction of new products or acquisition of products from another company; alternatively, products may have not been captured in FLIP 2013 despite being offered by one of the sampled companies at the time of data collection. ^f^In 2013, Kraft Foods and Heinz were two separate companies prior to merging in 2015 and forming The Kraft Heinz Company. However, to enable examination of changes in the nutritional quality of the company’s products over time – and because Kraft Heinz was assessed as one company using the FCR scoring tool – Kraft and Heinz were treated as a single company in 2013, as was done in similar research conducted on the Australian market [24]

### Changes in the healthfulness of companies’ products from 2013 to 2017

Absolute and percentage changes in mean HSRs from 2013 to 2017 are presented for companies’ total product portfolios in Table 2, and by matched versus unmatched products in Table 3. Similarly, changes in median calorie and nutrient amounts per 100 g/mL are presented for companies’ total product portfolios in Table 4, and for matched products in Table 5. Changes in mean HSRs and median calorie/nutrient amounts among total products in each food category are shown in Supplementary Table 2 (Additional File 1), and for matched products in Supplementary Table 3 (Additional File 1). HSRs, calorie and nutrient amounts in companies’ products in 2013 are presented by food category in Supplementary Table 4 (Additional File 1); 2017 values have been published elsewhere [3], but were used for these analyses.

**Table 2:** Changes in the mean Health Star Rating (HSR) and proportion of the total products offered by each of the sampled companies with an HSR ≥3.5 between 2013 and 2017

Company	Products (n)		Mean HSR (SD) in 2013	Mean HSR (SD) in 2017	∆Mean HSR		*p*-value^*^	Absolute ∆ in % of products with HSR ≥ 3.5 (%)	*p*-value^**^
	2013	2017			Absolute	%^**a**^			
A. Lassonde	55	77	2.3 (1.1)	2.3 (0.9)	0.0	2.0	0.091	−3.6	0.70
Agropur	99	123	3.6 (1.4)	3.6 (1.3)	0.0	0.5	0.85	5.9	0.44
Campbell Soup	197	222	3.1 (0.8)	3.2 (0.8)	0.1	3.2	0.43	−0.4	1.00
Canada Bread	101	96	3.5 (0.7)	3.0 (1.3)	− 0.4	−12.4	0.25	−14.6	**0.03**
Canada Dry Mott’s	50	64	2.3 (1.3)	2.1 (1.2)	−0.2	−9.2	0.37	−10.6	0.30
Coca-Cola	83	138	2.3 (1.4)	2.2 (1.3)	−0.2	−7.0	0.55	−4.5	0.50
Danone	63	132	3.9 (0.9)	3.5 (1.0)	−0.4	−10.1	**0.03**	−1.8	0.94
General Mills	300	382	2.9 (1.3)	2.8 (1.3)	−0.1	−5.1	0.20	−4.0	0.32
George Weston	104	169	3.3 (0.9)	3.2 (0.9)	−0.1	−3.3	0.22	−6.5	0.33
Kellogg	129	138	2.9 (1.1)	2.8 (1.1)	−0.1	−2.9	0.54	−0.8	1.00
Kraft Heinz^b^	612	460	2.2 (1.1)	2.9 (1.3)	0.7	31.1	**< 0.001**	23.6	**< 0.001**
Loblaw	2260	3099	2.9 (1.2)	3.1 (1.3)	0.2	7.5	**< 0.001**	8.9	**< 0.001**
Maple Leaf Foods	178	158	2.3 (1.1)	2.3 (1.1)	0.0	−0.1	0.91	0.7	0.99
Mondelēz	45	227	1.2 (0.9)	1.9 (1.1)	0.7	58.0	**< 0.001**	12.8	0.01*
Nestlé	277	313	2.4 (1.0)	2.4 (1.1)	0.0	0.4	0.92	−3.5	0.36
Ocean Spray	32	38	2.5 (1.0)	2.9 (1.2)	0.3	12.8	0.28	8.6	0.53*
Parmalat	72	120	3.3 (1.3)	3.0 (1.4)	−0.4	−10.8	0.091	−4.4	0.65
PepsiCo	267	340	2.7 (1.1)	2.6 (1.1)	−0.1	−3.7	0.20	0.0	1.00
Saputo	78	95	2.3 (1.6)	3.1 (1.6)	0.8	33.2	**0.007**	22.7	**0.005**
Sobeys	1248	1633	2.8 (1.2)	2.9 (1.2)	0.1	3.3	**0.02**	4.4	**0.02**
Sun-Rype	40	36	2.6 (1.0)	2.4 (0.7)	−0.2	−8.1	0.63	−11.9	0.16*
Unilever	200	217	2.8 (0.8)	2.8 (0.8)	0.0	0.3	0.93	3.0	0.57

^a^Percentage change was calculated by dividing the difference in mean HSRs between 2013 and 2017 by the mean HSR in 2013, multiplied by 100. ^*^Results of Mann-Whitney U tests; *p*-values < 0.05 were considered statistically significant (shown in boldface). ^b^In 2013, Kraft Foods and Heinz were two separate companies prior to merging in 2015 and forming The Kraft Heinz Company. However, to enable examination of changes in the nutritional quality of the company’s products over time – and because Kraft Heinz was assessed as one company using the FCR scoring tool – Kraft and Heinz were treated as a single company in 2013, as was done in similar research conducted on the Australian market [24]. ^**^Results of Pearson chi-squared tests; an asterisk indicates that the Fisher’s exact test was used instead due to small group sizes; *p*-values < 0.05 were considered statistically significant (shown in boldface)

**Table 3:** Mean and median changes in Health Star Ratings (HSRs) of matched products – and changes in mean HSRs of unmatched products – between 2013 and 2017^1^

Company	Matched products^**1**^					Unmatched products				
	Mean ∆HSR^**2**^		Median ∆HSR^**2**^		*p*-value^**3**^	Mean (SD) HSR		Change in mean HSR^**4**^		*p*-value^**5**^
	Abs.	%	Abs.	%		Products discontinued/sold to other companies (2013)	New/acquired products (2017)	Abs.	%	
A. Lassonde	−0.2	−3.2	0.0	0.0	0.27	2.0 (0.8)	2.4 (0.9)	0.4	20.0	**0.005**
Agropur	−0.2	8.9	0.0	0.0	0.35	3.2 (1.5)	3.5 (1.2)	0.3	8.7	0.44
Campbell Soup	0.0	−0.4	0.0	0.0	0.21	3.0 (0.9)	3.3 (1.0)	0.3	8.7	0.14
Canada Bread	0.0	−0.2	0.0	0.0	0.51	3.5 (0.7)	3.3 (1.3)	−0.2	−6.8	0.55
Canada Dry Mott’s	0.0	1.7	0.0	0.0	0.41	2.6 (1.3)	1.9 (1.3)	−0.7	−25.5	0.18
Coca-Cola	0.1	6.0	0.0	0.0	0.31	2.2 (1.2)	1.9 (1.0)	−0.3	−14.2	0.24
Danone	0.0	0.3	0.0	0.0	1.00	4.3 (0.9)	3.4 (1.1)	−0.8	−18.9	**0.001**
General Mills	0.0	2.0	0.0	0.0	0.10	3.3 (1.3)	2.8 (1.2)	−0.5	−16.2	**< 0.001**
George Weston	0.0	−0.5	0.0	0.0	0.89	3.5 (0.8)	3.2 (0.9)	−0.3	−8.1	0.06
Kellogg	0.0	1.5	0.0	0.0	0.24	3.0 (1.1)	2.7 (1.0)	−0.3	−10.1	0.13
Kraft Heinz^6^	0.7	44.4	0.0	0.0	**< 0.001**	2.3 (1.1)	2.7 (1.1)	0.5	20.1	**< 0.001**
Loblaw	0.1	8.8	0.0	0.0	**< 0.001**	2.7 (1.2)	3.1 (1.2)	0.4	13.3	**< 0.001**
Maple Leaf Foods	0.1	8.6	0.0	0.0	**0.03**	2.6 (1.1)	2.6 (1.2)	0.0	−0.8	0.99
Mondelēz	0.2	20.2	0.0	0.0	**< 0.001**	0.9 (0.6)	2.0 (1.2)	1.1	116.6	**< 0.001**
Nestlé	0.1	6.9	0.0	0.0	**0.003**	2.6 (1.0)	2.4 (1.0)	−0.1	−5.2	0.21
Ocean Spray	0.6	34.1	0.0	0.0	**0.04**	2.6 (1.2)	2.4 (1.0)	−0.1	−5.8	0.97
Parmalat	0.0	22.4	0.0	0.0	0.96	3.8 (1.0)	3.0 (1.5)	−0.9	−23.0	**0.004**
PepsiCo	0.0	0.9	0.0	0.0	0.72	2.7 (1.1)	2.4 (1.1)	−0.3	−9.9	**0.047**
Saputo	−0.3	−14.3	0.0	0.0	**0.04**	2.0 (1.6)	3.2 (1.6)	1.1	55.7	**0.004**
Sobeys	0.1	7.0	0.0	0.0	**< 0.001**	2.8 (1.1)	2.9 (1.3)	0.1	3.6	0.051
Sun-Rype	0.0	0.0	0.0	0.0	N/A	2.6 (1.1)	2.2 (0.4)	−0.4	−16.9	0.52
Unilever	0.1	8.2	0.0	0.0	**0.001**	2.6 (0.8)	2.5 (0.7)	−0.1	−5.6	0.09

^1^Individual products offered by the sampled companies in both 2013 and 2017 were matched either by barcode or manually by identifying products with identical brand and product names, and the same or similar Nutrition Facts tables, ingredients lists and product packaging. Unmatched products refer to those collected in either 2013 or 2017, but not both years (e.g., newly developed or acquired products, or products that were discontinued or sold to companies not included in this sample). ^2^Absolute and percentage changes were calculated based on the 2013 and 2017 HSRs of each matched product (by dividing the difference in 2013 and 2017 HSRs by the 2013 HSR, multiplied by 100), and then averaged across all products to generate mean and median absolute and percentage changes. ^3^Results of Wilcoxon signed-rank tests; *p*-values < 0.05 were considered statistically significant (shown in boldface). ^4^Absolute and percentage changes were determined based on the mean HSRs for all unmatched products in 2013 and 2017; percentage change was calculated by dividing the difference between mean HSRs in 2013 and 2017 by the mean HSR in 2013, multiplied by 100. ^5^Results of Mann-Whitney U tests; *p*-values < 0.05 were considered statistically significant (shown in boldface). ^6^In 2013, Kraft Foods and Heinz were two separate companies prior to merging in 2015 and forming The Kraft Heinz Company. However, to enable examination of changes in the nutritional quality of the company’s products over time – and because Kraft Heinz was assessed as one company using the FCR scoring tool – Kraft and Heinz were treated as a single company in 2013, as was done in similar research conducted on the Australian market [24]

**Table 4:**
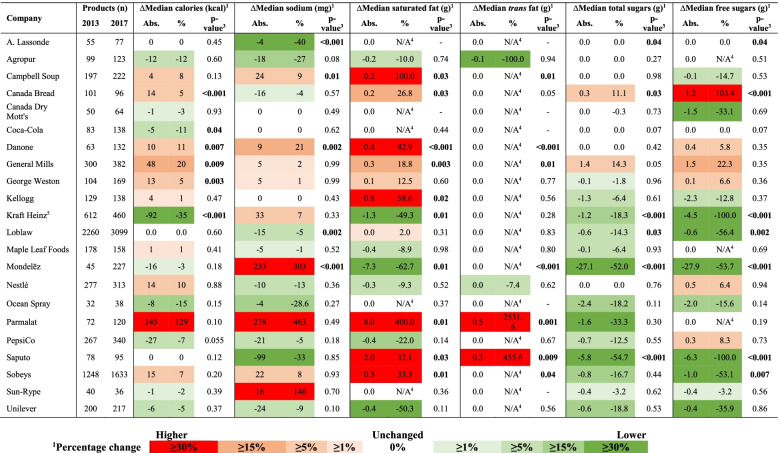
Absolute and percentage changes in median calories, sodium, saturated fat, *trans* fat, total sugars and free sugars per 100 g (or mL) in the product portfolios of the sampled companies between 2013 and 2017.^1,2^

^1^Median nutrient values in each company’s products in 2013 are presented overall and by food category in Supplementary Table 4 (Additional File 1); 2017 values have been published elsewhere [3]. ^2^Percentage change was calculated by dividing the difference in median calorie or nutrient contents between 2013 and 2017 by the median amount of calories or the nutrient in 2013, multiplied by 100. Percentage change colour scale was adapted from: Neal B, Sacks G, Shahid M, Taylor F, Huffman M. FoodSwitch: State of the Food Supply (April 2019) 2019. Available from: https://www.georgeinstitute.org/sites/default/files/food_supply_report.pdf. ^3^Results of Mann-Whitney U tests; *p*-values < 0.05 were considered statistically significant (shown in boldface). A dash (−) indicates that a p-value could not be calculated for companies that had 0 g of that nutrient in all products offered in both 2013 and 2017. ^4^N/A indicates that percentage change could not be determined when the median nutrient value per 100 g (or mL) in 2013 was 0 g. ^5^In 2013, Kraft Foods and Heinz were two separate companies prior to merging in 2015 and forming The Kraft Heinz Company. However, to enable examination of changes in the nutritional quality of the company’s products over time – and because Kraft Heinz was assessed as one company using the FCR scoring tool – Kraft and Heinz were treated as a single company in 2013, as was done in similar research conducted on the Australian market [24]

**Table 5:**
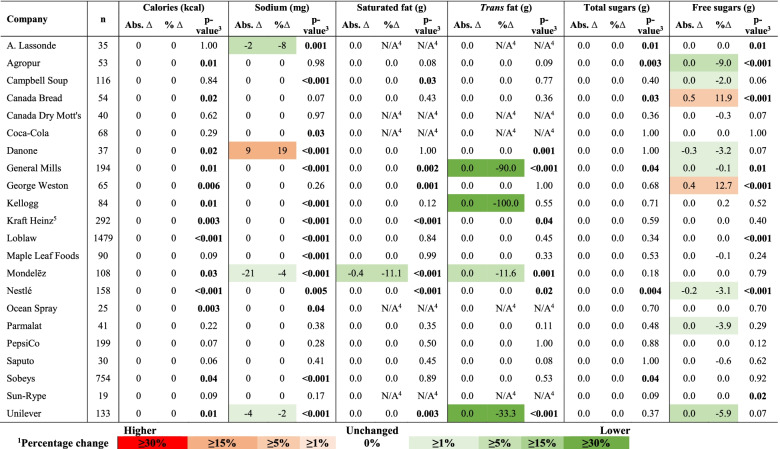
Median absolute and percentage changes in the calorie, sodium, saturated fat, *trans* fat, total sugars and free sugars contents per 100 g (or mL) for products offered by the sampled companies that were matched between 2013 and 2017.^1,2^

^1^Absolute and percentage changes were calculated based on the 2013 and 2017 cal/nutrient amount of each matched product (by dividing the difference in 2013 and 2017 cal/nutrient amount by the 2013 cal/nutrient amount, multiplied by 100), and then averaged across all products to generate median absolute and percentage changes. Percentage change colour scale was adapted from: Neal B, Sacks G, Shahid M, Taylor F, Huffman M. FoodSwitch: State of the Food Supply (April 2019) 2019. Available from: https://www.georgeinstitute.org/sites/default/files/food_supply_report.pdf. ^2^Individual products offered by the sampled companies in both 2013 and 2017 were matched either by barcode or manually by identifying products with identical brand and product names, and the same or similar Nutrition Facts tables, ingredients lists and product packaging. ^3^Results of Wilcoxon signed-rank tests; *p*-values < 0.05 were considered statistically significant (shown in boldface). ^4^N/A indicates that percentage change and p-values could not be calculated for companies whose matched products all contained 0 g of the nutrient in both 2013 and 2017. ^5^In 2013, Kraft Foods and Heinz were two separate companies prior to merging in 2015 and forming The Kraft Heinz Company. However, to enable examination of changes in the nutritional quality of the company’s products over time – and because Kraft Heinz was assessed as one company using the FCR scoring tool – Kraft and Heinz were treated as a single company in 2013, as was done in similar research conducted on the Australian market [24]

#### HSR system

Five companies (Kraft Heinz, Loblaws, Mondelēz, Saputo, Sobeys) had significantly higher HSRs and a greater proportion of products with an HSR ≥3.5 in 2017 than 2013 (Table 2). HSRs were reduced significantly in Danone products, and the proportion of products with an HSR ≥3.5 decreased for Canada Bread. Among matched products (Table 3), there were significant changes in the HSRs of products offered by 9 companies, with mean increases in the products of 7 companies and a mean decrease in 1 company’s products; however, median absolute and percentage changes in HSRs of matched products were 0.0 and 0.0%, respectively (Table 3). Unmatched products (e.g., discontinued or new) demonstrated larger absolute and percentage changes in mean HSRs; significant increases were observed for the products of 6 companies, and decreases for 4 companies (Table 3).

#### Calories per 100 g (or mL)

Between 2013 and 2017, calorie contents decreased significantly in the products of 2 companies (Coca-Cola, Kraft Heinz) and increased in the products of 4 companies (Canada Bread, Danone, General Mills, George Weston) (Table 4). For matched products, the amount of calories per 100 g (or mL) changed significantly for 13 companies, although median absolute and percentage changes were 0 kcal and 0%, respectively (Table 5).

#### Sodium per 100 g (or mL)

Sodium levels decreased significantly in the products of 2 companies (A. Lassonde, Loblaw) and increased for 3 companies (Campbell Soup, Danone, Mondelēz) (Table 4). Sodium contents of matched products decreased significantly for 3 companies (A. Lassonde, Mondelēz, Unilever) and increased in Danone products (Table 5). Ten companies had significant changes in sodium per 100 g (or mL) for matched products, with median absolute and percentage changes of 0 mg and 0%, respectively.

#### Saturated fat per 100 g (or mL)

Five companies had a median saturated fat content of 0 g in their product portfolios in 2013 and 2017 (A. Lassonde, Canada Dry Mott’s, Coca-Cola, Ocean Spray, Sun-Rype; Table 4). Saturated fat levels decreased significantly in the products of 2 companies (Kraft Heinz, Mondelēz), and increased in the products of 8 companies (Campbell Soup, Canada Bread, Danone, General Mills, Kellogg, Parmalat, Saputo, Sobeys). Saturated fat levels in matched products offered by Mondelēz decreased, and changed significantly for 6 companies despite median absolute and percentage changes of 0 g and 0%, respectively (Table 5).

#### Trans fat per 100 g (or mL)

Eighteen companies had a median *trans* fat content of 0 g in their product portfolios in 2013 and 2017 (Table 4). *Trans* fat contents increased significantly in products offered by 2 companies (Parmalat, Saputo). Five companies had significant changes in the distribution of *trans* fat but experienced no change in median *trans* fat contents per 100 g (or mL). *Trans* fat contents were reduced significantly in the matched products offered by 3 companies (General Mills, Mondelēz, Unilever) and changed significantly for 3 companies that had median absolute and percentage changes of 0 g and 0%, respectively (Table 5).

#### Total sugars per 100 g (or mL)

Total sugars levels decreased significantly in the product portfolios of 4 companies (Kraft Heinz, Loblaw, Mondelēz, Saputo), and increased in Canada Bread products (Table 4). The distribution of total sugars changed significantly in A. Lassonde products despite median absolute and percentages changes of 0 g and 0%, respectively. Total sugars in matched products changed significantly for 6 companies, yet median absolute and percentage changes were 0 g and 0%, respectively (Table 5).

#### Free sugars per 100 g (or mL)

Free sugars contents decreased significantly in the products offered by 5 companies (Kraft Heinz, Loblaw, Mondelēz, Saputo, Sobeys), and increased in Canada Bread products (Table 4). The distribution of free sugars changed significantly in A. Lassonde products, despite median absolute and percentages changes of 0 g and 0%, respectively. Among matched products, free sugars contents decreased significantly for 3 companies (Agropur, General Mills, Nestlé), increased for 2 companies (Canada Bread, George Weston), and changed significantly in A. Lassonde’s matched products but with median absolute and percentage changes of 0 g and 0%, respectively (Table 5).

### Changes in nutritional quality in relation to FCR tool scores

**Fig. 1:**
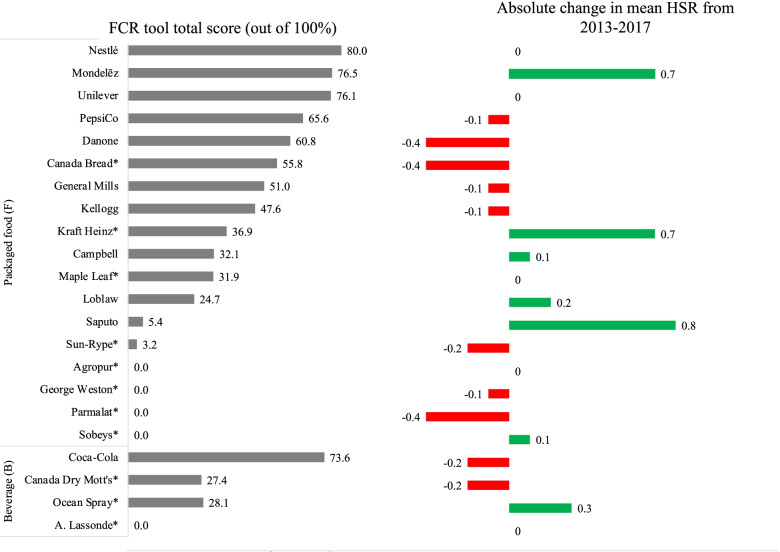
Food Company Reformulation (FCR) tool total scores and absolute changes in mean HSRs of the products offered by Canada’s top packaged food and beverage companies between 2013 and 2017. ^1^The FCR tool quantifies the strength of voluntary reported recent actions and commitments made by food companies to reduce energy/portion sizes, sodium, saturated fat, *trans* fat and (total, added or free) sugars in their products; scores were calculated out of 100%. Companies only or primarily offering beverages were not evaluated for sodium, saturated fat or *trans* fat. Health Star Ratings (HSRs) of products were examined in relation to FCR tool total scores. Details about the FCR scoring tool methodology and a complete breakdown of companies’ scores is provided elsewhere [26]. An asterisk (*) indicates that data on recent actions and commitments was based on publicly available information only since the company opted not to participate in the research process for the BIA-Obesity Canada 2017 project

**Fig. 2:**
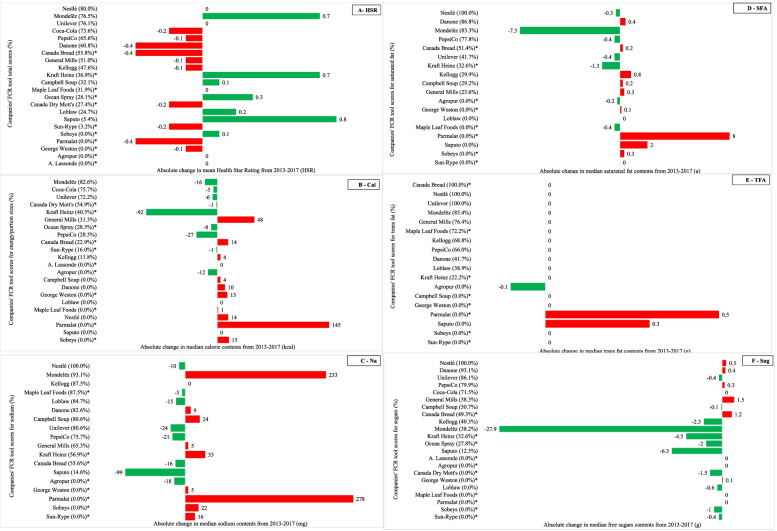
Average absolute changes in the mean Health Star Ratings (**A**), and median calorie (**B**), sodium (**C**), saturated fat (**D**), *trans* fat (**E**) and free sugars (**F**) contents per 100 g (or mL) in products offered by the sampled companies between 2013 and 2017, presented in relation to the strength of their voluntary recent actions and commitments concerning product (re) formulation as evaluated based on the Food Company Reformulation (FCR) scoring tool. ^1^The FCR tool quantifies the strength of voluntary reported recent actions and commitments made by food companies to reduce energy/portion sizes, sodium, saturated fat, *trans* fat and (total, added or free) sugars in their products; scores were calculated out of 100%. Companies only or primarily offering beverages were not evaluated for sodium, saturated fat or *trans* fat. Health Star Ratings (HSRs) of products were examined in relation to FCR tool total scores. Details about the FCR scoring tool methodology and a complete breakdown of companies’ scores is provided elsewhere [26]. ^2^Green bars depict absolute changes resulting in improved nutritional quality of products (i.e., an increase in HSR and a decrease in calorie, sodium, saturated fat, *trans* fat or free sugars contents), whereas red bars depict changes resulting in lower nutritional quality (i.e., a decrease in HSR and an increase in calorie or nutrient contents). An asterisk (*) indicates that data on recent actions and commitments was based on publicly available information only since the company opted not to participate in the research process for the BIA-Obesity Canada 2017 project

A detailed comparison of FCR scores and absolute changes in mean HSRs is provided in Fig. 1. Figure 2 shows absolute changes in mean HSRs and median calorie, sodium, saturated fat, *trans* fat and free sugars per 100 g (or mL) in companies’ product portfolios between 2013 and 2017, presented according to FCR score. GEE results are presented in Table 6 and described below.

**Table 6:** Results of generalized estimating equations examining Health Star Ratings (HSRs), and calories, sodium, saturated fat, *trans* fat, total sugars and free sugars per 100 g (or mL) in products offered by the top packaged food and beverage companies in Canada in relation to the year of collection (2013 or 2017), Food Company Reformulation (FCR) tool total scores or sub-scores for energy/portion sizes, sodium, saturated fat, *trans* fat or sugars, adjusted for food category^a, b, c, d^

			HSR^**e,k**^	Calories (kcal)^**f,l**^	Sodium (mg)^**f,l**^	Saturated fat (g)^**f,l**^	***Trans*** fat (g)^**g,k**^	Total sugars (g)^**h,k**^	Free sugars (g)^**f,l**^
Number of products included		n	14,767	14,767	14,230^b,i^	14,230^b,i^	14,230^b,i^	14,767	14,491^j^
Year^1^		*X*^2^	**124.74**	**43.42**	**117.42**	**7.57**	**25.30**	1.10	**6.91**
		*p*-value	**< 0.001**	**< 0.001**	**< 0.001**	**0.006**	**< 0.001**	0.29	**0.009**
	2017 vs. 2013	Exp(β)^e, f, g, h^	**1.095**	**0.990**	**0.945**	**1.028**	**0.963**		**0.986**
		95% CI	**1.078, 1.112**	**0.987. 0.993**	**0.935, 0.955**	**1.008, 1.049**	**0.949, 0.977**		**0.976, 0.997**
		*P*-value	**< 0.001**	**< 0.001**	**< 0.001**	**0.006**	**< 0.001**		**0.009**
FCR score^1,2^		*X*^2^	**72.09**	3.15	0.35	**6.91**	3.29	**42.44**	**56.28**
		*p*-value	**< 0.001**	0.08	0.56	**0.009**	0.07	**< 0.001**	**< 0.001**
		Exp(β)^e, f, g, h^	**0.892**			0.980		**1.545**	**1.109**
		95% CI	**0.866, 0.918**			0.949, 1.013		**1.359, 1.756**	**1.081, 1.139**
		P-value	**< 0.001**			0.23		**< 0.001**	**< 0.001**
Year*FCR score^1,2^		*X*^2^	0.42	**10.20**	2.87	**11.88**	**27.16**	3.24	3.58
		*p*-value	0.52	**0.001**	0.09	**0.001**	**< 0.001**	0.07	0.06
	2017 vs. 2013	Exp(β)^5–8^		**0.994**		**0.961**	**0.949**		
		95% CI		**0.990, 0.998**		**0.940, 0.983**	**0.931, 0.968**		
		*P*-value		**0.001**		**0.001**	**< 0.001**		

^a^The generalized estimating equation analyses tested whether higher FCR tool scores (entered as a standardized continuous variable) were associated with greater increases in HSRs (i.e., healthier) or decreases in calories, sodium, saturated fat, trans fat, total sugars or free sugars per 100 g (or mL) between 2013 and 2017. Coefficients for the “year” variable measured whether HSRs, calories or nutrient levels (per 100 g or mL) tended to be higher or lower in 2017 compared to 2013. The “FCR score” variable coefficients measured whether there was an association between FCR scores and HSRs, calories or nutrient levels in products (per 100 g or mL). The “year*FCR score” variable examined whether the change in HSRs or calorie/nutrient density over time differed between products offered by companies with higher versus lower FCR tool scores. An association between higher FCR scores and greater improvements in nutritional quality between 2013 and 2017 would be suggested if all three of the following results were observed and statistically significant (*p* < 0.05): 1) calorie or nutrient amounts were lower in 2017 than 2013 (and HSRs were higher in 2017 than 2013); 2) FCR scores were negatively associated with calorie/nutrient amounts (and positively associated with HSRs); and 3) the “year*FCR score” interaction term variable was negative for calorie/nutrient outcome models (i.e., exp.(β) < 1) and positive for the HSR outcome model (exp(β) > 1; indicating a greater change in nutritional quality in higher-scoring companies’ products between 2013 and 2017). ^b^The FCR tool quantifies the strength of voluntary recent actions and commitments reported by food companies to reduce energy/portion sizes, sodium, saturated fat, *trans* fat and (total, added or free) sugars in their products; scores were calculated out of 100%. Companies only or primarily offering beverages were not evaluated for sodium, saturated fat or *trans* fat. HSRs of products were examined in relation to FCR tool total scores. Details about the FCR scoring tool methodology and a complete breakdown of companies’ scores is provided elsewhere [26]. ^c^Food categories are defined in Health Canada’s Table of Reference Amounts for Food [39]. ^d^Boldface values indicate statistically significant tests of model effects and pairwise comparisons; p-values < 0.05 were considered significant. For all contrasts, the reference category is listed second. Pairwise contrasts are not shown for variables that did not have a significant overall effect. ^e^Based on a model with an inverse gaussian distribution and an identity link. ^f^Based on a model with a Tweedie distribution and a logarithm link. ^g^Based on a model with a normal distribution and an identity link. ^h^Based on a model with a Tweedie distribution and an identity link. ^i^Products offered by beverage companies did not receive FCR tool scores for sodium, saturated fat or sugars, resulting in the exclusion of 537 products and a smaller sample size for these models. ^j^Model excluded 276 products for which free sugars could not be estimated in the absence of an ingredients list. ^k^Exp(β) is the expected change in the mean of the dependent variable for each 1-unit change in a covariate. ^l^A 1-unit change in a covariate multiplies the mean value of the dependent variable by exp.(β)

#### HSRs and FCR tool total scores

HSRs were higher in 2017 than 2013 (exp(β) =1.095, *p* < 0.001). Higher FCR total scores were associated with lower HSRs (exp(β) =0.892, *p* < 0.001). There was no significant difference in the change in HSRs from 2013 to 2017 between products offered by companies with higher versus lower FCR total scores (*p* = 0.52).

#### Calories per 100 g (or mL) and FCR energy/portion size scores

Calories per 100 g (or mL) were lower in 2017 than 2013 (exp(β) = 0.990, *p* < 0.001). Differences in calories between 2013 and 2017 were larger among products of companies with higher FCR scores compared to the products of lower-scoring companies (exp(β) = 0.994, *p* = 0.001). However, it was unclear whether higher FCR scores were associated with larger increases or decreases in calories since the association was not significant (*p* = 0.08).

#### Sodium per 100 g (or mL) and FCR sodium scores

Sodium contents per 100 g (or mL) were lower in 2017 than 2013 (exp(β) = 0.945, *p* < 0.001). There was no significant association between FCR sodium scores and sodium contents (*p* = 0.56). There was no significant difference in the change in sodium contents from 2013 to 2017 between products offered by companies with higher versus lower FCR total scores (*p* = 0.09).

#### Saturated fat per 100 g (or mL) and FCR saturated fat scores

Saturated fat contents per 100 g (or mL) were higher in 2017 than 2013 (exp(β) = 1.028, *p* = 0.006). There was no significant association between FCR saturated fat scores and saturated fat contents (*p* = 0.23). Differences in saturated fat contents between 2013 and 2017 were smaller among products of companies with higher FCR scores for saturated fat compared to those of companies with lower scores (exp(β) = 0.961, *p* = 0.001).

#### Trans fat per 100 g (or mL) and FCR trans fat scores

*Trans* fat contents per 100 g (or mL) were lower in 2017 than 2013 (exp(β) = 0.963, *p* < 0.001). Differences in *trans* fat contents between 2013 and 2017 were larger among products of companies with higher FCR scores, compared to products of lower-scoring companies (exp(β) = 0.949, *p* < 0.001). However, it was unclear whether higher FCR scores were associated with larger increases or decreases in *trans* fat since the association was not significant (*p* = 0.07).

#### Total sugars per 100 g (or mL) and FCR sugars scores

Total sugars contents per 100 g (or mL) were not significantly different between 2013 and 2017 (*p* = 0.29). Higher FCR scores for sugars were associated with higher total sugars contents (exp(β) = 1.545, p < 0.001). There was no significant difference in the change in total sugars contents from 2013 to 2017 between products offered by companies with higher versus lower FCR total scores (*p =* 0.07).

#### Free sugars per 100 g (or mL) and FCR sugars scores

Free sugars contents per 100 g (or mL) were lower in 2017 than 2013 (exp(β) = 0.986, *p* = 0.009). Higher FCR sugars scores were associated with higher free sugars contents (exp(β) = 1.109, p < 0.001). There was no significant difference in the change in free sugars contents from 2013 to 2017 between products offered by companies with higher versus lower FCR total scores (*p* = 0.06).

## Discussion

While some of Canada’s leading food and beverage companies have recently improved the healthfulness of their products, most companies’ product portfolios did not change significantly or were less healthy in 2017 than 2013. Higher FCR scores, which identify companies with stronger commitments to improving product healthfulness, were not associated with greater improvements in HSRs, calories or any of the nutrients/components examined. Additionally, higher FCR scores were associated with lower HSRs (i.e., less healthy), and higher total and free sugars contents, while no relationship was observed between FCR scores and calories, sodium, saturated fat or *trans* fat contents. Most companies reported weak recent actions or commitments to make their products healthier, or none at all, as demonstrated by the low FCR tool scores [26]. Even among companies reporting stronger recent actions and commitments concerning product (re) formulation (i.e., higher FCR scores), larger improvements in the healthfulness of their products were not seen, suggesting a considerable degree of “over-reporting” by companies. For example, while Nestlé had the highest FCR scores in total and for each nutrient/component except energy/portion sizes, there was no significant change in the mean HSRs or median calories, sodium, saturated fat, *trans* fat or total and free sugars contents of their products from 2013 to 2017. In combination, these results suggest that most companies’ voluntary reformulation initiatives often do not result in healthier packaged foods and beverages.

Previous studies have identified important weaknesses in the voluntary actions and commitments of packaged food and beverage companies concerning product (re) formulation that limit their potential to significantly improve the healthfulness of their products or the diet quality of consumers [20–22, 24–26, 49]. Several companies in this sample had reduced or committed to reducing levels of one or more nutrients/components of concern in their products [26]. Many of these recent actions and commitments were, however, poorly defined, applicable to few food categories, affected a small (or undisclosed) proportion of the company’s sales, not expressed in relation to government or WHO reformulation targets and recommendations, applied inconsistently across national markets, and progress in meeting reformulation targets was not publicly and regularly reported [26]. Previous research suggests that food companies’ voluntary pledges are often little more than a public relations or a corporate social responsibility strategy intended to convince consumers, policymakers and shareholders that the company prioritizes nutrition and health, while also helping to circumvent government regulations concerning the nutritional quality of the packaged food supply [50–52]. Findings from this study clearly support this notion and demonstrate that many of Canada’s top food companies are failing to substantially ameliorate the healthfulness of their products, irrespective of whether they have made commitments to do so or not.

Many countries have introduced voluntary or mandatory nutrient reduction targets, with sodium and *trans* fat reformulation policies being the most common [53, 54]. In Canada, the federal government published voluntary sodium reduction targets for each food category in 2012 [55], and banned partially hydrogenated oils (PHOs) in packaged and restaurant foods in 2018 [56], following the release of voluntary targets in 2006 [57]. An assessment conducted by Health Canada in 2017 found that, on average, products in most food categories failed to meet their sodium targets, with many categories showing an increase in sodium since 2012 [31]. Our findings are consistent with these results, as only two companies significantly reduced the median sodium content of their product portfolios and three companies increased average sodium levels in their products from 2013 to 2017. In light of this lack of improvement in sodium levels, Health Canada recently released updated voluntary sodium reduction targets for 2020–2025 [58]. Although there was relatively little change in the *trans* fat content of the product portfolios of most companies in this study, nearly all had a median *trans* fat content of 0 g in 2013 and 2017, suggesting that they were already on track to comply with Health Canada’s prohibition on adding PHOs to their Canadian products by September 2018 [56, 59]. Nonetheless, in the absence of government-endorsed reformulation targets for other nutrients in Canada, the nutritional quality of the packaged food supply is largely reliant on the voluntary actions and commitments of food and beverage companies to offer healthier products. Establishing stronger voluntary nutrient reduction targets and, more importantly, ensuring that companies actually make meaningful improvements to the nutritional quality of their products, will therefore be critical in reducing intakes of nutrients/components of concern by Canadians.

Food companies can make their product portfolios healthier through reformulation of existing products, and by acquiring or discontinuing healthier and less healthy products, respectively [24, 28]. For several of the food categories examined, there were substantial differences between companies in terms of the change in nutrient composition that occurred from 2013 to 2017 (e.g., sauces, bakery products, dairy products, fats/oils; **Supplementary Table 2,** Additional File 1), demonstrating that improving the healthfulness of companies’ product offerings is possible. There was also considerable turnover in the Canadian product portfolios of these companies between 2013 and 2017, with less than two-thirds of the total products collected in 2013 being sold in 2017. There were considerably greater changes in the HSRs, calories and nutrient amounts of products that were newly introduced, discontinued, and acquired from or sold to unsampled companies between 2013 and 2017 than in those of matched products. While there were changes in median calorie and nutrient amounts in several companies’ product portfolios, median changes in calories and nutrients for matched products were zero for most companies. These differences indicate that most of the observed change in the healthfulness of companies’ product portfolios is likely due to changes to a company’s product mix (e.g., introducing or acquiring new healthier products, or discontinuing or selling off less healthy products), rather than actual reformulation of products. The former scenario may not necessarily improve the healthfulness of Canada’s overall packaged food supply; although, the acquisition of healthier products by top food companies with the most influence on the Canadian market could help ensure that these products are more accessible, available and marketed to consumers over less healthy alternatives. Nonetheless, Health Canada’s 2017 sodium monitoring data showed very few improvements overall in the Canadian packaged food supply when data were sales-weighted [31].

A strength of this study is its use of a large and highly representative sample of major packaged food and beverage companies and their Canadian product portfolios. Changes in companies’ products were assessed in terms of both a nutrient profile model and several individual nutrients/components, accounting for inherent differences in the nature of companies’ product portfolios. This work was novel in its examination of the nutritional composition of matched, newly acquired/introduced and discontinued products, which are often neglected in cross-sectional analyses of packaged food supplies. A limitation of this study is that it cannot directly assess companies’ compliance with their voluntary product (re) formulation recent actions and commitments. A compliance assessment was not feasible given the limited information made public by companies concerning their product (re) formulation actions and commitments, and since FLIP may not include all products offered by these companies in Canada in 2013 and 2017, given that data were only collected from an outlet of 3–4 top grocery retailers at one point in time [26]. Although the FCR scoring tool comprehensively evaluated and quantified the strength of companies’ reformulation actions and commitments, effectively differentiating between companies based on their performance in this area, it also has several weaknesses, which have been described in detail elsewhere [26]. In addition, since the data were not sales-weighted, it is unclear how the healthfulness of products constituting larger proportions of companies’ Canadian sales may have changed in recent years. Lastly, the policy and food supply data used in this study are from 2017 or earlier; companies may have taken further actions to improve the healthfulness of their products since then.

## Conclusions

While some top packaged food and beverage companies in Canada made their products healthier between 2013 and 2017, many companies’ product portfolios were unchanged or became significantly less healthy during this period. The product portfolios of companies reporting stronger voluntary recent actions and/or commitments concerning product (re) formulation did not undergo greater improvement in HSRs, calories or nutrients/components of public health concern, and their products were not significantly healthier. These findings suggest that company commitments to improve the nutritional quality of the packaged food supply do not signify improvements in the nutritional profile of their products, demonstrating a need for mandatory government interventions to improve the nutritional quality of the Canadian packaged food supply.

## Supplementary Information

**Additional file 1: Supplementary Table 1**. Weighted Food Company Reformulation tool scores of the top packaged food and beverage companies in Canada. **Supplementary Table 2.** Absolute and percentage changes in mean Health Star Ratings and median calories, sodium, saturated fat, trans fat, total sugars and free sugars per 100 g (or mL) from 2013 to 2017 in the total portfolio of products offered by each company, presented by food category. **Supplementary Table 3.** Mean and median absolute and percentage changes in Health Star Ratings, calories, sodium, saturated fat, trans fat, total sugars and free sugars per 100 g (or mL) in products offered by each company that were matched between 2013 and 2017, presented by food category. **Supplementary Table 4.** Mean Health Star Ratings and median amounts of calories, sodium, saturated fat, trans fat, total sugars and free sugars per 100 g (or mL) in products offered by each company in 2013, presented overall and by food category. **Supplementary Fig. 1.** The approach used to derive the sample of products examined in this study.

**Additional file 2:.** Evaluating the Validity, Internal Consistency and Inter-rater Reliability of the Food Company Reformulation (FCR) Scoring Tool.

## Data Availability

The datasets generated and/or analysed during the current study are not publicly available but are available from the corresponding author on reasonable request. The Food Label Information Program (FLIP) 2017 database is owned and maintained by Dr. Mary R. L’Abbé (the senior author) and is not publicly available; permission to access this data was granted by Dr. L’Abbé and is open to other researchers for non-commercial uses through research collaborations and data sharing agreements.

## Abbreviations

BIA-Obesity: Business Impact Assessment – Obesity and population-level nutrition
FCR: Food Company Reformulation
FLIP: Food Label Information Program
FVNL: Fruit vegetable nut and legume
GEE: Generalized estimating equation
HSR: Health Star Rating
NCD: Non-communicable disease
NFt: Nutrition Facts table
NP: Nutrient profile
TRA: Table of Reference Amounts for Food
SMART: Specific, measurable, attainable, relevant and timely
WHO: World Health Organization
