# Supplementary material for: The relationship between voluntary product (re) formulation commitments and changes in the nutritional quality of products offered by the top packaged food and beverage companies in Canada from 2013 to 2017
Source: BMC Public Health. 2022 Feb 10;22:271. doi: 10.1186/s12889-022-12683-2 (PMC8832833; doi:10.1186/s12889-022-12683-2)
Supplement: Supplementary file 1 — Additional file 1: Supplementary Table 1. Weighted Food Company Reformulation tool scores of the top packaged food and beverage companies in Canada. Supplementary Table 2. Absolute and percentage changes in mean Health Star Ratings and median calories, sodium, saturated fat, trans fat, total sugars and free sugars per 100 g (or mL) from 2013 to 2017 in the total portfolio of products offered by each company, presented by food category. Supplementary Table 3. Mean and median absolute and percentage changes in Health Star Ratings, calories, sodium, saturated fat, trans fat, total sugars and free sugars per 100 g (or mL) in products offered by each company that were matched between 2013 and 2017, presented by food category. Supplementary Table 4. Mean Health Star Ratings and median amounts of calories, sodium, saturated fat, trans fat, total sugars and free sugars per 100 g (or mL) in products offered by each company in 2013, presented overall and by food category. Supplementary Fig. 1. The approach used to derive the sample of products examined in this study. [file 12889_2022_12683_MOESM1_ESM.pdf]

Additional File 1

SUPPLEMENTARY MATERIAL

The relationship between voluntary product (re)formulation commitments and changes in the nutritional quality of products offered by the top packaged food and beverage companies in Canada from 2013-2017

Laura Vergeer, Mavra Ahmed, Lana Vanderlee, Christine Mulligan, Madyson Weippert, Beatriz Franco-Arellano, Kacie Dickinson, Jodi T. Bernstein, Marie-Ève Labonté, Mary R. L'Abbé

Table of Contents

Supplemental Table 1. Weighted Food Company Reformulation tool scores of the top packaged food and beverage companies in Canada……………………………………………………………1

Supplemental Table 2. Absolute and percentage changes in mean Health Star Ratings and median calories, sodium, saturated fat, trans fat, total sugars and free sugars per 100 g (or mL) from 2013-2017 in the total portfolio of products offered by each company, presented by food category ……..………………..……………………..……………………………………..…….. 2

Supplemental Table 3. Mean and median absolute and percentage changes in Health Star Ratings, calories, sodium, saturated fat, trans fat, total sugars and free sugars per 100 g (or mL) in products offered by each company that were matched between 2013 and 2017, presented by food category.…………..………..………………………………………………………….….… 13

Supplemental Table 4. Mean Health Star Ratings and median amounts of calories, sodium, saturated fat, trans fat, total sugars and free sugars per 100 g (or mL) in products offered by each company in 2013, presented overall and by food category…………………………………….. 34

Supplementary Figure 1. The approach used to derive the sample of products examined in this study…………………………………………………………………………………….… 45

Supplementary Table 1. Weighted Food Company Reformulation tool scores of the top packaged food and beverage companies in Canada.[1]

Company	Weighted FCR tool score (out of 100.0)[1]					
	**Total (%)**	**Energy/portion sizes (%)**	**Sodium (%)**	**Saturated fat (%)**	***Trans* fat (%)**	**Sugars (%)**
A. Lassonde[2]	0.0	0.0	N/A[2]	N/A[2]	N/A[2]	0.0
Agropur	0.0	0.0	0.0	0.0	0.0	0.0
Campbell Soup	32.1	0.0	80.6	29.2	0.0	50.7
Canada Bread	55.8	22.9	55.6	51.4	100.0	49.3
Canada Dry Mott's[2]	27.4	54.9	N/A[2]	N/A[2]	N/A[2]	0.0
Coca-Cola[2]	73.6	75.7	N/A[2]	N/A[2]	N/A[2]	71.5
Danone	60.8	0.0	82.6	86.8	41.7	93.1
General Mills	51.0	31.3	65.3	23.6	76.4	58.3
George Weston	0.0	0.0	0.0	0.0	0.0	0.0
Kellogg	47.6	11.8	87.5	29.9	68.8	40.3
Kraft Heinz	36.9	40.3	56.9	32.6	22.2	32.6
Loblaw	24.7	0.0	84.7	0.0	38.9	0.0
Maple Leaf Foods	31.9	0.0	87.5	0.0	72.2	0.0
Mondelēz	76.5	82.6	93.1	83.3	85.4	38.2
Nestlé	80.0	0.0	100.0	100.0	100.0	100.0
Ocean Spray[2]	28.1	28.5	N/A[2]	N/A[2]	N/A[2]	27.8
Parmalat	0.0	0.0	0.0	0.0	0.0	0.0
PepsiCo	65.6	28.5	75.7	77.8	66.0	79.9
Saputo	5.4	0.0	14.6	0.0	0.0	12.5
Sobeys	0.0	0.0	0.0	0.0	0.0	0.0
Sun-Rype	3.2	16.0	0.0	0.0	0.0	0.0
Unilever	76.1	72.2	80.6	41.7	100.0	86.1

[1]The FCR tool quantifies the strength of voluntary reported recent actions and commitments made by food companies to reduce energy/portion sizes, sodium, saturated fat, *trans* fat and (total, added or free) sugars in their products; details about the FCR scoring tool methodology and a complete breakdown of companies' scores is provided elsewhere (26). [2]Companies only or primarily offering beverages were not evaluated for sodium, saturated fat or *trans* fat.

Supplementary Table 2. Absolute and percentage changes in mean Health Star Ratings and median calories, sodium, saturated fat, trans fat, total sugars and free sugars per 100 g (or mL) from 2013-2017 in products offered by each company, presented by food category.[1]

			A. Lassonde	Agropur	Campbell Soup	Canada Bread	Canada Dry Mott's	Coca-Cola	Danone	General Mills	George Weston	Kellogg	Kraft Heinz	Loblaw	Maple Leaf Foods	Mondelēz	Nestlé	Ocean Spray	Parmalat	PepsiCo	Saputo	Sobeys	Sun-Rype	Unilever
**Bakery products (A)**	**Number of products**	**2013**	**0**	**2**	**23**	**78**	**0**	**0**	**0**	**60**	**103**	**68**	**130**	**308**	**0**	**11**	**1**	**0**	**0**	**53**	**27**	**194**	**2**	**0**
		**2017**	**0**	**1**	**27**	**90**	**0**	**0**	**4**	**97**	**164**	**72**	**0**	**456**	**0**	**164**	**1**	**0**	**0**	**50**	**0**	**333**	**0**	**0**
	**Mean Health Star Rating**	**Absolute change**	—	**-0.8**	**-0.1**	**-0.4**	—	—	—	**0.2**	**-0.1**	**-0.1**	—	**0.4**	—	**0.1**	**0.5**	—	—	**0.0**	—	**0.1**	—	—
		**% change**	—	**-23.1**	**-7.2**	**-11.1**	—	—	—	**10.7**	**-2.1**	**-2.6**	—	**18.0**	—	**6.1**	**100.0**	—	—	**1.9**	—	**3.4**	—	—
	**Median calories per 100 g/mL (kcal)**	**Absolute change**	—	**22.7**	**0.0**	**13.3**	—	—	—	**16.7**	**13.3**	**0.0**	—	**-28.6**	—	**-22.7**	**-26.3**	—	—	**0.0**	—	**-32.9**	—	—
		**% change**	—	**5.9**	**0.0**	**5.3**	—	—	—	**4.2**	**5.0**	**0.0**	—	**-6.7**	—	**-4.7**	**-5.0**	—	—	**0.0**	—	**-8.4**	—	—
	**Median sodium per 100 g/mL (mg)**	**Absolute change**	—	**-45.5**	**-150.0**	**-41.6**	—	—	—	**-7.7**	**5.8**	**28.3**	—	**-44.1**	—	**112.7**	**563.9**	—	—	**59.3**	—	**16.8**	—	—
		**% change**	—	**-10.0**	**-23.1**	**-10.0**	—	—	—	**-2.3**	**1.3**	**7.4**	—	**-11.5**	—	**45.1**	**714.3**	—	—	**22.0**	—	**5.0**	—	—
	**Median saturated fat per 100 g/mL (g)**	**Absolute change**	—	**1.1**	**0.0**	**0.2**	—	—	—	**-0.2**	**0.0**	**0.6**	—	**-0.6**	—	**-0.7**	**-9.5**	—	—	**0.1**	—	**0.4**	—	—
		**% change**	—	**33.3**	**0.0**	**25.0**	—	—	—	**-4.5**	**3.2**	**25.6**	—	**-16.2**	—	**-16.7**	**-51.5**	—	—	**5.0**	—	**11.4**	—	—
	**Median trans fat per 100 g/mL (g)**	**Absolute change**	—	**0.0**	**0.0**	**0.0**	—	—	—	**0.0**	**0.0**	**0.0**	—	**0.0**	—	**0.0**	**0.0**	—	—	**0.0**	—	**0.0**	—	—
		**% change**	—	**N/A**	**N/A**	**N/A**	—	—	—	**N/A**	**N/A**	**N/A**	—	**N/A**	—	**N/A**	**N/A**	—	—	**N/A**	—	**N/A**	—	—
	**Median total sugars per 100 g/mL (g)**	**Absolute change**	—	**0.0**	**12.1**	**0.6**	—	—	—	**-6.7**	**-0.4**	**-2.0**	—	**-5.5**	—	**-4.1**	**-36.8**	—	—	**-3.6**	—	**-2.9**	—	—
		**% change**	—	**0.0**	**169.2**	**21.5**	—	—	—	**-22.9**	**-9.9**	**-8.0**	—	**-25.3**	—	**-17.1**	**-100.0**	—	—	**-15.9**	—	**-12.8**	—	—
	**Median free sugars per 100 g/mL (g)**	**Absolute change**	—	**-0.3**	**12.4**	**0.9**	—	—	—	**-7.3**	**0.0**	**-2.2**	—	**-3.9**	—	**-6.6**	**-36.8**	—	—	**-3.6**	—	**-5.5**	—	—
		**% change**	—	**-9.0**	**183.4**	**56.6**	—	—	—	**-29.8**	**1.6**	**-9.5**	—	**-20.1**	—	**-27.5**	**-100.0**	—	—	**-16.8**	—	**-27.2**	—	—
Baked goods, pastries and other sweetened bakery products (A4, A5, A6, A7, A8, A9, A10, A14, A15, A17, A22, A23)	Number of products	2013	0	0	7	6	0	0	0	20	6	23	67	167	0	9	1	0	0	4	25	111	0	0
		2017	0	0	13	22	0	0	0	27	11	28	0	227	0	83	0	0	0	0	0	189	0	0
	Mean Health Star Rating	Absolute change	—	—	0.4	-0.8	—	—	—	0.2	0.1	0.0	—	0.1	—	-0.1	—	—	—	—	—	-0.1	—	—
		% change	—	—	31.3	-43.0	—	—	—	11.1	6.2	-1.9	—	5.2	—	-3.2	—	—	—	—	—	-7.3	—	—
	Median calories per 100 g/mL (kcal)	Absolute change	—	—	0.0	-37.2	—	—	—	18.2	39.0	-46.1	—	-2.3	—	-1.7	—	—	—	—	—	-8.1	—	—
		% change	—	—	0.0	-7.8	—	—	—	4.8	11.0	-11.5	—	-0.6	—	-0.4	—	—	—	—	—	-2.1	—	—
	Median sodium per 100 g/mL (mg)	Absolute change	—	—	107.7	41.6	—	—	—	-3.0	-114.7	0.0	—	-36.8	—	53.1	—	—	—	—	—	20.4	—	—
		% change	—	—	53.8	13.8	—	—	—	-0.9	-27.9	0.0	—	-12.3	—	22.1	—	—	—	—	—	7.4	—	—
	Median saturated fat per 100 g/mL (g)	Absolute change	—	—	1.9	-0.9	—	—	—	0.2	1.4	0.1	—	-0.2	—	0.5	—	—	—	—	—	0.7	—	—
		% change	—	—	20.0	-7.8	—	—	—	3.8	28.6	4.6	—	-3.3	—	7.5	—	—	—	—	—	10.3	—	—
	Median trans fat per 100 g/mL (g)	Absolute change	—	—	0.0	0.2	—	—	—	-0.2	0.0	0.0	—	0.0	—	0.0	—	—	—	—	—	0.0	—	—
		% change	—	—	N/A	N/A	—	—	—	-54.6	-2.9	N/A	—	25.0	—	N/A	—	—	—	—	—	-18.6	—	—
	Median total sugars per 100 g/mL (g)	Absolute change	—	—	-0.5	43.6	—	—	—	0.0	-9.7	-6.7	—	-1.9	—	9.2	—	—	—	—	—	1.4	—	—
		% change	—	—	-1.5	4356.3	—	—	—	0.0	-25.6	-24.0	—	-6.8	—	38.1	—	—	—	—	—	5.5	—	—
	Median free sugars per 100 g/mL (g)	Absolute change	—	—	-0.5	40.9	—	—	—	-1.1	-4.9	-8.9	—	-1.5	—	9.2	—	—	—	—	—	2.7	—	—
		% change	—	—	-1.5	6811.6	—	—	—	-5.3	-15.0	-31.8	—	-5.7	—	38.1	—	—	—	—	—	11.9	—	—
Bread (A1, A2, A3, A24)	Number of products	2013	0	0	0	72	0	0	0	11	89	0	0	40	0	0	0	0	0	0	0	25	0	0
		2017	0	0	0	68	0	0	0	13	137	0	0	112	0	0	0	0	0	0	0	98	0	0
	Mean Health Star Rating	Absolute change	—	—	—	0.1	—	—	—	0.3	0.0	—	—	0.5	—	—	—	—	—	—	—	0.1	—	—
		% change	—	—	—	3.9	—	—	—	14.2	-0.1	—	—	15.3	—	—	—	—	—	—	—	3.5	—	—
	Median calories per 100 g/mL (kcal)	Absolute change	—	—	—	10.3	—	—	—	-16.2	8.9	—	—	-17.9	—	—	—	—	—	—	—	2.2	—	—
		% change	—	—	—	4.1	—	—	—	-5.2	3.4	—	—	-6.3	—	—	—	—	—	—	—	0.8	—	—
	Median sodium per 100 g/mL (mg)	Absolute change	—	—	—	-45.4	—	—	—	-61.6	14.3	—	—	50.8	—	—	—	—	—	—	—	-22.3	—	—
		% change	—	—	—	-10.4	—	—	—	-7.8	3.2	—	—	12.7	—	—	—	—	—	—	—	-4.2	—	—
	Median saturated fat per 100 g/mL (g)	Absolute change	—	—	—	0.0	—	—	—	-0.4	0.0	—	—	-0.1	—	—	—	—	—	—	—	-0.3	—	—
		% change	—	—	—	5.6	—	—	—	-9.3	2.4	—	—	-13.8	—	—	—	—	—	—	—	-28.6	—	—
	Median trans fat per 100 g/mL (g)	Absolute change	—	—	—	0.0	—	—	—	-0.9	0.0	—	—	0.0	—	—	—	—	—	—	—	0.0	—	—
		% change	—	—	—	N/A	—	—	—	-100.0	N/A	—	—	N/A	—	—	—	—	—	—	—	N/A	—	—
	Median total sugars per 100 g/mL (g)	Absolute change	—	—	—	0.1	—	—	—	-0.3	-0.5	—	—	-1.1	—	—	—	—	—	—	—	-0.8	—	—
		% change	—	—	—	3.2	—	—	—	-4.4	-13.6	—	—	-23.7	—	—	—	—	—	—	—	-18.1	—	—
	Median free sugars per 100 g/mL (g)	Absolute change	—	—	—	0.5	—	—	—	-1.9	-0.1	—	—	-1.4	—	—	—	—	—	—	—	-0.4	—	—
		% change	—	—	—	31.8	—	—	—	-34.7	-4.3	—	—	-35.1	—	—	—	—	—	—	—	-13.5	—	—
Crackers, croutons and rice cakes (A11, A12, A13, A16, A21, A25)	Number of products	2013	0	2	16	0	0	0	0	2	8	14	63	81	0	2	0	0	0	18	0	31	0	0
		2017	0	1	14	0	0	0	0	7	13	10	0	89	0	73	1	0	0	21	0	26	0	0
	Mean Health Star Rating	Absolute change	—	-0.8	-0.2	—	—	—	—	-0.1	-0.4	0.1	—	0.3	—	-1.0	—	—	—	0.3	—	-0.1	—	—
		% change	—	-23.1	-8.9	—	—	—	—	-3.6	-21.2	3.7	—	12.6	—	-27.3	—	—	—	13.5	—	-3.9	—	—
	Median calories per 100 g/mL (kcal)	Absolute change	—	22.7	0.0	—	—	—	—	5.0	0.0	0.0	—	0.0	—	5.6	—	—	—	0.0	—	0.0	—	—
		% change	—	5.9	0.0	—	—	—	—	1.1	0.0	0.0	—	0.0	—	1.3	—	—	—	0.0	—	0.0	—	—
	Median sodium per 100 g/mL (mg)	Absolute change	—	-45.5	50.0	—	—	—	—	56.6	50.0	25.0	—	-100.0	—	188.6	—	—	—	146.6	—	-13.6	—	—
		% change	—	-10.0	6.3	—	—	—	—	13.3	5.0	3.4	—	-14.3	—	45.3	—	—	—	19.2	—	-1.7	—	—
	Median saturated fat per 100 g/mL (g)	Absolute change	—	1.1	0.3	—	—	—	—	-0.1	0.3	0.0	—	0.0	—	-0.7	—	—	—	0.0	—	0.0	—	—
		% change	—	33.3	11.1	—	—	—	—	-1.4	11.1	0.0	—	0.0	—	-32.5	—	—	—	0.0	—	0.0	—	—
	Median trans fat per 100 g/mL (g)	Absolute change	—	0.0	0.0	—	—	—	—	-0.2	0.0	0.0	—	0.0	—	0.0	—	—	—	0.0	—	0.0	—	—
		% change	—	N/A	N/A	—	—	—	—	-100.0	N/A	N/A	—	N/A	—	N/A	—	—	—	N/A	—	N/A	—	—
	Median total sugars per 100 g/mL (g)	Absolute change	—	0.0	-4.2	—	—	—	—	3.2	0.0	2.0	—	0.0	—	5.0	—	—	—	0.0	—	-3.0	—	—
		% change	—	0.0	-100.0	—	—	—	—	N/A	N/A	40.9	—	0.0	—	N/A	—	—	—	0.0	—	-60.0	—	—
	Median free sugars per 100 g/mL (g)	Absolute change	—	-0.3	-3.8	—	—	—	—	0.0	0.0	1.9	—	-0.2	—	4.3	—	—	—	-0.2	—	-4.5	—	—
		% change	—	-9.0	-100.0	—	—	—	—	N/A	N/A	43.1	—	-6.8	—	N/A	—	—	—	-3.8	—	-100.0	—	—
Grain-based, protein and energy bars (A18, A19, A20)	Number of products	2013	0	0	0	0	0	0	0	27	0	31	0	20	0	0	0	0	0	31	2	27	2	0
		2017	0	0	0	0	0	0	4	50	3	34	0	28	0	8	0	0	0	29	0	20	0	0
	Mean Health Star Rating	Absolute change	—	—	—	—	—	—	—	0.1	—	0.0	—	0.6	—	—	—	—	—	0.0	—	-0.1	—	—
		% change	—	—	—	—	—	—	—	5.7	—	-1.8	—	26.2	—	—	—	—	—	0.8	—	-5.2	—	—
	Median calories per 100 g/mL (kcal)	Absolute change	—	—	—	—	—	—	—	-11.1	—	-2.9	—	-5.5	—	—	—	—	—	-5.5	—	18.1	—	—
		% change	—	—	—	—	—	—	—	-2.5	—	-0.7	—	-1.3	—	—	—	—	—	-1.3	—	4.3	—	—
	Median sodium per 100 g/mL (mg)	Absolute change	—	—	—	—	—	—	—	19.5	—	-16.7	—	7.1	—	—	—	—	—	7.1	—	-115.4	—	—
		% change	—	—	—	—	—	—	—	7.2	—	-6.2	—	3.6	—	—	—	—	—	2.9	—	-42.9	—	—
	Median saturated fat per 100 g/mL (g)	Absolute change	—	—	—	—	—	—	—	1.4	—	3.0	—	1.6	—	—	—	—	—	0.0	—	1.6	—	—
		% change	—	—	—	—	—	—	—	33.3	—	206.6	—	43.6	—	—	—	—	—	0.0	—	72.5	—	—

			A. Lassonde	Agropur	Campbell Soup	Canada Bread	Canada Dry Mott's	Coca-Cola	Danone	General Mills	George Weston	Kellogg	Kraft Heinz	Loblaw	Maple Leaf Foods	Mondelēz	Nestlé	Ocean Spray	Parmalat	PepsiCo	Saputo	Sobeys	Sun-Rype	Unilever
	Median trans fat per	Absolute change	—	—	—	—	—	—	—	0.0	—	0.0	—	0.0	—	—	—	—	—	0.0	—	0.0	—	—
	100 g/mL (g)	% change	—	—	—	—	—	—	—	N/A	—	N/A	—	N/A	—	—	—	—	—	N/A	—	N/A	—	—
	Median total sugars	Absolute change	—	—	—	—	—	—	—	-7.0	—	1.0	—	-7.5	—	—	—	—	—	0.0	—	0.9	—	—
	per 100 g/mL (g)	% change	—	—	—	—	—	—	—	-20.3	—	3.2	—	-21.7	—	—	—	—	—	0.0	—	3.1	—	—
	Median free sugars	Absolute change	—	—	—	—	—	—	—	-10.7	—	-3.8	—	-5.6	—	—	—	—	—	-3.2	—	-0.6	—	—
	per 100 g/mL (g)	% change	—	—	—	—	—	—	—	-33.6	—	-13.6	—	-18.5	—	—	—	—	—	-14.1	—	-2.5	—	—
Beverages	Number of products	2013	1	0	1	0	16	28	0	0	0	0	44	49	0	0	42	0	0	33	1	45	0	10
(B)		2017	5	0	3	0	25	73	1	0	0	0	47	104	0	0	53	3	0	72	0	56	3	24
	Mean Health Star	Absolute change	0.8	—	0.2	—	0.0	0.0	—	—	—	—	0.3	0.2	—	—	0.3	—	—	-0.1	—	0.3	—	0.1
	Rating	% change	53.3	—	8.3	—	-0.3	-1.1	—	—	—	—	16.2	12.4	—	—	17.4	—	—	-3.9	—	24.6	—	4.7
	Median calories per	Absolute change	-21.9	—	-54.9	—	1.7	0.0	—	—	—	—	-2.0	-6.1	—	—	1.9	—	—	-0.1	—	-36.0	—	0.0
	100 g/mL (kcal)	% change	-60.9	—	-72.2	—	4.9	0.0	—	—	—	—	-100.0	-19.0	—	—	8.1	—	—	-0.2	—	-90.0	—	N/A
	Median sodium per	Absolute change	-2.0	—	-33.6	—	-2.5	-14.8	—	—	—	—	-2.0	-0.4	—	—	1.7	—	—	-9.0	—	0.0	—	0.0
	100 g/mL (mg)	% change	-100.0	—	-79.9	—	-13.9	-56.8	—	—	—	—	-33.3	-6.1	—	—	108.3	—	—	-29.0	—	0.0	—	N/A
	Median saturated	Absolute change	0.0	—	-0.6	—	0.0	0.0	—	—	—	—	0.0	0.0	—	—	0.0	—	—	0.0	—	0.0	—	0.0
	fat per 100 g/mL (g)	% change	N/A	—	-100.0	—	N/A	N/A	—	—	—	—	N/A	N/A	—	—	N/A	—	—	N/A	—	N/A	—	N/A
	Median trans fat	Absolute change	0.0	—	0.0	—	0.0	0.0	—	—	—	—	0.0	0.0	—	—	0.0	—	—	0.0	—	0.0	—	0.0
	per 100 g/mL (g)	% change	N/A	—	-100.0	—	N/A	N/A	—	—	—	—	N/A	N/A	—	—	N/A	—	—	N/A	—	N/A	—	N/A
	Median total sugars	Absolute change	-5.7	—	-8.2	—	0.6	-0.2	—	—	—	—	0.0	-2.5	—	—	-0.6	—	—	-0.1	—	-10.0	—	0.0
	per 100 g/mL (g)	% change	-64.8	—	-63.7	—	6.2	-3.9	—	—	—	—	N/A	-36.7	—	—	-13.4	—	—	-2.4	—	-100.0	—	N/A
	Median free sugars	Absolute change	-5.7	—	-8.2	—	0.6	-0.2	—	—	—	—	0.0	-4.0	—	—	-2.3	—	—	-0.1	—	-9.6	—	0.0
	per 100 g/mL (g)	% change	-64.8	—	-63.7	—	6.2	-3.9	—	—	—	—	N/A	-59.3	—	—	-52.0	—	—	-2.4	—	-100.0	—	N/A
Carbonated	Number of products	2013	1	0	1	0	16	28	0	0	0	0	33	44	0	0	28	0	0	33	1	43	0	4
and non-		2017	5	0	3	0	25	73	1	0	0	0	43	92	0	0	31	3	0	72	0	54	3	8
carbonated	Mean Health Star	Absolute change	0.8	—	0.2	—	0.0	0.0	—	—	—	—	0.2	0.2	—	—	0.5	—	—	-0.1	—	0.4	—	0.2
beverages	Rating	% change	53.3	—	8.3	—	-0.3	-1.1	—	—	—	—	14.4	10.0	—	—	27.0	—	—	-3.9	—	24.9	—	11.5
(B1)	Median calories per	Absolute change	-21.9	—	-54.9	—	1.7	0.0	—	—	—	—	-2.0	-12.1	—	—	-1.5	—	—	-0.1	—	-35.1	—	8.8
	100 g/mL (kcal)	% change	-60.9	—	-72.2	—	4.9	0.0	—	—	—	—	-100.0	-43.1	—	—	-100.0	—	—	-0.2	—	-92.1	—	82.9
	Median sodium per	Absolute change	-2.0	—	-33.6	—	-2.5	-14.8	—	—	—	—	-1.0	-0.4	—	—	0.0	—	—	-9.0	—	0.0	—	-25.5
	100 g/mL (mg)	% change	-100.0	—	-79.9	—	-13.9	-56.8	—	—	—	—	-33.3	-6.1	—	—	N/A	—	—	-29.0	—	0.0	—	-100.0
	Median saturated fat	Absolute change	0.0	—	-0.6	—	0.0	0.0	—	—	—	—	0.0	0.0	—	—	0.0	—	—	0.0	—	0.0	—	0.0
	per 100 g/mL (g)	% change	N/A	—	-100.0	—	N/A	N/A	—	—	—	—	N/A	N/A	—	—	N/A	—	—	N/A	—	N/A	—	N/A
	Median trans fat per	Absolute change	0.0	—	0.0	—	0.0	0.0	—	—	—	—	0.0	0.0	—	—	0.0	—	—	0.0	—	0.0	—	0.0
	100 g/mL (g)	% change	N/A	—	-100.0	—	N/A	N/A	—	—	—	—	N/A	N/A	—	—	N/A	—	—	N/A	—	N/A	—	N/A
	Median total sugars	Absolute change	-5.7	—	-8.2	—	0.6	-0.2	—	—	—	—	0.0	-4.1	—	—	0.0	—	—	-0.1	—	-9.6	—	2.0
	per 100 g/mL (g)	% change	-64.8	—	-63.7	—	6.2	-3.9	—	—	—	—	N/A	-63.7	—	—	N/A	—	—	-2.4	—	-100.0	—	71.5
	Median free sugars	Absolute change	-5.7	—	-8.2	—	0.6	-0.2	—	—	—	—	0.0	-5.1	—	—	0.0	—	—	-0.1	—	-9.6	—	2.0
	per 100 g/mL (g)	% change	-64.8	—	-63.7	—	6.2	-3.9	—	—	—	—	N/A	-78.9	—	—	N/A	—	—	-2.4	—	-100.0	—	71.5
Coffee, tea	Number of products	2013	0	0	0	0	0	0	0	0	0	0	11	5	0	0	14	0	0	0	0	2	0	6
and hot		2017	0	0	0	0	0	0	0	0	0	0	4	12	0	0	22	0	0	0	0	2	0	16
chocolate	Mean Health Star	Absolute change	—	—	—	—	—	—	—	—	—	—	0.3	0.5	—	—	0.1	—	—	—	—	0.0	—	0.0
(B3-B5)	Rating	% change	—	—	—	—	—	—	—	—	—	—	17.9	43.9	—	—	7.2	—	—	—	—	0.0	—	0.0
	Median calories per	Absolute change	—	—	—	—	—	—	—	—	—	—	-5.1	-26.3	—	—	-5.7	—	—	—	—	-2.9	—	0.0
	100 g/mL (kcal)	% change	—	—	—	—	—	—	—	—	—	—	-18.7	-46.0	—	—	-13.7	—	—	—	—	-4.2	—	N/A
	Median sodium per	Absolute change	—	—	—	—	—	—	—	—	—	—	-6.6	21.5	—	—	-1.0	—	—	—	—	12.9	—	0.0
	100 g/mL (mg)	% change	—	—	—	—	—	—	—	—	—	—	-14.2	35.8	—	—	-2.9	—	—	—	—	21.4	—	N/A
	Median saturated fat	Absolute change	—	—	—	—	—	—	—	—	—	—	-0.3	-0.2	—	—	-0.2	—	—	—	—	0.0	—	0.0
	per 100 g/mL (g)	% change	—	—	—	—	—	—	—	—	—	—	-25.6	-22.9	—	—	-15.6	—	—	—	—	0.0	—	N/A
	Median trans fat per	Absolute change	—	—	—	—	—	—	—	—	—	—	0.0	0.0	—	—	0.0	—	—	—	—	0.0	—	0.0
	100 g/mL (g)	% change	—	—	—	—	—	—	—	—	—	—	-36.0	N/A	—	—	N/A	—	—	—	—	N/A	—	N/A
	Median total sugars	Absolute change	—	—	—	—	—	—	—	—	—	—	0.2	-5.3	—	—	-0.6	—	—	—	—	0.6	—	0.0
	per 100 g/mL (g)	% change	—	—	—	—	—	—	—	—	—	—	16.8	-54.7	—	—	-9.8	—	—	—	—	5.1	—	N/A
	Median free sugars	Absolute change	—	—	—	—	—	—	—	—	—	—	-0.9	-4.0	—	—	-1.6	—	—	—	—	1.1	—	0.0
	per 100 g/mL (g)	% change	—	—	—	—	—	—	—	—	—	—	-79.9	-49.6	—	—	-27.4	—	—	—	—	11.2	—	N/A
Cereals and	Number of products	2013	0	0	1	13	0	0	0	21	1	45	5	141	0	0	1	0	0	33	0	54	0	9
other grain		2017	0	0	0	0	0	0	0	33	4	45	4	230	0	1	0	0	0	46	0	78	0	13
products	Mean Health Star	Absolute change	—	—	—	—	—	—	—	0.1	-1.8	0.1	0.4	0.0	—	—	—	—	—	0.1	—	0.3	—	0.0
(C)	Rating	% change	—	—	—	—	—	—	—	2.6	-43.8	3.2	18.8	0.2	—	—	—	—	—	4.3	—	7.8	—	-0.8
	Median calories per	Absolute change	—	—	—	—	—	—	—	-8.2	-23.6	0.0	0.0	-6.1	—	—	—	—	—	-21.2	—	0.6	—	-21.8
	100 g/mL (kcal)	% change	—	—	—	—	—	—	—	-2.1	-5.1	0.0	0.0	-1.7	—	—	—	—	—	-5.4	—	0.2	—	-5.5
	Median sodium per	Absolute change	—	—	—	—	—	—	—	-52.9	39.1	-113.0	-283.3	0.7	—	—	—	—	—	-47.1	—	-4.3	—	27.8
	100 g/mL (mg)	% change	—	—	—	—	—	—	—	-9.0	55.8	-29.1	-20.2	5.9	—	—	—	—	—	-10.7	—	-20.0	—	2.8
	Median saturated	Absolute change	—	—	—	—	—	—	—	0.1	0.7	0.6	-0.7	0.0	—	—	—	—	—	0.0	—	0.2	—	0.0
	fat per 100 g/mL (g)	% change	—	—	—	—	—	—	—	14.8	36.4	N/A	-100.0	11.5	—	—	—	—	—	-1.7	—	100.0	—	N/A
	Median trans fat	Absolute change	—	—	—	—	—	—	—	0.0	0.0	0.0	-0.7	0.0	—	—	—	—	—	0.0	—	0.0	—	0.0
	per 100 g/mL (g)	% change	—	—	—	—	—	—	—	N/A	N/A	N/A	-100.0	N/A	—	—	—	—	—	N/A	—	N/A	—	N/A
	Median total sugars	Absolute change	—	—	—	—	—	—	—	-1.5	-1.1	-1.8	-3.3	0.0	—	—	—	—	—	0.9	—	-0.9	—	0.4
	per 100 g/mL (g)	% change	—	—	—	—	—	—	—	-5.2	-5.0	-8.3	-33.3	0.0	—	—	—	—	—	4.4	—	-26.1	—	15.2
	Median free sugars	Absolute change	—	—	—	—	—	—	—	-1.5	-1.1	-1.7	-3.0	0.0	—	—	—	—	—	0.5	—	0.0	—	0.8
	per 100 g/mL (g)	% change	—	—	—	—	—	—	—	-6.0	-6.3	-9.0	-49.4	N/A	—	—	—	—	—	3.1	—	N/A	—	N/A
Breakfast	Number of products	2013	0	0	0	0	0	0	0	20	1	45	0	41	0	0	0	0	0	30	0	16	0	0
cereals, hot		2017	0	0	0	0	0	0	0	32	3	45	0	51	0	0	0	0	0	43	0	22	0	0
and ready-to-	Mean Health Star	Absolute change	—	—	—	—	—	—	—	0.1	-1.5	0.1	—	0.0	—	—	—	—	—	0.1	—	0.4	—	—
eat (C1-C4)	Rating	% change	—	—	—	—	—	—	—	2.1	-37.5	3.2	—	1.1	—	—	—	—	—	4.1	—	13.5	—	—
	Median calories per	Absolute change	—	—	—	—	—	—	—	-6.1	-5.5	0.0	—	-22.2	—	—	—	—	—	-22.7	—	12.1	—	—
	100 g/mL (kcal)	% change	—	—	—	—	—	—	—	-1.6	-1.2	0.0	—	-5.6	—	—	—	—	—	-5.7	—	3.2	—	—

			A. Lassonde	Agropur	Campbell Soup	Canada Bread	Canada Dry Mott's	Coca-Cola	Danone	General Mills	George Weston	Kellogg	Kraft Heinz	Loblaw	Maple Leaf Foods	Mondelēz	Nestlé	Ocean Spray	Parmalat	PepsiCo	Saputo	Sobeys	Sun-Rype	Unilever
	Median sodium per 100 g/mL (mg)	Absolute change	—	—	—	—	—	—	—	-52.3	30.0	-113.0	—	0.0	—	—	—	—	—	-47.1	—	-250.1	—	—
		% change	—	—	—	—	—	—	—	-9.2	42.9	-29.1	—	0.0	—	—	—	—	—	-10.7	—	-51.0	—	—
	Median saturated fat per 100 g/mL (g)	Absolute change	—	—	—	—	—	—	—	0.1	0.7	0.6	—	-0.1	—	—	—	—	—	-0.2	—	0.8	—	—
		% change	—	—	—	—	—	—	—	9.6	36.4	N/A	—	-10.0	—	—	—	—	—	-13.4	—	N/A	—	—
	Median trans fat per 100 g/mL (g)	Absolute change	—	—	—	—	—	—	—	0.0	0.0	0.0	—	0.0	—	—	—	—	—	0.0	—	0.0	—	—
		% change	—	—	—	—	—	—	—	N/A	N/A	N/A	—	N/A	—	—	—	—	—	N/A	—	N/A	—	—
	Median total sugars per 100 g/mL (g)	Absolute change	—	—	—	—	—	—	—	-1.5	1.6	-1.8	—	-1.8	—	—	—	—	—	-1.2	—	2.7	—	—
		% change	—	—	—	—	—	—	—	-5.0	7.4	-8.3	—	-9.1	—	—	—	—	—	-5.3	—	14.3	—	—
	Median free sugars per 100 g/mL (g)	Absolute change	—	—	—	—	—	—	—	-1.5	1.8	-1.7	—	-1.9	—	—	—	—	—	-1.0	—	0.3	—	—
		% change	—	—	—	—	—	—	—	-6.0	10.3	-9.0	—	-12.1	—	—	—	—	—	-5.3	—	1.9	—	—
Grains (e.g., rice, barley), including flavoured (C7)	Number of products	2013	0	0	0	0	0	0	0	1	0	0	0	30	0	0	1	0	0	2	0	13	0	8
		2017	0	0	0	0	0	0	0	1	0	0	0	59	0	0	0	0	0	2	0	12	0	12
	Mean Health Star Rating	Absolute change	—	—	—	—	—	—	—	0.0	—	—	—	0.0	—	—	—	—	—	0.0	—	0.4	—	0.0
		% change	—	—	—	—	—	—	—	0.0	—	—	—	0.2	—	—	—	—	—	0.0	—	13.4	—	0.0
	Median calories per 100 g/mL (kcal)	Absolute change	—	—	—	—	—	—	—	0.0	—	—	—	2.5	—	—	—	—	—	-7.1	—	0.6	—	-22.2
		% change	—	—	—	—	—	—	—	0.0	—	—	—	0.7	—	—	—	—	—	-2.1	—	0.2	—	-5.6
	Median sodium per 100 g/mL (mg)	Absolute change	—	—	—	—	—	—	—	0.0	—	—	—	0.0	—	—	—	—	—	-107.1	—	0.0	—	24.3
		% change	—	—	—	—	—	—	—	0.0	—	—	—	N/A	—	—	—	—	—	-6.1	—	N/A	—	2.4
	Median saturated fat per 100 g/mL (g)	Absolute change	—	—	—	—	—	—	—	0.0	—	—	—	0.2	—	—	—	—	—	-0.1	—	0.0	—	0.2
		% change	—	—	—	—	—	—	—	0.0	—	—	—	N/A	—	—	—	—	—	-50.0	—	N/A	—	58.3
	Median trans fat per 100 g/mL (g)	Absolute change	—	—	—	—	—	—	—	0.0	—	—	—	0.0	—	—	—	—	—	0.0	—	0.0	—	0.0
		% change	—	—	—	—	—	—	—	N/A	—	—	—	N/A	—	—	—	—	—	N/A	—	N/A	—	N/A
	Median total sugars per 100 g/mL (g)	Absolute change	—	—	—	—	—	—	—	0.0	—	—	—	0.0	—	—	—	—	—	0.0	—	0.0	—	0.4
		% change	—	—	—	—	—	—	—	0.0	—	—	—	N/A	—	—	—	—	—	0.0	—	N/A	—	13.5
	Median free sugars per 100 g/mL (g)	Absolute change	—	—	—	—	—	—	—	0.0	—	—	—	0.0	—	—	—	—	—	2.0	—	0.0	—	0.7
		% change	—	—	—	—	—	—	—	N/A	—	—	—	N/A	—	—	—	—	—	283.9	—	N/A	—	N/A
Other (C5, C6, C10, C11)	Number of products	2013	0	0	1	0	0	0	0	0	0	0	5	10	0	0	0	0	0	1	0	2	0	1
		2017	0	0	0	0	0	0	0	0	1	0	4	14	0	1	0	0	0	1	0	7	0	1
	Mean Health Star Rating	Absolute change	—	—	—	—	—	—	—	—	—	—	0.4	0.6	—	—	—	—	—	0.0	—	1.6	—	0.0
		% change	—	—	—	—	—	—	—	—	—	—	18.8	15.8	—	—	—	—	—	0.0	—	82.1	—	0.0
	Median calories per 100 g/mL (kcal)	Absolute change	—	—	—	—	—	—	—	—	—	—	0.0	-16.7	—	—	—	—	—	0.0	—	0.0	—	-71.4
		% change	—	—	—	—	—	—	—	—	—	—	0.0	-4.7	—	—	—	—	—	0.0	—	0.0	—	-20.0
	Median sodium per 100 g/mL (mg)	Absolute change	—	—	—	—	—	—	—	—	—	—	-283.3	0.0	—	—	—	—	—	0.0	—	-1466.7	—	-28.6
		% change	—	—	—	—	—	—	—	—	—	—	-20.2	N/A	—	—	—	—	—	0.0	—	-100.0	—	-100.0
	Median saturated fat per 100 g/mL (g)	Absolute change	—	—	—	—	—	—	—	—	—	—	-0.7	0.0	—	—	—	—	—	0.0	—	0.0	—	0.0
		% change	—	—	—	—	—	—	—	—	—	—	-100.0	N/A	—	—	—	—	—	0.0	—	N/A	—	N/A
	Median trans fat per 100 g/mL (g)	Absolute change	—	—	—	—	—	—	—	—	—	—	-0.7	0.0	—	—	—	—	—	0.0	—	0.0	—	0.0
		% change	—	—	—	—	—	—	—	—	—	—	-100.0	N/A	—	—	—	—	—	N/A	—	N/A	—	N/A
	Median total sugars per 100 g/mL (g)	Absolute change	—	—	—	—	—	—	—	—	—	—	-3.3	0.0	—	—	—	—	—	0.0	—	-3.3	—	0.0
		% change	—	—	—	—	—	—	—	—	—	—	-33.3	N/A	—	—	—	—	—	0.0	—	-50.0	—	0.0
	Median free sugars per 100 g/mL (g)	Absolute change	—	—	—	—	—	—	—	—	—	—	-3.0	0.0	—	—	—	—	—	0.0	—	-2.3	—	1.4
		% change	—	—	—	—	—	—	—	—	—	—	-49.4	N/A	—	—	—	—	—	N/A	—	-100.0	—	5.3
Pastas, including filled pastas, without sauce (C8)	Number of products	2013	0	0	0	13	0	0	0	0	0	0	0	60	0	0	0	0	0	0	0	23	0	0
		2017	0	0	0	0	0	0	0	0	0	0	0	106	0	0	0	0	0	0	0	37	0	0
	Mean Health Star Rating	Absolute change	—	—	—	—	—	—	—	—	—	—	—	-0.1	—	—	—	—	—	—	—	-0.1	—	—
		% change	—	—	—	—	—	—	—	—	—	—	—	-2.9	—	—	—	—	—	—	—	-1.8	—	—
	Median calories per 100 g/mL (kcal)	Absolute change	—	—	—	—	—	—	—	—	—	—	—	-7.1	—	—	—	—	—	—	—	0.0	—	—
		% change	—	—	—	—	—	—	—	—	—	—	—	-2.0	—	—	—	—	—	—	—	0.0	—	—
	Median sodium per 100 g/mL (mg)	Absolute change	—	—	—	—	—	—	—	—	—	—	—	5.9	—	—	—	—	—	—	—	5.9	—	—
		% change	—	—	—	—	—	—	—	—	—	—	—	100.0	—	—	—	—	—	—	—	N/A	—	—
	Median saturated fat per 100 g/mL (g)	Absolute change	—	—	—	—	—	—	—	—	—	—	—	0.1	—	—	—	—	—	—	—	0.2	—	—
		% change	—	—	—	—	—	—	—	—	—	—	—	24.5	—	—	—	—	—	—	—	100.0	—	—
	Median trans fat per 100 g/mL (g)	Absolute change	—	—	—	—	—	—	—	—	—	—	—	0.0	—	—	—	—	—	—	—	0.0	—	—
		% change	—	—	—	—	—	—	—	—	—	—	—	N/A	—	—	—	—	—	—	—	N/A	—	—
	Median total sugars per 100 g/mL (g)	Absolute change	—	—	—	—	—	—	—	—	—	—	—	0.0	—	—	—	—	—	—	—	-1.2	—	—
		% change	—	—	—	—	—	—	—	—	—	—	—	0.0	—	—	—	—	—	—	—	-33.3	—	—
	Median free sugars per 100 g/mL (g)	Absolute change	—	—	—	—	—	—	—	—	—	—	—	0.0	—	—	—	—	—	—	—	0.0	—	—
		% change	—	—	—	—	—	—	—	—	—	—	—	N/A	—	—	—	—	—	—	—	N/A	—	—
**Dairy products and substitutes (D)**	**Number of products**	**2013**	**0**	**97**	**1**	**0**	**0**	**0**	**63**	**77**	**0**	**3**	**93**	**151**	**2**	**0**	**18**	**0**	**62**	**0**	**40**	**78**	**0**	**0**
		**2017**	**0**	**119**	**6**	**0**	**0**	**0**	**110**	**110**	**0**	**3**	**113**	**226**	**0**	**0**	**19**	**0**	**107**	**0**	**89**	**87**	**0**	**0**
	**Mean Health Star Rating**	**Absolute change**	**—**	**0.1**	**-0.3**	**—**	**—**	**—**	**-0.3**	**-0.2**	**—**	**0.0**	**0.0**	**0.1**	**—**	**—**	**-0.2**	**—**	**-0.4**	**—**	**-0.3**	**-0.1**	**—**	**—**
		**% change**	**—**	**2.6**	**-5.6**	**—**	**—**	**—**	**-6.9**	**-4.1**	**—**	**0.0**	**-0.4**	**2.8**	**—**	**—**	**-10.5**	**—**	**-10.8**	**—**	**-8.5**	**-2.9**	**—**	**—**
	**Median calories per 100 g/mL (kcal)**	**Absolute change**	**—**	**-14.3**	**-38.7**	**—**	**—**	**—**	**0.0**	**0.0**	**—**	**0.0**	**14.3**	**14.3**	**—**	**—**	**83.3**	**—**	**53.9**	**—**	**136.4**	**0.0**	**—**	**—**
		**% change**	**—**	**-14.3**	**-44.6**	**—**	**—**	**—**	**0.0**	**0.0**	**—**	**0.0**	**5.0**	**5.0**	**—**	**—**	**55.6**	**—**	**51.8**	**—**	**140.7**	**0.0**	**—**	**—**
	**Median sodium per 100 g/mL (mg)**	**Absolute change**	**—**	**-20.7**	**-24.4**	**—**	**—**	**—**	**9.3**	**1.1**	**—**	**0.0**	**0.0**	**-5.9**	**—**	**—**	**-60.7**	**—**	**78.2**	**—**	**105.4**	**59.0**	**—**	**—**
		**% change**	**—**	**-31.0**	**-28.9**	**—**	**—**	**—**	**20.6**	**2.7**	**—**	**0.0**	**0.0**	**-1.1**	**—**	**—**	**-52.0**	**—**	**141.8**	**—**	**111.4**	**9.7**	**—**	**—**
	**Median saturated fat per 100 g/mL (g)**	**Absolute change**	**—**	**-0.2**	**-0.6**	**—**	**—**	**—**	**0.3**	**0.2**	**—**	**0.0**	**2.7**	**0.6**	**—**	**—**	**-1.3**	**—**	**2.1**	**—**	**5.5**	**1.7**	**—**	**—**
		**% change**	**—**	**-10.0**	**-82.0**	**—**	**—**	**—**	**28.6**	**20.0**	**—**	**0.0**	**20.5**	**5.0**	**—**	**—**	**-50.0**	**—**	**136.3**	**—**	**455.6**	**11.1**	**—**	**—**
	**Median trans fat per 100 g/mL (g)**	**Absolute change**	**—**	**-0.1**	**-0.1**	**—**	**—**	**—**	**0.0**	**0.0**	**—**	**0.0**	**0.0**	**0.0**	**—**	**—**	**0.0**	**—**	**0.1**	**—**	**0.3**	**0.0**	**—**	**—**
		**% change**	**—**	**-100.0**	**-100.0**	**—**	**—**	**—**	**N/A**	**N/A**	**—**	**N/A**	**7.1**	**0.0**	**—**	**—**	**N/A**	**—**	**700.0**	**—**	**983.3**	**0.0**	**—**	**—**
	**Median total sugars per 100 g/mL (g)**	**Absolute change**	**—**	**0.0**	**-4.1**	**—**	**—**	**—**	**-0.8**	**0.4**	**—**	**0.0**	**-4.4**	**0.0**	**—**	**—**	**0.2**	**—**	**-3.2**	**—**	**0.0**	**0.0**	**—**	**—**
		**% change**	**—**	**0.0**	**-37.6**	**—**	**—**	**—**	**-7.0**	**4.5**	**—**	**0.0**	**-100.0**	**N/A**	**—**	**—**	**1.6**	**—**	**-49.2**	**—**	**0.0**	**N/A**	**—**	**—**
	**Median free sugars per 100 g/mL (g)**	**Absolute change**	**—**	**0.0**	**-1.9**	**—**	**—**	**—**	**-0.4**	**0.0**	**—**	**3.2**	**0.0**	**0.0**	**—**	**—**	**3.2**	**—**	**-4.6**	**—**	**0.0**	**0.0**	**—**	**—**
		**% change**	**—**	**N/A**	**-24.0**	**—**	**—**	**—**	**-6.1**	**-0.4**	**—**	**102.1**	**N/A**	**N/A**	**—**	**—**	**30.9**	**—**	**-100.0**	**—**	**N/A**	**N/A**	**—**	**—**
Cheese (D1-D5)	Number of products	2013	0	36	0	0	0	0	0	5	0	0	87	101	0	0	0	0	23	0	17	67	0	0
		2017	0	43	0	0	0	0	0	6	0	0	105	152	0	0	0	0	53	0	44	73	0	0

			A. Lassonde	Agropur	Campbell Soup	Canada Bread	Canada Dry Mott's	Coca-Cola	Danone	General Mills	George Weston	Kellogg	Kraft Heinz	Loblaw	Maple Leaf Foods	Mondelēz	Nestlé	Ocean Spray	Parmalat	PepsiCo	Saputo	Sobeys	Sun-Rype	Unilever
	Mean Health Star Rating	Absolute change	—	-0.1	—	—	—	—	—	-0.1	—	—	0.0	0.0	—	—	—	—	-0.3	—	-0.6	-0.1	—	—
		% change	—	-3.0	—	—	—	—	—	-3.6	—	—	-1.6	1.7	—	—	—	—	-9.0	—	-15.1	-2.4	—	—
	Median calories per 100 g/mL (kcal)	Absolute change	—	16.7	—	—	—	—	—	-27.3	—	—	0.0	16.7	—	—	—	—	33.3	—	66.7	33.3	—	—
		% change	—	5.0	—	—	—	—	—	-18.7	—	—	0.0	5.0	—	—	—	—	10.0	—	25.0	10.0	—	—
	Median sodium per 100 g/mL (mg)	Absolute change	—	0.0	—	—	—	—	—	-45.5	—	—	0.0	0.0	—	—	—	—	33.3	—	-33.3	0.0	—	—
		% change	—	0.0	—	—	—	—	—	-14.7	—	—	0.0	0.0	—	—	—	—	5.0	—	-4.5	0.0	—	—
	Median saturated fat per 100 g/mL (g)	Absolute change	—	1.7	—	—	—	—	—	-3.0	—	—	2.7	0.0	—	—	—	—	0.0	—	5.0	0.0	—	—
		% change	—	11.1	—	—	—	—	—	-47.1	—	—	20.5	0.0	—	—	—	—	0.0	—	42.9	0.0	—	—
	Median trans fat per 100 g/mL (g)	Absolute change	—	0.3	—	—	—	—	—	0.2	—	—	0.2	0.0	—	—	—	—	0.0	—	0.3	0.0	—	—
		% change	—	100.0	—	—	—	—	—	N/A	—	—	25.0	5.0	—	—	—	—	0.0	—	42.9	7.1	—	—
	Median total sugars per 100 g/mL (g)	Absolute change	—	0.0	—	—	—	—	—	-0.6	—	—	0.0	0.0	—	—	—	—	0.0	—	0.0	0.0	—	—
		% change	—	N/A	—	—	—	—	—	-19.7	—	—	N/A	N/A	—	—	—	—	N/A	—	N/A	N/A	—	—
	Median free sugars per 100 g/mL (g)	Absolute change	—	0.0	—	—	—	—	—	0.0	—	—	0.0	0.0	—	—	—	—	0.0	—	0.0	0.0	—	—
		% change	—	N/A	—	—	—	—	—	N/A	—	—	N/A	N/A	—	—	—	—	N/A	—	N/A	N/A	—	—
Cream and cream substitutes (D6, D7, D8, D14)	Number of products	2013	0	10	0	0	0	0	0	4	0	0	6	8	2	0	11	0	1	0	6	6	0	0
		2017	0	4	0	0	0	0	6	3	0	0	5	13	0	0	11	0	3	0	13	7	0	0
	Mean Health Star Rating	Absolute change	—	-1.9	—	—	—	—	—	0.1	—	—	-0.4	-0.6	—	—	-1.3	—	-1.3	—	-1.2	-0.8	—	—
		% change	—	-78.7	—	—	—	—	—	6.7	—	—	-18.6	-27.3	—	—	-71.8	—	-38.1	—	-53.8	-39.3	—	—
	Median calories per 100 g/mL (kcal)	Absolute change	—	66.7	—	—	—	—	—	-66.7	—	—	-50.0	28.6	—	—	0.0	—	33.3	—	33.3	28.6	—	—
		% change	—	40.0	—	—	—	—	—	-28.6	—	—	-39.1	23.5	—	—	0.0	—	50.0	—	16.7	23.5	—	—
	Median sodium per 100 g/mL (mg)	Absolute change	—	-33.3	—	—	—	—	—	8.3	—	—	5.6	-22.6	—	—	-166.7	—	33.3	—	33.3	2.4	—	—
		% change	—	-50.0	—	—	—	—	—	20.0	—	—	100.0	-25.3	—	—	-83.3	—	33.3	—	50.0	3.7	—	—
	Median saturated fat per 100 g/mL (g)	Absolute change	—	8.3	—	—	—	—	—	-4.2	—	—	0.6	3.8	—	—	-8.7	—	2.0	—	0.0	2.0	—	—
		% change	—	166.7	—	—	—	—	—	-33.3	—	—	11.1	81.8	—	—	-86.7	—	150.0	—	0.0	42.4	—	—
	Median trans fat per 100 g/mL (g)	Absolute change	—	-0.3	—	—	—	—	—	0.3	—	—	0.0	0.0	—	—	0.0	—	0.3	—	-0.3	0.3	—	—
		% change	—	-100.0	—	—	—	—	—	N/A	—	—	N/A	N/A	—	—	N/A	—	N/A	—	-100.0	N/A	—	—
	Median total sugars per 100 g/mL (g)	Absolute change	—	-3.3	—	—	—	—	—	0.0	—	—	-3.5	-0.8	—	—	0.0	—	0.0	—	26.7	0.0	—	—
		% change	—	-50.0	—	—	—	—	—	0.0	—	—	-44.2	-14.3	—	—	0.0	—	0.0	—	400.0	0.0	—	—
	Median free sugars per 100 g/mL (g)	Absolute change	—	0.0	—	—	—	—	—	0.0	—	—	-3.5	-2.2	—	—	0.0	—	-3.3	—	27.8	0.3	—	—
		% change	—	N/A	—	—	—	—	—	N/A	—	—	-44.2	-100.0	—	—	0.0	—	-100.0	—	832.9	15.5	—	—
Milk and milk alternatives (D10, D11)	Number of products	2013	0	18	0	0	0	0	0	3	0	0	0	15	0	0	4	0	3	0	12	5	0	0
		2017	0	22	4	0	0	0	20	2	0	0	0	15	0	0	5	0	0	0	26	5	0	0
	Mean Health Star Rating	Absolute change	—	0.0	—	—	—	—	—	-0.2	—	—	—	0.7	—	—	1.9	—	—	—	0.1	0.8	—	—
		% change	—	-0.7	—	—	—	—	—	-4.0	—	—	—	23.4	—	—	102.7	—	—	—	3.1	33.3	—	—
	Median calories per 100 g/mL (kcal)	Absolute change	—	2.0	—	—	—	—	—	4.0	—	—	—	0.0	—	—	-17.5	—	—	—	-2.0	20.0	—	—
		% change	—	4.0	—	—	—	—	—	8.3	—	—	—	0.0	—	—	-21.9	—	—	—	-3.6	25.0	—	—
	Median sodium per 100 g/mL (mg)	Absolute change	—	-2.0	—	—	—	—	—	0.0	—	—	—	0.0	—	—	-7.3	—	—	—	-9.0	-68.7	—	—
		% change	—	-4.3	—	—	—	—	—	0.0	—	—	—	0.0	—	—	-12.8	—	—	—	-15.3	-41.2	—	—
	Median saturated fat per 100 g/mL (g)	Absolute change	—	0.0	—	—	—	—	—	0.4	—	—	—	-0.2	—	—	-0.9	—	—	—	0.0	-0.6	—	—
		% change	—	0.0	—	—	—	—	—	33.3	—	—	—	-33.3	—	—	-52.1	—	—	—	-1.3	-48.4	—	—
	Median trans fat per 100 g/mL (g)	Absolute change	—	0.0	—	—	—	—	—	0.0	—	—	—	0.0	—	—	0.0	—	—	—	0.0	0.0	—	—
		% change	—	N/A	—	—	—	—	—	N/A	—	—	—	N/A	—	—	N/A	—	—	—	N/A	N/A	—	—
	Median total sugars per 100 g/mL (g)	Absolute change	—	0.0	—	—	—	—	—	0.0	—	—	—	-0.4	—	—	-2.6	—	—	—	-1.4	4.9	—	—
		% change	—	0.0	—	—	—	—	—	0.0	—	—	—	-7.7	—	—	-24.4	—	—	—	-21.9	74.0	—	—
	Median free sugars per 100 g/mL (g)	Absolute change	—	0.0	—	—	—	—	—	0.0	—	—	—	0.0	—	—	-2.7	—	—	—	-1.5	-3.8	—	—
		% change	—	N/A	—	—	—	—	—	N/A	—	—	—	N/A	—	—	-44.7	—	—	—	-100.0	-100.0	—	—
Yogurt, yogurt drinks and shakes (D12, D13, D15)	Number of products	2013	0	33	1	0	0	0	63	65	0	3	0	27	0	0	3	0	35	0	5	0	0	0
		2017	0	50	2	0	0	0	84	99	0	3	3	46	0	0	3	0	51	0	6	2	0	0
	Mean Health Star Rating	Absolute change	—	0.3	-0.3	—	—	—	-0.2	-0.2	—	0.0	—	0.1	—	—	0.2	—	-0.2	—	0.7	—	—	—
		% change	—	6.9	-5.6	—	—	—	-5.3	-6.1	—	0.0	—	1.4	—	—	5.6	—	-5.1	—	28.2	—	—	—
	Median calories per 100 g/mL (kcal)	Absolute change	—	-9.0	-21.3	—	—	—	10.0	0.0	—	0.0	—	-11.8	—	—	0.0	—	10.0	—	-13.0	—	—	—
		% change	—	-10.9	-24.6	—	—	—	11.1	0.0	—	0.0	—	-12.9	—	—	0.0	—	11.1	—	-13.2	—	—	—
	Median sodium per 100 g/mL (mg)	Absolute change	—	-3.6	-31.2	—	—	—	9.3	0.0	—	0.0	—	-10.0	—	—	0.0	—	0.0	—	-7.6	—	—	—
		% change	—	-7.4	-37.0	—	—	—	20.6	0.0	—	0.0	—	-20.0	—	—	0.0	—	0.0	—	-9.8	—	—	—
	Median saturated fat per 100 g/mL (g)	Absolute change	—	-0.4	-0.3	—	—	—	0.4	0.2	—	0.0	—	0.8	—	—	0.0	—	0.2	—	0.2	—	—	—
		% change	—	-44.4	-44.9	—	—	—	42.9	20.0	—	0.0	—	N/A	—	—	0.0	—	20.0	—	19.8	—	—	—
	Median trans fat per 100 g/mL (g)	Absolute change	—	0.0	0.0	—	—	—	0.0	0.0	—	0.0	—	0.0	—	—	0.0	—	0.1	—	0.0	—	—	—
		% change	—	N/A	-50.0	—	—	—	N/A	N/A	—	N/A	—	N/A	—	—	N/A	—	N/A	—	4.8	—	—	—
	Median total sugars per 100 g/mL (g)	Absolute change	—	-1.7	-1.8	—	—	—	0.0	-0.1	—	0.0	—	-1.0	—	—	0.0	—	0.7	—	-1.4	—	—	—
		% change	—	-14.3	-16.9	—	—	—	0.0	-1.3	—	0.0	—	-10.2	—	—	0.0	—	5.6	—	-10.0	—	—	—
	Median free sugars per 100 g/mL (g)	Absolute change	—	-1.7	1.2	—	—	—	0.4	-1.0	—	3.2	—	-1.2	—	—	3.0	—	0.5	—	1.5	—	—	—
		% change	—	-20.4	15.6	—	—	—	5.8	-11.3	—	102.1	—	-18.1	—	—	29.7	—	5.2	—	13.8	—	—	—
**Desserts (E)**	**Number of products**	**2013**	**0**	**0**	**0**	**0**	**1**	**0**	**0**	**0**	**0**	**0**	**44**	**98**	**0**	**0**	**98**	**0**	**0**	**0**	**0**	**84**	**0**	**61**
		**2017**	**0**	**0**	**0**	**0**	**0**	**0**	**17**	**1**	**0**	**0**	**41**	**102**	**0**	**0**	**115**	**0**	**0**	**0**	**0**	**66**	**0**	**62**
	**Mean Health Star Rating**	**Absolute change**	—	—	—	—	—	—	—	—	—	—	**-0.1**	**-0.1**	—	—	**0.0**	—	—	—	—	**0.1**	—	**-0.1**
		**% change**	—	—	—	—	—	—	—	—	—	—	**-4.3**	**-2.0**	—	—	**1.6**	—	—	—	—	**4.2**	—	**-4.2**
	**Median calories per 100 g/mL (kcal)**	**Absolute change**	—	—	—	—	—	—	—	—	—	—	**0.0**	**14.3**	—	—	**14.0**	—	—	—	—	**-7.1**	—	**50.6**
		**% change**	—	—	—	—	—	—	—	—	—	—	**0.0**	**12.7**	—	—	**10.3**	—	—	—	—	**-6.4**	—	**39.5**
	**Median sodium per 100 g/mL (mg)**	**Absolute change**	—	—	—	—	—	—	—	—	—	—	**21.5**	**0.7**	—	—	**0.0**	—	—	—	—	**-2.0**	—	**7.0**
		**% change**	—	—	—	—	—	—	—	—	—	—	**24.4**	**1.4**	—	—	**0.0**	—	—	—	—	**-4.0**	—	**15.9**
	**Median saturated fat per 100 g/mL (g)**	**Absolute change**	—	—	—	—	—	—	—	—	—	—	**0.0**	**0.2**	—	—	**-0.2**	—	—	—	—	**-0.3**	—	**2.1**
		**% change**	—	—	—	—	—	—	—	—	—	—	**N/A**	**9.4**	—	—	**-5.3**	—	—	—	—	**-16.2**	—	**58.7**
	**Median trans fat per 100 g/mL (g)**	**Absolute change**	—	—	—	—	—	—	—	—	—	—	**0.0**	**-0.1**	—	—	**-0.1**	—	—	—	—	**0.0**	—	**0.0**
		**% change**	—	—	—	—	—	—	—	—	—	—	**N/A**	**-100.0**	—	—	**-47.0**	—	—	—	—	**0.0**	—	**38.9**
	**Median total sugars per 100 g/mL (g)**	**Absolute change**	—	—	—	—	—	—	—	—	—	—	**0.0**	**1.3**	—	—	**0.7**	—	—	—	—	**-0.8**	—	**2.4**
		**% change**	—	—	—	—	—	—	—	—	—	—	**0.0**	**9.7**	—	—	**4.7**	—	—	—	—	**-6.1**	—	**15.0**

			A. Lassonde	Agropur	Campbell Soup	Canada Bread	Canada Dry Mott's	Coca-Cola	Danone	General Mills	George Weston	Kellogg	Kraft Heinz	Loblaw	Maple Leaf Foods	Mondelēz	Nestlé	Ocean Spray	Parmalat	PepsiCo	Saputo	Sobeys	Sun-Rype	Unilever
	Median free sugars per 100 g/mL (g)	Absolute change	—	—	—	—	—	—	—	—	—	—	0.9	-0.3	—	—	0.9	—	—	—	—	-1.8	—	2.9
		% change	—	—	—	—	—	—	—	—	—	—	7.6	-2.5	—	—	7.3	—	—	—	—	-15.9	—	22.3
Dessert toppings and fillings (F)	Number of products	2013	0	0	0	0	0	0	0	10	0	0	4	14	0	0	0	0	0	0	0	8	0	0
		2017	0	0	0	0	0	0	0	5	0	0	1	14	0	0	0	0	0	0	0	6	0	0
	Mean Health Star Rating	Absolute change	—	—	—	—	—	—	—	-0.5	—	—	-0.6	0.1	—	—	—	—	—	—	—	0.0	—	—
		% change	—	—	—	—	—	—	—	-42.9	—	—	-29.4	5.1	—	—	—	—	—	—	—	-2.0	—	—
	Median calories per 100 g/mL (kcal)	Absolute change	—	—	—	—	—	—	—	18.9	—	—	25.6	-21.4	—	—	—	—	—	—	—	-61.5	—	—
		% change	—	—	—	—	—	—	—	4.7	—	—	8.0	-7.0	—	—	—	—	—	—	—	-21.6	—	—
	Median sodium per 100 g/mL (mg)	Absolute change	—	—	—	—	—	—	—	12.3	—	—	-14.7	-37.5	—	—	—	—	—	—	—	-83.6	—	—
		% change	—	—	—	—	—	—	—	6.2	—	—	-16.1	-21.2	—	—	—	—	—	—	—	-49.1	—	—
	Median saturated fat per 100 g/mL (g)	Absolute change	—	—	—	—	—	—	—	7.1	—	—	-1.5	-0.7	—	—	—	—	—	—	—	2.1	—	—
		% change	—	—	—	—	—	—	—	131.6	—	—	-100.0	-55.1	—	—	—	—	—	—	—	N/A	—	—
	Median trans fat per 100 g/mL (g)	Absolute change	—	—	—	—	—	—	—	-6.1	—	—	0.0	0.0	—	—	—	—	—	—	—	0.0	—	—
		% change	—	—	—	—	—	—	—	-100.0	—	—	N/A	N/A	—	—	—	—	—	—	—	N/A	—	—
	Median total sugars per 100 g/mL (g)	Absolute change	—	—	—	—	—	—	—	-0.4	—	—	21.5	-0.6	—	—	—	—	—	—	—	-12.8	—	—
		% change	—	—	—	—	—	—	—	-0.7	—	—	53.8	-1.5	—	—	—	—	—	—	—	-28.5	—	—
	Median free sugars per 100 g/mL (g)	Absolute change	—	—	—	—	—	—	—	-0.4	—	—	21.5	-7.7	—	—	—	—	—	—	—	-16.9	—	—
		% change	—	—	—	—	—	—	—	-0.7	—	—	53.8	-18.3	—	—	—	—	—	—	—	-37.7	—	—
Eggs and egg substitutes (G)	Number of products	2013	0	0	0	0	0	0	0	0	0	0	0	7	0	0	0	0	0	0	0	6	0	0
		2017	0	0	0	0	0	0	0	0	0	0	0	13	0	0	0	0	0	0	0	3	0	0
	Mean Health Star Rating	Absolute change	—	—	—	—	—	—	—	—	—	—	—	0.0	—	—	—	—	—	—	—	0.1	—	—
		% change	—	—	—	—	—	—	—	—	—	—	—	-0.1	—	—	—	—	—	—	—	2.1	—	—
	Median calories per 100 g/mL (kcal)	Absolute change	—	—	—	—	—	—	—	—	—	—	—	-5.9	—	—	—	—	—	—	—	0.0	—	—
		% change	—	—	—	—	—	—	—	—	—	—	—	-4.2	—	—	—	—	—	—	—	0.0	—	—
	Median sodium per 100 g/mL (mg)	Absolute change	—	—	—	—	—	—	—	—	—	—	—	0.0	—	—	—	—	—	—	—	3.3	—	—
		% change	—	—	—	—	—	—	—	—	—	—	—	0.0	—	—	—	—	—	—	—	2.6	—	—
	Median saturated fat per 100 g/mL (g)	Absolute change	—	—	—	—	—	—	—	—	—	—	—	-0.4	—	—	—	—	—	—	—	0.0	—	—
		% change	—	—	—	—	—	—	—	—	—	—	—	-13.2	—	—	—	—	—	—	—	0.0	—	—
	Median trans fat per 100 g/mL (g)	Absolute change	—	—	—	—	—	—	—	—	—	—	—	0.0	—	—	—	—	—	—	—	0.0	—	—
		% change	—	—	—	—	—	—	—	—	—	—	—	N/A	—	—	—	—	—	—	—	N/A	—	—
	Median total sugars per 100 g/mL (g)	Absolute change	—	—	—	—	—	—	—	—	—	—	—	0.0	—	—	—	—	—	—	—	0.0	—	—
		% change	—	—	—	—	—	—	—	—	—	—	—	N/A	—	—	—	—	—	—	—	N/A	—	—
	Median free sugars per 100 g/mL (g)	Absolute change	—	—	—	—	—	—	—	—	—	—	—	0.0	—	—	—	—	—	—	—	0.0	—	—
		% change	—	—	—	—	—	—	—	—	—	—	—	N/A	—	—	—	—	—	—	—	N/A	—	—
Fats and oils (H)	Number of products	2013	0	0	0	1	0	0	0	0	0	0	101	105	1	0	0	0	9	0	0	56	0	16
		2017	0	3	7	1	0	0	0	0	0	0	85	130	0	0	0	0	13	0	2	69	0	21
	Mean Health Star Rating	Absolute change	—	—	—	0.0	—	—	—	—	—	—	2.6	1.3	—	—	—	—	-0.5	—	—	1.4	—	0.7
		% change	—	—	—	0.0	—	—	—	—	—	—	141.2	53.5	—	—	—	—	-26.6	—	—	61.0	—	24.1
	Median calories per 100 g/mL (kcal)	Absolute change	—	—	—	0.0	—	—	—	—	—	—	0.0	0.0	—	—	—	—	0.0	—	—	-33.3	—	33.3
		% change	—	—	—	0.0	—	—	—	—	—	—	0.0	0.0	—	—	—	—	0.0	—	—	-6.7	—	5.3
	Median sodium per 100 g/mL (mg)	Absolute change	—	—	—	0.0	—	—	—	—	—	—	-66.7	-100.0	—	—	—	—	-50.0	—	—	-25.0	—	-33.3
		% change	—	—	—	N/A	—	—	—	—	—	—	-7.4	-16.7	—	—	—	—	-8.3	—	—	-3.8	—	-5.0
	Median saturated fat per 100 g/mL (g)	Absolute change	—	—	—	0.0	—	—	—	—	—	—	0.0	0.0	—	—	—	—	15.0	—	—	0.0	—	0.0
		% change	—	—	—	0.0	—	—	—	—	—	—	0.0	0.0	—	—	—	—	42.9	—	—	0.0	—	0.0
	Median trans fat per 100 g/mL (g)	Absolute change	—	—	—	0.0	—	—	—	—	—	—	0.0	0.0	—	—	—	—	0.0	—	—	0.0	—	0.0
		% change	—	—	—	0.0	—	—	—	—	—	—	N/A	N/A	—	—	—	—	0.0	—	—	N/A	—	N/A
	Median total sugars per 100 g/mL (g)	Absolute change	—	—	—	0.0	—	—	—	—	—	—	0.0	0.0	—	—	—	—	0.0	—	—	0.0	—	0.0
		% change	—	—	—	N/A	—	—	—	—	—	—	0.0	N/A	—	—	—	—	N/A	—	—	N/A	—	N/A
	Median free sugars per 100 g/mL (g)	Absolute change	—	—	—	0.0	—	—	—	—	—	—	0.7	0.0	—	—	—	—	0.0	—	—	0.0	—	0.0
		% change	—	—	—	N/A	—	—	—	—	—	—	11.1	N/A	—	—	—	—	N/A	—	—	N/A	—	N/A
Butter, margarine, shortening, lard, etc. (H1)	Number of products	2013	0	0	0	1	0	0	0	0	0	0	0	19	0	0	0	0	9	0	0	12	0	12
		2017	0	3	0	1	0	0	0	0	0	0	0	18	0	0	0	0	13	0	2	13	0	10
	Mean Health Star Rating	Absolute change	—	—	—	0.0	—	—	—	—	—	—	—	-0.4	—	—	—	—	-0.5	—	—	-0.9	—	0.0
		% change	—	—	—	0.0	—	—	—	—	—	—	—	-17.9	—	—	—	—	-26.6	—	—	-45.0	—	0.2
	Median calories per 100 g/mL (kcal)	Absolute change	—	—	—	0.0	—	—	—	—	—	—	—	0.0	—	—	—	—	0.0	—	—	0.0	—	0.0
		% change	—	—	—	0.0	—	—	—	—	—	—	—	0.0	—	—	—	—	0.0	—	—	0.0	—	0.0
	Median sodium per 100 g/mL (mg)	Absolute change	—	—	—	0.0	—	—	—	—	—	—	—	-225.0	—	—	—	—	-50.0	—	—	-150.0	—	0.0
		% change	—	—	—	N/A	—	—	—	—	—	—	—	-37.5	—	—	—	—	-8.3	—	—	-25.0	—	0.0
	Median saturated fat per 100 g/mL (g)	Absolute change	—	—	—	0.0	—	—	—	—	—	—	—	22.5	—	—	—	—	15.0	—	—	17.5	—	0.0
		% change	—	—	—	0.0	—	—	—	—	—	—	—	150.0	—	—	—	—	42.9	—	—	77.8	—	0.0
	Median trans fat per 100 g/mL (g)	Absolute change	—	—	—	0.0	—	—	—	—	—	—	—	-1.0	—	—	—	—	0.0	—	—	0.5	—	0.0
		% change	—	—	—	0.0	—	—	—	—	—	—	—	-100.0	—	—	—	—	0.0	—	—	33.3	—	N/A
	Median total sugars per 100 g/mL (g)	Absolute change	—	—	—	0.0	—	—	—	—	—	—	—	0.0	—	—	—	—	0.0	—	—	0.0	—	0.0
		% change	—	—	—	N/A	—	—	—	—	—	—	—	N/A	—	—	—	—	N/A	—	—	N/A	—	N/A
	Median free sugars per 100 g/mL (g)	Absolute change	—	—	—	0.0	—	—	—	—	—	—	—	0.0	—	—	—	—	0.0	—	—	0.0	—	0.0
		% change	—	—	—	N/A	—	—	—	—	—	—	—	N/A	—	—	—	—	N/A	—	—	N/A	—	N/A
Mayonnaise and salad dressings (H4, H5)	Number of products	2013	0	0	0	0	0	0	0	0	0	0	101	50	1	0	0	0	0	0	0	31	0	4
		2017	0	0	7	0	0	0	0	0	0	0	85	64	0	0	0	0	0	0	0	44	0	9
	Mean Health Star Rating	Absolute change	—	—	—	—	—	—	—	—	—	—	2.6	2.7	—	—	—	—	—	—	—	2.6	—	2.4
		% change	—	—	—	—	—	—	—	—	—	—	141.2	168.1	—	—	—	—	—	—	—	159.5	—	163.0
	Median calories per 100 g/mL (kcal)	Absolute change	—	—	—	—	—	—	—	—	—	—	0.0	0.0	—	—	—	—	—	—	—	-2.3	—	142.9
		% change	—	—	—	—	—	—	—	—	—	—	0.0	0.0	—	—	—	—	—	—	—	-0.6	—	28.6
	Median sodium per 100 g/mL (mg)	Absolute change	—	—	—	—	—	—	—	—	—	—	-66.7	-100.0	—	—	—	—	—	—	—	-66.7	—	-71.4
		% change	—	—	—	—	—	—	—	—	—	—	-7.4	-12.5	—	—	—	—	—	—	—	-8.3	—	-9.5
	Median saturated fat per 100 g/mL (g)	Absolute change	—	—	—	—	—	—	—	—	—	—	0.0	0.0	—	—	—	—	—	—	—	0.0	—	2.0
		% change	—	—	—	—	—	—	—	—	—	—	0.0	0.0	—	—	—	—	—	—	—	0.0	—	42.9

			A. Lassonde	Agropur	Campbell Soup	Canada Bread	Canada Dry Mott's	Coca-Cola	Danone	General Mills	George Weston	Kellogg	Kraft Heinz	Loblaw	Maple Leaf Foods	Mondelēz	Nestlé	Ocean Spray	Parmalat	PepsiCo	Saputo	Sobeys	Sun-Rype	Unilever
	Median trans fat per 100 g/mL (g)	Absolute change	—	—	—	—	—	—	—	—	—	—	0.0	0.0	—	—	—	—	—	—	—	0.0	—	0.0
		% change	—	—	—	—	—	—	—	—	—	—	N/A	N/A	—	—	—	—	—	—	—	N/A	—	N/A
	Median total sugars per 100 g/mL (g)	Absolute change	—	—	—	—	—	—	—	—	—	—	0.0	0.0	—	—	—	—	—	—	—	0.0	—	0.0
		% change	—	—	—	—	—	—	—	—	—	—	0.0	0.0	—	—	—	—	—	—	—	0.0	—	N/A
	Median free sugars per 100 g/mL (g)	Absolute change	—	—	—	—	—	—	—	—	—	—	0.7	0.0	—	—	—	—	—	—	—	-0.1	—	0.0
		% change	—	—	—	—	—	—	—	—	—	—	11.1	-0.6	—	—	—	—	—	—	—	-1.6	—	N/A
Oils (H2, H6)	Number of products	2013	0	0	0	0	0	0	0	0	0	0	0	36	0	0	0	0	0	0	0	13	0	0
		2017	0	0	0	0	0	0	0	0	0	0	0	48	0	0	0	0	0	0	0	12	0	2
	Mean Health Star Rating	Absolute change	—	—	—	—	—	—	—	—	—	—	—	0.1	—	—	—	—	—	—	—	0.1	—	—
		% change	—	—	—	—	—	—	—	—	—	—	—	1.9	—	—	—	—	—	—	—	3.1	—	—
	Median calories per 100 g/mL (kcal)	Absolute change	—	—	—	—	—	—	—	—	—	—	—	0.0	—	—	—	—	—	—	—	0.0	—	—
		% change	—	—	—	—	—	—	—	—	—	—	—	0.0	—	—	—	—	—	—	—	0.0	—	—
	Median sodium per 100 g/mL (mg)	Absolute change	—	—	—	—	—	—	—	—	—	—	—	0.0	—	—	—	—	—	—	—	0.0	—	—
		% change	—	—	—	—	—	—	—	—	—	—	—	N/A	—	—	—	—	—	—	—	N/A	—	—
	Median saturated fat per 100 g/mL (g)	Absolute change	—	—	—	—	—	—	—	—	—	—	—	-2.5	—	—	—	—	—	—	—	-2.5	—	—
		% change	—	—	—	—	—	—	—	—	—	—	—	-20.0	—	—	—	—	—	—	—	-16.7	—	—
	Median trans fat per 100 g/mL (g)	Absolute change	—	—	—	—	—	—	—	—	—	—	—	0.0	—	—	—	—	—	—	—	0.0	—	—
		% change	—	—	—	—	—	—	—	—	—	—	—	N/A	—	—	—	—	—	—	—	N/A	—	—
	Median total sugars per 100 g/mL (g)	Absolute change	—	—	—	—	—	—	—	—	—	—	—	0.0	—	—	—	—	—	—	—	0.0	—	—
		% change	—	—	—	—	—	—	—	—	—	—	—	N/A	—	—	—	—	—	—	—	N/A	—	—
	Median free sugars per 100 g/mL (g)	Absolute change	—	—	—	—	—	—	—	—	—	—	—	0.0	—	—	—	—	—	—	—	0.0	—	—
		% change	—	—	—	—	—	—	—	—	—	—	—	N/A	—	—	—	—	—	—	—	N/A	—	—
**Marine and fresh water animals (I)**	**Number of products**	**2013**	0	0	0	0	0	0	0	0	0	0	0	64	0	0	0	0	0	0	0	35	0	0
		**2017**	0	0	0	0	0	0	0	0	0	0	0	87	0	0	0	0	0	0	0	48	0	0
	**Mean Health Star Rating**	**Absolute change**	—	—	—	—	—	—	—	—	—	—	—	-0.2	—	—	—	—	—	—	—	0.1	—	—
		**% change**	—	—	—	—	—	—	—	—	—	—	—	-4.5	—	—	—	—	—	—	—	2.1	—	—
	**Median calories per 100 g/mL (kcal)**	**Absolute change**	—	—	—	—	—	—	—	—	—	—	—	0.6	—	—	—	—	—	—	—	8.1	—	—
		**% change**	—	—	—	—	—	—	—	—	—	—	—	0.6	—	—	—	—	—	—	—	8.1	—	—
	**Median sodium per 100 g/mL (mg)**	**Absolute change**	—	—	—	—	—	—	—	—	—	—	—	59.8	—	—	—	—	—	—	—	16.9	—	—
		**% change**	—	—	—	—	—	—	—	—	—	—	—	20.1	—	—	—	—	—	—	—	5.1	—	—
	**Median saturated fat per 100 g/mL (g)**	**Absolute change**	—	—	—	—	—	—	—	—	—	—	—	0.1	—	—	—	—	—	—	—	0.2	—	—
		**% change**	—	—	—	—	—	—	—	—	—	—	—	16.6	—	—	—	—	—	—	—	66.7	—	—
	**Median trans fat per 100 g/mL (g)**	**Absolute change**	—	—	—	—	—	—	—	—	—	—	—	0.0	—	—	—	—	—	—	—	0.0	—	—
		**% change**	—	—	—	—	—	—	—	—	—	—	—	N/A	—	—	—	—	—	—	—	N/A	—	—
	**Median total sugars per 100 g/mL (g)**	**Absolute change**	—	—	—	—	—	—	—	—	—	—	—	0.0	—	—	—	—	—	—	—	0.0	—	—
		**% change**	—	—	—	—	—	—	—	—	—	—	—	N/A	—	—	—	—	—	—	—	N/A	—	—
	**Median free sugars per 100 g/mL (g)**	**Absolute change**	—	—	—	—	—	—	—	—	—	—	—	0.0	—	—	—	—	—	—	—	0.0	—	—
		**% change**	—	—	—	—	—	—	—	—	—	—	—	N/A	—	—	—	—	—	—	—	N/A	—	—
**Fruit and fruit juices (J)**	**Number of products**	**2013**	52	0	20	0	25	55	0	0	0	0	8	155	0	0	0	30	0	23	5	120	25	0
		**2017**	71	0	11	0	25	65	0	0	0	0	9	201	0	0	0	32	0	24	0	126	18	0
	**Mean Health Star Rating**	**Absolute change**	0.0	—	0.2	—	0.1	0.0	—	—	—	—	0.0	0.3	—	—	—	0.4	—	-0.7	—	0.2	-0.2	—
		**% change**	0.5	—	10.0	—	3.7	1.4	—	—	—	—	1.0	10.3	—	—	—	16.6	—	-16.0	—	7.9	-6.3	—
	**Median calories per 100 g/mL (kcal)**	**Absolute change**	0.0	—	0.0	—	0.0	0.0	—	—	—	—	0.0	-4.0	—	—	—	-6.0	—	1.3	—	-2.0	0.0	—
		**% change**	0.0	—	0.0	—	0.0	0.0	—	—	—	—	0.0	-7.7	—	—	—	-12.0	—	3.0	—	-3.8	0.0	—
	**Median sodium per 100 g/mL (mg)**	**Absolute change**	-3.0	—	-6.0	—	0.0	2.0	—	—	—	—	0.0	-2.0	—	—	—	-6.0	—	0.0	—	-1.0	0.0	—
		**% change**	-33.3	—	-37.5	—	N/A	100.0	—	—	—	—	0.0	-33.3	—	—	—	-42.9	—	N/A	—	-15.0	0.0	—
	**Median saturated fat per 100 g/mL (g)**	**Absolute change**	0.0	—	0.0	—	0.0	0.0	—	—	—	—	0.0	0.0	—	—	—	0.0	—	0.0	—	0.0	0.0	—
		**% change**	N/A	—	N/A	—	N/A	N/A	—	—	—	—	N/A	N/A	—	—	—	N/A	—	N/A	—	N/A	N/A	—
	**Median trans fat per 100 g/mL (g)**	**Absolute change**	0.0	—	0.0	—	0.0	0.0	—	—	—	—	0.0	0.0	—	—	—	0.0	—	0.0	—	0.0	0.0	—
		**% change**	N/A	—	N/A	—	N/A	N/A	—	—	—	—	N/A	N/A	—	—	—	N/A	—	N/A	—	N/A	N/A	—
	**Median total sugars per 100 g/mL (g)**	**Absolute change**	0.0	—	-0.2	—	0.0	0.0	—	—	—	—	0.0	-0.4	—	—	—	-2.0	—	0.0	—	-1.1	-0.4	—
		**% change**	0.0	—	-1.9	—	0.0	0.0	—	—	—	—	0.0	-3.8	—	—	—	-15.6	—	0.0	—	-9.5	-3.3	—
	**Median free sugars per 100 g/mL (g)**	**Absolute change**	0.0	—	-0.2	—	0.0	0.0	—	—	—	—	0.0	-0.8	—	—	—	-1.4	—	0.0	—	-1.2	-0.4	—
		**% change**	0.0	—	-1.9	—	N/A	0.0	—	—	—	—	0.0	-8.7	—	—	—	-11.5	—	0.0	—	-12.0	-3.3	—
Fruit juices, nectars and fruit drinks (J11)	Number of products	2013	52	0	20	0	8	55	0	0	0	0	8	82	0	0	0	25	0	23	5	68	25	0
		2017	71	0	11	0	8	65	0	0	0	0	9	102	0	0	0	27	0	24	0	56	18	0
	Mean Health Star Rating	Absolute change	0.0	—	0.2	—	0.6	0.0	—	—	—	—	0.0	0.5	—	—	—	0.5	—	-0.7	—	-0.1	-0.2	—
		% change	0.5	—	10.0	—	34.5	1.4	—	—	—	—	1.0	20.4	—	—	—	18.5	—	-16.0	—	-4.1	-6.3	—
	Median calories per 100 g/mL (kcal)	Absolute change	0.0	—	0.0	—	-28.0	0.0	—	—	—	—	0.0	0.0	—	—	—	-4.0	—	1.3	—	-3.0	0.0	—
		% change	0.0	—	0.0	—	-43.8	0.0	—	—	—	—	0.0	0.0	—	—	—	-8.3	—	3.0	—	-6.3	0.0	—
	Median sodium per 100 g/mL (mg)	Absolute change	-3.0	—	-6.0	—	13.0	2.0	—	—	—	—	0.0	-4.0	—	—	—	-8.0	—	0.0	—	6.5	0.0	—
		% change	-33.3	—	-37.5	—	76.5	100.0	—	—	—	—	0.0	-50.0	—	—	—	-50.0	—	N/A	—	86.7	0.0	—
	Median saturated fat per 100 g/mL (g)	Absolute change	0.0	—	0.0	—	0.0	0.0	—	—	—	—	0.0	0.0	—	—	—	0.0	—	0.0	—	0.0	0.0	—
		% change	N/A	—	N/A	—	N/A	N/A	—	—	—	—	N/A	N/A	—	—	—	N/A	—	N/A	—	N/A	N/A	—
	Median trans fat per 100 g/mL (g)	Absolute change	0.0	—	0.0	—	0.0	0.0	—	—	—	—	0.0	0.0	—	—	—	0.0	—	0.0	—	0.0	0.0	—
		% change	N/A	—	N/A	—	N/A	N/A	—	—	—	—	N/A	N/A	—	—	—	N/A	—	N/A	—	N/A	N/A	—
	Median total sugars per 100 g/mL (g)	Absolute change	0.0	—	-0.2	—	-6.2	0.0	—	—	—	—	0.0	0.0	—	—	—	-0.8	—	0.0	—	-0.9	-0.4	—
		% change	0.0	—	-1.9	—	-43.4	0.0	—	—	—	—	0.0	0.0	—	—	—	-6.9	—	0.0	—	-8.1	-3.3	—
	Median free sugars per 100 g/mL (g)	Absolute change	0.0	—	-0.2	—	-6.2	0.0	—	—	—	—	0.0	0.0	—	—	—	-0.8	—	0.0	—	-0.9	-0.4	—
		% change	0.0	—	-1.9	—	-43.4	0.0	—	—	—	—	0.0	0.0	—	—	—	-6.9	—	0.0	—	-8.1	-3.3	—
Fruit, frozen or canned, coated or uncoated (J1-J5)	Number of products	2013	0	0	0	0	0	0	0	0	0	0	0	36	0	0	0	0	0	0	0	24	0	0
		2017	0	0	0	0	0	0	0	0	0	0	0	54	0	0	0	0	0	0	0	42	0	0
	Mean Health Star Rating	Absolute change	—	—	—	—	—	—	—	—	—	—	—	0.2	—	—	—	—	—	—	—	0.2	—	—
		% change	—	—	—	—	—	—	—	—	—	—	—	4.3	—	—	—	—	—	—	—	5.7	—	—
	Median calories per 100 g/mL (kcal)	Absolute change	—	—	—	—	—	—	—	—	—	—	—	-7.1	—	—	—	—	—	—	—	-4.2	—	—
		% change	—	—	—	—	—	—	—	—	—	—	—	-12.5	—	—	—	—	—	—	—	-6.9	—	—

			A. Lassonde	Agropur	Campbell Soup	Canada Bread	Canada Dry Mott's	Coca-Cola	Danone	General Mills	George Weston	Kellogg	Kraft Heinz	Loblaw	Maple Leaf Foods	Mondelēz	Nestlé	Ocean Spray	Parmalat	PepsiCo	Saputo	Sobeys	Sun-Rype	Unilever
	Median sodium per 100 g/mL (mg)	Absolute change	—	—	—	—	—	—	—	—	—	—	—	0.0	—	—	—	—	—	—	—	-6.7	—	—
		% change	—	—	—	—	—	—	—	—	—	—	—	N/A	—	—	—	—	—	—	—	-100.0	—	—
	Median saturated fat per 100 g/mL (g)	Absolute change	—	—	—	—	—	—	—	—	—	—	—	0.0	—	—	—	—	—	—	—	0.0	—	—
		% change	—	—	—	—	—	—	—	—	—	—	—	N/A	—	—	—	—	—	—	—	N/A	—	—
	Median trans fat per 100 g/mL (g)	Absolute change	—	—	—	—	—	—	—	—	—	—	—	0.0	—	—	—	—	—	—	—	0.0	—	—
		% change	—	—	—	—	—	—	—	—	—	—	—	N/A	—	—	—	—	—	—	—	N/A	—	—
	Median total sugars per 100 g/mL (g)	Absolute change	—	—	—	—	—	—	—	—	—	—	—	-0.5	—	—	—	—	—	—	—	-3.8	—	—
		% change	—	—	—	—	—	—	—	—	—	—	—	-5.2	—	—	—	—	—	—	—	-29.9	—	—
	Median free sugars per 100 g/mL (g)	Absolute change	—	—	—	—	—	—	—	—	—	—	—	0.0	—	—	—	—	—	—	—	-2.7	—	—
		% change	—	—	—	—	—	—	—	—	—	—	—	N/A	—	—	—	—	—	—	—	-45.2	—	—
Other, e.g., apple sauce, dried or candied fruit, fruit relishes (J6, J7, J8, J9, J10, J12)	Number of products	2013	0	0	0	0	17	0	0	0	0	0	0	37	0	0	0	5	0	0	0	28	0	0
		2017	0	0	0	0	17	0	0	0	0	0	0	45	0	0	0	5	0	0	0	28	0	0
	Mean Health Star Rating	Absolute change	—	—	—	—	-0.1	—	—	—	—	—	—	-0.1	—	—	—	0.2	—	—	—	-0.3	—	—
		% change	—	—	—	—	-3.0	—	—	—	—	—	—	-3.5	—	—	—	7.4	—	—	—	-7.3	—	—
	Median calories per 100 g/mL (kcal)	Absolute change	—	—	—	—	0.0	—	—	—	—	—	—	0.0	—	—	—	-25.0	—	—	—	10.0	—	—
		% change	—	—	—	—	0.0	—	—	—	—	—	—	0.0	—	—	—	-7.1	—	—	—	3.4	—	—
	Median sodium per 100 g/mL (mg)	Absolute change	—	—	—	—	0.0	—	—	—	—	—	—	-16.7	—	—	—	0.0	—	—	—	0.0	—	—
		% change	—	—	—	—	N/A	—	—	—	—	—	—	-100.0	—	—	—	N/A	—	—	—	N/A	—	—
	Median saturated fat per 100 g/mL (g)	Absolute change	—	—	—	—	0.0	—	—	—	—	—	—	0.0	—	—	—	0.0	—	—	—	0.0	—	—
		% change	—	—	—	—	N/A	—	—	—	—	—	—	N/A	—	—	—	N/A	—	—	—	N/A	—	—
	Median trans fat per 100 g/mL (g)	Absolute change	—	—	—	—	0.0	—	—	—	—	—	—	0.0	—	—	—	0.0	—	—	—	0.0	—	—
		% change	—	—	—	—	N/A	—	—	—	—	—	—	N/A	—	—	—	N/A	—	—	—	N/A	—	—
	Median total sugars per 100 g/mL (g)	Absolute change	—	—	—	—	0.0	—	—	—	—	—	—	9.2	—	—	—	7.5	—	—	—	10.0	—	—
		% change	—	—	—	—	0.0	—	—	—	—	—	—	24.4	—	—	—	11.5	—	—	—	42.1	—	—
	Median free sugars per 100 g/mL (g)	Absolute change	—	—	—	—	0.0	—	—	—	—	—	—	0.0	—	—	—	16.2	—	—	—	0.0	—	—
		% change	—	—	—	—	N/A	—	—	—	—	—	—	N/A	—	—	—	50.7	—	—	—	N/A	—	—
**Legumes, includes tofu and tempeh (K)**	**Number of products**	**2013**	**0**	**0**	**0**	**0**	**0**	**0**	**0**	**1**	**0**	**0**	**2**	**43**	**0**	**0**	**0**	**0**	**0**	**0**	**0**	**12**	**0**	**0**
		**2017**	**0**	**0**	**0**	**0**	**0**	**0**	**0**	**0**	**0**	**0**	**0**	**59**	**2**	**0**	**0**	**0**	**0**	**0**	**0**	**18**	**0**	**0**
	**Mean Health Star Rating**	**Absolute change**	—	—	—	—	—	—	—	—	—	—	—	**0.0**	—	—	—	—	—	—	—	**0.2**	—	—
		**% change**	—	—	—	—	—	—	—	—	—	—	—	**0.9**	—	—	—	—	—	—	—	**3.6**	—	—
	**Median calories per 100 g/mL (kcal)**	**Absolute change**	—	—	—	—	—	—	—	—	—	—	—	**20.0**	—	—	—	—	—	—	—	**-6.0**	—	—
		**% change**	—	—	—	—	—	—	—	—	—	—	—	**20.0**	—	—	—	—	—	—	—	**-6.7**	—	—
	**Median sodium per 100 g/mL (mg)**	**Absolute change**	—	—	—	—	—	—	—	—	—	—	—	**2.8**	—	—	—	—	—	—	—	**-44.0**	—	—
		**% change**	—	—	—	—	—	—	—	—	—	—	—	**55.2**	—	—	—	—	—	—	—	**-37.3**	—	—
	**Median saturated fat per 100 g/mL (g)**	**Absolute change**	—	—	—	—	—	—	—	—	—	—	—	**0.0**	—	—	—	—	—	—	—	**0.0**	—	—
		**% change**	—	—	—	—	—	—	—	—	—	—	—	**0.0**	—	—	—	—	—	—	—	**-12.5**	—	—
	**Median trans fat per 100 g/mL (g)**	**Absolute change**	—	—	—	—	—	—	—	—	—	—	—	**0.0**	—	—	—	—	—	—	—	**0.0**	—	—
		**% change**	—	—	—	—	—	—	—	—	—	—	—	**N/A**	—	—	—	—	—	—	—	**N/A**	—	—
	**Median total sugars per 100 g/mL (g)**	**Absolute change**	—	—	—	—	—	—	—	—	—	—	—	**0.2**	—	—	—	—	—	—	—	**0.0**	—	—
		**% change**	—	—	—	—	—	—	—	—	—	—	—	**25.0**	—	—	—	—	—	—	—	**0.0**	—	—
	**Median free sugars per 100 g/mL (g)**	**Absolute change**	—	—	—	—	—	—	—	—	—	—	—	**0.0**	—	—	—	—	—	—	—	**0.0**	—	—
		**% change**	—	—	—	—	—	—	—	—	—	—	—	**N/A**	—	—	—	—	—	—	—	**N/A**	—	—
**Meat, poultry, their products and substitutes (L)**	**Number of products**	**2013**	**0**	**0**	**0**	**0**	**0**	**0**	**0**	**0**	**0**	**0**	**0**	**169**	**138**	**0**	**0**	**0**	**0**	**0**	**0**	**81**	**0**	**0**
		**2017**	**0**	**0**	**0**	**0**	**0**	**0**	**0**	**0**	**0**	**0**	**0**	**253**	**126**	**0**	**0**	**0**	**0**	**0**	**0**	**105**	**0**	**0**
	**Mean Health Star Rating**	**Absolute change**	—	—	—	—	—	—	—	—	—	—	—	**0.3**	**0.1**	—	—	—	—	—	—	**0.0**	—	—
		**% change**	—	—	—	—	—	—	—	—	—	—	—	**11.9**	**2.5**	—	—	—	—	—	—	**-1.6**	—	—
	**Median calories per 100 g/mL (kcal)**	**Absolute change**	—	—	—	—	—	—	—	—	—	—	—	**-18.3**	**1.4**	—	—	—	—	—	—	**10.0**	—	—
		**% change**	—	—	—	—	—	—	—	—	—	—	—	**-8.4**	**0.6**	—	—	—	—	—	—	**5.0**	—	—
	**Median sodium per 100 g/mL (mg)**	**Absolute change**	—	—	—	—	—	—	—	—	—	—	—	**-19.0**	**-5.4**	—	—	—	—	—	—	**-63.2**	—	—
		**% change**	—	—	—	—	—	—	—	—	—	—	—	**-3.9**	**-0.6**	—	—	—	—	—	—	**-10.7**	—	—
	**Median saturated fat per 100 g/mL (g)**	**Absolute change**	—	—	—	—	—	—	—	—	—	—	—	**-0.9**	**-1.6**	—	—	—	—	—	—	**0.2**	—	—
		**% change**	—	—	—	—	—	—	—	—	—	—	—	**-20.0**	**-34.7**	—	—	—	—	—	—	**7.1**	—	—
	**Median trans fat per 100 g/mL (g)**	**Absolute change**	—	—	—	—	—	—	—	—	—	—	—	**0.0**	**0.0**	—	—	—	—	—	—	**0.1**	—	—
		**% change**	—	—	—	—	—	—	—	—	—	—	—	**-8.3**	**N/A**	—	—	—	—	—	—	**N/A**	—	—
	**Median total sugars per 100 g/mL (g)**	**Absolute change**	—	—	—	—	—	—	—	—	—	—	—	**0.0**	**0.0**	—	—	—	—	—	—	**0.0**	—	—
		**% change**	—	—	—	—	—	—	—	—	—	—	—	**N/A**	**N/A**	—	—	—	—	—	—	**N/A**	—	—
	**Median free sugars per 100 g/mL (g)**	**Absolute change**	—	—	—	—	—	—	—	—	—	—	—	**0.0**	**0.0**	—	—	—	—	—	—	**0.0**	—	—
		**% change**	—	—	—	—	—	—	—	—	—	—	—	**N/A**	**N/A**	—	—	—	—	—	—	**N/A**	—	—
**Miscellaneous (M)**	**Number of products**	**2013**	**0**	**0**	**0**	**0**	**1**	**0**	**0**	**28**	**0**	**1**	**17**	**66**	**2**	**0**	**3**	**0**	**0**	**17**	**0**	**21**	**0**	**2**
		**2017**	**0**	**0**	**0**	**0**	**2**	**0**	**0**	**44**	**1**	**1**	**11**	**81**	**1**	**7**	**5**	**0**	**0**	**17**	**0**	**44**	**0**	**1**
	**Mean Health Star Rating**	**Absolute change**	—	—	—	—	**0.0**	—	—	**-0.2**	—	**0.5**	**0.4**	**0.0**	**0.0**	—	**-0.1**	—	—	**0.1**	—	**0.0**	—	**0.0**
		**% change**	—	—	—	—	**0.0**	—	—	**-11.9**	—	**25.0**	**40.1**	**2.1**	**0.0**	—	**-16.0**	—	—	**2.4**	—	**-1.1**	—	**0.0**
	**Median calories per 100 g/mL (kcal)**	**Absolute change**	—	—	—	—	**100.0**	—	—	**15.0**	—	**13.2**	**-41.7**	**-2.3**	**0.0**	—	**0.0**	—	—	**-8.3**	—	**3.7**	—	**21.4**
		**% change**	—	—	—	—	**N/A**	—	—	**5.0**	—	**3.7**	**-11.1**	**-0.7**	**0.0**	—	**0.0**	—	—	**-3.3**	—	**1.3**	—	**5.6**
	**Median sodium per 100 g/mL (mg)**	**Absolute change**	—	—	—	—	**-2500.0**	—	—	**-16.0**	—	**-13.2**	**38.1**	**-126.8**	**-125.0**	—	**0.0**	—	—	**3.4**	—	**-245.2**	—	**-1270.4**
		**% change**	—	—	—	—	**-8.3**	—	—	**-4.2**	—	**-2.1**	**1.6**	**-26.9**	**-5.6**	—	**0.0**	—	—	**0.9**	—	**-40.9**	—	**-20.8**
	**Median saturated fat per 100 g/mL (g)**	**Absolute change**	—	—	—	—	**0.0**	—	—	**0.1**	—	**0.0**	**-3.1**	**1.9**	**3.1**	—	**0.0**	—	—	**0.3**	—	**0.5**	—	**1.3**
		**% change**	—	—	—	—	**N/A**	—	—	**5.6**	—	**N/A**	**-100.0**	**448.5**	**33.3**	—	**N/A**	—	—	**35.2**	—	**27.8**	—	**69.0**
	**Median trans fat per 100 g/mL (g)**	**Absolute change**	—	—	—	—	**0.0**	—	—	**0.0**	—	**0.0**	**0.0**	**0.0**	**0.0**	—	**0.0**	—	—	**0.0**	—	**0.0**	—	**0.0**
		**% change**	—	—	—	—	**N/A**	—	—	**N/A**	—	**N/A**	**N/A**	**N/A**	**N/A**	—	**N/A**	—	—	**N/A**	—	**N/A**	—	**N/A**
	**Median total sugars per 100 g/mL (g)**	**Absolute change**	—	—	—	—	**0.0**	—	—	**0.9**	—	**0.4**	**0.0**	**7.8**	**0.0**	—	**2.5**	—	—	**0.9**	—	**-0.9**	—	**13.2**
		**% change**	—	—	—	—	**N/A**	—	—	**4.9**	—	**3.7**	**N/A**	**N/A**	**N/A**	—	**21.4**	—	—	**4.2**	—	**-6.3**	—	**69.0**
	**Median free sugars per 100 g/mL (g)**	**Absolute change**	—	—	—	—	**0.0**	—	—	**1.3**	—	**0.1**	**0.0**	**2.3**	**0.0**	—	**2.7**	—	—	**1.2**	—	**-1.9**	—	**12.4**
		**% change**	—	—	—	—	**N/A**	—	—	**7.4**	—	**1.2**	**N/A**	**N/A**	**N/A**	—	**25.9**	—	—	**6.0**	—	**-13.0**	—	**65.0**
Baking/cooking	Number of products	2013	0	0	0	0	0	0	0	20	0	1	17	42	2	0	0	0	0	17	0	18	0	0
		2017	0	0	0	0	0	0	0	30	1	1	11	62	1	7	0	0	0	17	0	34	0	0

			A. Lassonde	Agropur	Campbell Soup	Canada Bread	Canada Dry Mott's	Coca-Cola	Danone	General Mills	George Weston	Kellogg	Kraft Heinz	Loblaw	Maple Leaf Foods	Mondelēz	Nestlé	Ocean Spray	Parmalat	PepsiCo	Saputo	Sobeys	Sun-Rype	Unilever
ingredients, e.g., baking	Mean Health Star	Absolute change	—	—	—	—	—	—	—	-0.2	—	0.5	0.4	-0.1	0.0	—	—	—	—	0.1	—	0.0	—	—
	Rating	% change	—	—	—	—	—	—	—	-7.5	—	25.0	40.1	-6.5	0.0	—	—	—	—	2.4	—	2.3	—	—
powder, yeast,	Median calories per	Absolute change	—	—	—	—	—	—	—	13.2	—	13.2	-41.7	-12.0	0.0	—	—	—	—	-8.3	—	4.6	—	—
	100 g/mL (kcal)	% change	—	—	—	—	—	—	—	4.5	—	3.7	-11.1	-3.3	0.0	—	—	—	—	-3.3	—	1.6	—	—
sprinkles, bread	Median sodium per	Absolute change	—	—	—	—	—	—	—	-5.7	—	-13.2	38.1	-50.5	-125.0	—	—	—	—	3.4	—	-224.7	—	—
	100 g/mL (mg)	% change	—	—	—	—	—	—	—	-1.6	—	-2.1	1.6	-13.3	-5.6	—	—	—	—	0.9	—	-40.5	—	—
crumbs, cocoa	Median saturated fat	Absolute change	—	—	—	—	—	—	—	0.5	—	0.0	-3.1	1.4	3.1	—	—	—	—	0.3	—	0.8	—	—
	per 100 g/mL (g)	% change	—	—	—	—	—	—	—	25.0	—	N/A	-100.0	58.0	33.3	—	—	—	—	35.2	—	36.0	—	—
powder, etc. (M1, M3,	Median trans fat per	Absolute change	—	—	—	—	—	—	—	0.0	—	0.0	0.0	0.0	0.0	—	—	—	—	0.0	—	0.0	—	—
	100 g/mL (g)	% change	—	—	—	—	—	—	—	N/A	—	N/A	N/A	N/A	N/A	—	—	—	—	N/A	—	N/A	—	—
M5, M8, M11, M12)	Median total sugars	Absolute change	—	—	—	—	—	—	—	12.8	—	0.4	0.0	11.2	0.0	—	—	—	—	0.9	—	3.2	—	—
	per 100 g/mL (g)	% change	—	—	—	—	—	—	—	63.8	—	3.7	N/A	134.3	N/A	—	—	—	—	4.2	—	21.7	—	—
	Median free sugars	Absolute change	—	—	—	—	—	—	—	13.4	—	0.1	0.0	12.5	0.0	—	—	—	—	1.2	—	2.7	—	—
	per 100 g/mL (g)	% change	—	—	—	—	—	—	—	70.1	—	1.2	N/A	208.2	N/A	—	—	—	—	6.0	—	18.9	—	—
Seasoning salts and	Number of products	2013	0	0	0	0	1	0	0	8	0	0	0	24	0	0	3	0	0	0	0	3	0	2
		2017	0	0	0	0	2	0	0	14	0	0	0	19	0	0	5	0	0	0	0	10	0	1
mixes (M9, M10)	Mean Health Star	Absolute change	—	—	—	—	0.0	—	—	-0.2	—	—	—	0.5	—	—	-0.1	—	—	—	—	0.0	—	0.0
	Rating	% change	—	—	—	—	0.0	—	—	-22.1	—	—	—	24.9	—	—	-16.0	—	—	—	—	1.3	—	0.0
	Median calories per	Absolute change	—	—	—	—	100.0	—	—	0.0	—	—	—	250.0	—	—	0.0	—	—	—	—	-164.8	—	21.4
	100 g/mL (kcal)	% change	—	—	—	—	N/A	—	—	0.0	—	—	—	N/A	—	—	0.0	—	—	—	—	-53.6	—	5.6
	Median sodium per	Absolute change	—	—	—	—	-2500.0	—	—	333.3	—	—	—	-437.5	—	—	0.0	—	—	—	—	445.1	—	-1270.4
	100 g/mL (mg)	% change	—	—	—	—	-8.3	—	—	6.1	—	—	—	-8.0	—	—	0.0	—	—	—	—	8.0	—	-20.8
	Median saturated fat	Absolute change	—	—	—	—	0.0	—	—	0.0	—	—	—	0.0	—	—	0.0	—	—	—	—	0.0	—	1.3
	per 100 g/mL (g)	% change	—	—	—	—	N/A	—	—	N/A	—	—	—	N/A	—	—	N/A	—	—	—	—	N/A	—	69.0
	Median trans fat per	Absolute change	—	—	—	—	0.0	—	—	0.0	—	—	—	0.0	—	—	0.0	—	—	—	—	0.0	—	0.0
	100 g/mL (g)	% change	—	—	—	—	N/A	—	—	N/A	—	—	—	N/A	—	—	N/A	—	—	—	—	N/A	—	N/A
	Median total sugars	Absolute change	—	—	—	—	0.0	—	—	0.0	—	—	—	0.0	—	—	2.5	—	—	—	—	-14.3	—	13.2
	per 100 g/mL (g)	% change	—	—	—	—	N/A	—	—	N/A	—	—	—	N/A	—	—	21.4	—	—	—	—	-100.0	—	69.0
	Median free sugars	Absolute change	—	—	—	—	0.0	—	—	0.0	—	—	—	0.0	—	—	2.7	—	—	—	—	-14.3	—	12.4
	per 100 g/mL (g)	% change	—	—	—	—	N/A	—	—	N/A	—	—	—	N/A	—	—	25.9	—	—	—	—	-100.0	—	65.0
**Combinatio**	**Number of products**	**2013**	**0**	**0**	**1**	**3**	**0**	**0**	**0**	**38**	**0**	**3**	**66**	**202**	**26**	**0**	**83**	**0**	**0**	**0**	**0**	**91**	**0**	**21**
**n dishes (N)**		**2017**	**0**	**0**	**6**	**2**	**0**	**0**	**0**	**45**	**0**	**0**	**47**	**243**	**20**	**0**	**84**	**0**	**0**	**0**	**0**	**150**	**0**	**22**
	**Mean Health Star**	**Absolute change**	—	—	**0.1**	**-1.5**	—	—	—	**0.0**	—	—	**0.0**	**0.1**	**-0.6**	—	**0.1**	—	—	—	—	**-0.1**	—	**0.0**
	**Rating**	**% change**	—	—	**2.1**	**-42.9**	—	—	—	**0.5**	—	—	**-1.0**	**3.5**	**-20.6**	—	**1.7**	—	—	—	—	**-4.1**	—	**1.5**
	**Median calories per**	**Absolute change**	—	—	**6.2**	**24.5**	—	—	—	**12.4**	—	—	**8.2**	**-1.4**	**45.7**	—	**8.5**	—	—	—	—	**25.5**	—	**1.0**
	**100 g/mL (kcal)**	**% change**	—	—	**6.4**	**11.0**	—	—	—	**7.2**	—	—	**7.0**	**-0.8**	**18.0**	—	**4.4**	—	—	—	—	**16.1**	—	**0.9**
	**Median sodium per**	**Absolute change**	—	—	**2.0**	**660.2**	—	—	—	**-68.3**	—	—	**5.0**	**4.4**	**50.8**	—	**64.6**	—	—	—	—	**28.6**	—	**-4.7**
	**100 g/mL (mg)**	**% change**	—	—	**0.8**	**198.1**	—	—	—	**-14.5**	—	—	**1.8**	**1.4**	**11.1**	—	**17.8**	—	—	—	—	**8.8**	—	**-1.9**
	**Median saturated**	**Absolute change**	—	—	**0.0**	**-1.4**	—	—	—	**0.4**	—	—	**-0.4**	**0.1**	**3.2**	—	**-0.1**	—	—	—	—	**0.3**	—	**0.0**
	**fat per 100 g/mL (g)**	**% change**	—	—	**0.0**	**-41.6**	—	—	—	**25.1**	—	—	**-46.3**	**4.4**	**91.2**	—	**-3.0**	—	—	—	—	**25.1**	—	**-0.9**
	**Median trans fat**	**Absolute change**	—	—	**-0.1**	**0.0**	—	—	—	**-0.1**	—	—	**0.0**	**0.0**	**0.1**	—	**0.0**	—	—	—	—	**0.0**	—	**0.0**
	**per 100 g/mL (g)**	**% change**	—	—	**-50.8**	**16.9**	—	—	—	**-100.0**	—	—	**N/A**	**-36.9**	**53.3**	—	**-12.0**	—	—	—	—	**-11.5**	—	**N/A**
	**Median total sugars**	**Absolute change**	—	—	**0.0**	**-2.5**	—	—	—	**0.3**	—	—	**0.3**	**0.1**	**-1.4**	—	**-0.5**	—	—	—	—	**0.0**	—	**0.0**
	**per 100 g/mL (g)**	**% change**	—	—	**0.0**	**-56.2**	—	—	—	**14.4**	—	—	**7.8**	**4.9**	**-19.8**	—	**-17.6**	—	—	—	—	**0.2**	—	**-0.4**
	**Median free sugars**	**Absolute change**	—	—	**-0.4**	**0.4**	—	—	—	**-0.1**	—	—	**-0.3**	**-0.3**	**-0.4**	—	**0.0**	—	—	—	—	**-0.2**	—	**-0.4**
	**per 100 g/mL (g)**	**% change**	—	—	**-46.7**	**N/A**	—	—	—	**-12.4**	—	—	**-23.1**	**-35.0**	**-7.2**	—	**1.2**	—	—	—	—	**-13.0**	—	**-33.5**
**Nuts and**	**Number of products**	**2013**	**0**	**0**	**0**	**0**	**0**	**0**	**0**	**0**	**0**	**0**	**11**	**29**	**0**	**0**	**0**	**0**	**0**	**0**	**0**	**12**	**0**	**5**
**seeds (O)**		**2017**	**0**	**0**	**0**	**0**	**0**	**0**	**0**	**0**	**0**	**0**	**11**	**44**	**0**	**0**	**0**	**0**	**0**	**0**	**0**	**28**	**0**	**0**
	**Mean Health Star**	**Absolute change**	—	—	—	—	—	—	—	—	—	—	**0.2**	**0.1**	—	—	—	—	—	—	—	**0.3**	—	—
	**Rating**	**% change**	—	—	—	—	—	—	—	—	—	—	**6.2**	**2.6**	—	—	—	—	—	—	—	**7.8**	—	—
	**Median calories per**	**Absolute change**	—	—	—	—	—	—	—	—	—	—	**61.5**	**-33.3**	—	—	—	—	—	—	—	**0.0**	—	—
	**100 g/mL (kcal)**	**% change**	—	—	—	—	—	—	—	—	—	—	**11.4**	**-5.0**	—	—	—	—	—	—	—	**0.0**	—	—
	**Median sodium per**	**Absolute change**	—	—	—	—	—	—	—	—	—	—	**33.3**	**0.0**	—	—	—	—	—	—	—	**0.0**	—	—
	**100 g/mL (mg)**	**% change**	—	—	—	—	—	—	—	—	—	—	**9.1**	**N/A**	—	—	—	—	—	—	—	**N/A**	—	—
	**Median saturated**	**Absolute change**	—	—	—	—	—	—	—	—	—	—	**0.0**	**-0.3**	—	—	—	—	—	—	—	**0.2**	—	—
	**fat per 100 g/mL (g)**	**% change**	—	—	—	—	—	—	—	—	—	—	**0.0**	**-4.0**	—	—	—	—	—	—	—	**2.4**	—	—
	**Median trans fat**	**Absolute change**	—	—	—	—	—	—	—	—	—	—	**0.0**	**0.0**	—	—	—	—	—	—	—	**0.0**	—	—
	**per 100 g/mL (g)**	**% change**	—	—	—	—	—	—	—	—	—	—	**N/A**	**N/A**	—	—	—	—	—	—	—	**N/A**	—	—
	**Median total sugars**	**Absolute change**	—	—	—	—	—	—	—	—	—	—	**0.0**	**0.7**	—	—	—	—	—	—	—	**-1.3**	—	—
	**per 100 g/mL (g)**	**% change**	—	—	—	—	—	—	—	—	—	—	**0.0**	**20.0**	—	—	—	—	—	—	—	**-25.0**	—	—
	**Median free sugars**	**Absolute change**	—	—	—	—	—	—	—	—	—	—	**0.0**	**0.0**	—	—	—	—	—	—	—	**0.0**	—	—
	**per 100 g/mL (g)**	**% change**	—	—	—	—	—	—	—	—	—	—	**-2.0**	**N/A**	—	—	—	—	—	—	—	**N/A**	—	—
Nut butters,	Number of products	2013	0	0	0	0	0	0	0	0	0	0	11	9	0	0	0	0	0	0	0	6	0	5
pastes and		2017	0	0	0	0	0	0	0	0	0	0	11	12	0	0	0	0	0	0	0	8	0	0
creams (O2,	Mean Health Star	Absolute change	—	—	—	—	—	—	—	—	—	—	0.2	0.1	—	—	—	—	—	—	—	0.5	—	—
O3)	Rating	% change	—	—	—	—	—	—	—	—	—	—	6.2	3.3	—	—	—	—	—	—	—	13.4	—	—
	Median calories per	Absolute change	—	—	—	—	—	—	—	—	—	—	61.5	-66.7	—	—	—	—	—	—	—	0.0	—	—
	100 g/mL (kcal)	% change	—	—	—	—	—	—	—	—	—	—	11.4	-10.0	—	—	—	—	—	—	—	0.0	—	—
	Median sodium per	Absolute change	—	—	—	—	—	—	—	—	—	—	33.3	-17.7	—	—	—	—	—	—	—	-100.0	—	—
	100 g/mL (mg)	% change	—	—	—	—	—	—	—	—	—	—	9.1	-82.5	—	—	—	—	—	—	—	-75.0	—	—
	Median saturated fat	Absolute change	—	—	—	—	—	—	—	—	—	—	0.0	0.0	—	—	—	—	—	—	—	0.0	—	—
	per 100 g/mL (g)	% change	—	—	—	—	—	—	—	—	—	—	0.0	0.0	—	—	—	—	—	—	—	0.0	—	—
	Median trans fat per	Absolute change	—	—	—	—	—	—	—	—	—	—	0.0	0.0	—	—	—	—	—	—	—	0.0	—	—
	100 g/mL (g)	% change	—	—	—	—	—	—	—	—	—	—	N/A	N/A	—	—	—	—	—	—	—	N/A	—	—
	Median total sugars	Absolute change	—	—	—	—	—	—	—	—	—	—	0.0	-0.5	—	—	—	—	—	—	—	0.0	—	—
	per 100 g/mL (g)	% change	—	—	—	—	—	—	—	—	—	—	0.0	-6.7	—	—	—	—	—	—	—	0.0	—	—

			A. Lassonde	Agropur	Campbell Soup	Canada Bread	Canada Dry Mott's	Coca-Cola	Danone	General Mills	George Weston	Kellogg	Kraft Heinz	Loblaw	Maple Leaf Foods	Mondelēz	Nestlé	Ocean Spray	Parmalat	PepsiCo	Saputo	Sobeys	Sun-Rype	Unilever
	Median free sugars	Absolute change	—	—	—	—	—	—	—	—	—	—	0.0	0.0	—	—	—	—	—	—	—	-1.0	—	—
	per 100 g/mL (g)	% change	—	—	—	—	—	—	—	—	—	—	-2.0	N/A	—	—	—	—	—	—	—	-100.0	—	—
Nuts, seeds	Number of products	2013	0	0	0	0	0	0	0	0	0	0	0	20	0	0	0	0	0	0	0	6	0	0
and flours		2017	0	0	0	0	0	0	0	0	0	0	0	32	0	0	0	0	0	0	0	20	0	0
(O1, O4)	Mean Health Star	Absolute change	—	—	—	—	—	—	—	—	—	—	—	0.1	—	—	—	—	—	—	—	-0.2	—	—
	Rating	% change	—	—	—	—	—	—	—	—	—	—	—	1.4	—	—	—	—	—	—	—	-3.3	—	—
	Median calories per	Absolute change	—	—	—	—	—	—	—	—	—	—	—	-3.3	—	—	—	—	—	—	—	60.0	—	—
	100 g/mL (kcal)	% change	—	—	—	—	—	—	—	—	—	—	—	-0.5	—	—	—	—	—	—	—	9.1	—	—
	Median sodium per	Absolute change	—	—	—	—	—	—	—	—	—	—	—	0.0	—	—	—	—	—	—	—	0.0	—	—
	100 g/mL (mg)	% change	—	—	—	—	—	—	—	—	—	—	—	N/A	—	—	—	—	—	—	—	N/A	—	—
	Median saturated fat	Absolute change	—	—	—	—	—	—	—	—	—	—	—	0.1	—	—	—	—	—	—	—	-0.5	—	—
	per 100 g/mL (g)	% change	—	—	—	—	—	—	—	—	—	—	—	1.4	—	—	—	—	—	—	—	-6.7	—	—
	Median trans fat per	Absolute change	—	—	—	—	—	—	—	—	—	—	—	0.0	—	—	—	—	—	—	—	0.0	—	—
	100 g/mL (g)	% change	—	—	—	—	—	—	—	—	—	—	—	N/A	—	—	—	—	—	—	—	N/A	—	—
	Median total sugars	Absolute change	—	—	—	—	—	—	—	—	—	—	—	0.0	—	—	—	—	—	—	—	-2.0	—	—
	per 100 g/mL (g)	% change	—	—	—	—	—	—	—	—	—	—	—	0.0	—	—	—	—	—	—	—	-50.0	—	—
	Median free sugars	Absolute change	—	—	—	—	—	—	—	—	—	—	—	0.0	—	—	—	—	—	—	—	0.0	—	—
	per 100 g/mL (g)	% change	—	—	—	—	—	—	—	—	—	—	—	N/A	—	—	—	—	—	—	—	N/A	—	—
Potatoes (P)	Number of products	2013	0	0	0	0	0	0	0	8	0	0	0	12	0	0	0	0	0	0	0	16	0	4
		2017	0	0	0	0	0	0	0	7	0	0	0	23	0	0	0	0	0	0	0	23	0	0
	Mean Health Star	Absolute change	—	—	—	—	—	—	—	0.1	—	—	—	0.1	—	—	—	—	—	—	—	0.4	—	—
	Rating	% change	—	—	—	—	—	—	—	2.6	—	—	—	2.0	—	—	—	—	—	—	—	12.7	—	—
	Median calories per	Absolute change	—	—	—	—	—	—	—	0.0	—	—	—	-14.3	—	—	—	—	—	—	—	-17.6	—	—
	100 g/mL (kcal)	% change	—	—	—	—	—	—	—	0.0	—	—	—	-9.7	—	—	—	—	—	—	—	-12.0	—	—
	Median sodium per	Absolute change	—	—	—	—	—	—	—	-25.8	—	—	—	11.8	—	—	—	—	—	—	—	-99.5	—	—
	100 g/mL (mg)	% change	—	—	—	—	—	—	—	-1.6	—	—	—	6.3	—	—	—	—	—	—	—	-56.4	—	—
	Median saturated	Absolute change	—	—	—	—	—	—	—	0.0	—	—	—	0.1	—	—	—	—	—	—	—	-0.4	—	—
	fat per 100 g/mL (g)	% change	—	—	—	—	—	—	—	N/A	—	—	—	20.0	—	—	—	—	—	—	—	-63.2	—	—
	Median trans fat	Absolute change	—	—	—	—	—	—	—	0.0	—	—	—	0.0	—	—	—	—	—	—	—	0.0	—	—
	per 100 g/mL (g)	% change	—	—	—	—	—	—	—	N/A	—	—	—	N/A	—	—	—	—	—	—	—	N/A	—	—
	Median total sugars	Absolute change	—	—	—	—	—	—	—	0.0	—	—	—	0.0	—	—	—	—	—	—	—	-0.4	—	—
	per 100 g/mL (g)	% change	—	—	—	—	—	—	—	0.0	—	—	—	N/A	—	—	—	—	—	—	—	-100.0	—	—
	Median free sugars	Absolute change	—	—	—	—	—	—	—	0.0	—	—	—	0.0	—	—	—	—	—	—	—	0.0	—	—
	per 100 g/mL (g)	% change	—	—	—	—	—	—	—	N/A	—	—	—	N/A	—	—	—	—	—	—	—	N/A	—	—
Salads (Q)	Number of products	2013	0	0	0	0	0	0	0	0	0	0	1	8	0	0	0	0	0	0	0	16	0	0
		2017	0	0	0	0	0	0	0	0	0	0	0	32	0	0	0	0	0	0	0	29	0	0
	Mean Health Star	Absolute change	—	—	—	—	—	—	—	—	—	—	—	0.2	—	—	—	—	—	—	—	0.4	—	—
	Rating	% change	—	—	—	—	—	—	—	—	—	—	—	4.5	—	—	—	—	—	—	—	9.4	—	—
	Median calories per	Absolute change	—	—	—	—	—	—	—	—	—	—	—	-14.0	—	—	—	—	—	—	—	-55.0	—	—
	100 g/mL (kcal)	% change	—	—	—	—	—	—	—	—	—	—	—	-9.8	—	—	—	—	—	—	—	-37.9	—	—
	Median sodium per	Absolute change	—	—	—	—	—	—	—	—	—	—	—	-8.3	—	—	—	—	—	—	—	-129.1	—	—
	100 g/mL (mg)	% change	—	—	—	—	—	—	—	—	—	—	—	-3.2	—	—	—	—	—	—	—	-50.2	—	—
	Median saturated	Absolute change	—	—	—	—	—	—	—	—	—	—	—	0.8	—	—	—	—	—	—	—	0.5	—	—
	fat per 100 g/mL (g)	% change	—	—	—	—	—	—	—	—	—	—	—	61.1	—	—	—	—	—	—	—	50.0	—	—
	Median trans fat	Absolute change	—	—	—	—	—	—	—	—	—	—	—	0.1	—	—	—	—	—	—	—	0.0	—	—
	per 100 g/mL (g)	% change	—	—	—	—	—	—	—	—	—	—	—	N/A	—	—	—	—	—	—	—	N/A	—	—
	Median total sugars	Absolute change	—	—	—	—	—	—	—	—	—	—	—	-2.2	—	—	—	—	—	—	—	-3.9	—	—
	per 100 g/mL (g)	% change	—	—	—	—	—	—	—	—	—	—	—	-47.0	—	—	—	—	—	—	—	-65.9	—	—
	Median free sugars	Absolute change	—	—	—	—	—	—	—	—	—	—	—	-3.9	—	—	—	—	—	—	—	-3.8	—	—
	per 100 g/mL (g)	% change	—	—	—	—	—	—	—	—	—	—	—	-100.0	—	—	—	—	—	—	—	-78.4	—	—
Sauces,	Number of products	2013	0	0	15	6	0	0	0	10	0	0	66	221	1	0	3	2	1	13	5	82	0	25
dips,		2017	0	0	21	0	0	0	0	7	0	0	80	258	1	0	3	2	0	19	4	83	0	19
gravies and	Mean Health Star	Absolute change	—	—	0.3	—	—	—	—	0.3	—	—	0.4	0.1	0.0	—	-1.0	0.0	—	0.2	0.0	0.1	—	-0.2
condiments	Rating	% change	—	—	9.1	—	—	—	—	11.4	—	—	20.6	3.1	0.0	—	-40.0	0.0	—	5.9	0.0	2.7	—	-6.7
(R)	Median calories per	Absolute change	—	—	0.0	—	—	—	—	-10.0	—	—	-40.6	16.7	0.0	—	233.3	0.0	—	0.0	-32.3	-13.3	—	16.7
	100 g/mL (kcal)	% change	—	—	0.0	—	—	—	—	-20.0	—	—	-28.9	14.3	0.0	—	350.0	0.0	—	0.0	-16.7	-10.0	—	20.0
	Median sodium per	Absolute change	—	—	-91.7	—	—	—	—	-160.0	—	—	-91.7	-50.0	0.0	—	-7466.7	0.0	—	-125.0	0.0	-50.0	—	-83.3
	100 g/mL (mg)	% change	—	—	-19.6	—	—	—	—	-18.2	—	—	-11.5	-10.0	0.0	—	-81.2	0.0	—	-20.0	0.0	-8.8	—	-14.7
	Median saturated	Absolute change	—	—	0.0	—	—	—	—	0.0	—	—	0.1	0.0	0.0	—	0.0	0.0	—	0.0	-0.8	0.0	—	-0.3
	fat per 100 g/mL (g)	% change	—	—	N/A	—	—	—	—	N/A	—	—	N/A	N/A	N/A	—	N/A	N/A	—	0.0	-8.3	N/A	—	-100.0
	Median trans fat	Absolute change	—	—	0.0	—	—	—	—	0.0	—	—	0.0	0.0	0.0	—	0.0	0.0	—	0.0	0.0	0.0	—	0.0
	per 100 g/mL (g)	% change	—	—	N/A	—	—	—	—	N/A	—	—	N/A	N/A	N/A	—	N/A	N/A	—	N/A	N/A	N/A	—	N/A
	Median total sugars	Absolute change	—	—	0.1	—	—	—	—	-2.0	—	—	-6.9	-1.4	0.0	—	33.3	0.0	—	0.0	3.2	0.0	—	-1.7
	per 100 g/mL (g)	% change	—	—	5.0	—	—	—	—	-33.3	—	—	-51.6	-21.9	0.0	—	500.0	0.0	—	N/A	100.0	0.0	—	-50.0
	Median free sugars	Absolute change	—	—	0.0	—	—	—	—	-1.4	—	—	-10.4	-2.9	0.0	—	32.5	0.0	—	0.0	-0.6	0.0	—	0.3
	per 100 g/mL (g)	% change	—	—	N/A	—	—	—	—	-62.1	—	—	-77.7	-86.6	0.0	—	487.9	0.0	—	N/A	-100.0	-1.3	—	33.6
Snacks (S)	Number of products	2013	0	0	0	0	0	0	0	3	0	9	6	103	8	1	0	0	0	92	0	75	0	0
		2017	0	0	0	3	0	0	0	7	0	17	0	145	8	6	1	1	0	109	0	79	0	0
	Mean Health Star	Absolute change	—	—	—	—	—	—	—	-0.8	—	-0.2	—	0.0	0.1	-0.1	—	—	—	0.0	—	0.0	—	—
	Rating	% change	—	—	—	—	—	—	—	-29.4	—	-10.3	—	1.4	13.3	-4.2	—	—	—	-0.1	—	0.8	—	—
	Median calories per	Absolute change	—	—	—	—	—	—	—	0.0	—	0.0	—	0.0	0.0	-2.6	—	—	—	0.0	—	-20.0	—	—
	100 g/mL (kcal)	% change	—	—	—	—	—	—	—	0.0	—	0.0	—	0.0	0.0	-0.6	—	—	—	0.0	—	-3.7	—	—
	Median sodium per	Absolute change	—	—	—	—	—	—	—	316.2	—	35.7	—	-40.0	-30.0	157.4	—	—	—	-20.0	—	0.0	—	—
	100 g/mL (mg)	% change	—	—	—	—	—	—	—	113.3	—	5.9	—	-8.0	-2.5	14.8	—	—	—	-2.9	—	0.0	—	—
	Median saturated	Absolute change	—	—	—	—	—	—	—	2.4	—	1.8	—	1.4	1.0	-1.0	—	—	—	0.0	—	0.0	—	—
	fat per 100 g/mL (g)	% change	—	—	—	—	—	—	—	104.8	—	25.0	—	48.1	11.8	-34.8	—	—	—	0.0	—	0.0	—	—

			A. Lassonde	Agropur	Campbell Soup	Canada Bread	Canada Dry Mott's	Coca-Cola	Danone	General Mills	George Weston	Kellogg	Kraft Heinz	Loblaw	Maple Leaf Foods	Mondelēz	Nestlé	Ocean Spray	Parmalat	PepsiCo	Saputo	Sobeys	Sun-Rype	Unilever
	Median trans fat	Absolute change	—	—	—	—	—	—	—	0.0	—	0.0	—	0.0	0.0	0.0	—	—	—	0.0	—	0.0	—	—
	per 100 g/mL (g)	% change	—	—	—	—	—	—	—	N/A	—	N/A	—	N/A	0.0	N/A	—	—	—	N/A	—	N/A	—	—
	Median total sugars	Absolute change	—	—	—	—	—	—	—	0.0	—	0.0	—	0.0	1.0	-1.7	—	—	—	0.0	—	0.0	—	—
	per 100 g/mL (g)	% change	—	—	—	—	—	—	—	0.0	—	0.0	—	0.0	N/A	-27.5	—	—	—	0.0	—	0.0	—	—
	Median free sugars	Absolute change	—	—	—	—	—	—	—	-0.5	—	0.5	—	-1.0	1.0	-3.4	—	—	—	0.4	—	0.0	—	—
	per 100 g/mL (g)	% change	—	—	—	—	—	—	—	-14.0	—	19.6	—	-100.0	N/A	-69.9	—	—	—	40.8	—	N/A	—	—
Soups (T)	Number of products	2013	0	0	127	0	0	0	0	0	0	0	1	54	0	0	0	0	0	0	0	32	0	45
		2017	0	0	135	0	0	0	0	0	0	0	0	66	0	0	0	0	0	0	0	41	0	51
	Mean Health Star	Absolute change	—	—	-0.1	—	—	—	—	—	—	—	—	0.0	—	—	—	—	—	—	—	-0.1	—	0.0
	Rating	% change	—	—	-2.0	—	—	—	—	—	—	—	—	0.7	—	—	—	—	—	—	—	-2.6	—	0.4
	Median calories per	Absolute change	—	—	4.0	—	—	—	—	—	—	—	—	4.0	—	—	—	—	—	—	—	8.0	—	-2.0
	100 g/mL (kcal)	% change	—	—	9.1	—	—	—	—	—	—	—	—	10.0	—	—	—	—	—	—	—	22.2	—	-20.0
	Median sodium per	Absolute change	—	—	16.0	—	—	—	—	—	—	—	—	-6.8	—	—	—	—	—	—	—	10.0	—	5.3
	100 g/mL (mg)	% change	—	—	6.3	—	—	—	—	—	—	—	—	-2.5	—	—	—	—	—	—	—	4.3	—	1.9
	Median saturated	Absolute change	—	—	0.2	—	—	—	—	—	—	—	—	0.0	—	—	—	—	—	—	—	0.0	—	0.0
	fat per 100 g/mL (g)	% change	—	—	100.0	—	—	—	—	—	—	—	—	25.0	—	—	—	—	—	—	—	0.0	—	N/A
	Median trans fat	Absolute change	—	—	0.0	—	—	—	—	—	—	—	—	0.0	—	—	—	—	—	—	—	0.0	—	0.0
	per 100 g/mL (g)	% change	—	—	N/A	—	—	—	—	—	—	—	—	N/A	—	—	—	—	—	—	—	N/A	—	N/A
	Median total sugars	Absolute change	—	—	0.0	—	—	—	—	—	—	—	—	0.2	—	—	—	—	—	—	—	0.0	—	0.2
	per 100 g/mL (g)	% change	—	—	0.0	—	—	—	—	—	—	—	—	50.0	—	—	—	—	—	—	—	0.0	—	42.9
	Median free sugars	Absolute change	—	—	-0.1	—	—	—	—	—	—	—	—	-0.3	—	—	—	—	—	—	—	0.2	—	0.2
	per 100 g/mL (g)	% change	—	—	-16.7	—	—	—	—	—	—	—	—	-82.3	—	—	—	—	—	—	—	N/A	—	N/A
Sugars and sweets (U)	Number of products	2013	0	0	0	0	2	0	0	11	0	0	12	116	0	33	28	0	0	3	0	38	13	1
		2017	0	0	0	0	4	0	0	17	0	0	8	132	0	49	32	0	0	3	0	55	15	0
	Mean Health Star	Absolute change	—	—	—	—	0.3	—	—	0.3	—	—	0.4	-0.1	—	-0.1	0.0	—	—	0.0	—	-0.2	0.0	—
	Rating	% change	—	—	—	—	20.0	—	—	16.8	—	—	58.8	-3.7	—	-10.6	-6.1	—	—	0.0	—	-12.7	0.0	—
	Median calories per	Absolute change	—	—	—	—	-33.3	—	—	-15.8	—	—	-74.4	14.7	—	23.8	3.4	—	—	0.0	—	16.7	0.0	—
	100 g/mL (kcal)	% change	—	—	—	—	-10.0	—	—	-4.3	—	—	-14.5	4.4	—	4.8	0.7	—	—	0.0	—	5.0	0.0	—
	Median sodium per	Absolute change	—	—	—	—	-16.7	—	—	21.7	—	—	-79.2	2.8	—	7.2	3.5	—	—	0.0	—	-10.0	0.0	—
	100 g/mL (mg)	% change	—	—	—	—	-25.0	—	—	12.5	—	—	-91.7	9.1	—	10.3	4.2	—	—	0.0	—	-54.4	0.0	—
	Median saturated	Absolute change	—	—	—	—	0.0	—	—	0.4	—	—	-4.5	0.0	—	2.3	-0.4	—	—	0.0	—	0.0	0.0	—
	fat per 100 g/mL (g)	% change	—	—	—	—	N/A	—	—	25.0	—	—	-27.3	N/A	—	16.3	-2.8	—	—	N/A	—	N/A	N/A	—
	Median trans fat	Absolute change	—	—	—	—	0.0	—	—	-1.3	—	—	-0.1	0.0	—	-0.2	0.0	—	—	0.0	—	0.0	0.0	—
	per 100 g/mL (g)	% change	—	—	—	—	N/A	—	—	-100.0	—	—	-100.0	N/A	—	-100.0	N/A	—	—	N/A	—	N/A	N/A	—
	Median total sugars	Absolute change	—	—	—	—	0.0	—	—	-6.5	—	—	3.1	3.6	—	-4.0	-2.3	—	—	0.0	—	0.0	0.0	—
	per 100 g/mL (g)	% change	—	—	—	—	0.0	—	—	-13.0	—	—	5.8	6.8	—	-7.4	-4.1	—	—	0.0	—	0.0	0.0	—
	Median free sugars	Absolute change	—	—	—	—	-5.9	—	—	-25.9	—	—	2.8	-2.0	—	-4.8	-2.6	—	—	0.0	—	-5.0	36.5	—
	per 100 g/mL (g)	% change	—	—	—	—	-7.7	—	—	-51.8	—	—	5.2	-3.8	—	-8.9	-4.7	—	—	0.0	—	-8.3	113.5	—
Confectionary (U1, U3, U4, U10, U11)	Number of products	2013	0	0	0	0	0	0	0	11	0	0	12	48	0	33	23	0	0	0	0	17	13	0
		2017	0	0	0	0	0	0	0	17	0	0	6	56	0	49	28	0	0	0	0	30	15	0
	Mean Health Star	Absolute change	—	—	—	—	—	—	—	0.3	—	—	0.5	0.1	—	-0.1	0.0	—	—	—	—	-0.1	0.0	—
	Rating	% change	—	—	—	—	—	—	—	16.8	—	—	64.7	5.8	—	-10.6	-0.3	—	—	—	—	-9.6	0.0	—
	Median calories per	Absolute change	—	—	—	—	—	—	—	-15.8	—	—	-31.7	-49.0	—	23.8	-3.9	—	—	—	—	25.7	0.0	—
	100 g/mL (kcal)	% change	—	—	—	—	—	—	—	-4.3	—	—	-6.2	-9.2	—	4.8	-0.7	—	—	—	—	7.9	0.0	—
	Median sodium per	Absolute change	—	—	—	—	—	—	—	21.7	—	—	-43.5	4.2	—	7.2	3.1	—	—	—	—	6.3	0.0	—
	100 g/mL (mg)	% change	—	—	—	—	—	—	—	12.5	—	—	-50.3	6.7	—	10.3	3.7	—	—	—	—	25.0	0.0	—
	Median saturated fat	Absolute change	—	—	—	—	—	—	—	0.4	—	—	3.2	-3.1	—	2.3	-0.3	—	—	—	—	0.0	0.0	—
	per 100 g/mL (g)	% change	—	—	—	—	—	—	—	25.0	—	—	19.2	-20.5	—	16.3	-2.0	—	—	—	—	N/A	N/A	—
	Median trans fat per	Absolute change	—	—	—	—	—	—	—	-1.3	—	—	-0.1	0.0	—	-0.2	0.1	—	—	—	—	0.0	0.0	—
	100 g/mL (g)	% change	—	—	—	—	—	—	—	-100.0	—	—	-100.0	N/A	—	-100.0	N/A	—	—	—	—	N/A	N/A	—
	Median total sugars	Absolute change	—	—	—	—	—	—	—	-6.5	—	—	-4.0	2.5	—	-4.0	-0.4	—	—	—	—	-9.5	0.0	—
	per 100 g/mL (g)	% change	—	—	—	—	—	—	—	-13.0	—	—	-7.5	5.1	—	-7.4	-0.8	—	—	—	—	-15.6	0.0	—
	Median free sugars	Absolute change	—	—	—	—	—	—	—	-25.9	—	—	-4.5	2.5	—	-4.8	-1.2	—	—	—	—	-9.9	36.5	—
	per 100 g/mL (g)	% change	—	—	—	—	—	—	—	-51.8	—	—	-8.3	5.3	—	-8.9	-2.3	—	—	—	—	-16.2	113.5	—
Sugars and syrups (U8, U9, U12, U14, U15)	Number of products	2013	0	0	0	0	2	0	0	0	0	0	0	68	0	0	5	0	0	3	0	21	0	1
		2017	0	0	0	0	4	0	0	0	0	0	2	76	0	0	4	0	0	3	0	25	0	0
	Mean Health Star	Absolute change	—	—	—	—	0.3	—	—	—	—	—	—	-0.1	—	—	-0.1	—	—	0.0	—	-0.2	—	—
	Rating	% change	—	—	—	—	20.0	—	—	—	—	—	—	-7.4	—	—	-3.8	—	—	0.0	—	-12.7	—	—
	Median calories per	Absolute change	—	—	—	—	-33.3	—	—	—	—	—	—	0.0	—	—	-16.7	—	—	0.0	—	0.0	—	—
	100 g/mL (kcal)	% change	—	—	—	—	-10.0	—	—	—	—	—	—	0.0	—	—	-4.8	—	—	0.0	—	0.0	—	—
	Median sodium per	Absolute change	—	—	—	—	-16.7	—	—	—	—	—	—	0.0	—	—	41.7	—	—	0.0	—	0.0	—	—
	100 g/mL (mg)	% change	—	—	—	—	-25.0	—	—	—	—	—	—	N/A	—	—	33.3	—	—	0.0	—	N/A	—	—
	Median saturated fat	Absolute change	—	—	—	—	0.0	—	—	—	—	—	—	0.0	—	—	0.0	—	—	0.0	—	0.0	—	—
	per 100 g/mL (g)	% change	—	—	—	—	N/A	—	—	—	—	—	—	N/A	—	—	N/A	—	—	N/A	—	N/A	—	—
	Median trans fat per	Absolute change	—	—	—	—	0.0	—	—	—	—	—	—	0.0	—	—	0.0	—	—	0.0	—	0.0	—	—
	100 g/mL (g)	% change	—	—	—	—	N/A	—	—	—	—	—	—	N/A	—	—	N/A	—	—	N/A	—	N/A	—	—
	Median total sugars	Absolute change	—	—	—	—	0.0	—	—	—	—	—	—	5.0	—	—	13.3	—	—	0.0	—	18.3	—	—
	per 100 g/mL (g)	% change	—	—	—	—	0.0	—	—	—	—	—	—	8.3	—	—	22.2	—	—	0.0	—	30.6	—	—
	Median free sugars	Absolute change	—	—	—	—	-5.9	—	—	—	—	—	—	-6.7	—	—	13.3	—	—	0.0	—	6.6	—	—
	per 100 g/mL (g)	% change	—	—	—	—	-7.7	—	—	—	—	—	—	-11.1	—	—	22.2	—	—	0.0	—	11.0	—	—
Vegetables (V)	Number of products	2013	2	0	8	0	5	0	0	33	0	0	1	145	0	0	0	0	0	0	0	92	0	1
		2017	1	0	6	0	8	0	0	9	0	0	3	200	0	0	0	0	0	0	0	106	0	4
	Mean Health Star	Absolute change	0.8	—	-0.4	—	-0.2	—	—	0.1	—	—	-1.2	0.3	—	—	—	—	—	—	—	0.0	—	-0.6
	Rating	% change	42.9	—	-11.1	—	-11.8	—	—	3.5	—	—	-23.3	8.0	—	—	—	—	—	—	—	-0.7	—	-17.9
	Median calories per	Absolute change	0.3	—	0.0	—	0.0	—	—	-20.0	—	—	13.3	0.0	—	—	—	—	—	—	—	0.3	—	16.7
	100 g/mL (kcal)	% change	1.4	—	0.0	—	0.0	—	—	-50.0	—	—	66.7	0.0	—	—	—	—	—	—	—	1.0	—	100.0

			A. Lassonde	Agropur	Campbell Soup	Canada Bread	Canada Dry Mott's	Coca-Cola	Danone	General Mills	George Weston	Kellogg	Kraft Heinz	Loblaw	Maple Leaf Foods	Mondelēz	Nestlé	Ocean Spray	Parmalat	PepsiCo	Saputo	Sobeys	Sun-Rype	Unilever
	**Median sodium per**	**Absolute change**	**-15.7**	—	**-4.2**	—	**0.0**	—	—	**-6.0**	—	—	**0.0**	**-129.3**	—	—	—	—	—	—	—	**-56.0**	—	**-33.3**
	**100 g/mL (mg)**	**% change**	**-8.5**	—	**-5.5**	—	**0.0**	—	—	**-4.0**	—	—	**0.0**	**-55.4**	—	—	—	—	—	—	—	**-31.1**	—	**-4.3**
	**Median saturated**	**Absolute change**	**0.0**	—	**0.0**	—	**0.0**	—	—	**0.0**	—	—	**0.0**	**0.0**	—	—	—	—	—	—	—	**0.0**	—	**0.0**
	**fat per 100 g/mL (g)**	**% change**	**N/A**	—	**N/A**	—	**N/A**	—	—	**N/A**	—	—	**N/A**	**N/A**	—	—	—	—	—	—	—	**N/A**	—	**N/A**
	**Median trans fat**	**Absolute change**	**0.0**	—	**0.0**	—	**0.0**	—	—	**0.0**	—	—	**0.0**	**0.0**	—	—	—	—	—	—	—	**0.0**	—	**0.0**
	**per 100 g/mL (g)**	**% change**	**N/A**	—	**N/A**	—	**N/A**	—	—	**N/A**	—	—	**N/A**	**N/A**	—	—	—	—	—	—	—	**N/A**	—	**N/A**
	**Median total sugars**	**Absolute change**	**0.0**	—	**-0.4**	—	**0.2**	—	—	**-1.2**	—	—	**2.6**	**0.5**	—	—	—	—	—	—	—	**0.4**	—	**6.7**
	**per 100 g/mL (g)**	**% change**	**1.4**	—	**-8.3**	—	**5.0**	—	—	**-37.5**	—	—	**108.3**	**31.9**	—	—	—	—	—	—	—	**17.6**	—	**N/A**
	**Median free sugars**	**Absolute change**	**0.0**	—	**0.0**	—	**-1.5**	—	—	**0.0**	—	—	**2.4**	**0.0**	—	—	—	—	—	—	—	**0.0**	—	**5.7**
	**per 100 g/mL (g)**	**% change**	**N/A**	—	**N/A**	—	**-100.0**	—	—	**N/A**	—	—	**N/A**	**N/A**	—	—	—	—	—	—	—	**N/A**	—	**N/A**
Vegetable	Number of products	2013	2	0	5	0	5	0	0	0	0	0	1	7	0	0	0	0	0	0	0	5	0	0
juice and		2017	1	0	5	0	8	0	0	0	0	0	1	10	0	0	0	0	0	0	0	8	0	0
vegetable	Mean Health Star	Absolute change	0.8	—	0.0	—	-0.2	—	—	—	—	—	0.0	0.1	—	—	—	—	—	—	—	0.1	—	—
drink (V7)	Rating	% change	42.9	—	0.0	—	-11.8	—	—	—	—	—	0.0	4.0	—	—	—	—	—	—	—	6.0	—	—
	Median calories per	Absolute change	0.3	—	0.0	—	0.0	—	—	—	—	—	0.0	0.0	—	—	—	—	—	—	—	-4.0	—	—
	100 g/mL (kcal)	% change	1.4	—	0.0	—	0.0	—	—	—	—	—	0.0	0.0	—	—	—	—	—	—	—	-16.7	—	—
	Median sodium per	Absolute change	-15.7	—	0.0	—	0.0	—	—	—	—	—	0.0	-18.0	—	—	—	—	—	—	—	-36.0	—	—
	100 g/mL (mg)	% change	-8.5	—	0.0	—	0.0	—	—	—	—	—	0.0	-9.4	—	—	—	—	—	—	—	-15.0	—	—
	Median saturated fat	Absolute change	0.0	—	0.0	—	0.0	—	—	—	—	—	0.0	0.0	—	—	—	—	—	—	—	0.0	—	—
	per 100 g/mL (g)	% change	N/A	—	N/A	—	N/A	—	—	—	—	—	N/A	N/A	—	—	—	—	—	—	—	N/A	—	—
	Median trans fat per	Absolute change	0.0	—	0.0	—	0.0	—	—	—	—	—	0.0	0.0	—	—	—	—	—	—	—	0.0	—	—
	100 g/mL (g)	% change	N/A	—	N/A	—	N/A	—	—	—	—	—	N/A	N/A	—	—	—	—	—	—	—	N/A	—	—
	Median total sugars	Absolute change	0.0	—	-0.4	—	0.2	—	—	—	—	—	0.0	0.4	—	—	—	—	—	—	—	0.0	—	—
	per 100 g/mL (g)	% change	1.4	—	-10.0	—	5.0	—	—	—	—	—	0.0	14.3	—	—	—	—	—	—	—	0.0	—	—
	Median free sugars	Absolute change	0.0	—	-0.6	—	-1.5	—	—	—	—	—	0.0	0.0	—	—	—	—	—	—	—	-0.6	—	—
	per 100 g/mL (g)	% change	N/A	—	-100.0	—	-100.0	—	—	—	—	—	N/A	N/A	—	—	—	—	—	—	—	-100.0	—	—
Vegetables	Number of products	2013	0	0	0	0	0	0	0	6	0	0	0	35	0	0	0	0	0	0	0	13	0	1
with sauce,		2017	0	0	0	0	0	0	0	2	0	0	2	45	0	0	0	0	0	0	0	21	0	4
pastes (V2,	Mean Health Star	Absolute change	—	—	—	—	—	—	—	0.7	—	—	—	0.2	—	—	—	—	—	—	—	0.1	—	-0.6
V9, V10,	Rating	% change	—	—	—	—	—	—	—	17.4	—	—	—	4.6	—	—	—	—	—	—	—	1.6	—	-17.9
V11, V12)	Median calories per	Absolute change	—	—	—	—	—	—	—	-42.1	—	—	—	-13.2	—	—	—	—	—	—	—	0.0	—	16.7
	100 g/mL (kcal)	% change	—	—	—	—	—	—	—	-53.9	—	—	—	-24.8	—	—	—	—	—	—	—	0.0	—	100.0
	Median sodium per	Absolute change	—	—	—	—	—	—	—	-87.0	—	—	—	0.0	—	—	—	—	—	—	—	0.0	—	-33.3
	100 g/mL (mg)	% change	—	—	—	—	—	—	—	-37.7	—	—	—	0.0	—	—	—	—	—	—	—	0.0	—	-4.3
	Median saturated fat	Absolute change	—	—	—	—	—	—	—	-0.7	—	—	—	0.0	—	—	—	—	—	—	—	0.0	—	0.0
	per 100 g/mL (g)	% change	—	—	—	—	—	—	—	-100.0	—	—	—	N/A	—	—	—	—	—	—	—	N/A	—	N/A
	Median trans fat per	Absolute change	—	—	—	—	—	—	—	0.0	—	—	—	0.0	—	—	—	—	—	—	—	0.0	—	0.0
	100 g/mL (g)	% change	—	—	—	—	—	—	—	N/A	—	—	—	N/A	—	—	—	—	—	—	—	N/A	—	N/A
	Median total sugars	Absolute change	—	—	—	—	—	—	—	0.9	—	—	—	-3.5	—	—	—	—	—	—	—	0.0	—	6.7
	per 100 g/mL (g)	% change	—	—	—	—	—	—	—	29.0	—	—	—	-70.0	—	—	—	—	—	—	—	0.0	—	N/A
	Median free sugars	Absolute change	—	—	—	—	—	—	—	-1.4	—	—	—	0.0	—	—	—	—	—	—	—	0.0	—	5.7
	per 100 g/mL (g)	% change	—	—	—	—	—	—	—	-100.0	—	—	—	N/A	—	—	—	—	—	—	—	N/A	—	N/A
Vegetables	Number of products	2013	0	0	3	0	0	0	0	27	0	0	0	103	0	0	0	0	0	0	0	74	0	0
without		2017	0	0	1	0	0	0	0	7	0	0	0	145	0	0	0	0	0	0	0	77	0	0
sauce (V1,	Mean Health Star	Absolute change	—	—	0.0	—	—	—	—	0.0	—	—	—	0.3	—	—	—	—	—	—	—	0.1	—	—
V3, V4, V5,	Rating	% change	—	—	0.0	—	—	—	—	0.5	—	—	—	8.8	—	—	—	—	—	—	—	1.8	—	—
V8)	Median calories per	Absolute change	—	—	0.0	—	—	—	—	-16.0	—	—	—	0.0	—	—	—	—	—	—	—	-0.4	—	—
	100 g/mL (kcal)	% change	—	—	0.0	—	—	—	—	-44.4	—	—	—	0.0	—	—	—	—	—	—	—	-1.2	—	—
	Median sodium per	Absolute change	—	—	0.0	—	—	—	—	48.0	—	—	—	-113.4	—	—	—	—	—	—	—	-47.5	—	—
	100 g/mL (mg)	% change	—	—	0.0	—	—	—	—	54.5	—	—	—	-61.6	—	—	—	—	—	—	—	-38.3	—	—
	Median saturated fat	Absolute change	—	—	0.0	—	—	—	—	0.0	—	—	—	0.0	—	—	—	—	—	—	—	0.0	—	—
	per 100 g/mL (g)	% change	—	—	N/A	—	—	—	—	N/A	—	—	—	N/A	—	—	—	—	—	—	—	N/A	—	—
	Median trans fat per	Absolute change	—	—	0.0	—	—	—	—	0.0	—	—	—	0.0	—	—	—	—	—	—	—	0.0	—	—
	100 g/mL (g)	% change	—	—	N/A	—	—	—	—	N/A	—	—	—	N/A	—	—	—	—	—	—	—	N/A	—	—
	Median total sugars	Absolute change	—	—	0.0	—	—	—	—	-1.2	—	—	—	0.4	—	—	—	—	—	—	—	0.4	—	—
	per 100 g/mL (g)	% change	—	—	0.0	—	—	—	—	-37.5	—	—	—	36.0	—	—	—	—	—	—	—	25.0	—	—
	Median free sugars	Absolute change	—	—	0.0	—	—	—	—	0.0	—	—	—	0.0	—	—	—	—	—	—	—	0.0	—	—
	per 100 g/mL (g)	% change	—	—	N/A	—	—	—	—	N/A	—	—	—	N/A	—	—	—	—	—	—	—	N/A	—	—

[1]Food categories are based on those defined in Health Canada's Table of Reference Amounts for Foods (TRA), listed in brackets. Values at the TRA major food category level are shown in bold. TRA minor food categories that were combined are indicated in brackets. If none of the sampled products fell into that food category, it was omitted from the table.

Supplementary Table 3. Mean and median absolute and percentage changes in Health Star Ratings (HSRs), calories, sodium, saturated fat, trans fat, total sugars and free sugars per 100 g (or mL) in products offered by each company that were matched between 2013 and 2017, presented by food category. [1]

				A. Lassonde	Agropur	Campbell Soup	Canada Bread	Canada Dry Mott's	Coca-Cola	Danone	General Mills	George Weston	Kellogg	Kraft Heinz	Loblaw	Maple Leaf Foods	Mondelēz	Nestlé	Ocean Spray	Parmalat	PepsiCo	Saputo	Sobeys	Sun-Rype	Unilever
**Total product**	**Number of products**			35	53	116	54	40	68	37	194	65	84	292	1479	90	108	158	25	41	199	30	754	19	133
**portfolio**	**HSR**	**Absolute**	**Mean**	-0.2	-0.2	0.0	0.0	0.0	0.1	0.0	0.0	0.0	0.0	0.7	0.1	0.1	0.2	0.1	0.6	0.0	0.0	-0.3	0.1	0.0	0.1
		**change**	**Median**	0.0	0.0	0.0	0.0	0.0	0.0	0.0	0.0	0.0	0.0	0.0	0.0	0.0	0.0	0.0	0.0	0.0	0.0	0.0	0.0	0.0	0.0
		**Percent**	**Mean**	-3.2	8.9	-0.4	-0.2	1.7	6.0	0.3	2.0	-0.5	1.5	44.4	8.8	8.6	20.2	6.9	34.1	22.4	0.9	-14.3	7.0	0.0	8.2
		**change**	**Median**	0.0	0.0	0.0	0.0	0.0	0.0	0.0	0.0	0.0	0.0	0.0	0.0	0.0	0.0	0.0	0.0	0.0	0.0	0.0	0.0	0.0	0.0
	**Calories**	**Absolute**	**Mean**	0.0	-5.2	0.4	4.4	2.1	-0.1	1.2	-2.8	6.8	-3.8	-4.1	-2.2	-3.8	-3.9	-3.9	-5.3	2.4	-1.7	-3.1	0.8	-0.7	-3.6
	**(kcal per**	**change**	**Median**	0.0	0.0	0.0	0.0	0.0	0.0	0.0	0.0	0.0	0.0	0.0	0.0	0.0	0.0	0.0	0.0	0.0	0.0	0.0	0.0	0.0	0.0
	**100 g/mL)**	**Percent**	**Mean**	0.1	-2.1	0.6	1.5	N/A	-0.3	2.5	-1.4	2.3	-1.1	-0.8	N/A	-1.4	-0.8	-1.7	-5.2	2.3	-0.5	-2.5	1.1	-1.5	0.1
		**change**	**Median**	0.0	0.0	0.0	0.0	0.0	0.0	0.0	0.0	0.0	0.0	0.0	0.0	0.0	0.0	0.0	0.0	0.0	0.0	0.0	0.0	0.0	0.0
	**Sodium**	**Absolute**	**Mean**	-2.1	44.4	12.1	-18.3	-49.5	-0.2	6.5	-50.3	-15.2	-15.7	-25.5	-32.9	-48.8	-48.0	-9.4	-2.4	8.9	-15.1	-1.7	-26.2	0.6	-16.3
	**(mg per**	**change**	**Median**	-2.0	0.0	0.0	0.0	0.0	0.0	8.6	0.0	0.0	0.0	0.0	0.0	0.0	-20.8	0.0	0.0	0.0	0.0	0.0	0.0	0.0	-4.0
	**100 g/mL)**	**Percent**	**Mean**	-9.8	3.6	3.6	-2.9	-0.2	-2.9	15.2	-4.2	-1.0	-3.8	N/A	N/A	-5.9	-6.6	N/A	-15.4	0.3	N/A	-0.8	N/A	2.5	-4.4
		**change**	**Median**	-8.4	0.0	0.0	0.0	0.0	0.0	18.8	0.0	0.0	0.0	0.0	0.0	0.0	-3.5	0.0	0.0	0.0	0.0	0.0	0.0	0.0	-2.0
	**Saturated**	**Absolute**	**Mean**	0.0	0.7	0.0	0.0	0.0	0.0	0.0	0.3	0.2	0.4	-0.2	0.0	0.0	-1.1	-0.4	0.0	0.2	0.0	0.1	0.0	0.0	-0.2
	**fat (g per**	**change**	**Median**	0.0	0.0	0.0	0.0	0.0	0.0	0.0	0.0	0.0	0.0	0.0	0.0	0.0	-0.4	0.0	0.0	0.0	0.0	0.0	0.0	0.0	0.0
	**100 g/mL)**	**Percent**	**Mean**	N/A	N/A	N/A	19.2	N/A	N/A	-0.8	N/A	N/A	N/A	N/A	N/A	-4.1	-23.0	-10.5	N/A	0.4	0.2	2.7	N/A	N/A	N/A
		**change**	**Median**	N/A	0.0	0.0	0.0	N/A	N/A	0.0	0.0	0.0	0.0	0.0	0.0	0.0	-11.1	0.0	N/A	0.0	0.0	0.0	0.0	N/A	0.0
	**Trans fat**	**Absolute**	**Mean**	0.0	0.0	0.0	0.0	0.0	0.0	0.0	-0.4	0.0	-0.3	0.0	0.0	0.0	-0.2	-0.1	0.0	0.1	0.0	0.0	0.0	0.0	0.0
	**(g per 100**	**change**	**Median**	0.0	0.0	0.0	0.0	0.0	0.0	0.0	0.0	0.0	0.0	0.0	0.0	0.0	0.0	0.0	0.0	0.0	0.0	0.0	0.0	0.0	0.0
	**g/mL)**	**Percent**	**Mean**	N/A	23.7	N/A	N/A	N/A	N/A	N/A	N/A	N/A	N/A	N/A	N/A	N/A	N/A	N/A	N/A	18.1	N/A	2.9	N/A	N/A	N/A
		**change**	**Median**	N/A	0.0	0.0	0.0	N/A	N/A	N/A	-90.0	0.0	N/A	0.0	0.0	0.0	-3.1	0.0	N/A	0.0	0.0	0.0	0.0	N/A	-33.3
	**Total**	**Absolute**	**Mean**	-0.1	-0.4	-0.1	0.6	0.4	0.0	0.0	-0.8	0.2	-0.1	-0.1	0.2	0.0	0.4	-0.3	0.6	0.2	0.1	0.2	0.2	-0.2	0.0
	**sugars (g**	**change**	**Median**	0.0	0.0	0.0	0.0	0.0	0.0	0.0	0.0	0.0	0.0	0.0	0.0	0.0	0.0	0.0	0.0	0.0	0.0	0.0	0.0	0.0	0.0
	**per 100**	**Percent**	**Mean**	-1.3	-10.4	N/A	N/A	-0.6	-0.1	3.7	1.5	0.9	N/A	N/A	N/A	N/A	2.4	-0.3	-8.6	11.4	N/A	N/A	N/A	-1.5	N/A
	**g/mL)**	**change**	**Median**	0.0	0.0	0.0	0.0	0.0	0.0	0.0	0.0	0.0	0.0	0.0	0.0	0.0	0.0	0.0	0.0	0.0	0.0	0.0	0.0	0.0	0.0
	**Free**	**Absolute**	**Mean**	-0.1	-0.3	-0.1	1.2	-0.3	0.0	0.0	-1.4	0.7	-0.1	-0.1	-0.3	-0.1	0.2	-0.6	2.0	-0.3	0.2	-0.1	0.1	15.6	-0.2
	**sugars (g**	**change**	**Median**	0.0	0.0	0.0	0.5	0.0	0.0	-0.3	0.0	0.4	0.0	0.0	0.0	0.0	0.0	-0.2	0.0	0.0	0.0	0.0	0.0	0.0	0.0
	**per 100**	**Percent**	**Mean**	-1.4	-19.5	N/A	N/A	-19.9	-0.1	N/A	N/A	N/A	N/A	N/A	N/A	N/A	1.0	N/A	-2.4	N/A	N/A	-22.5	N/A	55.4	N/A
	**g/mL)**	**change**	**Median**	0.0	-9.0	-1.9	16.6	-0.3	0.0	-2.7	-0.1	13.1	0.2	0.0	0.0	-0.1	0.0	-3.0	0.0	-2.1	0.0	-0.6	0.0	0.0	0.0
**Bakery**	**Number of products**			0	1	11	53	0	0	0	46	64	44	0	206	0	81	0	0	0	29	0	107	0	0
**products (A)**	**HSR**	**Absolute**	**Mean**	—	0.0	0.0	0.0	—	—	—	0.1	0.0	0.0	—	0.1	—	0.3	—	—	—	0.0	—	0.1	—	—
		**change**	**Median**	—	0.0	0.0	0.0	—	—	—	0.0	0.0	0.0	—	0.0	—	0.0	—	—	—	0.0	—	0.0	—	—
		**Percent**	**Mean**	—	0.0	-1.8	-0.2	—	—	—	9.2	-0.2	0.5	—	6.3	—	27.6	—	—	—	-1.3	—	5.1	—	—
		**change**	**Median**	—	0.0	0.0	0.0	—	—	—	0.0	0.0	0.0	—	0.0	—	0.0	—	—	—	0.0	—	0.0	—	—
	**Calories**	**Absolute**	**Mean**	—	0.0	4.5	4.4	—	—	—	-5.8	7.0	-6.6	—	-1.6	—	-1.5	—	—	—	-3.2	—	0.5	—	—
	**(kcal per**	**change**	**Median**	—	0.0	0.0	0.0	—	—	—	0.0	0.0	0.0	—	0.0	—	0.0	—	—	—	0.0	—	0.0	—	—
	**100 g/mL)**	**Percent**	**Mean**	—	0.0	1.1	1.5	—	—	—	-1.2	2.3	-1.8	—	-0.2	—	-0.3	—	—	—	-0.7	—	0.3	—	—
		**change**	**Median**	—	0.0	0.0	0.0	—	—	—	0.0	0.0	0.0	—	0.0	—	0.0	—	—	—	0.0	—	0.0	—	—
	**Sodium**	**Absolute**	**Mean**	—	0.0	27.3	-18.6	—	—	—	-25.2	-15.4	-19.7	—	-39.4	—	-66.6	—	—	—	-6.1	—	-33.8	—	—
	**(mg per**	**change**	**Median**	—	0.0	0.0	0.0	—	—	—	0.0	0.0	0.0	—	0.0	—	-35.7	—	—	—	0.0	—	0.0	—	—
	**100 g/mL)**	**Percent**	**Mean**	—	0.0	3.4	-2.9	—	—	—	-1.6	-0.9	-6.5	—	-7.4	—	-11.5	—	—	—	-1.0	—	-6.1	—	—
		**change**	**Median**	—	0.0	0.0	0.0	—	—	—	0.0	0.0	0.0	—	0.0	—	-8.3	—	—	—	0.0	—	0.0	—	—
	**Saturated**	**Absolute**	**Mean**	—	0.0	0.0	0.0	—	—	—	0.3	0.2	0.6	—	0.1	—	-1.5	—	—	—	0.1	—	-0.1	—	—
	**fat (g per**	**change**	**Median**	—	0.0	0.0	0.0	—	—	—	0.0	0.0	0.0	—	0.0	—	-1.0	—	—	—	0.0	—	0.0	—	—
	**100 g/mL)**	**Percent**	**Mean**	—	0.0	0.0	19.6	—	—	—	11.2	15.2	10.6	—	5.7	—	-27.7	—	—	—	0.6	—	0.4	—	—
		**change**	**Median**	—	0.0	0.0	0.0	—	—	—	0.0	0.0	0.0	—	0.0	—	-33.3	—	—	—	0.0	—	0.0	—	—
	**Trans fat**	**Absolute**	**Mean**	—	0.0	0.0	0.0	—	—	—	-0.6	0.0	-0.6	—	0.0	—	-0.2	—	—	—	0.0	—	0.0	—	—
	**(g per 100**	**change**	**Median**	—	0.0	0.0	0.0	—	—	—	0.0	0.0	0.0	—	0.0	—	0.0	—	—	—	0.0	—	0.0	—	—
	**g/mL)**	**Percent**	**Mean**	—	N/A	N/A	-3.2	—	—	—	-61.1	-3.1	-75.0	—	-8.2	—	-71.3	—	—	—	-100.0	—	-22.1	—	—
		**change**	**Median**	—	N/A	N/A	0.0	—	—	—	-90.0	0.0	-100.0	—	0.0	—	-100.0	—	—	—	-100.0	—	0.0	—	—
	**Total**	**Absolute**	**Mean**	—	0.0	0.5	0.6	—	—	—	-2.3	0.1	0.2	—	-0.2	—	0.0	—	—	—	0.6	—	0.5	—	—
	**sugars (g**	**change**	**Median**	—	0.0	0.0	0.0	—	—	—	0.0	0.0	0.0	—	0.0	—	0.0	—	—	—	0.0	—	0.0	—	—
	**per 100**	**Percent**	**Mean**	—	0.0	0.0	5.9	—	—	—	-5.8	0.3	3.0	—	-0.3	—	2.1	—	—	—	4.3	—	1.5	—	—
	**g/mL)**	**change**	**Median**	—	0.0	0.0	0.0	—	—	—	0.0	0.0	0.0	—	0.0	—	0.0	—	—	—	0.0	—	0.0	—	—
	**Free**	**Absolute**	**Mean**	—	-0.3	0.4	1.2	—	—	—	-2.4	0.6	0.0	—	0.1	—	-0.1	—	—	—	1.8	—	1.0	—	—
	**sugars (g**	**change**	**Median**	—	-0.3	0.0	0.5	—	—	—	0.0	0.4	0.0	—	0.0	—	0.0	—	—	—	0.1	—	0.4	—	—
	**per 100**	**Percent**	**Mean**	—	-9.0	-0.9	58.4	—	—	—	-10.0	33.6	4.9	—	9.6	—	0.7	—	—	—	11.5	—	9.2	—	—
	**g/mL)**	**change**	**Median**	—	-9.0	0.0	11.9	—	—	—	-8.1	12.7	0.2	—	0.0	—	-0.6	—	—	—	4.5	—	7.2	—	—
Baked goods,	Number of products			0	0	3	15	0	0	0	19	3	21	0	118	0	44	0	0	0	0	0	56	0	0
pastries and	HSR	Absolute	Mean	—	—	0.0	-0.1	—	—	—	0.1	-0.7	-0.1	—	0.1	—	0.3	—	—	—	—	—	0.0	—	—
other		change	Median	—	—	0.0	0.0	—	—	—	0.0	-0.5	0.0	—	0.0	—	0.0	—	—	—	—	—	0.0	—	—
sweetened		Percent	Mean	—	—	0.0	-2.2	—	—	—	9.8	-44.4	-6.2	—	5.3	—	33.9	—	—	—	—	—	4.0	—	—
bakery		change (%)	Median	—	—	0.0	0.0	—	—	—	0.0	-33.3	0.0	—	0.0	—	0.0	—	—	—	—	—	0.0	—	—
products (A4,	Calories	Absolute	Mean	—	—	16.7	5.9	—	—	—	-7.3	38.3	-4.4	—	-0.2	—	1.5	—	—	—	—	—	1.5	—	—
A5, A6, A7,	(kcal per	change	Median	—	—	0.0	0.0	—	—	—	0.0	42.3	0.0	—	0.0	—	0.0	—	—	—	—	—	0.0	—	—
A8, A9, A10,	100 g/mL)	Percent	Mean	—	—	3.7	1.4	—	—	—	-1.4	10.6	-1.5	—	0.2	—	0.4	—	—	—	—	—	0.5	—	—
A14, A15,		change (%)	Median	—	—	0.0	0.0	—	—	—	0.0	12.0	0.0	—	0.0	—	0.0	—	—	—	—	—	0.0	—	—
A17, A23)	Sodium	Absolute	Mean	—	—	0.0	-19.7	—	—	—	-23.1	15.8	-7.4	—	-41.8	—	-42.0	—	—	—	—	—	-27.7	—	—
	(mg per	change	Median	—	—	0.0	-11.6	—	—	—	0.0	14.1	0.0	—	0.0	—	-14.1	—	—	—	—	—	0.0	—	—
	100 g/mL)	Percent	Mean	—	—	0.0	-3.8	—	—	—	-3.7	3.4	-1.3	—	-8.9	—	-10.8	—	—	—	—	—	-2.5	—	—

				A. Lassonde	Agropur	Campbell Soup	Canada Bread	Canada Dry Mott's	Coca-Cola	Danone	General Mills	George Weston	Kellogg	Kraft Heinz	Loblaw	Maple Leaf Foods	Mondelēz	Nestlé	Ocean Spray	Parmalat	PepsiCo	Saputo	Sobeys	Sun-Rype	Unilever
		change (%)	Median	—	—	0.0	-3.2	—	—	—	0.0	4.0	0.0	—	0.0	—	-4.4	—	—	—	—	—	0.0	—	—
	Saturated	Absolute	Mean	—	—	0.0	-0.5	—	—	—	0.6	1.5	1.3	—	0.2	—	-2.2	—	—	—	—	—	-0.2	—	—
	fat (g per	change	Median	—	—	0.0	0.0	—	—	—	0.0	1.4	0.0	—	0.0	—	-1.6	—	—	—	—	—	0.0	—	—
	100 g/mL)	Percent	Mean	—	—	0.0	-5.2	—	—	—	18.6	28.0	26.2	—	11.4	—	-28.1	—	—	—	—	—	-0.7	—	—
		change (%)	Median	—	—	0.0	0.0	—	—	—	0.0	28.6	0.0	—	0.0	—	-33.3	—	—	—	—	—	0.0	—	—
	Trans fat (g	Absolute	Mean	—	—	0.0	0.1	—	—	—	-1.0	0.0	-1.2	—	0.0	—	-0.3	—	—	—	—	—	0.0	—	—
	per 100	change	Median	—	—	0.0	0.0	—	—	—	0.0	0.0	0.0	—	0.0	—	0.0	—	—	—	—	—	0.0	—	—
	g/mL)	Percent	Mean	—	—	N/A	-3.4	—	—	—	-50.0	-8.3	-75.0	—	-3.6	—	-76.7	—	—	—	—	—	-7.9	—	—
		change (%)	Median	—	—	N/A	0.0	—	—	—	-70.0	0.0	-100.0	—	0.0	—	-100.0	—	—	—	—	—	0.0	—	—
	Total	Absolute	Mean	—	—	0.0	1.9	—	—	—	-0.2	5.0	-0.4	—	-0.2	—	0.0	—	—	—	—	—	0.4	—	—
	sugars (g	change	Median	—	—	0.0	1.3	—	—	—	0.0	4.2	0.0	—	0.0	—	0.0	—	—	—	—	—	0.0	—	—
	per 100	Percent	Mean	—	—	0.0	3.8	—	—	—	0.6	17.8	-0.6	—	-0.9	—	0.5	—	—	—	—	—	2.2	—	—
	g/mL)	change (%)	Median	—	—	0.0	0.0	—	—	—	0.0	10.0	0.0	—	0.0	—	0.0	—	—	—	—	—	0.0	—	—
	Free sugars	Absolute	Mean	—	—	0.0	3.2	—	—	—	-0.9	7.7	-0.1	—	0.4	—	-0.2	—	—	—	—	—	1.2	—	—
	(g per 100	change	Median	—	—	0.0	2.1	—	—	—	0.0	8.4	0.0	—	0.0	—	0.0	—	—	—	—	—	0.8	—	—
	g/mL)	Percent	Mean	—	—	0.0	5.1	—	—	—	-1.8	29.0	6.0	—	5.1	—	-0.6	—	—	—	—	—	10.2	—	—
		change (%)	Median	—	—	0.0	4.6	—	—	—	0.0	30.8	0.2	—	0.0	—	0.0	—	—	—	—	—	9.6	—	—
Bread (A1, A2,	Number of products			0	0	0	38	0	0	0	9	56	0	0	22	0	0	0	0	0	0	0	18	0	0
A3, A24)	HSR	Absolute	Mean	—	—	—	0.0	—	—	—	0.2	0.1	—	—	0.0	—	—	—	—	—	—	—	0.3	—	—
		change	Median	—	—	—	0.0	—	—	—	0.0	0.0	—	—	0.0	—	—	—	—	—	—	—	0.0	—	—
		Percent	Mean	—	—	—	0.6	—	—	—	10.6	2.8	—	—	1.2	—	—	—	—	—	—	—	12.5	—	—
		change (%)	Median	—	—	—	0.0	—	—	—	0.0	0.0	—	—	0.0	—	—	—	—	—	—	—	0.0	—	—
	Calories	Absolute	Mean	—	—	—	3.9	—	—	—	-13.2	3.3	—	—	-5.8	—	—	—	—	—	—	—	2.3	—	—
	(kcal per	change	Median	—	—	—	0.0	—	—	—	0.0	0.0	—	—	0.0	—	—	—	—	—	—	—	0.0	—	—
	100 g/mL)	Percent	Mean	—	—	—	1.6	—	—	—	-3.6	1.4	—	—	-1.5	—	—	—	—	—	—	—	1.2	—	—
		change (%)	Median	—	—	—	0.0	—	—	—	0.0	0.0	—	—	0.0	—	—	—	—	—	—	—	0.0	—	—
	Sodium	Absolute	Mean	—	—	—	-18.2	—	—	—	-116.3	-21.1	—	—	-11.7	—	—	—	—	—	—	—	-68.6	—	—
	(mg per	change	Median	—	—	—	0.0	—	—	—	-138.5	0.0	—	—	0.0	—	—	—	—	—	—	—	-41.6	—	—
	100 g/mL)	Percent	Mean	—	—	—	-2.6	—	—	—	-12.7	-1.7	—	—	-1.6	—	—	—	—	—	—	—	-10.8	—	—
		change (%)	Median	—	—	—	0.0	—	—	—	-17.8	0.0	—	—	0.0	—	—	—	—	—	—	—	-6.6	—	—
	Saturated	Absolute	Mean	—	—	—	0.2	—	—	—	0.0	0.1	—	—	0.0	—	—	—	—	—	—	—	-0.1	—	—
	fat (g per	change	Median	—	—	—	0.0	—	—	—	0.0	0.0	—	—	0.0	—	—	—	—	—	—	—	0.0	—	—
	100 g/mL)	Percent	Mean	—	—	—	29.4	—	—	—	13.5	15.3	—	—	-1.9	—	—	—	—	—	—	—	-0.9	—	—
		change (%)	Median	—	—	—	0.0	—	—	—	0.0	0.0	—	—	0.0	—	—	—	—	—	—	—	0.0	—	—
	Trans fat (g	Absolute	Mean	—	—	—	0.0	—	—	—	-0.9	0.0	—	—	0.0	—	—	—	—	—	—	—	0.0	—	—
	per 100	change	Median	—	—	—	0.0	—	—	—	-0.7	0.0	—	—	0.0	—	—	—	—	—	—	—	0.0	—	—
	g/mL)	Percent	Mean	—	—	—	0.0	—	—	—	-78.3	0.0	—	—	-40.6	—	—	—	—	—	—	—	-100.0	—	—
		change (%)	Median	—	—	—	0.0	—	—	—	-90.0	0.0	—	—	-40.6	—	—	—	—	—	—	—	-100.0	—	—
	Total	Absolute	Mean	—	—	—	0.1	—	—	—	0.2	-0.2	—	—	-0.1	—	—	—	—	—	—	—	0.1	—	—
	sugars (g	change	Median	—	—	—	0.0	—	—	—	0.0	0.0	—	—	0.0	—	—	—	—	—	—	—	0.0	—	—
	per 100	Percent	Mean	—	—	—	6.6	—	—	—	4.2	-0.7	—	—	6.9	—	—	—	—	—	—	—	2.1	—	—
	g/mL)	change (%)	Median	—	—	—	0.0	—	—	—	0.0	0.0	—	—	0.0	—	—	—	—	—	—	—	0.0	—	—
	Free sugars	Absolute	Mean	—	—	—	0.5	—	—	—	-1.2	0.3	—	—	-0.4	—	—	—	—	—	—	—	-0.2	—	—
	(g per 100	change	Median	—	—	—	0.5	—	—	—	-0.1	0.4	—	—	0.0	—	—	—	—	—	—	—	0.4	—	—
	g/mL)	Percent	Mean	—	—	—	78.2	—	—	—	-13.5	34.8	—	—	66.2	—	—	—	—	—	—	—	12.9	—	—
		change (%)	Median	—	—	—	18.5	—	—	—	-18.0	12.7	—	—	0.6	—	—	—	—	—	—	—	12.5	—	—
Crackers,	Number of products			0	1	8	0	0	0	0	2	5	5	0	54	0	37	0	0	0	13	0	19	0	0
croutons and	HSR	Absolute	Mean	—	0.0	-0.1	—	—	—	—	0.3	-0.1	0.6	—	0.2	—	0.4	—	—	—	0.0	—	0.1	—	—
rice cakes		change	Median	—	0.0	0.0	—	—	—	—	0.3	0.0	0.5	—	0.0	—	0.0	—	—	—	0.0	—	0.0	—	—
(A11, A12,		Percent	Mean	—	0.0	-2.5	—	—	—	—	12.5	-6.7	22.0	—	10.7	—	20.1	—	—	—	-1.5	—	2.5	—	—
A13, A16,		change (%)	Median	—	0.0	0.0	—	—	—	—	12.5	0.0	20.0	—	0.0	—	0.0	—	—	—	0.0	—	0.0	—	—
A21, A25)	Calories	Absolute	Mean	—	0.0	0.0	—	—	—	—	-18.5	30.0	0.0	—	-2.7	—	-5.2	—	—	—	0.0	—	0.0	—	—
	(kcal per	change	Median	—	0.0	0.0	—	—	—	—	-18.5	0.0	0.0	—	0.0	—	0.0	—	—	—	0.0	—	0.0	—	—
	100 g/mL)	Percent	Mean	—	0.0	0.1	—	—	—	—	-3.8	7.5	0.0	—	-0.6	—	-1.2	—	—	—	0.0	—	0.0	—	—
		change (%)	Median	—	0.0	0.0	—	—	—	—	-3.8	0.0	0.0	—	0.0	—	0.0	—	—	—	0.0	—	0.0	—	—
	Sodium	Absolute	Mean	—	0.0	37.5	—	—	—	—	-9.3	30.0	0.0	—	-45.6	—	-95.8	—	—	—	-13.8	—	-4.5	—	—
	(mg per	change	Median	—	0.0	0.0	—	—	—	—	-9.3	0.0	0.0	—	0.0	—	-100.0	—	—	—	0.0	—	0.0	—	—
	100 g/mL)	Percent	Mean	—	0.0	4.7	—	—	—	—	-2.2	4.8	0.2	—	-4.8	—	-12.2	—	—	—	-2.3	—	-1.4	—	—
		change (%)	Median	—	0.0	0.0	—	—	—	—	-2.2	0.0	0.0	—	0.0	—	-9.1	—	—	—	0.0	—	0.0	—	—
	Saturated	Absolute	Mean	—	0.0	0.0	—	—	—	—	0.0	0.5	-0.3	—	-0.2	—	-0.7	—	—	—	0.0	—	0.0	—	—
	fat (g per	change	Median	—	0.0	0.0	—	—	—	—	0.0	0.0	-0.5	—	0.0	—	-0.5	—	—	—	0.0	—	0.0	—	—
	100 g/mL)	Percent	Mean	—	0.0	0.0	—	—	—	—	0.0	6.7	-29.6	—	-5.9	—	-27.2	—	—	—	-2.3	—	0.0	—	—
		change (%)	Median	—	0.0	0.0	—	—	—	—	0.0	0.0	-29.2	—	0.0	—	-20.0	—	—	—	0.0	—	0.0	—	—
	Trans fat (g	Absolute	Mean	—	0.0	0.0	—	—	—	—	-0.2	0.0	0.0	—	0.0	—	-0.1	—	—	—	0.0	—	0.0	—	—
	per 100	change	Median	—	0.0	0.0	—	—	—	—	-0.2	0.0	0.0	—	0.0	—	0.0	—	—	—	0.0	—	0.0	—	—
	g/mL)	Percent	Mean	—	N/A	N/A	—	—	—	—	-100.0	0.0	N/A	—	-40.0	—	-57.3	—	—	—	N/A	—	N/A	—	—
		change (%)	Median	—	N/A	N/A	—	—	—	—	-100.0	0.0	N/A	—	0.0	—	-66.7	—	—	—	N/A	—	N/A	—	—
	Total	Absolute	Mean	—	0.0	0.6	—	—	—	—	0.0	0.0	2.0	—	-0.1	—	0.1	—	—	—	1.1	—	-0.3	—	—
	sugars (g	change	Median	—	0.0	0.0	—	—	—	—	0.0	0.0	0.0	—	0.0	—	0.0	—	—	—	0.0	—	0.0	—	—
	per 100	Percent	Mean	—	0.0	0.0	—	—	—	—	N/A	0.0	25.0	—	-1.2	—	5.1	—	—	—	10.0	—	-10.0	—	—
	g/mL)	change (%)	Median	—	0.0	0.0	—	—	—	—	N/A	0.0	0.0	—	0.0	—	0.0	—	—	—	0.0	—	0.0	—	—
	Free sugars	Absolute	Mean	—	-0.3	0.5	—	—	—	—	0.0	0.0	1.7	—	-0.2	—	-0.1	—	—	—	0.9	—	-0.1	—	—
	(g per 100	change	Median	—	-0.3	0.0	—	—	—	—	0.0	0.0	0.0	—	-0.1	—	-0.1	—	—	—	0.0	—	0.0	—	—

				A. Lassonde	Agropur	Campbell Soup	Canada Bread	Canada Dry Mott's	Coca-Cola	Danone	General Mills	George Weston	Kellogg	Kraft Heinz	Loblaw	Maple Leaf Foods	Mondelēz	Nestlé	Ocean Spray	Parmalat	PepsiCo	Saputo	Sobeys	Sun-Rype	Unilever
	g/mL)	Percent	Mean	—	-9.0	-3.8	—	—	—	—	N/A	-0.7	26.6	—	-5.6	—	3.2	—	—	—	16.6	—	-1.3	—	—
		change (%)	Median	—	-9.0	-3.8	—	—	—	—	N/A	-0.7	-1.0	—	-4.6	—	-3.8	—	—	—	0.1	—	-0.6	—	—
Grain-based,	Number of products			0	0	0	0	0	0	0	16	0	18	0	12	0	0	0	0	0	16	0	14	0	0
protein and	HSR	Absolute	Mean	—	—	—	—	—	—	—	0.2	—	0.0	—	0.2	—	—	—	—	—	0.0	—	0.1	—	—
energy bars		change	Median	—	—	—	—	—	—	—	0.0	—	0.0	—	0.3	—	—	—	—	—	0.0	—	0.0	—	—
(A18, A19,		Percent	Mean	—	—	—	—	—	—	—	7.2	—	2.2	—	6.4	—	—	—	—	—	-1.0	—	3.2	—	—
A20)		change (%)	Median	—	—	—	—	—	—	—	0.0	—	0.0	—	10.0	—	—	—	—	—	0.0	—	0.0	—	—
	Calories	Absolute	Mean	—	—	—	—	—	—	—	1.8	—	-10.9	—	-2.4	—	—	—	—	—	-5.8	—	-4.8	—	—
	(kcal per	change	Median	—	—	—	—	—	—	—	0.0	—	-2.4	—	0.0	—	—	—	—	—	0.0	—	0.0	—	—
	100 g/mL)	Percent	Mean	—	—	—	—	—	—	—	0.6	—	-2.7	—	-0.6	—	—	—	—	—	-1.4	—	-1.1	—	—
		change (%)	Median	—	—	—	—	—	—	—	0.0	—	-0.5	—	0.0	—	—	—	—	—	0.0	—	0.0	—	—
	Sodium	Absolute	Mean	—	—	—	—	—	—	—	21.5	—	-39.6	—	-38.8	—	—	—	—	—	0.1	—	-53.1	—	—
	(mg per	change	Median	—	—	—	—	—	—	—	0.0	—	-21.4	—	-34.0	—	—	—	—	—	0.0	—	-61.7	—	—
	100 g/mL)	Percent	Mean	—	—	—	—	—	—	—	7.4	—	-14.4	—	-14.4	—	—	—	—	—	0.0	—	-20.8	—	—
		change (%)	Median	—	—	—	—	—	—	—	0.0	—	-5.3	—	-14.1	—	—	—	—	—	0.0	—	-25.4	—	—
	Saturated	Absolute	Mean	—	—	—	—	—	—	—	0.0	—	0.1	—	0.6	—	—	—	—	—	0.2	—	0.2	—	—
	fat (g per	change	Median	—	—	—	—	—	—	—	0.0	—	0.0	—	0.0	—	—	—	—	—	0.0	—	0.0	—	—
	100 g/mL)	Percent	Mean	—	—	—	—	—	—	—	2.6	—	1.4	—	15.4	—	—	—	—	—	2.5	—	6.5	—	—
		change (%)	Median	—	—	—	—	—	—	—	0.0	—	0.0	—	0.0	—	—	—	—	—	0.0	—	0.0	—	—
	Trans fat (g	Absolute	Mean	—	—	—	—	—	—	—	0.0	—	0.0	—	0.0	—	—	—	—	—	-0.1	—	-0.1	—	—
	per 100	change	Median	—	—	—	—	—	—	—	0.0	—	0.0	—	0.0	—	—	—	—	—	0.0	—	0.0	—	—
	g/mL)	Percent	Mean	—	—	—	—	—	—	—	N/A	—	N/A	—	N/A	—	—	—	—	—	-100.0	—	-100.0	—	—
		change (%)	Median	—	—	—	—	—	—	—	N/A	—	N/A	—	N/A	—	—	—	—	—	-100.0	—	-100.0	—	—
	Total	Absolute	Mean	—	—	—	—	—	—	—	-6.4	—	0.4	—	-1.5	—	—	—	—	—	0.2	—	2.2	—	—
	sugars (g	change	Median	—	—	—	—	—	—	—	-9.0	—	0.0	—	0.0	—	—	—	—	—	0.0	—	1.6	—	—
	per 100	Percent	Mean	—	—	—	—	—	—	—	-17.9	—	2.4	—	-4.3	—	—	—	—	—	0.8	—	6.6	—	—
	g/mL)	change (%)	Median	—	—	—	—	—	—	—	-26.1	—	0.0	—	0.0	—	—	—	—	—	0.0	—	4.5	—	—
	Free sugars	Absolute	Mean	—	—	—	—	—	—	—	-5.1	—	-0.2	—	-1.2	—	—	—	—	—	2.5	—	2.9	—	—
	(g per 100	change	Median	—	—	—	—	—	—	—	-4.0	—	0.5	—	-0.5	—	—	—	—	—	3.2	—	3.0	—	—
	g/mL)	Percent	Mean	—	—	—	—	—	—	—	-18.5	—	-1.0	—	-3.8	—	—	—	—	—	8.9	—	8.1	—	—
		change (%)	Median	—	—	—	—	—	—	—	-17.0	—	1.8	—	-1.5	—	—	—	—	—	12.6	—	11.5	—	—
**Beverages (B)**	**Number of products**			**1**	**0**	**1**	**0**	**14**	**17**	**0**	**0**	**0**	**0**	**24**	**30**	**0**	**0**	**26**	**0**	**0**	**23**	**0**	**32**	**0**	**4**
	**HSR**	**Absolute**	**Mean**	**0.0**	—	**-0.5**	—	**0.1**	**0.0**	—	—	—	—	**0.0**	**0.0**	—	—	**0.1**	—	—	**0.0**	—	**0.0**	—	**0.0**
		**change**	**Median**	**0.0**	—	**-0.5**	—	**0.0**	**0.0**	—	—	—	—	**0.0**	**0.0**	—	—	**0.0**	—	—	**0.0**	—	**0.0**	—	**0.0**
		**Percent**	**Mean**	**0.0**	—	**-25.0**	—	**10.7**	**0.0**	—	—	—	—	**2.8**	**5.3**	—	—	**8.7**	—	—	**2.2**	—	**1.6**	—	**0.0**
		**change**	**Median**	**0.0**	—	**-25.0**	—	**0.0**	**0.0**	—	—	—	—	**0.0**	**0.0**	—	—	**0.0**	—	—	**0.0**	—	**0.0**	—	**0.0**
	**Calories**	**Absolute**	**Mean**	**0.0**	—	**-8.0**	—	**-0.3**	**-0.7**	—	—	—	—	**0.3**	**0.2**	—	—	**-1.4**	—	—	**-0.5**	—	**-0.3**	—	**0.0**
	**(kcal per**	**change**	**Median**	**0.0**	—	**-8.0**	—	**0.0**	**0.0**	—	—	—	—	**0.0**	**0.0**	—	—	**0.0**	—	—	**0.0**	—	**0.0**	—	**0.0**
	**100 g/mL)**	**Percent**	**Mean**	**0.0**	—	**-10.5**	—	**-0.4**	**-3.0**	—	—	—	—	**0.7**	**N/A**	—	—	**7.1**	—	—	**-0.4**	—	**3.5**	—	**0.0**
		**change**	**Median**	**0.0**	—	**-10.5**	—	**0.0**	**0.0**	—	—	—	—	**0.0**	**0.0**	—	—	**0.0**	—	—	**0.0**	—	**0.0**	—	**0.0**
	**Sodium**	**Absolute**	**Mean**	**2.0**	—	**0.0**	—	**-1.0**	**-1.1**	—	—	—	—	**-1.1**	**-0.3**	—	—	**0.8**	—	—	**0.4**	—	**0.6**	—	**0.0**
	**(mg per**	**change**	**Median**	**2.0**	—	**0.0**	—	**0.0**	**-1.4**	—	—	—	—	**0.0**	**-0.2**	—	—	**0.0**	—	—	**0.0**	—	**0.0**	—	**0.0**
	**100 g/mL)**	**Percent**	**Mean**	**100.0**	—	**0.0**	—	**-3.5**	**-12.3**	—	—	—	—	**-4.2**	**-3.6**	—	—	**N/A**	—	—	**0.3**	—	**-8.8**	—	**0.0**
		**change**	**Median**	**100.0**	—	**0.0**	—	**0.0**	**-11.1**	—	—	—	—	**0.0**	**-6.1**	—	—	**0.0**	—	—	**0.0**	—	**0.0**	—	**0.0**
	**Saturated**	**Absolute**	**Mean**	**0.0**	—	**0.0**	—	**0.0**	**0.0**	—	—	—	—	**-0.1**	**0.0**	—	—	**0.0**	—	—	**0.0**	—	**0.0**	—	**0.0**
	**fat (g per**	**change**	**Median**	**0.0**	—	**0.0**	—	**0.0**	**0.0**	—	—	—	—	**0.0**	**0.0**	—	—	**0.0**	—	—	**0.0**	—	**0.0**	—	**0.0**
	**100 g/mL)**	**Percent**	**Mean**	**N/A**	—	**0.0**	—	**N/A**	**N/A**	—	—	—	—	**-25.9**	**43.7**	—	—	**-2.8**	—	—	**N/A**	—	**0.0**	—	**N/A**
		**change**	**Median**	**N/A**	—	**0.0**	—	**N/A**	**N/A**	—	—	—	—	**-20.0**	**43.7**	—	—	**0.0**	—	—	**N/A**	—	**0.0**	—	**N/A**
	**Trans fat**	**Absolute**	**Mean**	**0.0**	—	**0.0**	—	**0.0**	**0.0**	—	—	—	—	**0.0**	**0.0**	—	—	**0.0**	—	—	**0.0**	—	**0.0**	—	**0.0**
	**(g per 100**	**change**	**Median**	**0.0**	—	**0.0**	—	**0.0**	**0.0**	—	—	—	—	**0.0**	**0.0**	—	—	**0.0**	—	—	**0.0**	—	**0.0**	—	**0.0**
	**g/mL)**	**Percent**	**Mean**	**N/A**	—	**-100.0**	—	**N/A**	**N/A**	—	—	—	—	**-58.6**	**N/A**	—	—	**N/A**	—	—	**N/A**	—	**N/A**	—	**N/A**
		**change**	**Median**	**N/A**	—	**-100.0**	—	**N/A**	**N/A**	—	—	—	—	**-74.7**	**N/A**	—	—	**N/A**	—	—	**N/A**	—	**N/A**	—	**N/A**
	**Total**	**Absolute**	**Mean**	**-0.4**	—	**-0.8**	—	**-0.2**	**-0.1**	—	—	—	—	**0.3**	**0.1**	—	—	**-0.3**	—	—	**-0.2**	—	**-0.1**	—	**0.0**
	**sugars (g**	**change**	**Median**	**-0.4**	—	**-0.8**	—	**0.0**	**0.0**	—	—	—	—	**0.0**	**0.0**	—	—	**0.0**	—	—	**0.0**	—	**0.0**	—	**0.0**
	**per 100**	**Percent**	**Mean**	**-4.5**	—	**-6.3**	—	**-2.8**	**-1.6**	—	—	—	—	**94.3**	**N/A**	—	—	**-5.8**	—	—	**-3.4**	—	**-1.5**	—	**0.0**
	**g/mL)**	**change**	**Median**	**-4.5**	—	**-6.3**	—	**0.0**	**0.0**	—	—	—	—	**36.5**	**0.0**	—	—	**0.0**	—	—	**0.0**	—	**0.0**	—	**0.0**
	**Free**	**Absolute**	**Mean**	**-0.4**	—	**-1.8**	—	**-0.2**	**-0.1**	—	—	—	—	**0.3**	**0.2**	—	—	**-0.3**	—	—	**-0.2**	—	**-0.1**	—	**0.0**
	**sugars (g**	**change**	**Median**	**-0.4**	—	**-1.8**	—	**0.0**	**0.0**	—	—	—	—	**0.0**	**0.0**	—	—	**0.0**	—	—	**0.0**	—	**0.0**	—	**0.0**
	**per 100**	**Percent**	**Mean**	**-4.5**	—	**-13.9**	—	**-2.8**	**-1.6**	—	—	—	—	**N/A**	**N/A**	—	—	**-6.2**	—	—	**-3.4**	—	**-0.9**	—	**0.0**
	**g/mL)**	**change**	**Median**	**-4.5**	—	**-13.9**	—	**0.0**	**0.0**	—	—	—	—	**0.0**	**0.0**	—	—	**-1.4**	—	—	**0.0**	—	**0.0**	—	**0.0**
Carbonated and	Number of products			1	0	1	0	14	17	0	0	0	0	20	27	0	0	16	0	0	23	0	30	0	1
non-carbonated	HSR	Absolute	Mean	0.0	—	-0.5	—	0.1	0.0	—	—	—	—	0.0	0.0	—	—	0.2	—	—	0.0	—	0.0	—	0.0
beverages (B1)		change	Median	0.0	—	-0.5	—	0.0	0.0	—	—	—	—	0.0	0.0	—	—	0.0	—	—	0.0	—	0.0	—	0.0
		Percent	Mean	0.0	—	-25.0	—	10.7	0.0	—	—	—	—	0.0	4.0	—	—	12.5	—	—	2.2	—	1.7	—	0.0
		change (%)	Median	0.0	—	-25.0	—	0.0	0.0	—	—	—	—	0.0	0.0	—	—	0.0	—	—	0.0	—	0.0	—	0.0
	Calories	Absolute	Mean	0.0	—	-8.0	—	-0.3	-0.7	—	—	—	—	0.0	-1.1	—	—	-2.5	—	—	-0.5	—	-0.1	—	0.0
	(kcal per	change	Median	0.0	—	-8.0	—	0.0	0.0	—	—	—	—	0.0	0.0	—	—	0.0	—	—	0.0	—	0.0	—	0.0
	100 g/mL)	Percent	Mean	0.0	—	-10.5	—	-0.4	-3.0	—	—	—	—	0.0	N/A	—	—	-11.4	—	—	-0.4	—	4.2	—	0.0
		change (%)	Median	0.0	—	-10.5	—	0.0	0.0	—	—	—	—	0.0	0.0	—	—	-13.3	—	—	0.0	—	0.0	—	0.0
	Sodium	Absolute	Mean	2.0	—	0.0	—	-1.0	-1.1	—	—	—	—	0.0	-0.4	—	—	-0.1	—	—	0.4	—	-0.3	—	0.0
	(mg per	change	Median	2.0	—	0.0	—	0.0	-1.4	—	—	—	—	0.0	-0.4	—	—	0.0	—	—	0.0	—	0.0	—	0.0
	100 g/mL)	Percent	Mean	100.0	—	0.0	—	-3.5	-12.3	—	—	—	—	0.0	-4.4	—	—	N/A	—	—	0.3	—	-11.9	—	0.0
		change (%)	Median	100.0	—	0.0	—	0.0	-11.1	—	—	—	—	0.0	-6.1	—	—	-0.3	—	—	0.0	—	0.0	—	0.0

				A. Lassonde	Agropur	Campbell Soup	Canada Bread	Canada Dry Mott's	Coca-Cola	Danone	General Mills	George Weston	Kellogg	Kraft Heinz	Loblaw	Maple Leaf Foods	Mondelēz	Nestlé	Ocean Spray	Parmalat	PepsiCo	Saputo	Sobeys	Sun-Rype	Unilever
	Saturated	Absolute	Mean	0.0	—	0.0	—	0.0	0.0	—	—	—	—	0.0	0.0	—	—	0.0	—	—	0.0	—	0.0	—	0.0
	fat (g per	change	Median	0.0	—	0.0	—	0.0	0.0	—	—	—	—	0.0	0.0	—	—	0.0	—	—	0.0	—	0.0	—	0.0
	100 g/mL)	Percent	Mean	N/A	—	0.0	—	N/A	N/A	—	—	—	—	N/A	N/A	—	—	-22.2	—	—	N/A	—	N/A	—	N/A
		change (%)	Median	N/A	—	0.0	—	N/A	N/A	—	—	—	—	N/A	N/A	—	—	-22.2	—	—	N/A	—	N/A	—	N/A
	Trans fat (g	Absolute	Mean	0.0	—	0.0	—	0.0	0.0	—	—	—	—	0.0	0.0	—	—	0.0	—	—	0.0	—	0.0	—	0.0
	per 100	change	Median	0.0	—	0.0	—	0.0	0.0	—	—	—	—	0.0	0.0	—	—	0.0	—	—	0.0	—	0.0	—	0.0
	g/mL)	Percent	Mean	N/A	—	-100.0	—	N/A	N/A	—	—	—	—	N/A	N/A	—	—	N/A	—	—	N/A	—	N/A	—	N/A
		change (%)	Median	N/A	—	-100.0	—	N/A	N/A	—	—	—	—	N/A	N/A	—	—	N/A	—	—	N/A	—	N/A	—	N/A
	Total	Absolute	Mean	-0.4	—	-0.8	—	-0.2	-0.1	—	—	—	—	0.0	0.0	—	—	-0.4	—	—	-0.2	—	-0.2	—	0.0
	sugars (g	change	Median	-0.4	—	-0.8	—	0.0	0.0	—	—	—	—	0.0	0.0	—	—	0.0	—	—	0.0	—	0.0	—	0.0
	per 100	Percent	Mean	-4.5	—	-6.3	—	-2.8	-1.6	—	—	—	—	0.0	N/A	—	—	-10.7	—	—	-3.4	—	-2.2	—	0.0
	g/mL)	change (%)	Median	-4.5	—	-6.3	—	0.0	0.0	—	—	—	—	0.0	0.0	—	—	-9.4	—	—	0.0	—	0.0	—	0.0
	Free sugars	Absolute	Mean	-0.4	—	-1.8	—	-0.2	-0.1	—	—	—	—	0.0	0.0	—	—	-0.4	—	—	-0.2	—	-0.2	—	0.0
	(g per 100	change	Median	-0.4	—	-1.8	—	0.0	0.0	—	—	—	—	0.0	0.0	—	—	0.0	—	—	0.0	—	0.0	—	0.0
	g/mL)	Percent	Mean	-4.5	—	-13.9	—	-2.8	-1.6	—	—	—	—	0.0	N/A	—	—	-10.7	—	—	-3.4	—	-2.2	—	0.0
		change (%)	Median	-4.5	—	-13.9	—	0.0	0.0	—	—	—	—	0.0	0.0	—	—	-9.4	—	—	0.0	—	0.0	—	0.0
Coffee, tea and	Number of products			0	0	0	0	0	0	0	0	0	0	4	3	0	0	10	0	0	0	0	2	0	3
hot chocolate	HSR	Absolute	Mean	—	—	—	—	—	—	—	—	—	—	0.3	0.0	—	—	0.1	—	—	—	—	0.0	—	0.0
		change	Median	—	—	—	—	—	—	—	—	—	—	0.3	0.0	—	—	0.0	—	—	—	—	0.0	—	0.0
		Percent	Mean	—	—	—	—	—	—	—	—	—	—	16.7	16.7	—	—	2.5	—	—	—	—	0.0	—	0.0
		change (%)	Median	—	—	—	—	—	—	—	—	—	—	16.7	0.0	—	—	0.0	—	—	—	—	0.0	—	0.0
	Calories	Absolute	Mean	—	—	—	—	—	—	—	—	—	—	1.8	11.7	—	—	0.4	—	—	—	—	-2.9	—	0.0
	(kcal per	change	Median	—	—	—	—	—	—	—	—	—	—	3.2	11.4	—	—	0.0	—	—	—	—	-2.9	—	0.0
	100 g/mL)	Percent	Mean	—	—	—	—	—	—	—	—	—	—	2.5	20.5	—	—	20.0	—	—	—	—	-4.2	—	N/A
		change (%)	Median	—	—	—	—	—	—	—	—	—	—	1.2	20.0	—	—	0.0	—	—	—	—	-4.2	—	N/A
	Sodium	Absolute	Mean	—	—	—	—	—	—	—	—	—	—	-6.8	0.9	—	—	2.3	—	—	—	—	12.9	—	0.0
	(mg per	change	Median	—	—	—	—	—	—	—	—	—	—	-3.5	0.1	—	—	0.0	—	—	—	—	12.9	—	0.0
	100 g/mL)	Percent	Mean	—	—	—	—	—	—	—	—	—	—	-13.6	3.2	—	—	29.1	—	—	—	—	37.5	—	N/A
		change (%)	Median	—	—	—	—	—	—	—	—	—	—	-8.3	1.9	—	—	0.0	—	—	—	—	37.5	—	N/A
	Saturated	Absolute	Mean	—	—	—	—	—	—	—	—	—	—	-0.3	0.3	—	—	0.0	—	—	—	—	0.0	—	0.0
	fat (g per	change	Median	—	—	—	—	—	—	—	—	—	—	-0.2	0.3	—	—	0.0	—	—	—	—	0.0	—	0.0
	100 g/mL)	Percent	Mean	—	—	—	—	—	—	—	—	—	—	-25.9	43.7	—	—	0.0	—	—	—	—	0.0	—	N/A
		change (%)	Median	—	—	—	—	—	—	—	—	—	—	-20.0	43.7	—	—	0.0	—	—	—	—	0.0	—	N/A
	Trans fat (g	Absolute	Mean	—	—	—	—	—	—	—	—	—	—	0.0	0.0	—	—	0.0	—	—	—	—	0.0	—	0.0
	per 100	change	Median	—	—	—	—	—	—	—	—	—	—	-0.1	0.0	—	—	0.0	—	—	—	—	0.0	—	0.0
	g/mL)	Percent	Mean	—	—	—	—	—	—	—	—	—	—	-58.6	N/A	—	—	N/A	—	—	—	—	N/A	—	N/A
		change (%)	Median	—	—	—	—	—	—	—	—	—	—	-74.7	N/A	—	—	N/A	—	—	—	—	N/A	—	N/A
	Total	Absolute	Mean	—	—	—	—	—	—	—	—	—	—	1.6	0.9	—	—	-0.1	—	—	—	—	0.6	—	0.0
	sugars (g	change	Median	—	—	—	—	—	—	—	—	—	—	1.3	1.0	—	—	0.0	—	—	—	—	0.6	—	0.0
	per 100	Percent	Mean	—	—	—	—	—	—	—	—	—	—	117.8	9.4	—	—	-1.6	—	—	—	—	5.3	—	N/A
	g/mL)	change (%)	Median	—	—	—	—	—	—	—	—	—	—	37.2	8.8	—	—	0.0	—	—	—	—	5.3	—	N/A
	Free sugars	Absolute	Mean	—	—	—	—	—	—	—	—	—	—	1.6	1.3	—	—	0.0	—	—	—	—	1.1	—	0.0
	(g per 100	change	Median	—	—	—	—	—	—	—	—	—	—	1.0	1.5	—	—	0.2	—	—	—	—	1.1	—	0.0
	g/mL)	Percent	Mean	—	—	—	—	—	—	—	—	—	—	N/A	14.8	—	—	-1.7	—	—	—	—	11.4	—	N/A
		change (%)	Median	—	—	—	—	—	—	—	—	—	—	4.4	15.2	—	—	5.3	—	—	—	—	11.4	—	N/A
**Cereals and**	**Number of products**			**0**	**0**	**0**	**0**	**0**	**0**	**0**	**18**	**1**	**30**	**4**	**102**	**0**	**0**	**0**	**0**	**0**	**31**	**0**	**30**	**0**	**7**
**other grain**	**HSR**	**Absolute**	**Mean**	—	—	—	—	—	—	—	**0.3**	**-1.0**	**0.1**	**0.3**	**0.0**	—	—	—	—	—	**0.0**	—	**0.0**	—	**0.0**
**products (C)**		**change**	**Median**	—	—	—	—	—	—	—	**0.0**	**-1.0**	**0.0**	**0.3**	**0.0**	—	—	—	—	—	**0.0**	—	**0.0**	—	**0.0**
		**Percent**	**Mean**	—	—	—	—	—	—	—	**11.9**	**-25.0**	**2.6**	**16.7**	**-0.1**	—	—	—	—	—	**1.9**	—	**1.1**	—	**0.0**
		**change**	**Median**	—	—	—	—	—	—	—	**0.0**	**-25.0**	**0.0**	**16.7**	**0.0**	—	—	—	—	—	**0.0**	—	**0.0**	—	**0.0**
	**Calories**	**Absolute**	**Mean**	—	—	—	—	—	—	—	**-8.1**	**-5.5**	**-4.7**	**8.3**	**-5.4**	—	—	—	—	—	**-15.0**	—	**0.3**	—	**-21.1**
	**(kcal per**	**change**	**Median**	—	—	—	—	—	—	—	**0.0**	**-5.5**	**0.0**	**0.0**	**0.0**	—	—	—	—	—	**-14.3**	—	**0.0**	—	**-28.9**
	**100 g/mL)**	**Percent**	**Mean**	—	—	—	—	—	—	—	**-1.9**	**-1.2**	**-1.3**	**2.3**	**-1.4**	—	—	—	—	—	**-3.8**	—	**0.1**	—	**-5.4**
		**change**	**Median**	—	—	—	—	—	—	—	**0.0**	**-1.2**	**0.0**	**0.0**	**0.0**	—	—	—	—	—	**-4.2**	—	**0.0**	—	**-7.3**
	**Sodium**	**Absolute**	**Mean**	—	—	—	—	—	—	—	**-33.2**	**-6.4**	**-17.7**	**-16.7**	**-1.7**	—	—	—	—	—	**-6.8**	—	**-5.4**	—	**-5.8**
	**(mg per**	**change**	**Median**	—	—	—	—	—	—	—	**-12.7**	**-6.4**	**0.0**	**-16.7**	**0.0**	—	—	—	—	—	**0.0**	—	**0.0**	—	**-24.4**
	**100 g/mL)**	**Percent**	**Mean**	—	—	—	—	—	—	—	**-5.0**	**-9.1**	**-2.9**	**-2.0**	**-5.0**	—	—	—	—	—	**-0.4**	—	**-1.7**	—	**-14.5**
		**change**	**Median**	—	—	—	—	—	—	—	**-4.0**	**-9.1**	**0.0**	**-2.4**	**0.0**	—	—	—	—	—	**0.0**	—	**0.0**	—	**-2.4**
	**Saturated**	**Absolute**	**Mean**	—	—	—	—	—	—	—	**0.1**	**0.7**	**0.0**	**-0.4**	**0.0**	—	—	—	—	—	**-0.1**	—	**0.0**	—	**-0.1**
	**fat (g per**	**change**	**Median**	—	—	—	—	—	—	—	**0.0**	**0.7**	**0.0**	**-0.5**	**0.0**	—	—	—	—	—	**0.0**	—	**0.0**	—	**0.0**
	**100 g/mL)**	**Percent**	**Mean**	—	—	—	—	—	—	—	**-4.8**	**36.4**	**5.7**	**-77.8**	**15.3**	—	—	—	—	—	**-10.2**	—	**0.0**	—	**-43.8**
		**change**	**Median**	—	—	—	—	—	—	—	**0.0**	**36.4**	**0.0**	**-100.0**	**0.0**	—	—	—	—	—	**0.0**	—	**0.0**	—	**-31.2**
	**Trans fat**	**Absolute**	**Mean**	—	—	—	—	—	—	—	**0.0**	**0.0**	**0.0**	**-0.4**	**0.0**	—	—	—	—	—	**0.0**	—	**0.0**	—	**0.0**
	**(g per 100**	**change**	**Median**	—	—	—	—	—	—	—	**0.0**	**0.0**	**0.0**	**-0.5**	**0.0**	—	—	—	—	—	**0.0**	—	**0.0**	—	**0.0**
	**g/mL)**	**Percent**	**Mean**	—	—	—	—	—	—	—	**-100.0**	**N/A**	**N/A**	**-83.3**	**-100.0**	—	—	—	—	—	**N/A**	—	**N/A**	—	**N/A**
		**change**	**Median**	—	—	—	—	—	—	—	**-100.0**	**N/A**	**N/A**	**-100.0**	**-100.0**	—	—	—	—	—	**N/A**	—	**N/A**	—	**N/A**
	**Total**	**Absolute**	**Mean**	—	—	—	—	—	—	—	**-0.7**	**8.9**	**-0.5**	**-1.7**	**0.4**	—	—	—	—	—	**-0.2**	—	**0.2**	—	**0.6**
	**sugars (g**	**change**	**Median**	—	—	—	—	—	—	—	**0.0**	**8.9**	**0.0**	**-1.7**	**0.0**	—	—	—	—	—	**0.0**	—	**0.0**	—	**0.0**
	**per 100**	**Percent**	**Mean**	—	—	—	—	—	—	—	**-1.9**	**40.5**	**-0.5**	**-22.2**	**1.7**	—	—	—	—	—	**-0.5**	—	**2.8**	—	**11.5**
	**g/mL)**	**change**	**Median**	—	—	—	—	—	—	—	**0.0**	**40.5**	**0.0**	**-33.3**	**0.0**	—	—	—	—	—	**0.0**	—	**0.0**	—	**0.0**
	**Free**	**Absolute**	**Mean**	—	—	—	—	—	—	—	**-0.4**	**9.5**	**-0.8**	**-1.3**	**0.3**	—	—	—	—	—	**-0.8**	—	**0.0**	—	**1.2**
	**sugars (g**	**change**	**Median**	—	—	—	—	—	—	—	**0.0**	**9.5**	**0.0**	**-1.5**	**0.0**	—	—	—	—	—	**0.0**	—	**0.0**	—	**0.8**
	**per 100**	**Percent**	**Mean**	—	—	—	—	—	—	—	**23.4**	**54.5**	**-2.0**	**-23.0**	**4.6**	—	—	—	—	—	**3.7**	—	**19.7**	—	**23.5**

				A. Lassonde	Agropur	Campbell Soup	Canada Bread	Canada Dry Mott's	Coca-Cola	Danone	General Mills	George Weston	Kellogg	Kraft Heinz	Loblaw	Maple Leaf Foods	Mondelēz	Nestlé	Ocean Spray	Parmalat	PepsiCo	Saputo	Sobeys	Sun-Rype	Unilever
	**g/mL)**	**change**	**Median**	—	—	—	—	—	—	—	**-0.4**	**54.5**	**0.0**	**-49.4**	**1.1**	—	—	—	—	—	**-7.0**	—	**0.0**	—	**23.5**
Breakfast	Number of products			0	0	0	0	0	0	0	17	1	30	0	32	0	0	0	0	0	28	0	9	0	0
cereals, hot and	HSR	Absolute	Mean	—	—	—	—	—	—	—	0.3	-1.0	0.1	—	-0.1	—	—	—	—	—	0.0	—	0.0	—	—
ready-to-eat		change	Median	—	—	—	—	—	—	—	0.0	-1.0	0.0	—	0.0	—	—	—	—	—	0.0	—	0.0	—	—
(C1, C2, C3,		Percent	Mean	—	—	—	—	—	—	—	12.6	-25.0	2.6	—	-2.3	—	—	—	—	—	2.1	—	0.0	—	—
C4)		change (%)	Median	—	—	—	—	—	—	—	0.0	-25.0	0.0	—	0.0	—	—	—	—	—	0.0	—	0.0	—	—
	Calories	Absolute	Mean	—	—	—	—	—	—	—	-8.6	-5.5	-4.7	—	-13.8	—	—	—	—	—	-16.1	—	2.0	—	—
	(kcal per	change	Median	—	—	—	—	—	—	—	0.0	-5.5	0.0	—	-9.1	—	—	—	—	—	-22.7	—	0.0	—	—
	100 g/mL)	Percent	Mean	—	—	—	—	—	—	—	-2.0	-1.2	-1.3	—	-3.5	—	—	—	—	—	-4.1	—	0.5	—	—
		change (%)	Median	—	—	—	—	—	—	—	0.0	-1.2	0.0	—	-1.9	—	—	—	—	—	-5.7	—	0.0	—	—
	Sodium	Absolute	Mean	—	—	—	—	—	—	—	-35.1	-6.4	-17.7	—	1.9	—	—	—	—	—	0.1	—	1.0	—	—
	(mg per	change	Median	—	—	—	—	—	—	—	-17.2	-6.4	0.0	—	0.0	—	—	—	—	—	0.0	—	0.0	—	—
	100 g/mL)	Percent	Mean	—	—	—	—	—	—	—	-5.3	-9.1	-2.9	—	1.2	—	—	—	—	—	0.0	—	1.1	—	—
		change (%)	Median	—	—	—	—	—	—	—	-4.3	-9.1	0.0	—	0.0	—	—	—	—	—	0.0	—	0.0	—	—
	Saturated	Absolute	Mean	—	—	—	—	—	—	—	0.1	0.7	0.0	—	-0.1	—	—	—	—	—	-0.1	—	0.0	—	—
	fat (g per	change	Median	—	—	—	—	—	—	—	0.0	0.7	0.0	—	0.0	—	—	—	—	—	0.0	—	0.0	—	—
	100 g/mL)	Percent	Mean	—	—	—	—	—	—	—	-5.1	36.4	5.7	—	-3.1	—	—	—	—	—	-7.7	—	0.0	—	—
		change (%)	Median	—	—	—	—	—	—	—	0.0	36.4	0.0	—	0.0	—	—	—	—	—	0.0	—	0.0	—	—
	Trans fat (g	Absolute	Mean	—	—	—	—	—	—	—	0.0	0.0	0.0	—	0.0	—	—	—	—	—	0.0	—	0.0	—	—
	per 100	change	Median	—	—	—	—	—	—	—	0.0	0.0	0.0	—	0.0	—	—	—	—	—	0.0	—	0.0	—	—
	g/mL)	Percent	Mean	—	—	—	—	—	—	—	-100.0	N/A	N/A	—	N/A	—	—	—	—	—	N/A	—	N/A	—	—
		change (%)	Median	—	—	—	—	—	—	—	-100.0	N/A	N/A	—	N/A	—	—	—	—	—	N/A	—	N/A	—	—
	Total	Absolute	Mean	—	—	—	—	—	—	—	-0.8	8.9	-0.5	—	1.4	—	—	—	—	—	-0.2	—	0.2	—	—
	sugars (g	change	Median	—	—	—	—	—	—	—	0.0	8.9	0.0	—	0.0	—	—	—	—	—	0.0	—	0.0	—	—
	per 100	Percent	Mean	—	—	—	—	—	—	—	-2.0	40.5	-0.5	—	3.3	—	—	—	—	—	-0.6	—	1.3	—	—
	g/mL)	change (%)	Median	—	—	—	—	—	—	—	0.0	40.5	0.0	—	0.0	—	—	—	—	—	0.0	—	0.0	—	—
	Free sugars	Absolute	Mean	—	—	—	—	—	—	—	-0.5	9.5	-0.8	—	0.9	—	—	—	—	—	-1.0	—	-0.5	—	—
	(g per 100	change	Median	—	—	—	—	—	—	—	0.0	9.5	0.0	—	0.1	—	—	—	—	—	-0.2	—	0.0	—	—
	g/mL)	Percent	Mean	—	—	—	—	—	—	—	23.4	54.5	-2.0	—	3.0	—	—	—	—	—	-5.8	—	-1.2	—	—
		change (%)	Median	—	—	—	—	—	—	—	-0.4	54.5	0.0	—	1.1	—	—	—	—	—	-7.5	—	-0.3	—	—
Grains (e.g.,	Number of products			0	0	0	0	0	0	0	1	0	0	0	22	0	0	0	0	0	2	0	7	0	6
rice, barley),	HSR	Absolute	Mean	—	—	—	—	—	—	—	0.0	—	—	—	0.0	—	—	—	—	—	0.0	—	0.0	—	0.0
including		change	Median	—	—	—	—	—	—	—	0.0	—	—	—	0.0	—	—	—	—	—	0.0	—	0.0	—	0.0
flavoured (C8)		Percent	Mean	—	—	—	—	—	—	—	0.0	—	—	—	0.1	—	—	—	—	—	0.0	—	0.0	—	0.0
		change (%)	Median	—	—	—	—	—	—	—	0.0	—	—	—	0.0	—	—	—	—	—	0.0	—	0.0	—	0.0
	Calories	Absolute	Mean	—	—	—	—	—	—	—	0.0	—	—	—	-4.9	—	—	—	—	—	-7.1	—	0.0	—	-12.7
	(kcal per	change	Median	—	—	—	—	—	—	—	0.0	—	—	—	0.0	—	—	—	—	—	-7.1	—	0.0	—	-14.4
	100 g/mL)	Percent	Mean	—	—	—	—	—	—	—	0.0	—	—	—	-1.5	—	—	—	—	—	-2.1	—	0.0	—	-3.0
		change (%)	Median	—	—	—	—	—	—	—	0.0	—	—	—	0.0	—	—	—	—	—	-2.1	—	0.0	—	-3.7
	Sodium	Absolute	Mean	—	—	—	—	—	—	—	0.0	—	—	—	-7.5	—	—	—	—	—	-107.1	—	0.0	—	-2.1
	(mg per	change	Median	—	—	—	—	—	—	—	0.0	—	—	—	0.0	—	—	—	—	—	-107.1	—	0.0	—	-11.5
	100 g/mL)	Percent	Mean	—	—	—	—	—	—	—	0.0	—	—	—	-13.7	—	—	—	—	—	-5.4	—	0.0	—	-0.3
		change (%)	Median	—	—	—	—	—	—	—	0.0	—	—	—	0.0	—	—	—	—	—	-5.4	—	0.0	—	-1.2
	Saturated	Absolute	Mean	—	—	—	—	—	—	—	0.0	—	—	—	0.1	—	—	—	—	—	-0.1	—	0.0	—	-0.2
	fat (g per	change	Median	—	—	—	—	—	—	—	0.0	—	—	—	0.0	—	—	—	—	—	-0.1	—	0.0	—	0.0
	100 g/mL)	Percent	Mean	—	—	—	—	—	—	—	0.0	—	—	—	29.1	—	—	—	—	—	-50.0	—	0.0	—	-43.8
		change (%)	Median	—	—	—	—	—	—	—	0.0	—	—	—	0.0	—	—	—	—	—	-50.0	—	0.0	—	-31.2
	Trans fat (g	Absolute	Mean	—	—	—	—	—	—	—	0.0	—	—	—	0.0	—	—	—	—	—	0.0	—	0.0	—	0.0
	per 100	change	Median	—	—	—	—	—	—	—	0.0	—	—	—	0.0	—	—	—	—	—	0.0	—	0.0	—	0.0
	g/mL)	Percent	Mean	—	—	—	—	—	—	—	N/A	—	—	—	N/A	—	—	—	—	—	N/A	—	N/A	—	N/A
		change (%)	Median	—	—	—	—	—	—	—	N/A	—	—	—	N/A	—	—	—	—	—	N/A	—	N/A	—	N/A
	Total	Absolute	Mean	—	—	—	—	—	—	—	0.0	—	—	—	0.1	—	—	—	—	—	0.0	—	0.0	—	0.7
	sugars (g	change	Median	—	—	—	—	—	—	—	0.0	—	—	—	0.0	—	—	—	—	—	0.0	—	0.0	—	0.0
	per 100	Percent	Mean	—	—	—	—	—	—	—	0.0	—	—	—	0.0	—	—	—	—	—	0.0	—	N/A	—	13.7
	g/mL)	change (%)	Median	—	—	—	—	—	—	—	0.0	—	—	—	0.0	—	—	—	—	—	0.0	—	N/A	—	0.0
	Free sugars	Absolute	Mean	—	—	—	—	—	—	—	0.0	—	—	—	0.0	—	—	—	—	—	2.0	—	0.0	—	1.1
	(g per 100	change	Median	—	—	—	—	—	—	—	0.0	—	—	—	0.0	—	—	—	—	—	2.0	—	0.0	—	0.8
	g/mL)	Percent	Mean	—	—	—	—	—	—	—	N/A	—	—	—	42.9	—	—	—	—	—	203.5	—	N/A	—	41.8
		change (%)	Median	—	—	—	—	—	—	—	N/A	—	—	—	42.9	—	—	—	—	—	203.5	—	N/A	—	41.8
Other (C5, C6,	Number of products			0	0	0	0	0	0	0	0	0	0	4	6	0	0	0	0	0	1	0	2	0	1
C10, C11)	HSR	Absolute	Mean	—	—	—	—	—	—	—	—	—	—	0.3	0.0	—	—	—	—	—	0.0	—	0.3	—	0.0
		change	Median	—	—	—	—	—	—	—	—	—	—	0.3	0.0	—	—	—	—	—	0.0	—	0.3	—	0.0
		Percent	Mean	—	—	—	—	—	—	—	—	—	—	16.7	0.0	—	—	—	—	—	0.0	—	16.7	—	0.0
		change (%)	Median	—	—	—	—	—	—	—	—	—	—	16.7	0.0	—	—	—	—	—	0.0	—	16.7	—	0.0
	Calories	Absolute	Mean	—	—	—	—	—	—	—	—	—	—	8.3	0.0	—	—	—	—	—	0.0	—	0.0	—	-71.4
	(kcal per	change	Median	—	—	—	—	—	—	—	—	—	—	0.0	0.0	—	—	—	—	—	0.0	—	0.0	—	-71.4
	100 g/mL)	Percent	Mean	—	—	—	—	—	—	—	—	—	—	2.3	0.0	—	—	—	—	—	0.0	—	0.0	—	-20.0
		change (%)	Median	—	—	—	—	—	—	—	—	—	—	0.0	0.0	—	—	—	—	—	0.0	—	0.0	—	-20.0
	Sodium	Absolute	Mean	—	—	—	—	—	—	—	—	—	—	-16.7	0.0	—	—	—	—	—	0.0	—	-83.3	—	-28.6
	(mg per	change	Median	—	—	—	—	—	—	—	—	—	—	-16.7	0.0	—	—	—	—	—	0.0	—	-83.3	—	-28.6
	100 g/mL)	Percent	Mean	—	—	—	—	—	—	—	—	—	—	-2.0	0.0	—	—	—	—	—	0.0	—	-5.3	—	-100.0
		change (%)	Median	—	—	—	—	—	—	—	—	—	—	-2.4	0.0	—	—	—	—	—	0.0	—	-5.3	—	-100.0
	Saturated	Absolute	Mean	—	—	—	—	—	—	—	—	—	—	-0.4	0.0	—	—	—	—	—	0.0	—	0.7	—	0.0

				A. Lassonde	Agropur	Campbell Soup	Canada Bread	Canada Dry Mott's	Coca-Cola	Danone	General Mills	George Weston	Kellogg	Kraft Heinz	Loblaw	Maple Leaf Foods	Mondelēz	Nestlé	Ocean Spray	Parmalat	PepsiCo	Saputo	Sobeys	Sun-Rype	Unilever
	fat (g per	change	Median	—	—	—	—	—	—	—	—	—	—	-0.5	0.0	—	—	—	—	—	0.0	—	0.7	—	0.0
	100 g/mL)	Percent	Mean	—	—	—	—	—	—	—	—	—	—	-77.8	0.0	—	—	—	—	—	0.0	—	N/A	—	N/A
		change (%)	Median	—	—	—	—	—	—	—	—	—	—	-100.0	0.0	—	—	—	—	—	0.0	—	N/A	—	N/A
	Trans fat (g	Absolute	Mean	—	—	—	—	—	—	—	—	—	—	-0.4	0.0	—	—	—	—	—	0.0	—	0.0	—	0.0
	per 100	change	Median	—	—	—	—	—	—	—	—	—	—	-0.5	0.0	—	—	—	—	—	0.0	—	0.0	—	0.0
	g/mL)	Percent	Mean	—	—	—	—	—	—	—	—	—	—	-83.3	N/A	—	—	—	—	—	N/A	—	N/A	—	N/A
		change (%)	Median	—	—	—	—	—	—	—	—	—	—	-100.0	N/A	—	—	—	—	—	N/A	—	N/A	—	N/A
	Total	Absolute	Mean	—	—	—	—	—	—	—	—	—	—	-1.7	0.0	—	—	—	—	—	0.0	—	1.7	—	0.0
	sugars (g	change	Median	—	—	—	—	—	—	—	—	—	—	-1.7	0.0	—	—	—	—	—	0.0	—	1.7	—	0.0
	per 100	Percent	Mean	—	—	—	—	—	—	—	—	—	—	-22.2	N/A	—	—	—	—	—	0.0	—	25.0	—	0.0
	g/mL)	change (%)	Median	—	—	—	—	—	—	—	—	—	—	-33.3	N/A	—	—	—	—	—	0.0	—	25.0	—	0.0
	Free sugars	Absolute	Mean	—	—	—	—	—	—	—	—	—	—	-1.3	0.0	—	—	—	—	—	0.0	—	2.2	—	1.4
	(g per 100	change	Median	—	—	—	—	—	—	—	—	—	—	-1.5	0.0	—	—	—	—	—	0.0	—	2.2	—	1.4
	g/mL)	Percent	Mean	—	—	—	—	—	—	—	—	—	—	-23.0	N/A	—	—	—	—	—	N/A	—	92.7	—	5.3
		change (%)	Median	—	—	—	—	—	—	—	—	—	—	-49.4	N/A	—	—	—	—	—	N/A	—	92.7	—	5.3
Pastas,	Number of products			0	0	0	0	0	0	0	0	0	0	0	42	0	0	0	0	0	0	0	12	0	0
including filled	HSR	Absolute	Mean	—	—	—	—	—	—	—	—	—	—	—	0.1	—	—	—	—	—	—	—	0.0	—	—
pastas, without		change	Median	—	—	—	—	—	—	—	—	—	—	—	0.0	—	—	—	—	—	—	—	0.0	—	—
sauce (C8)		Percent	Mean	—	—	—	—	—	—	—	—	—	—	—	1.5	—	—	—	—	—	—	—	0.0	—	—
		change (%)	Median	—	—	—	—	—	—	—	—	—	—	—	0.0	—	—	—	—	—	—	—	0.0	—	—
	Calories	Absolute	Mean	—	—	—	—	—	—	—	—	—	—	—	0.1	—	—	—	—	—	—	—	-0.7	—	—
	(kcal per	change	Median	—	—	—	—	—	—	—	—	—	—	—	0.0	—	—	—	—	—	—	—	0.0	—	—
	100 g/mL)	Percent	Mean	—	—	—	—	—	—	—	—	—	—	—	0.1	—	—	—	—	—	—	—	-0.3	—	—
		change (%)	Median	—	—	—	—	—	—	—	—	—	—	—	0.0	—	—	—	—	—	—	—	0.0	—	—
	Sodium	Absolute	Mean	—	—	—	—	—	—	—	—	—	—	—	-1.8	—	—	—	—	—	—	—	-0.4	—	—
	(mg per	change	Median	—	—	—	—	—	—	—	—	—	—	—	0.0	—	—	—	—	—	—	—	0.0	—	—
	100 g/mL)	Percent	Mean	—	—	—	—	—	—	—	—	—	—	—	-8.8	—	—	—	—	—	—	—	-10.0	—	—
		change (%)	Median	—	—	—	—	—	—	—	—	—	—	—	0.0	—	—	—	—	—	—	—	-10.0	—	—
	Saturated	Absolute	Mean	—	—	—	—	—	—	—	—	—	—	—	0.0	—	—	—	—	—	—	—	0.0	—	—
	fat (g per	change	Median	—	—	—	—	—	—	—	—	—	—	—	0.0	—	—	—	—	—	—	—	0.0	—	—
	100 g/mL)	Percent	Mean	—	—	—	—	—	—	—	—	—	—	—	27.6	—	—	—	—	—	—	—	0.0	—	—
		change (%)	Median	—	—	—	—	—	—	—	—	—	—	—	0.0	—	—	—	—	—	—	—	0.0	—	—
	Trans fat (g	Absolute	Mean	—	—	—	—	—	—	—	—	—	—	—	0.0	—	—	—	—	—	—	—	0.0	—	—
	per 100	change	Median	—	—	—	—	—	—	—	—	—	—	—	0.0	—	—	—	—	—	—	—	0.0	—	—
	g/mL)	Percent	Mean	—	—	—	—	—	—	—	—	—	—	—	-100.0	—	—	—	—	—	—	—	N/A	—	—
		change (%)	Median	—	—	—	—	—	—	—	—	—	—	—	-100.0	—	—	—	—	—	—	—	N/A	—	—
	Total	Absolute	Mean	—	—	—	—	—	—	—	—	—	—	—	0.0	—	—	—	—	—	—	—	0.0	—	—
	sugars (g	change	Median	—	—	—	—	—	—	—	—	—	—	—	0.0	—	—	—	—	—	—	—	0.0	—	—
	per 100	Percent	Mean	—	—	—	—	—	—	—	—	—	—	—	1.0	—	—	—	—	—	—	—	0.0	—	—
	g/mL)	change (%)	Median	—	—	—	—	—	—	—	—	—	—	—	0.0	—	—	—	—	—	—	—	0.0	—	—
	Free sugars	Absolute	Mean	—	—	—	—	—	—	—	—	—	—	—	0.0	—	—	—	—	—	—	—	0.0	—	—
	(g per 100	change	Median	—	—	—	—	—	—	—	—	—	—	—	0.0	—	—	—	—	—	—	—	0.0	—	—
	g/mL)	Percent	Mean	—	—	—	—	—	—	—	—	—	—	—	N/A	—	—	—	—	—	—	—	N/A	—	—
		change (%)	Median	—	—	—	—	—	—	—	—	—	—	—	N/A	—	—	—	—	—	—	—	N/A	—	—
**Dairy**	**Number of products**			**0**	**52**	**1**	**0**	**0**	**0**	**37**	**48**	**0**	**3**	**56**	**107**	**0**	**0**	**13**	**0**	**33**	**0**	**27**	**43**	**0**	**0**
**products (D)**	**HSR**	**Absolute**	**Mean**	—	**-0.2**	**0.0**	—	—	—	**0.0**	**-0.1**	—	**0.0**	**-0.2**	**0.1**	—	—	**0.1**	—	**0.1**	—	**-0.3**	**0.0**	—	—
		**change**	**Median**	—	**0.0**	**0.0**	—	—	—	**0.0**	**0.0**	—	**0.0**	**0.0**	**0.0**	—	—	**0.0**	—	**0.0**	—	**0.0**	**0.0**	—	—
		**Percent**	**Mean**	—	**9.1**	**0.0**	—	—	—	**0.3**	**-6.9**	—	**0.0**	**-5.1**	**15.6**	—	—	**11.9**	—	**27.8**	—	**-15.9**	**11.9**	—	—
		**change**	**Median**	—	**0.0**	**0.0**	—	—	—	**0.0**	**0.0**	—	**0.0**	**0.0**	**0.0**	—	—	**0.0**	—	**0.0**	—	**0.0**	**0.0**	—	—
	**Calories**	**Absolute**	**Mean**	—	**-5.3**	**0.0**	—	—	—	**1.2**	**-2.3**	—	**0.0**	**-4.3**	**-7.2**	—	—	**-6.1**	—	**3.0**	—	**0.1**	**2.9**	—	—
	**(kcal per**	**change**	**Median**	—	**0.0**	**0.0**	—	—	—	**0.0**	**0.0**	—	**0.0**	**0.0**	**0.0**	—	—	**0.0**	—	**0.0**	—	**0.0**	**0.0**	—	—
	**100 g/mL)**	**Percent**	**Mean**	—	**-2.2**	**0.0**	—	—	—	**2.5**	**-1.5**	—	**0.0**	**-1.7**	**-2.3**	—	—	**-7.6**	—	**2.9**	—	**-0.9**	**1.6**	—	—
		**change**	**Median**	—	**0.0**	**0.0**	—	—	—	**0.0**	**0.0**	—	**0.0**	**0.0**	**0.0**	—	—	**0.0**	—	**0.0**	—	**0.0**	**0.0**	—	—
	**Sodium**	**Absolute**	**Mean**	—	**45.3**	**0.0**	—	—	—	**6.5**	**-0.5**	—	**2.3**	**-2.0**	**-2.5**	—	—	**-54.4**	—	**7.1**	—	**-1.9**	**-17.2**	—	—
	**(mg per**	**change**	**Median**	—	**0.0**	**0.0**	—	—	—	**8.6**	**0.0**	—	**3.4**	**0.0**	**0.0**	—	—	**-6.0**	—	**0.0**	—	**0.0**	**0.0**	—	—
	**100 g/mL)**	**Percent**	**Mean**	—	**3.6**	**0.0**	—	—	—	**15.2**	**-1.2**	—	**3.1**	**-0.1**	**0.0**	—	—	**-34.7**	—	**2.6**	—	**-0.9**	**N/A**	—	—
		**change**	**Median**	—	**0.0**	**0.0**	—	—	—	**18.8**	**0.0**	—	**4.5**	**0.0**	**0.0**	—	—	**-10.7**	—	**0.0**	—	**0.0**	**0.0**	—	—
	**Saturated**	**Absolute**	**Mean**	—	**0.7**	**0.0**	—	—	—	**0.0**	**-0.1**	—	**0.0**	**-0.3**	**-0.1**	—	—	**-2.3**	—	**0.2**	—	**0.2**	**0.1**	—	—
	**fat (g per**	**change**	**Median**	—	**0.0**	**0.0**	—	—	—	**0.0**	**0.0**	—	**0.0**	**0.0**	**0.0**	—	—	**-0.7**	—	**0.0**	—	**0.0**	**0.0**	—	—
	**100 g/mL)**	**Percent**	**Mean**	—	**N/A**	**0.0**	—	—	—	**-0.8**	**N/A**	—	**0.0**	**-2.1**	**0.5**	—	—	**-38.8**	—	**0.5**	—	**3.7**	**-1.0**	—	—
		**change**	**Median**	—	**0.0**	**0.0**	—	—	—	**0.0**	**0.0**	—	**0.0**	**0.0**	**0.0**	—	—	**-33.3**	—	**0.0**	—	**0.0**	**0.0**	—	—
	**Trans fat**	**Absolute**	**Mean**	—	**0.0**	**0.0**	—	—	—	**0.0**	**0.0**	—	**0.0**	**0.0**	**0.0**	—	—	**-0.5**	—	**0.1**	—	**0.0**	**0.0**	—	—
	**(g per 100**	**change**	**Median**	—	**0.0**	**0.0**	—	—	—	**0.0**	**0.0**	—	**0.0**	**0.0**	**0.0**	—	—	**0.0**	—	**0.0**	—	**0.0**	**0.0**	—	—
	**g/mL)**	**Percent**	**Mean**	—	**23.7**	**0.0**	—	—	—	**N/A**	**N/A**	—	**N/A**	**-1.6**	**4.8**	—	—	**N/A**	—	**19.8**	—	**9.0**	**N/A**	—	—
		**change**	**Median**	—	**0.0**	**0.0**	—	—	—	**N/A**	**0.0**	—	**N/A**	**0.0**	**0.0**	—	—	**-100.0**	—	**0.0**	—	**0.0**	**0.0**	—	—
	**Total**	**Absolute**	**Mean**	—	**-0.4**	**0.0**	—	—	—	**0.0**	**-0.3**	—	**-0.1**	**0.1**	**0.1**	—	—	**-0.8**	—	**0.3**	—	**0.1**	**0.7**	—	—
	**sugars (g**	**change**	**Median**	—	**0.0**	**0.0**	—	—	—	**0.0**	**0.0**	—	**0.0**	**0.0**	**0.0**	—	—	**0.0**	—	**0.0**	—	**0.0**	**0.0**	—	—
	**per 100**	**Percent**	**Mean**	—	**-10.7**	**0.0**	—	—	—	**3.7**	**-2.3**	—	**-1.8**	**1.9**	**N/A**	—	—	**-7.3**	—	**11.4**	—	**N/A**	**N/A**	—	—
	**g/mL)**	**change**	**Median**	—	**0.0**	**0.0**	—	—	—	**0.0**	**0.0**	—	**0.0**	**0.0**	**0.0**	—	—	**0.0**	—	**0.0**	—	**0.0**	**0.0**	—	—
	**Free**	**Absolute**	**Mean**	—	**-0.3**	**3.1**	—	—	—	**0.0**	**-0.3**	—	**3.1**	**-0.1**	**-0.4**	—	—	**0.1**	—	**-0.4**	—	**0.1**	**0.1**	—	—
	**sugars (g**	**change**	**Median**	—	**0.0**	**3.1**	—	—	—	**-0.3**	**0.0**	—	**3.2**	**0.0**	**0.0**	—	—	**0.0**	—	**0.0**	—	**0.0**	**0.0**	—	—
	**per 100**	**Percent**	**Mean**	—	**-20.2**	**39.2**	—	—	—	**N/A**	**-6.7**	—	**98.6**	**-16.7**	**-32.9**	—	—	**-2.0**	—	**N/A**	—	**-10.5**	**4.6**	—	—
	**g/mL)**	**change**	**Median**	—	**-10.2**	**39.2**	—	—	—	**-2.7**	**-9.0**	—	**102.1**	**-3.6**	**-33.3**	—	—	**0.0**	—	**-2.1**	—	**0.0**	**-42.3**	—	—

Category	Nutrient	Measure	Statistic	A. Lassonde	Agropur	Campbell Soup	Canada Bread	Canada Dry Mott's	Coca-Cola	Danone	General Mills	George Weston	Kellogg	Kraft Heinz	Loblaw	Maple Leaf Foods	Mondelēz	Nestlé	Ocean Spray	Parmalat	PepsiCo	Saputo	Sobeys	Sun-Rype	Unilever
Cheese (D1, D2, D3, D4, D5)	Number of products			0	21	0	0	0	0	0	5	0	0	51	81	0	0	0	0	17	0	7	34	0	0
	HSR	Absolute change	Mean	—	-0.4	—	—	—	—	—	-0.5	—	—	-0.2	0.0	—	—	—	—	0.2	—	-0.4	0.0	—	—
			Median	—	0.0	—	—	—	—	—	0.0	—	—	0.0	0.0	—	—	—	—	0.0	—	0.0	0.0	—	—
		Percent change (%)	Mean	—	24.1	—	—	—	—	—	-28.3	—	—	-4.1	18.4	—	—	—	—	54.8	—	-12.9	9.6	—	—
			Median	—	0.0	—	—	—	—	—	0.0	—	—	0.0	0.0	—	—	—	—	0.0	—	0.0	0.0	—	—
	Calories (kcal per 100 g/mL)	Absolute change	Mean	—	-9.5	—	—	—	—	—	0.0	—	—	-4.5	-4.9	—	—	—	—	5.9	—	4.8	5.6	—	—
			Median	—	0.0	—	—	—	—	—	0.0	—	—	0.0	0.0	—	—	—	—	0.0	—	0.0	0.0	—	—
		Percent change (%)	Mean	—	-2.4	—	—	—	—	—	0.0	—	—	-1.6	-1.1	—	—	—	—	1.7	—	1.2	1.8	—	—
			Median	—	0.0	—	—	—	—	—	0.0	—	—	0.0	0.0	—	—	—	—	0.0	—	0.0	0.0	—	—
	Sodium (mg per 100 g/mL)	Absolute change	Mean	—	115.9	—	—	—	—	—	0.0	—	—	-2.2	-0.6	—	—	—	—	12.4	—	-4.8	-10.1	—	—
			Median	—	0.0	—	—	—	—	—	0.0	—	—	0.0	0.0	—	—	—	—	0.0	—	0.0	0.0	—	—
		Percent change (%)	Mean	—	17.5	—	—	—	—	—	0.0	—	—	-0.1	2.2	—	—	—	—	2.0	—	0.3	-1.1	—	—
			Median	—	0.0	—	—	—	—	—	0.0	—	—	0.0	0.0	—	—	—	—	0.0	—	0.0	0.0	—	—
	Saturated fat (g per 100 g/mL)	Absolute change	Mean	—	1.8	—	—	—	—	—	0.0	—	—	-0.3	-0.1	—	—	—	—	0.5	—	0.5	0.2	—	—
			Median	—	0.0	—	—	—	—	—	0.0	—	—	0.0	0.0	—	—	—	—	0.0	—	0.0	0.0	—	—
		Percent change (%)	Mean	—	25.2	—	—	—	—	—	0.0	—	—	-2.3	-0.7	—	—	—	—	5.9	—	2.8	1.1	—	—
			Median	—	0.0	—	—	—	—	—	0.0	—	—	0.0	0.0	—	—	—	—	0.0	—	0.0	0.0	—	—
	Trans fat (g per 100 g/mL)	Absolute change	Mean	—	0.1	—	—	—	—	—	0.1	—	—	0.0	0.0	—	—	—	—	0.1	—	0.1	0.0	—	—
			Median	—	0.0	—	—	—	—	—	0.0	—	—	0.0	0.0	—	—	—	—	0.0	—	0.0	0.0	—	—
		Percent change (%)	Mean	—	47.8	—	—	—	—	—	0.0	—	—	-1.6	6.3	—	—	—	—	31.7	—	19.0	1.1	—	—
			Median	—	50.0	—	—	—	—	—	0.0	—	—	0.0	0.0	—	—	—	—	0.0	—	0.0	0.0	—	—
	Total sugars (g per 100 g/mL)	Absolute change	Mean	—	-0.3	—	—	—	—	—	0.0	—	—	0.1	0.1	—	—	—	—	-0.1	—	0.0	0.7	—	—
			Median	—	0.0	—	—	—	—	—	0.0	—	—	0.0	0.0	—	—	—	—	0.0	—	0.0	0.0	—	—
		Percent change (%)	Mean	—	-33.3	—	—	—	—	—	0.0	—	—	3.6	2.9	—	—	—	—	-3.2	—	N/A	26.2	—	—
			Median	—	0.0	—	—	—	—	—	0.0	—	—	0.0	0.0	—	—	—	—	10.5	—	N/A	0.0	—	—
	Free sugars (g per 100 g/mL)	Absolute change	Mean	—	0.0	—	—	—	—	—	0.0	—	—	0.0	-0.4	—	—	—	—	-1.4	—	0.0	0.3	—	—
			Median	—	0.0	—	—	—	—	—	0.0	—	—	0.0	0.0	—	—	—	—	0.0	—	0.0	0.0	—	—
		Percent change (%)	Mean	—	-20.5	—	—	—	—	—	N/A	—	—	-25.2	-92.5	—	—	—	—	-89.8	—	N/A	29.7	—	—
			Median	—	-20.5	—	—	—	—	—	N/A	—	—	-9.5	-100.0	—	—	—	—	-100.0	—	N/A	-83.8	—	—
Cream and cream substitutes (D6, D7, D8, D14)	Number of products			0	2	0	0	0	0	0	3	0	0	5	8	0	0	7	0	0	0	5	5	0	0
	HSR	Absolute change	Mean	—	-1.5	—	—	—	—	—	-0.2	—	—	-0.3	-0.3	—	—	-1.0	—	—	—	-1.5	-0.4	—	—
			Median	—	-1.5	—	—	—	—	—	0.0	—	—	0.0	0.0	—	—	-1.0	—	—	—	-1.5	0.0	—	—
		Percent change (%)	Mean	—	-73.3	—	—	—	—	—	-16.7	—	—	-15.3	-13.8	—	—	-51.4	—	—	—	-73.7	-13.3	—	—
			Median	—	-73.3	—	—	—	—	—	0.0	—	—	0.0	0.0	—	—	-66.7	—	—	—	-75.0	0.0	—	—
	Calories (kcal per 100 g/mL)	Absolute change	Mean	—	0.0	—	—	—	—	—	-22.2	—	—	-2.3	-27.2	—	—	0.0	—	—	—	0.0	-18.6	—	—
			Median	—	0.0	—	—	—	—	—	0.0	—	—	0.0	0.0	—	—	0.0	—	—	—	0.0	-11.1	—	—
		Percent change (%)	Mean	—	0.0	—	—	—	—	—	-5.6	—	—	-2.7	-4.2	—	—	0.0	—	—	—	0.0	-7.5	—	—
			Median	—	0.0	—	—	—	—	—	0.0	—	—	0.0	0.0	—	—	0.0	—	—	—	0.0	-10.0	—	—
	Sodium (mg per 100 g/mL)	Absolute change	Mean	—	0.0	—	—	—	—	—	0.0	—	—	0.0	-14.1	—	—	-94.3	—	—	—	0.0	-19.3	—	—
			Median	—	0.0	—	—	—	—	—	0.0	—	—	0.0	0.0	—	—	-113.3	—	—	—	0.0	-11.1	—	—
		Percent change (%)	Mean	—	0.0	—	—	—	—	—	0.0	—	—	0.0	-7.3	—	—	-50.5	—	—	—	0.0	N/A	—	—
			Median	—	0.0	—	—	—	—	—	0.0	—	—	0.0	0.0	—	—	-57.1	—	—	—	0.0	-28.6	—	—
	Saturated fat (g per 100 g/mL)	Absolute change	Mean	—	0.0	—	—	—	—	—	-1.1	—	—	0.0	-0.2	—	—	-3.9	—	—	—	0.0	-0.9	—	—
			Median	—	0.0	—	—	—	—	—	0.0	—	—	0.0	0.0	—	—	-0.7	—	—	—	0.0	0.0	—	—
		Percent change (%)	Mean	—	0.0	—	—	—	—	—	-4.2	—	—	0.2	1.3	—	—	-46.7	—	—	—	0.0	-6.6	—	—
			Median	—	0.0	—	—	—	—	—	0.0	—	—	0.0	0.0	—	—	-33.3	—	—	—	0.0	0.0	—	—
	Trans fat (g per 100 g/mL)	Absolute change	Mean	—	0.0	—	—	—	—	—	0.1	—	—	0.0	0.0	—	—	-1.0	—	—	—	0.0	0.1	—	—
			Median	—	0.0	—	—	—	—	—	0.0	—	—	0.0	0.0	—	—	0.0	—	—	—	0.0	0.0	—	—
		Percent change (%)	Mean	—	N/A	—	—	—	—	—	N/A	—	—	0.0	0.0	—	—	-100.0	—	—	—	0.0	N/A	—	—
			Median	—	N/A	—	—	—	—	—	N/A	—	—	0.0	0.0	—	—	-100.0	—	—	—	0.0	N/A	—	—
	Total sugars (g per 100 g/mL)	Absolute change	Mean	—	0.0	—	—	—	—	—	0.0	—	—	-0.5	0.0	—	—	0.0	—	—	—	1.3	0.0	—	—
			Median	—	0.0	—	—	—	—	—	0.0	—	—	0.0	0.0	—	—	0.0	—	—	—	0.0	0.0	—	—
		Percent change (%)	Mean	—	0.0	—	—	—	—	—	0.0	—	—	-7.3	0.0	—	—	0.0	—	—	—	N/A	0.0	—	—
			Median	—	0.0	—	—	—	—	—	0.0	—	—	0.0	0.0	—	—	0.0	—	—	—	0.0	0.0	—	—
	Free sugars (g per 100 g/mL)	Absolute change	Mean	—	0.0	—	—	—	—	—	0.0	—	—	-0.6	-0.3	—	—	0.2	—	—	—	-1.3	-0.7	—	—
			Median	—	0.0	—	—	—	—	—	0.0	—	—	0.0	0.0	—	—	0.0	—	—	—	0.0	0.0	—	—
		Percent change (%)	Mean	—	N/A	—	—	—	—	—	N/A	—	—	-8.2	-16.2	—	—	0.5	—	—	—	-50.0	-42.3	—	—
			Median	—	N/A	—	—	—	—	—	N/A	—	—	0.0	-16.8	—	—	0.0	—	—	—	-50.0	-42.3	—	—
Milk and milk alternatives (D10, D11)	Number of products			0	17	0	0	0	0	0	2	0	0	0	11	0	0	3	0	0	0	11	4	0	0
	HSR	Absolute change	Mean	—	0.0	—	—	—	—	—	0.0	—	—	—	0.7	—	—	2.5	—	—	—	0.1	0.6	—	—
			Median	—	0.0	—	—	—	—	—	0.0	—	—	—	0.0	—	—	2.5	—	—	—	0.0	0.0	—	—
		Percent change (%)	Mean	—	-0.5	—	—	—	—	—	0.0	—	—	—	24.8	—	—	166.7	—	—	—	2.6	62.5	—	—
			Median	—	0.0	—	—	—	—	—	0.0	—	—	—	0.0	—	—	166.7	—	—	—	0.0	0.0	—	—
	Calories (kcal per 100 g/mL)	Absolute change	Mean	—	1.2	—	—	—	—	—	0.0	—	—	—	-9.1	—	—	-23.4	—	—	—	-0.4	7.3	—	—
			Median	—	0.0	—	—	—	—	—	0.0	—	—	—	0.0	—	—	-30.0	—	—	—	0.0	0.0	—	—
		Percent change (%)	Mean	—	2.9	—	—	—	—	—	0.0	—	—	—	-5.3	—	—	-29.4	—	—	—	-0.5	11.3	—	—
			Median	—	0.0	—	—	—	—	—	0.0	—	—	—	0.0	—	—	-37.5	—	—	—	0.0	0.0	—	—
	Sodium (mg per 100 g/mL)	Absolute change	Mean	—	-1.9	—	—	—	—	—	0.0	—	—	—	-1.2	—	—	-18.7	—	—	—	-1.3	-75.0	—	—
			Median	—	0.0	—	—	—	—	—	0.0	—	—	—	0.0	—	—	-6.0	—	—	—	0.0	-100.0	—	—
		Percent change (%)	Mean	—	-4.9	—	—	—	—	—	0.0	—	—	—	4.2	—	—	-38.1	—	—	—	-1.7	-40.0	—	—
			Median	—	0.0	—	—	—	—	—	0.0	—	—	—	0.0	—	—	-10.7	—	—	—	0.0	-50.0	—	—
	Saturated fat (g per	Absolute change	Mean	—	0.0	—	—	—	—	—	0.0	—	—	—	0.3	—	—	-0.7	—	—	—	0.0	-0.2	—	—
			Median	—	0.0	—	—	—	—	—	0.0	—	—	—	0.0	—	—	-0.9	—	—	—	0.0	0.0	—	—

				A. Lassonde	Agropur	Campbell Soup	Canada Bread	Canada Dry Mott's	Coca-Cola	Danone	General Mills	George Weston	Kellogg	Kraft Heinz	Loblaw	Maple Leaf Foods	Mondelēz	Nestlé	Ocean Spray	Parmalat	PepsiCo	Saputo	Sobeys	Sun-Rype	Unilever
	100 g/mL)	Percent	Mean	—	0.0	—	—	—	—	—	0.0	—	—	—	10.0	—	—	-48.2	—	—	—	-0.8	-16.1	—	—
		change (%)	Median	—	0.0	—	—	—	—	—	0.0	—	—	—	0.0	—	—	-53.1	—	—	—	0.0	0.0	—	—
	Trans fat (g	Absolute	Mean	—	0.0	—	—	—	—	—	0.0	—	—	—	-0.1	—	—	0.0	—	—	—	0.0	0.0	—	—
	per 100	change	Median	—	0.0	—	—	—	—	—	0.0	—	—	—	0.0	—	—	0.0	—	—	—	0.0	0.0	—	—
	g/mL)	Percent	Mean	—	-12.5	—	—	—	—	—	N/A	—	—	—	-100.0	—	—	N/A	—	—	—	0.0	N/A	—	—
		change (%)	Median	—	0.0	—	—	—	—	—	N/A	—	—	—	-100.0	—	—	N/A	—	—	—	0.0	N/A	—	—
	Total	Absolute	Mean	—	0.1	—	—	—	—	—	0.0	—	—	—	-0.6	—	—	-2.7	—	—	—	-0.2	1.8	—	—
	sugars (g	change	Median	—	0.0	—	—	—	—	—	0.0	—	—	—	0.0	—	—	-3.6	—	—	—	0.0	0.2	—	—
	per 100	Percent	Mean	—	2.7	—	—	—	—	—	0.0	—	—	—	-0.9	—	—	-22.1	—	—	—	-1.6	25.9	—	—
	g/mL)	change (%)	Median	—	0.0	—	—	—	—	—	0.0	—	—	—	0.0	—	—	-31.0	—	—	—	0.0	1.8	—	—
	Free sugars	Absolute	Mean	—	0.0	—	—	—	—	—	0.0	—	—	—	-1.2	—	—	-2.8	—	—	—	-0.2	-0.5	—	—
	(g per 100	change	Median	—	0.0	—	—	—	—	—	0.0	—	—	—	0.0	—	—	-3.8	—	—	—	0.0	0.0	—	—
	g/mL)	Percent	Mean	—	-0.2	—	—	—	—	—	N/A	—	—	—	-11.3	—	—	-36.7	—	—	—	-6.6	1.3	—	—
		change (%)	Median	—	-0.2	—	—	—	—	—	N/A	—	—	—	-11.9	—	—	-53.1	—	—	—	-6.3	1.3	—	—
Yogurt, yogurt	Number of products			0	12	1	0	0	0	37	38	0	3	0	7	0	0	3	0	16	0	4	0	0	0
drinks and	HSR	Absolute	Mean	—	0.3	0.0	—	—	—	0.0	0.0	—	0.0	—	0.1	—	—	0.2	—	-0.1	—	0.0	—	—	—
shakes (D12,		change	Median	—	0.5	0.0	—	—	—	0.0	0.0	—	0.0	—	0.0	—	—	0.0	—	0.0	—	0.0	—	—	—
D13, D15)		Percent	Mean	—	10.0	0.0	—	—	—	0.3	-3.7	—	0.0	—	1.6	—	—	4.8	—	-0.8	—	0.0	—	—	—
		change (%)	Median	—	16.7	0.0	—	—	—	0.0	0.0	—	0.0	—	0.0	—	—	0.0	—	0.0	—	0.0	—	—	—
	Calories	Absolute	Mean	—	-8.1	0.0	—	—	—	1.2	-1.1	—	0.0	—	-7.3	—	—	-2.8	—	-0.2	—	-6.6	—	—	—
	(kcal per	change	Median	—	0.0	0.0	—	—	—	0.0	0.0	—	0.0	—	-5.7	—	—	0.0	—	0.0	—	-6.6	—	—	—
	100 g/mL)	Percent	Mean	—	-9.4	0.0	—	—	—	2.5	-1.5	—	0.0	—	-8.6	—	—	-3.7	—	4.1	—	-6.9	—	—	—
		change (%)	Median	—	0.0	0.0	—	—	—	0.0	0.0	—	0.0	—	-6.3	—	—	0.0	—	0.0	—	-6.8	—	—	—
	Sodium	Absolute	Mean	—	-3.9	0.0	—	—	—	6.5	-0.6	—	2.3	—	-13.9	—	—	2.8	—	1.4	—	-0.9	—	—	—
	(mg per	change	Median	—	-5.0	0.0	—	—	—	8.6	0.0	—	3.4	—	-17.1	—	—	0.0	—	0.0	—	-0.9	—	—	—
	100 g/mL)	Percent	Mean	—	-8.3	0.0	—	—	—	15.2	-1.5	—	3.1	—	-23.9	—	—	5.6	—	3.2	—	-1.6	—	—	—
		change (%)	Median	—	-10.0	0.0	—	—	—	18.8	0.0	—	4.5	—	-30.0	—	—	0.0	—	0.0	—	-1.5	—	—	—
	Saturated	Absolute	Mean	—	-0.3	0.0	—	—	—	0.0	0.0	—	0.0	—	0.0	—	—	-0.1	—	-0.1	—	0.2	—	—	—
	fat (g per	change	Median	—	0.0	0.0	—	—	—	0.0	0.0	—	0.0	—	0.0	—	—	0.0	—	0.0	—	0.2	—	—	—
	100 g/mL)	Percent	Mean	—	-35.7	0.0	—	—	—	-0.8	-4.7	—	0.0	—	0.0	—	—	-11.1	—	-6.0	—	19.8	—	—	—
		change (%)	Median	—	-50.0	0.0	—	—	—	0.0	0.0	—	0.0	—	0.0	—	—	0.0	—	0.0	—	19.8	—	—	—
	Trans fat (g	Absolute	Mean	—	0.0	0.0	—	—	—	0.0	-0.1	—	0.0	—	0.0	—	—	0.0	—	0.0	—	0.0	—	—	—
	per 100	change	Median	—	0.0	0.0	—	—	—	0.0	0.0	—	0.0	—	0.0	—	—	0.0	—	0.0	—	0.0	—	—	—
	g/mL)	Percent	Mean	—	0.0	0.0	—	—	—	3.6	-23.2	—	N/A	—	0.0	—	—	N/A	—	0.0	—	4.8	—	—	—
		change (%)	Median	—	0.0	0.0	—	—	—	0.0	0.0	—	N/A	—	0.0	—	—	N/A	—	0.0	—	4.8	—	—	—
	Total	Absolute	Mean	—	-1.5	0.0	—	—	—	0.0	-0.4	—	-0.1	—	1.2	—	—	-0.8	—	0.6	—	-0.3	—	—	—
	sugars (g	change	Median	—	-1.0	0.0	—	—	—	0.0	0.0	—	0.0	—	1.1	—	—	0.0	—	0.0	—	-0.4	—	—	—
	per 100	Percent	Mean	—	-19.2	0.0	—	—	—	3.7	-2.8	—	-1.8	—	40.6	—	—	-7.1	—	15.1	—	-2.1	—	—	—
	g/mL)	change (%)	Median	—	-22.9	0.0	—	—	—	0.0	0.0	—	0.0	—	16.7	—	—	0.0	—	0.0	—	-3.1	—	—	—
	Free sugars	Absolute	Mean	—	-1.1	3.1	—	—	—	0.0	-0.4	—	3.1	—	1.0	—	—	2.6	—	0.6	—	2.7	—	—	—
	(g per 100	change	Median	—	-0.3	3.1	—	—	—	-0.3	0.0	—	3.2	—	0.8	—	—	3.0	—	0.0	—	2.5	—	—	—
	g/mL)	Percent	Mean	—	-30.1	39.2	—	—	—	-3.4	-6.7	—	98.6	—	42.7	—	—	27.8	—	1.1	—	24.0	—	—	—
		change (%)	Median	—	-25.7	39.2	—	—	—	-3.2	-9.0	—	102.1	—	30.4	—	—	28.0	—	0.1	—	22.7	—	—	—
**Desserts (E)**	**Number of products**			**0**	**0**	**0**	**0**	**0**	**0**	**0**	**0**	**0**	**0**	**29**	**50**	**0**	**0**	**49**	**0**	**0**	**0**	**0**	**48**	**0**	**28**
	**HSR**	**Absolute**	**Mean**	—	—	—	—	—	—	—	—	—	—	**0.0**	**0.0**	—	—	**0.1**	—	—	—	—	**0.0**	—	**0.0**
		**change**	**Median**	—	—	—	—	—	—	—	—	—	—	**0.0**	**0.0**	—	—	**0.0**	—	—	—	—	**0.0**	—	**0.0**
		**Percent**	**Mean**	—	—	—	—	—	—	—	—	—	—	**-0.5**	**0.1**	—	—	**6.4**	—	—	—	—	**0.0**	—	**3.0**
		**change**	**Median**	—	—	—	—	—	—	—	—	—	—	**0.0**	**0.0**	—	—	**0.0**	—	—	—	—	**0.0**	—	**0.0**
	**Calories**	**Absolute**	**Mean**	—	—	—	—	—	—	—	—	—	—	**-0.7**	**-1.1**	—	—	**-2.9**	—	—	—	—	**1.2**	—	**-8.5**
	**(kcal per**	**change**	**Median**	—	—	—	—	—	—	—	—	—	—	**0.0**	**0.0**	—	—	**0.0**	—	—	—	—	**0.0**	—	**-8.0**
	**100 g/mL)**	**Percent**	**Mean**	—	—	—	—	—	—	—	—	—	—	**-1.2**	**-0.5**	—	—	**-2.0**	—	—	—	—	**0.9**	—	**-5.7**
		**change**	**Median**	—	—	—	—	—	—	—	—	—	—	**0.0**	**0.0**	—	—	**0.0**	—	—	—	—	**0.0**	—	**-5.8**
	**Sodium**	**Absolute**	**Mean**	—	—	—	—	—	—	—	—	—	—	**3.0**	**-11.1**	—	—	**-1.0**	—	—	—	—	**-3.0**	—	**-2.6**
	**(mg per**	**change**	**Median**	—	—	—	—	—	—	—	—	—	—	**0.0**	**0.0**	—	—	**0.0**	—	—	—	—	**0.0**	—	**-4.0**
	**100 g/mL)**	**Percent**	**Mean**	—	—	—	—	—	—	—	—	—	—	**2.6**	**-7.8**	—	—	**8.8**	—	—	—	—	**-0.8**	—	**-5.0**
		**change**	**Median**	—	—	—	—	—	—	—	—	—	—	**0.0**	**0.0**	—	—	**0.0**	—	—	—	—	**0.0**	—	**-9.1**
	**Saturated**	**Absolute**	**Mean**	—	—	—	—	—	—	—	—	—	—	**0.1**	**0.0**	—	—	**-0.4**	—	—	—	—	**0.0**	—	**-0.6**
	**fat (g per**	**change**	**Median**	—	—	—	—	—	—	—	—	—	—	**0.0**	**0.0**	—	—	**0.0**	—	—	—	—	**0.0**	—	**-0.4**
	**100 g/mL)**	**Percent**	**Mean**	—	—	—	—	—	—	—	—	—	—	**40.9**	**6.3**	—	—	**-5.5**	—	—	—	—	**-6.0**	—	**-8.7**
		**change**	**Median**	—	—	—	—	—	—	—	—	—	—	**50.0**	**0.0**	—	—	**0.0**	—	—	—	—	**0.0**	—	**-11.1**
	**Trans fat**	**Absolute**	**Mean**	—	—	—	—	—	—	—	—	—	—	**0.0**	**0.0**	—	—	**0.0**	—	—	—	—	**0.0**	—	**-0.1**
	**(g per 100**	**change**	**Median**	—	—	—	—	—	—	—	—	—	—	**0.0**	**0.0**	—	—	**0.0**	—	—	—	—	**0.0**	—	**-0.1**
	**g/mL)**	**Percent**	**Mean**	—	—	—	—	—	—	—	—	—	—	**N/A**	**N/A**	—	—	**N/A**	—	—	—	—	**N/A**	—	**-24.8**
		**change**	**Median**	—	—	—	—	—	—	—	—	—	—	**N/A**	**0.0**	—	—	**0.0**	—	—	—	—	**0.0**	—	**-33.3**
	**Total**	**Absolute**	**Mean**	—	—	—	—	—	—	—	—	—	—	**0.0**	**0.1**	—	—	**-0.3**	—	—	—	—	**-0.1**	—	**-0.8**
	**sugars (g**	**change**	**Median**	—	—	—	—	—	—	—	—	—	—	**0.0**	**0.0**	—	—	**0.0**	—	—	—	—	**0.0**	—	**-0.6**
	**per 100**	**Percent**	**Mean**	—	—	—	—	—	—	—	—	—	—	**0.2**	**1.0**	—	—	**-1.6**	—	—	—	—	**-0.8**	—	**-5.4**
	**g/mL)**	**change**	**Median**	—	—	—	—	—	—	—	—	—	—	**0.0**	**0.0**	—	—	**0.0**	—	—	—	—	**0.0**	—	**-3.5**
	**Free**	**Absolute**	**Mean**	—	—	—	—	—	—	—	—	—	—	**0.2**	**-0.3**	—	—	**-0.9**	—	—	—	—	**-0.8**	—	**-1.8**
	**sugars (g**	**change**	**Median**	—	—	—	—	—	—	—	—	—	—	**0.0**	**-0.2**	—	—	**-0.2**	—	—	—	—	**-0.2**	—	**-1.9**
	**per 100**	**Percent**	**Mean**	—	—	—	—	—	—	—	—	—	—	**3.7**	**-1.3**	—	—	**-7.6**	—	—	—	—	**-6.2**	—	**-13.7**
	**g/mL)**	**change**	**Median**	—	—	—	—	—	—	—	—	—	—	**0.0**	**-2.7**	—	—	**-3.0**	—	—	—	—	**-2.3**	—	**-13.8**
**Dessert**	**Number of products**			**0**	**0**	**0**	**0**	**0**	**0**	**0**	**5**	**0**	**0**	**1**	**11**	**0**	**0**	**0**	**0**	**0**	**0**	**0**	**5**	**0**	**0**

				A. Lassonde	Agropur	Campbell Soup	Canada Bread	Canada Dry Mott's	Coca-Cola	Danone	General Mills	George Weston	Kellogg	Kraft Heinz	Loblaw	Maple Leaf Foods	Mondelēz	Nestlé	Ocean Spray	Parmalat	PepsiCo	Saputo	Sobeys	Sun-Rype	Unilever
toppings and	HSR	Absolute	Mean	—	—	—	—	—	—	—	-0.4	—	—	0.0	0.0	—	—	—	—	—	—	—	-0.1	—	—
fillings (F)		change	Median	—	—	—	—	—	—	—	-0.5	—	—	0.0	0.0	—	—	—	—	—	—	—	0.0	—	—
		Percent	Mean	—	—	—	—	—	—	—	-30.0	—	—	0.0	-1.8	—	—	—	—	—	—	—	-2.9	—	—
		change	Median	—	—	—	—	—	—	—	-33.3	—	—	0.0	0.0	—	—	—	—	—	—	—	0.0	—	—
	Calories	Absolute	Mean	—	—	—	—	—	—	—	6.1	—	—	38.5	-7.8	—	—	—	—	—	—	—	5.3	—	—
	(kcal per	change	Median	—	—	—	—	—	—	—	0.0	—	—	38.5	0.0	—	—	—	—	—	—	—	0.0	—	—
	100 g/mL)	Percent	Mean	—	—	—	—	—	—	—	1.5	—	—	12.5	-4.1	—	—	—	—	—	—	—	4.5	—	—
		change	Median	—	—	—	—	—	—	—	0.0	—	—	12.5	0.0	—	—	—	—	—	—	—	0.0	—	—
	Sodium	Absolute	Mean	—	—	—	—	—	—	—	0.0	—	—	0.0	3.0	—	—	—	—	—	—	—	8.0	—	—
	(mg per	change	Median	—	—	—	—	—	—	—	0.0	—	—	0.0	0.0	—	—	—	—	—	—	—	0.0	—	—
	100 g/mL)	Percent	Mean	—	—	—	—	—	—	—	0.0	—	—	0.0	-11.5	—	—	—	—	—	—	—	33.3	—	—
		change	Median	—	—	—	—	—	—	—	0.0	—	—	0.0	0.0	—	—	—	—	—	—	—	0.0	—	—
	Saturated	Absolute	Mean	—	—	—	—	—	—	—	5.3	—	—	0.0	0.0	—	—	—	—	—	—	—	0.0	—	—
	fat (g per	change	Median	—	—	—	—	—	—	—	6.3	—	—	0.0	0.0	—	—	—	—	—	—	—	0.0	—	—
	100 g/mL)	Percent	Mean	—	—	—	—	—	—	—	103.3	—	—	N/A	0.4	—	—	—	—	—	—	—	0.0	—	—
		change	Median	—	—	—	—	—	—	—	100.0	—	—	N/A	0.0	—	—	—	—	—	—	—	0.0	—	—
	Trans fat	Absolute	Mean	—	—	—	—	—	—	—	-5.6	—	—	0.0	0.0	—	—	—	—	—	—	—	0.0	—	—
	(g per 100	change	Median	—	—	—	—	—	—	—	-5.8	—	—	0.0	0.0	—	—	—	—	—	—	—	0.0	—	—
	g/mL)	Percent	Mean	—	—	—	—	—	—	—	-97.3	—	—	N/A	1.3	—	—	—	—	—	—	—	N/A	—	—
		change	Median	—	—	—	—	—	—	—	-100.0	—	—	N/A	1.3	—	—	—	—	—	—	—	N/A	—	—
	Total	Absolute	Mean	—	—	—	—	—	—	—	0.0	—	—	-7.7	1.8	—	—	—	—	—	—	—	-0.3	—	—
	sugars (g	change	Median	—	—	—	—	—	—	—	0.0	—	—	-7.7	0.0	—	—	—	—	—	—	—	0.0	—	—
	per 100	Percent	Mean	—	—	—	—	—	—	—	0.0	—	—	-11.1	6.2	—	—	—	—	—	—	—	-0.7	—	—
	g/mL)	change	Median	—	—	—	—	—	—	—	0.0	—	—	-11.1	0.0	—	—	—	—	—	—	—	0.0	—	—
	Free	Absolute	Mean	—	—	—	—	—	—	—	0.0	—	—	-7.7	-4.6	—	—	—	—	—	—	—	-6.3	—	—
	sugars (g	change	Median	—	—	—	—	—	—	—	0.0	—	—	-7.7	-1.2	—	—	—	—	—	—	—	-8.2	—	—
	per 100	Percent	Mean	—	—	—	—	—	—	—	0.0	—	—	-11.1	-14.3	—	—	—	—	—	—	—	-26.4	—	—
	g/mL)	change	Median	—	—	—	—	—	—	—	0.0	—	—	-11.1	-4.9	—	—	—	—	—	—	—	-34.0	—	—
Eggs and egg	Number of products			0	0	0	0	0	0	0	0	0	0	0	7	0	0	0	0	0	0	0	3	0	0
substitutes	HSR	Absolute	Mean	—	—	—	—	—	—	—	—	—	—	—	0.0	—	—	—	—	—	—	—	0.0	—	—
(G)		change	Median	—	—	—	—	—	—	—	—	—	—	—	0.0	—	—	—	—	—	—	—	0.0	—	—
		Percent	Mean	—	—	—	—	—	—	—	—	—	—	—	0.0	—	—	—	—	—	—	—	0.0	—	—
		change	Median	—	—	—	—	—	—	—	—	—	—	—	0.0	—	—	—	—	—	—	—	0.0	—	—
	Calories	Absolute	Mean	—	—	—	—	—	—	—	—	—	—	—	0.0	—	—	—	—	—	—	—	0.0	—	—
	(kcal per	change	Median	—	—	—	—	—	—	—	—	—	—	—	0.0	—	—	—	—	—	—	—	0.0	—	—
	100 g/mL)	Percent	Mean	—	—	—	—	—	—	—	—	—	—	—	0.0	—	—	—	—	—	—	—	0.0	—	—
		change	Median	—	—	—	—	—	—	—	—	—	—	—	0.0	—	—	—	—	—	—	—	0.0	—	—
	Sodium	Absolute	Mean	—	—	—	—	—	—	—	—	—	—	—	0.0	—	—	—	—	—	—	—	0.0	—	—
	(mg per	change	Median	—	—	—	—	—	—	—	—	—	—	—	0.0	—	—	—	—	—	—	—	0.0	—	—
	100 g/mL)	Percent	Mean	—	—	—	—	—	—	—	—	—	—	—	0.0	—	—	—	—	—	—	—	0.0	—	—
		change	Median	—	—	—	—	—	—	—	—	—	—	—	0.0	—	—	—	—	—	—	—	0.0	—	—
	Saturated	Absolute	Mean	—	—	—	—	—	—	—	—	—	—	—	0.0	—	—	—	—	—	—	—	0.0	—	—
	fat (g per	change	Median	—	—	—	—	—	—	—	—	—	—	—	0.0	—	—	—	—	—	—	—	0.0	—	—
	100 g/mL)	Percent	Mean	—	—	—	—	—	—	—	—	—	—	—	0.0	—	—	—	—	—	—	—	0.0	—	—
		change	Median	—	—	—	—	—	—	—	—	—	—	—	0.0	—	—	—	—	—	—	—	0.0	—	—
	Trans fat	Absolute	Mean	—	—	—	—	—	—	—	—	—	—	—	0.0	—	—	—	—	—	—	—	0.0	—	—
	(g per 100	change	Median	—	—	—	—	—	—	—	—	—	—	—	0.0	—	—	—	—	—	—	—	0.0	—	—
	g/mL)	Percent	Mean	—	—	—	—	—	—	—	—	—	—	—	N/A	—	—	—	—	—	—	—	N/A	—	—
		change	Median	—	—	—	—	—	—	—	—	—	—	—	N/A	—	—	—	—	—	—	—	N/A	—	—
	Total	Absolute	Mean	—	—	—	—	—	—	—	—	—	—	—	0.0	—	—	—	—	—	—	—	0.0	—	—
	sugars (g	change	Median	—	—	—	—	—	—	—	—	—	—	—	0.0	—	—	—	—	—	—	—	0.0	—	—
	per 100	Percent	Mean	—	—	—	—	—	—	—	—	—	—	—	N/A	—	—	—	—	—	—	—	N/A	—	—
	g/mL)	change	Median	—	—	—	—	—	—	—	—	—	—	—	N/A	—	—	—	—	—	—	—	N/A	—	—
	Free	Absolute	Mean	—	—	—	—	—	—	—	—	—	—	—	0.0	—	—	—	—	—	—	—	0.0	—	—
	sugars (g	change	Median	—	—	—	—	—	—	—	—	—	—	—	0.0	—	—	—	—	—	—	—	0.0	—	—
	per 100	Percent	Mean	—	—	—	—	—	—	—	—	—	—	—	N/A	—	—	—	—	—	—	—	N/A	—	—
	g/mL)	change	Median	—	—	—	—	—	—	—	—	—	—	—	N/A	—	—	—	—	—	—	—	N/A	—	—
Fats and oils	Number of products			0	0	0	1	0	0	0	0	0	0	76	80	0	0	0	0	8	0	0	36	0	14
(H)	HSR	Absolute	Mean	—	—	—	0.0	—	—	—	—	—	—	2.6	1.3	—	—	—	—	0.0	—	—	1.4	—	0.9
		change	Median	—	—	—	0.0	—	—	—	—	—	—	2.5	0.5	—	—	—	—	0.0	—	—	2.0	—	0.3
		Percent	Mean	—	—	—	0.0	—	—	—	—	—	—	163.3	91.7	—	—	—	—	0.0	—	—	102.2	—	65.5
		change	Median	—	—	—	0.0	—	—	—	—	—	—	150.0	16.7	—	—	—	—	0.0	—	—	100.0	—	8.3
	Calories	Absolute	Mean	—	—	—	0.0	—	—	—	—	—	—	-11.8	1.5	—	—	—	—	0.0	—	—	2.8	—	-3.6
	(kcal per	change	Median	—	—	—	0.0	—	—	—	—	—	—	0.0	0.0	—	—	—	—	0.0	—	—	0.0	—	0.0
	100 g/mL)	Percent	Mean	—	—	—	0.0	—	—	—	—	—	—	-1.7	-0.3	—	—	—	—	0.0	—	—	0.5	—	-1.0
		change	Median	—	—	—	0.0	—	—	—	—	—	—	0.0	0.0	—	—	—	—	0.0	—	—	0.0	—	0.0
	Sodium	Absolute	Mean	—	—	—	0.0	—	—	—	—	—	—	-74.2	-69.8	—	—	—	—	16.3	—	—	-27.3	—	-23.8
	(mg per	change	Median	—	—	—	0.0	—	—	—	—	—	—	0.0	0.0	—	—	—	—	0.0	—	—	0.0	—	0.0
	100 g/mL)	Percent	Mean	—	—	—	N/A	—	—	—	—	—	—	-6.5	-10.5	—	—	—	—	-10.7	—	—	-3.9	—	-3.7
		change	Median	—	—	—	N/A	—	—	—	—	—	—	0.0	-7.7	—	—	—	—	0.0	—	—	0.0	—	0.0
	Saturated	Absolute	Mean	—	—	—	0.0	—	—	—	—	—	—	-0.4	0.0	—	—	—	—	0.0	—	—	0.1	—	-0.7
	fat (g per	change	Median	—	—	—	0.0	—	—	—	—	—	—	0.0	0.0	—	—	—	—	0.0	—	—	0.0	—	0.0
	100 g/mL)	Percent	Mean	—	—	—	0.0	—	—	—	—	—	—	-11.6	-0.9	—	—	—	—	0.0	—	—	4.9	—	-1.8

				A. Lassonde	Agropur	Campbell Soup	Canada Bread	Canada Dry Mott's	Coca-Cola	Danone	General Mills	George Weston	Kellogg	Kraft Heinz	Loblaw	Maple Leaf Foods	Mondelēz	Nestlé	Ocean Spray	Parmalat	PepsiCo	Saputo	Sobeys	Sun-Rype	Unilever
		**change**	**Median**	—	—	—	**0.0**	—	—	—	—	—	—	**0.0**	**0.0**	—	—	—	—	**0.0**	—	—	**0.0**	—	**0.0**
	**Trans fat (g per 100 g/mL)**	**Absolute change**	**Mean**	—	—	—	**0.0**	—	—	—	—	—	—	**0.0**	**0.0**	—	—	—	—	**0.1**	—	—	**0.1**	—	**0.0**
			**Median**	—	—	—	**0.0**	—	—	—	—	—	—	**0.0**	**0.0**	—	—	—	—	**0.0**	—	—	**0.0**	—	**0.0**
		**Percent change**	**Mean**	—	—	—	**0.0**	—	—	—	—	—	—	**-71.4**	**-22.7**	—	—	—	—	**10.0**	—	—	**6.3**	—	**0.0**
			**Median**	—	—	—	**0.0**	—	—	—	—	—	—	**-100.0**	**0.0**	—	—	—	—	**0.0**	—	—	**0.0**	—	**0.0**
	**Total sugars (g per 100 g/mL)**	**Absolute change**	**Mean**	—	—	—	**0.0**	—	—	—	—	—	—	**-0.2**	**0.0**	—	—	—	—	**0.0**	—	—	**-0.4**	—	**0.0**
			**Median**	—	—	—	**0.0**	—	—	—	—	—	—	**0.0**	**0.0**	—	—	—	—	**0.0**	—	—	**0.0**	—	**0.0**
		**Percent change**	**Mean**	—	—	—	**N/A**	—	—	—	—	—	—	**-4.6**	**-8.5**	—	—	—	—	**N/A**	—	—	**-15.4**	—	**N/A**
			**Median**	—	—	—	**N/A**	—	—	—	—	—	—	**0.0**	**0.0**	—	—	—	—	**N/A**	—	—	**0.0**	—	**N/A**
	**Free sugars (g per 100 g/mL)**	**Absolute change**	**Mean**	—	—	—	**0.0**	—	—	—	—	—	—	**-0.2**	**-0.1**	—	—	—	—	**0.0**	—	—	**-0.3**	—	**0.0**
			**Median**	—	—	—	**0.0**	—	—	—	—	—	—	**0.0**	**0.0**	—	—	—	—	**0.0**	—	—	**0.0**	—	**0.0**
		**Percent change**	**Mean**	—	—	—	**N/A**	—	—	—	—	—	—	**-3.4**	**-9.8**	—	—	—	—	**N/A**	—	—	**-14.9**	—	**N/A**
			**Median**	—	—	—	**N/A**	—	—	—	—	—	—	**-0.1**	**-0.1**	—	—	—	—	**N/A**	—	—	**-1.3**	—	**N/A**
Butter, margarine, shortening, lard, etc. (H1)	Number of products			0	0	0	1	0	0	0	0	0	0	0	12	0	0	0	0	8	0	0	6	0	10
	HSR	Absolute change	Mean	—	—	—	0.0	—	—	—	—	—	—	—	0.1	—	—	—	—	0.0	—	—	0.0	—	0.2
			Median	—	—	—	0.0	—	—	—	—	—	—	—	0.0	—	—	—	—	0.0	—	—	0.0	—	0.0
		Percent change (%)	Mean	—	—	—	0.0	—	—	—	—	—	—	—	4.4	—	—	—	—	0.0	—	—	0.0	—	9.2
			Median	—	—	—	0.0	—	—	—	—	—	—	—	0.0	—	—	—	—	0.0	—	—	0.0	—	0.0
	Calories (kcal per 100 g/mL)	Absolute change	Mean	—	—	—	0.0	—	—	—	—	—	—	—	8.3	—	—	—	—	0.0	—	—	0.0	—	-5.0
			Median	—	—	—	0.0	—	—	—	—	—	—	—	0.0	—	—	—	—	0.0	—	—	0.0	—	0.0
		Percent change (%)	Mean	—	—	—	0.0	—	—	—	—	—	—	—	1.4	—	—	—	—	0.0	—	—	0.0	—	-1.4
			Median	—	—	—	0.0	—	—	—	—	—	—	—	0.0	—	—	—	—	0.0	—	—	0.0	—	0.0
	Sodium (mg per 100 g/mL)	Absolute change	Mean	—	—	—	0.0	—	—	—	—	—	—	—	-29.2	—	—	—	—	16.3	—	—	-41.7	—	-20.0
			Median	—	—	—	0.0	—	—	—	—	—	—	—	0.0	—	—	—	—	0.0	—	—	0.0	—	0.0
		Percent change (%)	Mean	—	—	—	N/A	—	—	—	—	—	—	—	-6.7	—	—	—	—	-10.7	—	—	-15.6	—	-3.3
			Median	—	—	—	N/A	—	—	—	—	—	—	—	0.0	—	—	—	—	0.0	—	—	-15.6	—	0.0
	Saturated fat (g per 100 g/mL)	Absolute change	Mean	—	—	—	0.0	—	—	—	—	—	—	—	0.0	—	—	—	—	0.0	—	—	0.8	—	-1.0
			Median	—	—	—	0.0	—	—	—	—	—	—	—	0.0	—	—	—	—	0.0	—	—	0.0	—	0.0
		Percent change (%)	Mean	—	—	—	0.0	—	—	—	—	—	—	—	0.0	—	—	—	—	0.0	—	—	1.9	—	-5.0
			Median	—	—	—	0.0	—	—	—	—	—	—	—	0.0	—	—	—	—	0.0	—	—	0.0	—	0.0
	Trans fat (g per 100 g/mL)	Absolute change	Mean	—	—	—	0.0	—	—	—	—	—	—	—	0.0	—	—	—	—	0.1	—	—	0.2	—	0.0
			Median	—	—	—	0.0	—	—	—	—	—	—	—	0.0	—	—	—	—	0.0	—	—	0.0	—	0.0
		Percent change (%)	Mean	—	—	—	0.0	—	—	—	—	—	—	—	0.0	—	—	—	—	10.0	—	—	10.0	—	0.0
			Median	—	—	—	0.0	—	—	—	—	—	—	—	0.0	—	—	—	—	0.0	—	—	0.0	—	0.0
	Total sugars (g per 100 g/mL)	Absolute change	Mean	—	—	—	0.0	—	—	—	—	—	—	—	0.0	—	—	—	—	0.0	—	—	0.0	—	0.0
			Median	—	—	—	0.0	—	—	—	—	—	—	—	0.0	—	—	—	—	0.0	—	—	0.0	—	0.0
		Percent change (%)	Mean	—	—	—	N/A	—	—	—	—	—	—	—	N/A	—	—	—	—	N/A	—	—	N/A	—	N/A
			Median	—	—	—	N/A	—	—	—	—	—	—	—	N/A	—	—	—	—	N/A	—	—	N/A	—	N/A
	Free sugars (g per 100 g/mL)	Absolute change	Mean	—	—	—	0.0	—	—	—	—	—	—	—	0.0	—	—	—	—	0.0	—	—	0.0	—	0.0
			Median	—	—	—	0.0	—	—	—	—	—	—	—	0.0	—	—	—	—	0.0	—	—	0.0	—	0.0
		Percent change (%)	Mean	—	—	—	N/A	—	—	—	—	—	—	—	N/A	—	—	—	—	N/A	—	—	N/A	—	N/A
			Median	—	—	—	N/A	—	—	—	—	—	—	—	N/A	—	—	—	—	N/A	—	—	N/A	—	N/A
Mayonnaise and salad dressings (H4, H5)	Number of products			0	0	0	0	0	0	0	0	0	0	76	38	0	0	0	0	0	0	0	20	0	4
	HSR	Absolute change	Mean	—	—	—	—	—	—	—	—	—	—	2.6	2.7	—	—	—	—	—	—	—	2.6	—	2.8
			Median	—	—	—	—	—	—	—	—	—	—	2.5	2.5	—	—	—	—	—	—	—	2.5	—	2.8
		Percent change (%)	Mean	—	—	—	—	—	—	—	—	—	—	163.3	191.6	—	—	—	—	—	—	—	183.3	—	206.3
			Median	—	—	—	—	—	—	—	—	—	—	150.0	166.7	—	—	—	—	—	—	—	183.3	—	200.0
	Calories (kcal per 100 g/mL)	Absolute change	Mean	—	—	—	—	—	—	—	—	—	—	-11.8	0.4	—	—	—	—	—	—	—	5.0	—	0.0
			Median	—	—	—	—	—	—	—	—	—	—	0.0	0.0	—	—	—	—	—	—	—	0.0	—	0.0
		Percent change (%)	Mean	—	—	—	—	—	—	—	—	—	—	-1.7	-1.0	—	—	—	—	—	—	—	0.9	—	0.0
			Median	—	—	—	—	—	—	—	—	—	—	0.0	0.0	—	—	—	—	—	—	—	0.0	—	0.0
	Sodium (mg per 100 g/mL)	Absolute change	Mean	—	—	—	—	—	—	—	—	—	—	-74.2	-137.6	—	—	—	—	—	—	—	-36.7	—	-33.3
			Median	—	—	—	—	—	—	—	—	—	—	0.0	-100.0	—	—	—	—	—	—	—	0.0	—	0.0
		Percent change (%)	Mean	—	—	—	—	—	—	—	—	—	—	-6.5	-11.3	—	—	—	—	—	—	—	-2.7	—	-4.5
			Median	—	—	—	—	—	—	—	—	—	—	0.0	-10.9	—	—	—	—	—	—	—	0.0	—	0.0
	Saturated fat (g per 100 g/mL)	Absolute change	Mean	—	—	—	—	—	—	—	—	—	—	-0.4	0.1	—	—	—	—	—	—	—	0.1	—	0.2
			Median	—	—	—	—	—	—	—	—	—	—	0.0	0.0	—	—	—	—	—	—	—	0.0	—	0.0
		Percent change (%)	Mean	—	—	—	—	—	—	—	—	—	—	-11.6	-1.9	—	—	—	—	—	—	—	9.6	—	6.2
			Median	—	—	—	—	—	—	—	—	—	—	0.0	0.0	—	—	—	—	—	—	—	0.0	—	0.0
	Trans fat (g per 100 g/mL)	Absolute change	Mean	—	—	—	—	—	—	—	—	—	—	0.0	-0.1	—	—	—	—	—	—	—	0.1	—	0.0
			Median	—	—	—	—	—	—	—	—	—	—	0.0	0.0	—	—	—	—	—	—	—	0.0	—	0.0
		Percent change (%)	Mean	—	—	—	—	—	—	—	—	—	—	-71.4	-41.7	—	—	—	—	—	—	—	0.0	—	N/A
			Median	—	—	—	—	—	—	—	—	—	—	-100.0	0.0	—	—	—	—	—	—	—	0.0	—	N/A
	Total sugars (g per 100 g/mL)	Absolute change	Mean	—	—	—	—	—	—	—	—	—	—	-0.2	0.0	—	—	—	—	—	—	—	-0.7	—	0.0
			Median	—	—	—	—	—	—	—	—	—	—	0.0	0.0	—	—	—	—	—	—	—	0.0	—	0.0
		Percent change (%)	Mean	—	—	—	—	—	—	—	—	—	—	-4.6	-8.5	—	—	—	—	—	—	—	-15.4	—	N/A
			Median	—	—	—	—	—	—	—	—	—	—	0.0	0.0	—	—	—	—	—	—	—	0.0	—	N/A
	Free sugars (g per 100 g/mL)	Absolute change	Mean	—	—	—	—	—	—	—	—	—	—	-0.2	-0.2	—	—	—	—	—	—	—	-0.6	—	0.0
			Median	—	—	—	—	—	—	—	—	—	—	0.0	0.0	—	—	—	—	—	—	—	0.0	—	0.0
		Percent change (%)	Mean	—	—	—	—	—	—	—	—	—	—	-3.4	-9.8	—	—	—	—	—	—	—	-14.9	—	N/A
			Median	—	—	—	—	—	—	—	—	—	—	-0.1	-0.1	—	—	—	—	—	—	—	-1.3	—	N/A
Oils (H2, H6)	Number of products			0	0	0	0	0	0	0	0	0	0	0	30	0	0	0	0	0	0	0	10	0	0
	HSR	Absolute	Mean	—	—	—	—	—	—	—	—	—	—	—	0.0	—	—	—	—	—	—	—	0.1	—	—

				A. Lassonde	Agropur	Campbell Soup	Canada Bread	Canada Dry Mott's	Coca-Cola	Danone	General Mills	George Weston	Kellogg	Kraft Heinz	Loblaw	Maple Leaf Foods	Mondelēz	Nestlé	Ocean Spray	Parmalat	PepsiCo	Saputo	Sobeys	Sun-Rype	Unilever
		change	Median	—	—	—	—	—	—	—	—	—	—	—	0.0	—	—	—	—	—	—	—	0.0	—	—
		Percent	Mean	—	—	—	—	—	—	—	—	—	—	—	0.0	—	—	—	—	—	—	—	1.4	—	—
		change (%)	Median	—	—	—	—	—	—	—	—	—	—	—	0.0	—	—	—	—	—	—	—	0.0	—	—
	Calories (kcal per 100 g/mL)	Absolute	Mean	—	—	—	—	—	—	—	—	—	—	—	0.0	—	—	—	—	—	—	—	0.0	—	—
		change	Median	—	—	—	—	—	—	—	—	—	—	—	0.0	—	—	—	—	—	—	—	0.0	—	—
		Percent	Mean	—	—	—	—	—	—	—	—	—	—	—	0.0	—	—	—	—	—	—	—	0.0	—	—
		change (%)	Median	—	—	—	—	—	—	—	—	—	—	—	0.0	—	—	—	—	—	—	—	0.0	—	—
	Sodium (mg per 100 g/mL)	Absolute	Mean	—	—	—	—	—	—	—	—	—	—	—	0.0	—	—	—	—	—	—	—	0.0	—	—
		change	Median	—	—	—	—	—	—	—	—	—	—	—	0.0	—	—	—	—	—	—	—	0.0	—	—
		Percent	Mean	—	—	—	—	—	—	—	—	—	—	—	N/A	—	—	—	—	—	—	—	N/A	—	—
		change (%)	Median	—	—	—	—	—	—	—	—	—	—	—	N/A	—	—	—	—	—	—	—	N/A	—	—
	Saturated fat (g per 100 g/mL)	Absolute	Mean	—	—	—	—	—	—	—	—	—	—	—	0.0	—	—	—	—	—	—	—	-0.5	—	—
		change	Median	—	—	—	—	—	—	—	—	—	—	—	0.0	—	—	—	—	—	—	—	0.0	—	—
		Percent	Mean	—	—	—	—	—	—	—	—	—	—	—	0.0	—	—	—	—	—	—	—	-4.2	—	—
		change (%)	Median	—	—	—	—	—	—	—	—	—	—	—	0.0	—	—	—	—	—	—	—	0.0	—	—
	Trans fat (g per 100 g/mL)	Absolute	Mean	—	—	—	—	—	—	—	—	—	—	—	0.0	—	—	—	—	—	—	—	0.0	—	—
		change	Median	—	—	—	—	—	—	—	—	—	—	—	0.0	—	—	—	—	—	—	—	0.0	—	—
		Percent	Mean	—	—	—	—	—	—	—	—	—	—	—	0.0	—	—	—	—	—	—	—	N/A	—	—
		change (%)	Median	—	—	—	—	—	—	—	—	—	—	—	0.0	—	—	—	—	—	—	—	N/A	—	—
	Total sugars (g per 100 g/mL)	Absolute	Mean	—	—	—	—	—	—	—	—	—	—	—	0.0	—	—	—	—	—	—	—	0.0	—	—
		change	Median	—	—	—	—	—	—	—	—	—	—	—	0.0	—	—	—	—	—	—	—	0.0	—	—
		Percent	Mean	—	—	—	—	—	—	—	—	—	—	—	N/A	—	—	—	—	—	—	—	N/A	—	—
		change (%)	Median	—	—	—	—	—	—	—	—	—	—	—	N/A	—	—	—	—	—	—	—	N/A	—	—
	Free sugars (g per 100 g/mL)	Absolute	Mean	—	—	—	—	—	—	—	—	—	—	—	0.0	—	—	—	—	—	—	—	0.0	—	—
		change	Median	—	—	—	—	—	—	—	—	—	—	—	0.0	—	—	—	—	—	—	—	0.0	—	—
		Percent	Mean	—	—	—	—	—	—	—	—	—	—	—	N/A	—	—	—	—	—	—	—	N/A	—	—
		change (%)	Median	—	—	—	—	—	—	—	—	—	—	—	N/A	—	—	—	—	—	—	—	N/A	—	—
**Marine and fresh water animals (I)**	**Number of products**			**0**	**0**	**0**	**0**	**0**	**0**	**0**	**0**	**0**	**0**	**0**	**44**	**0**	**0**	**0**	**0**	**0**	**0**	**0**	**25**	**0**	**0**
	**HSR**	**Absolute**	**Mean**	—	—	—	—	—	—	—	—	—	—	—	**0.0**	—	—	—	—	—	—	—	**0.1**	—	—
		**change**	**Median**	—	—	—	—	—	—	—	—	—	—	—	**0.0**	—	—	—	—	—	—	—	**0.0**	—	—
		**Percent**	**Mean**	—	—	—	—	—	—	—	—	—	—	—	**0.8**	—	—	—	—	—	—	—	**1.7**	—	—
		**change**	**Median**	—	—	—	—	—	—	—	—	—	—	—	**0.0**	—	—	—	—	—	—	—	**0.0**	—	—
	**Calories (kcal per 100 g/mL)**	**Absolute**	**Mean**	—	—	—	—	—	—	—	—	—	—	—	**1.6**	—	—	—	—	—	—	—	**4.8**	—	—
		**change**	**Median**	—	—	—	—	—	—	—	—	—	—	—	**0.0**	—	—	—	—	—	—	—	**0.0**	—	—
		**Percent**	**Mean**	—	—	—	—	—	—	—	—	—	—	—	**1.6**	—	—	—	—	—	—	—	**6.3**	—	—
		**change**	**Median**	—	—	—	—	—	—	—	—	—	—	—	**0.0**	—	—	—	—	—	—	—	**0.0**	—	—
	**Sodium (mg per 100 g/mL)**	**Absolute**	**Mean**	—	—	—	—	—	—	—	—	—	—	—	**-24.6**	—	—	—	—	—	—	—	**-36.6**	—	—
		**change**	**Median**	—	—	—	—	—	—	—	—	—	—	—	**0.0**	—	—	—	—	—	—	—	**0.0**	—	—
		**Percent**	**Mean**	—	—	—	—	—	—	—	—	—	—	—	**18.6**	—	—	—	—	—	—	—	**6.6**	—	—
		**change**	**Median**	—	—	—	—	—	—	—	—	—	—	—	**0.0**	—	—	—	—	—	—	—	**0.0**	—	—
	**Saturated fat (g per 100 g/mL)**	**Absolute**	**Mean**	—	—	—	—	—	—	—	—	—	—	—	**0.2**	—	—	—	—	—	—	—	**0.1**	—	—
		**change**	**Median**	—	—	—	—	—	—	—	—	—	—	—	**0.0**	—	—	—	—	—	—	—	**0.0**	—	—
		**Percent**	**Mean**	—	—	—	—	—	—	—	—	—	—	—	**7.2**	—	—	—	—	—	—	—	**34.0**	—	—
		**change**	**Median**	—	—	—	—	—	—	—	—	—	—	—	**0.0**	—	—	—	—	—	—	—	**0.0**	—	—
	**Trans fat (g per 100 g/mL)**	**Absolute**	**Mean**	—	—	—	—	—	—	—	—	—	—	—	**0.0**	—	—	—	—	—	—	—	**0.0**	—	—
		**change**	**Median**	—	—	—	—	—	—	—	—	—	—	—	**0.0**	—	—	—	—	—	—	—	**0.0**	—	—
		**Percent**	**Mean**	—	—	—	—	—	—	—	—	—	—	—	**-1.9**	—	—	—	—	—	—	—	**0.0**	—	—
		**change**	**Median**	—	—	—	—	—	—	—	—	—	—	—	**0.0**	—	—	—	—	—	—	—	**0.0**	—	—
	**Total sugars (g per 100 g/mL)**	**Absolute**	**Mean**	—	—	—	—	—	—	—	—	—	—	—	**-0.1**	—	—	—	—	—	—	—	**-0.3**	—	—
		**change**	**Median**	—	—	—	—	—	—	—	—	—	—	—	**0.0**	—	—	—	—	—	—	—	**0.0**	—	—
		**Percent**	**Mean**	—	—	—	—	—	—	—	—	—	—	—	**-21.3**	—	—	—	—	—	—	—	**-11.5**	—	—
		**change**	**Median**	—	—	—	—	—	—	—	—	—	—	—	**0.0**	—	—	—	—	—	—	—	**0.0**	—	—
	**Free sugars (g per 100 g/mL)**	**Absolute**	**Mean**	—	—	—	—	—	—	—	—	—	—	—	**0.0**	—	—	—	—	—	—	—	**-0.2**	—	—
		**change**	**Median**	—	—	—	—	—	—	—	—	—	—	—	**0.0**	—	—	—	—	—	—	—	**0.0**	—	—
		**Percent**	**Mean**	—	—	—	—	—	—	—	—	—	—	—	**-7.7**	—	—	—	—	—	—	—	**-8.5**	—	—
		**change**	**Median**	—	—	—	—	—	—	—	—	—	—	—	**0.2**	—	—	—	—	—	—	—	**-2.4**	—	—
**Fruit and fruit juices (J)**	**Number of products**			**33**	**0**	**6**	**0**	**18**	**51**	**0**	**0**	**0**	**0**	**8**	**109**	**0**	**0**	**0**	**23**	**0**	**16**	**0**	**84**	**11**	**0**
	**HSR**	**Absolute**	**Mean**	**-0.2**	—	**0.1**	—	**-0.1**	**0.1**	—	—	—	—	**0.0**	**0.0**	—	—	—	**0.6**	—	**0.0**	—	**0.0**	**0.0**	—
		**change**	**Median**	**0.0**	—	**0.0**	—	**0.0**	**0.0**	—	—	—	—	**0.0**	**0.0**	—	—	—	**0.0**	—	**0.0**	—	**0.0**	**0.0**	—
		**Percent**	**Mean**	**-4.1**	—	**7.8**	—	**-2.8**	**8.0**	—	—	—	—	**0.0**	**3.5**	—	—	—	**37.1**	—	**0.0**	—	**1.0**	**0.0**	—
		**change**	**Median**	**0.0**	—	**0.0**	—	**0.0**	**0.0**	—	—	—	—	**0.0**	**0.0**	—	—	—	**0.0**	—	**0.0**	—	**0.0**	**0.0**	—
	**Calories (kcal per 100 g/mL)**	**Absolute**	**Mean**	**0.0**	—	**-6.0**	—	**-0.8**	**0.1**	—	—	—	—	**0.0**	**-0.8**	—	—	—	**-5.8**	—	**0.3**	—	**0.2**	**-1.3**	—
		**change**	**Median**	**0.0**	—	**-2.0**	—	**0.0**	**0.0**	—	—	—	—	**0.0**	**0.0**	—	—	—	**-4.0**	—	**0.0**	—	**0.0**	**0.0**	—
		**Percent**	**Mean**	**0.0**	—	**-9.3**	—	**-2.0**	**0.2**	—	—	—	—	**0.0**	**-1.1**	—	—	—	**-5.6**	—	**0.6**	—	**-0.3**	**-2.6**	—
		**change**	**Median**	**0.0**	—	**-3.6**	—	**0.0**	**0.0**	—	—	—	—	**0.0**	**0.0**	—	—	—	**-7.1**	—	**0.0**	—	**0.0**	**0.0**	—
	**Sodium (mg per 100 g/mL)**	**Absolute**	**Mean**	**-1.8**	—	**-1.0**	—	**0.0**	**0.0**	—	—	—	—	**0.0**	**-1.3**	—	—	—	**-2.6**	—	**0.4**	—	**-0.3**	**1.1**	—
		**change**	**Median**	**-2.0**	—	**0.0**	—	**0.0**	**0.0**	—	—	—	—	**0.0**	**0.0**	—	—	—	**0.0**	—	**0.0**	—	**0.0**	**0.0**	—
		**Percent**	**Mean**	**-13.2**	—	**-11.1**	—	**0.0**	**0.5**	—	—	—	—	**0.0**	**N/A**	—	—	—	**-17.1**	—	**N/A**	—	**N/A**	**4.2**	—
		**change**	**Median**	**-20.0**	—	**0.0**	—	**0.0**	**0.0**	—	—	—	—	**0.0**	**0.0**	—	—	—	**0.0**	—	**0.0**	—	**0.0**	**0.0**	—
	**Saturated fat (g per 100 g/mL)**	**Absolute**	**Mean**	**0.0**	—	**0.0**	—	**0.0**	**0.0**	—	—	—	—	**0.0**	**0.0**	—	—	—	**0.0**	—	**0.0**	—	**0.0**	**0.0**	—
		**change**	**Median**	**0.0**	—	**0.0**	—	**0.0**	**0.0**	—	—	—	—	**0.0**	**0.0**	—	—	—	**0.0**	—	**0.0**	—	**0.0**	**0.0**	—
		**Percent**	**Mean**	**N/A**	—	**N/A**	—	**N/A**	**N/A**	—	—	—	—	**N/A**	**0.0**	—	—	—	**N/A**	—	**N/A**	—	**N/A**	**N/A**	—
		**change**	**Median**	**N/A**	—	**-100.0**	—	**N/A**	**N/A**	—	—	—	—	**N/A**	**0.0**	—	—	—	**N/A**	—	**N/A**	—	**N/A**	**N/A**	—

				A. Lassonde	Agropur	Campbell Soup	Canada Bread	Canada Dry Mott's	Coca-Cola	Danone	General Mills	George Weston	Kellogg	Kraft Heinz	Loblaw	Maple Leaf Foods	Mondelēz	Nestlé	Ocean Spray	Parmalat	PepsiCo	Saputo	Sobeys	Sun-Rype	Unilever
	**Trans fat (g per 100 g/mL)**	**Absolute change**	**Mean**	**0.0**	—	**0.0**	—	**0.0**	**0.0**	—	—	—	—	**0.0**	**0.0**	—	—	—	**0.0**	—	**0.0**	—	**0.0**	**0.0**	—
			**Median**	**0.0**	—	**0.0**	—	**0.0**	**0.0**	—	—	—	—	**0.0**	**0.0**	—	—	—	**0.0**	—	**0.0**	—	**0.0**	**0.0**	—
		**Percent change**	**Mean**	**N/A**	—	**N/A**	—	**N/A**	**N/A**	—	—	—	—	**N/A**	**N/A**	—	—	—	**N/A**	—	**N/A**	—	**N/A**	**N/A**	—
			**Median**	**N/A**	—	**N/A**	—	**N/A**	**N/A**	—	—	—	—	**N/A**	**N/A**	—	—	—	**N/A**	—	**N/A**	—	**N/A**	**N/A**	—
	**Total sugars (g per 100 g/mL)**	**Absolute change**	**Mean**	**-0.1**	—	**-1.7**	—	**-0.1**	**0.0**	—	—	—	—	**0.0**	**0.1**	—	—	—	**0.6**	—	**0.1**	—	**0.2**	**-0.3**	—
			**Median**	**0.0**	—	**-1.6**	—	**0.0**	**0.0**	—	—	—	—	**0.0**	**0.0**	—	—	—	**0.0**	—	**0.0**	—	**0.0**	**0.0**	—
		**Percent change**	**Mean**	**-1.3**	—	**-7.2**	—	**-1.6**	**0.2**	—	—	—	—	**0.0**	**-0.2**	—	—	—	**-9.4**	—	**1.1**	—	**-0.2**	**-2.6**	—
			**Median**	**0.0**	—	**-10.8**	—	**0.0**	**0.0**	—	—	—	—	**0.0**	**0.0**	—	—	—	**0.0**	—	**0.0**	—	**0.0**	**0.0**	—
	**Free sugars (g per 100 g/mL)**	**Absolute change**	**Mean**	**-0.1**	—	**-1.7**	—	**0.0**	**0.0**	—	—	—	—	**0.0**	**-0.1**	—	—	—	**2.1**	—	**0.1**	—	**-0.1**	**-0.3**	—
			**Median**	**0.0**	—	**-1.6**	—	**0.0**	**0.0**	—	—	—	—	**0.0**	**0.0**	—	—	—	**0.0**	—	**0.0**	—	**0.0**	**0.0**	—
		**Percent change**	**Mean**	**-1.3**	—	**-7.2**	—	**-1.7**	**0.2**	—	—	—	—	**0.0**	**N/A**	—	—	—	**-2.7**	—	**1.1**	—	**0.9**	**-2.6**	—
			**Median**	**0.0**	—	**-10.8**	—	**0.0**	**0.0**	—	—	—	—	**0.0**	**0.0**	—	—	—	**-3.0**	—	**0.0**	—	**0.0**	**0.0**	—
Fruit juices, nectars and fruit drinks (J11)	Number of products			33	0	6	0	5	51	0	0	0	0	8	53	0	0	0	18	0	16	0	40	11	0
	HSR	Absolute change	Mean	-0.2	—	0.1	—	0.0	0.1	—	—	—	—	0.0	0.1	—	—	—	0.7	—	0.0	—	0.0	0.0	—
			Median	0.0	—	0.0	—	0.0	0.0	—	—	—	—	0.0	0.0	—	—	—	0.0	—	0.0	—	0.0	0.0	—
		Percent change (%)	Mean	-4.1	—	7.8	—	0.0	8.0	—	—	—	—	0.0	7.7	—	—	—	45.8	—	0.0	—	4.0	0.0	—
			Median	0.0	—	0.0	—	0.0	0.0	—	—	—	—	0.0	0.0	—	—	—	0.0	—	0.0	—	0.0	0.0	—
	Calories (kcal per 100 g/mL)	Absolute change	Mean	0.0	—	-6.0	—	-1.3	0.1	—	—	—	—	0.0	0.2	—	—	—	-3.2	—	0.3	—	0.1	-1.3	—
			Median	0.0	—	-2.0	—	0.0	0.0	—	—	—	—	0.0	0.0	—	—	—	-2.0	—	0.0	—	0.0	0.0	—
		Percent change (%)	Mean	0.0	—	-9.3	—	-4.7	0.2	—	—	—	—	0.0	0.6	—	—	—	-6.0	—	0.6	—	-0.4	-2.6	—
			Median	0.0	—	-3.6	—	0.0	0.0	—	—	—	—	0.0	0.0	—	—	—	-3.8	—	0.0	—	0.0	0.0	—
	Sodium (mg per 100 g/mL)	Absolute change	Mean	-1.8	—	-1.0	—	0.0	0.0	—	—	—	—	0.0	-0.2	—	—	—	-3.3	—	0.4	—	0.1	1.1	—
			Median	-2.0	—	0.0	—	0.0	0.0	—	—	—	—	0.0	0.0	—	—	—	0.0	—	0.0	—	0.0	0.0	—
		Percent change (%)	Mean	-13.2	—	-11.1	—	0.0	0.5	—	—	—	—	0.0	N/A	—	—	—	-17.1	—	N/A	—	7.0	4.2	—
			Median	-20.0	—	0.0	—	0.0	0.0	—	—	—	—	0.0	0.0	—	—	—	0.0	—	0.0	—	0.0	0.0	—
	Saturated fat (g per 100 g/mL)	Absolute change	Mean	0.0	—	0.0	—	0.0	0.0	—	—	—	—	0.0	0.0	—	—	—	0.0	—	0.0	—	0.0	0.0	—
			Median	0.0	—	0.0	—	0.0	0.0	—	—	—	—	0.0	0.0	—	—	—	0.0	—	0.0	—	0.0	0.0	—
		Percent change (%)	Mean	N/A	—	N/A	—	N/A	N/A	—	—	—	—	N/A	N/A	—	—	—	N/A	—	N/A	—	N/A	N/A	—
			Median	N/A	—	-100.0	—	N/A	N/A	—	—	—	—	N/A	N/A	—	—	—	N/A	—	N/A	—	N/A	N/A	—
	Trans fat (g per 100 g/mL)	Absolute change	Mean	0.0	—	0.0	—	0.0	0.0	—	—	—	—	0.0	0.0	—	—	—	0.0	—	0.0	—	0.0	0.0	—
			Median	0.0	—	0.0	—	0.0	0.0	—	—	—	—	0.0	0.0	—	—	—	0.0	—	0.0	—	0.0	0.0	—
		Percent change (%)	Mean	N/A	—	N/A	—	N/A	N/A	—	—	—	—	N/A	N/A	—	—	—	N/A	—	N/A	—	N/A	N/A	—
			Median	N/A	—	N/A	—	N/A	N/A	—	—	—	—	N/A	N/A	—	—	—	N/A	—	N/A	—	N/A	N/A	—
	Total sugars (g per 100 g/mL)	Absolute change	Mean	-0.1	—	-1.7	—	-0.4	0.0	—	—	—	—	0.0	-0.1	—	—	—	-0.9	—	0.1	—	-0.3	-0.3	—
			Median	0.0	—	-1.6	—	0.0	0.0	—	—	—	—	0.0	0.0	—	—	—	-0.8	—	0.0	—	0.0	0.0	—
		Percent change (%)	Mean	-1.3	—	-7.2	—	-5.9	0.2	—	—	—	—	0.0	-1.2	—	—	—	-14.5	—	1.1	—	-2.2	-2.6	—
			Median	0.0	—	-10.8	—	0.0	0.0	—	—	—	—	0.0	0.0	—	—	—	-8.5	—	0.0	—	0.0	0.0	—
	Free sugars (g per 100 g/mL)	Absolute change	Mean	-0.1	—	-1.7	—	-0.4	0.0	—	—	—	—	0.0	-0.1	—	—	—	-0.9	—	0.1	—	-0.3	-0.3	—
			Median	0.0	—	-1.6	—	0.0	0.0	—	—	—	—	0.0	0.0	—	—	—	-0.8	—	0.0	—	0.0	0.0	—
		Percent change (%)	Mean	-1.3	—	-7.2	—	-5.9	0.2	—	—	—	—	0.0	-1.2	—	—	—	-14.5	—	1.1	—	-2.2	-2.6	—
			Median	0.0	—	-10.8	—	0.0	0.0	—	—	—	—	0.0	0.0	—	—	—	-8.5	—	0.0	—	0.0	0.0	—
Fruit, frozen or canned, coated or uncoated (J1, J2, J3, J4, J5)	Number of products			0	0	0	0	0	0	0	0	0	0	0	25	0	0	0	0	0	0	0	20	0	0
	HSR	Absolute change	Mean	—	—	—	—	—	—	—	—	—	—	—	0.0	—	—	—	—	—	—	—	0.0	—	—
			Median	—	—	—	—	—	—	—	—	—	—	—	0.0	—	—	—	—	—	—	—	0.0	—	—
		Percent change (%)	Mean	—	—	—	—	—	—	—	—	—	—	—	0.5	—	—	—	—	—	—	—	0.1	—	—
			Median	—	—	—	—	—	—	—	—	—	—	—	0.0	—	—	—	—	—	—	—	0.0	—	—
	Calories (kcal per 100 g/mL)	Absolute change	Mean	—	—	—	—	—	—	—	—	—	—	—	-3.4	—	—	—	—	—	—	—	-0.7	—	—
			Median	—	—	—	—	—	—	—	—	—	—	—	0.0	—	—	—	—	—	—	—	0.0	—	—
		Percent change (%)	Mean	—	—	—	—	—	—	—	—	—	—	—	-5.5	—	—	—	—	—	—	—	-1.2	—	—
			Median	—	—	—	—	—	—	—	—	—	—	—	0.0	—	—	—	—	—	—	—	0.0	—	—
	Sodium (mg per 100 g/mL)	Absolute change	Mean	—	—	—	—	—	—	—	—	—	—	—	0.1	—	—	—	—	—	—	—	-2.7	—	—
			Median	—	—	—	—	—	—	—	—	—	—	—	0.0	—	—	—	—	—	—	—	0.0	—	—
		Percent change (%)	Mean	—	—	—	—	—	—	—	—	—	—	—	4.6	—	—	—	—	—	—	—	-39.6	—	—
			Median	—	—	—	—	—	—	—	—	—	—	—	0.0	—	—	—	—	—	—	—	-50.0	—	—
	Saturated fat (g per 100 g/mL)	Absolute change	Mean	—	—	—	—	—	—	—	—	—	—	—	0.0	—	—	—	—	—	—	—	0.0	—	—
			Median	—	—	—	—	—	—	—	—	—	—	—	0.0	—	—	—	—	—	—	—	0.0	—	—
		Percent change (%)	Mean	—	—	—	—	—	—	—	—	—	—	—	0.0	—	—	—	—	—	—	—	N/A	—	—
			Median	—	—	—	—	—	—	—	—	—	—	—	0.0	—	—	—	—	—	—	—	N/A	—	—
	Trans fat (g per 100 g/mL)	Absolute change	Mean	—	—	—	—	—	—	—	—	—	—	—	0.0	—	—	—	—	—	—	—	0.0	—	—
			Median	—	—	—	—	—	—	—	—	—	—	—	0.0	—	—	—	—	—	—	—	0.0	—	—
		Percent change (%)	Mean	—	—	—	—	—	—	—	—	—	—	—	N/A	—	—	—	—	—	—	—	N/A	—	—
			Median	—	—	—	—	—	—	—	—	—	—	—	N/A	—	—	—	—	—	—	—	N/A	—	—
	Total sugars (g per 100 g/mL)	Absolute change	Mean	—	—	—	—	—	—	—	—	—	—	—	-0.1	—	—	—	—	—	—	—	-0.1	—	—
			Median	—	—	—	—	—	—	—	—	—	—	—	0.0	—	—	—	—	—	—	—	0.0	—	—
		Percent change (%)	Mean	—	—	—	—	—	—	—	—	—	—	—	0.1	—	—	—	—	—	—	—	-0.4	—	—
			Median	—	—	—	—	—	—	—	—	—	—	—	0.0	—	—	—	—	—	—	—	0.0	—	—
	Free sugars (g per 100 g/mL)	Absolute change	Mean	—	—	—	—	—	—	—	—	—	—	—	0.1	—	—	—	—	—	—	—	0.2	—	—
			Median	—	—	—	—	—	—	—	—	—	—	—	0.0	—	—	—	—	—	—	—	0.0	—	—
		Percent change (%)	Mean	—	—	—	—	—	—	—	—	—	—	—	8.7	—	—	—	—	—	—	—	7.2	—	—
			Median	—	—	—	—	—	—	—	—	—	—	—	-1.8	—	—	—	—	—	—	—	-2.5	—	—
Other, e.g., apple sauce, dried or	Number of products			0	0	0	0	13	0	0	0	0	0	0	31	0	0	0	5	0	0	0	24	0	0
	HSR	Absolute change	Mean	—	—	—	—	-0.2	—	—	—	—	—	—	0.0	—	—	—	0.2	—	—	—	-0.1	—	—
			Median	—	—	—	—	0.0	—	—	—	—	—	—	0.0	—	—	—	0.0	—	—	—	0.0	—	—

				A. Lassonde	Agropur	Campbell Soup	Canada Bread	Canada Dry Mott's	Coca-Cola	Danone	General Mills	George Weston	Kellogg	Kraft Heinz	Loblaw	Maple Leaf Foods	Mondelēz	Nestlé	Ocean Spray	Parmalat	PepsiCo	Saputo	Sobeys	Sun-Rype	Unilever
candied fruit,		Percent	Mean	—	—	—	—	-3.8	—	—	—	—	—	—	-1.2	—	—	—	5.7	—	—	—	-3.0	—	—
fruit relishes		change (%)	Median	—	—	—	—	0.0	—	—	—	—	—	—	0.0	—	—	—	0.0	—	—	—	0.0	—	—
(J6, J7, J8, J9,	Calories	Absolute	Mean	—	—	—	—	-0.6	—	—	—	—	—	—	-0.2	—	—	—	-15.0	—	—	—	1.3	—	—
J10, J12)	(kcal per	change	Median	—	—	—	—	0.0	—	—	—	—	—	—	0.0	—	—	—	-25.0	—	—	—	0.0	—	—
	100 g/mL)	Percent	Mean	—	—	—	—	-0.8	—	—	—	—	—	—	-0.5	—	—	—	-4.3	—	—	—	0.7	—	—
		change (%)	Median	—	—	—	—	0.0	—	—	—	—	—	—	0.0	—	—	—	-7.1	—	—	—	0.0	—	—
	Sodium	Absolute	Mean	—	—	—	—	0.0	—	—	—	—	—	—	-4.4	—	—	—	0.0	—	—	—	1.0	—	—
	(mg per	change	Median	—	—	—	—	0.0	—	—	—	—	—	—	0.0	—	—	—	0.0	—	—	—	0.0	—	—
	100 g/mL)	Percent	Mean	—	—	—	—	0.0	—	—	—	—	—	—	-29.4	—	—	—	N/A	—	—	—	-20.0	—	—
		change (%)	Median	—	—	—	—	0.0	—	—	—	—	—	—	0.0	—	—	—	N/A	—	—	—	0.0	—	—
	Saturated	Absolute	Mean	—	—	—	—	0.0	—	—	—	—	—	—	0.0	—	—	—	0.0	—	—	—	0.0	—	—
	fat (g per	change	Median	—	—	—	—	0.0	—	—	—	—	—	—	0.0	—	—	—	0.0	—	—	—	0.0	—	—
	100 g/mL)	Percent	Mean	—	—	—	—	N/A	—	—	—	—	—	—	N/A	—	—	—	N/A	—	—	—	N/A	—	—
		change (%)	Median	—	—	—	—	N/A	—	—	—	—	—	—	N/A	—	—	—	N/A	—	—	—	N/A	—	—
	Trans fat (g	Absolute	Mean	—	—	—	—	0.0	—	—	—	—	—	—	0.0	—	—	—	0.0	—	—	—	0.0	—	—
	per 100	change	Median	—	—	—	—	0.0	—	—	—	—	—	—	0.0	—	—	—	0.0	—	—	—	0.0	—	—
	g/mL)	Percent	Mean	—	—	—	—	N/A	—	—	—	—	—	—	N/A	—	—	—	N/A	—	—	—	N/A	—	—
		change (%)	Median	—	—	—	—	N/A	—	—	—	—	—	—	N/A	—	—	—	N/A	—	—	—	N/A	—	—
	Total	Absolute	Mean	—	—	—	—	0.1	—	—	—	—	—	—	0.7	—	—	—	6.0	—	—	—	1.4	—	—
	sugars (g	change	Median	—	—	—	—	0.0	—	—	—	—	—	—	0.0	—	—	—	7.5	—	—	—	0.0	—	—
	per 100	Percent	Mean	—	—	—	—	0.3	—	—	—	—	—	—	1.4	—	—	—	9.2	—	—	—	3.5	—	—
	g/mL)	change (%)	Median	—	—	—	—	0.0	—	—	—	—	—	—	0.0	—	—	—	11.5	—	—	—	0.0	—	—
	Free sugars	Absolute	Mean	—	—	—	—	0.1	—	—	—	—	—	—	-0.3	—	—	—	13.0	—	—	—	0.1	—	—
	(g per 100	change	Median	—	—	—	—	0.0	—	—	—	—	—	—	0.0	—	—	—	16.2	—	—	—	0.0	—	—
	g/mL)	Percent	Mean	—	—	—	—	9.0	—	—	—	—	—	—	-0.1	—	—	—	50.7	—	—	—	5.5	—	—
		change (%)	Median	—	—	—	—	9.0	—	—	—	—	—	—	1.5	—	—	—	50.7	—	—	—	6.1	—	—
**Legumes,**	**Number of products**			**0**	**0**	**0**	**0**	**0**	**0**	**0**	**0**	**0**	**0**	**0**	**34**	**0**	**0**	**0**	**0**	**0**	**0**	**0**	**12**	**0**	**0**
**includes tofu**	**HSR**	**Absolute**	**Mean**	—	—	—	—	—	—	—	—	—	—	—	**0.0**	—	—	—	—	—	—	—	**0.1**	—	—
**and tempeh**		**change**	**Median**	—	—	—	—	—	—	—	—	—	—	—	**0.0**	—	—	—	—	—	—	—	**0.0**	—	—
**(K)**		**Percent**	**Mean**	—	—	—	—	—	—	—	—	—	—	—	**-0.6**	—	—	—	—	—	—	—	**1.9**	—	—
		**change**	**Median**	—	—	—	—	—	—	—	—	—	—	—	**0.0**	—	—	—	—	—	—	—	**0.0**	—	—
	**Calories**	**Absolute**	**Mean**	—	—	—	—	—	—	—	—	—	—	—	**-10.4**	—	—	—	—	—	—	—	**-4.7**	—	—
	**(kcal per**	**change**	**Median**	—	—	—	—	—	—	—	—	—	—	—	**-2.3**	—	—	—	—	—	—	—	**-2.0**	—	—
	**100 g/mL)**	**Percent**	**Mean**	—	—	—	—	—	—	—	—	—	—	—	**-6.4**	—	—	—	—	—	—	—	**-5.3**	—	—
		**change**	**Median**	—	—	—	—	—	—	—	—	—	—	—	**-2.4**	—	—	—	—	—	—	—	**-2.1**	—	—
	**Sodium**	**Absolute**	**Mean**	—	—	—	—	—	—	—	—	—	—	—	**-9.0**	—	—	—	—	—	—	—	**-22.7**	—	—
	**(mg per**	**change**	**Median**	—	—	—	—	—	—	—	—	—	—	—	**0.0**	—	—	—	—	—	—	—	**0.0**	—	—
	**100 g/mL)**	**Percent**	**Mean**	—	—	—	—	—	—	—	—	—	—	—	**-16.3**	—	—	—	—	—	—	—	**-24.9**	—	—
		**change**	**Median**	—	—	—	—	—	—	—	—	—	—	—	**0.0**	—	—	—	—	—	—	—	**-30.8**	—	—
	**Saturated**	**Absolute**	**Mean**	—	—	—	—	—	—	—	—	—	—	—	**0.0**	—	—	—	—	—	—	—	**0.0**	—	—
	**fat (g per**	**change**	**Median**	—	—	—	—	—	—	—	—	—	—	—	**0.0**	—	—	—	—	—	—	—	**0.0**	—	—
	**100 g/mL)**	**Percent**	**Mean**	—	—	—	—	—	—	—	—	—	—	—	**-10.9**	—	—	—	—	—	—	—	**7.5**	—	—
		**change**	**Median**	—	—	—	—	—	—	—	—	—	—	—	**0.0**	—	—	—	—	—	—	—	**0.0**	—	—
	**Trans fat**	**Absolute**	**Mean**	—	—	—	—	—	—	—	—	—	—	—	**0.0**	—	—	—	—	—	—	—	**0.0**	—	—
	**(g per 100**	**change**	**Median**	—	—	—	—	—	—	—	—	—	—	—	**0.0**	—	—	—	—	—	—	—	**0.0**	—	—
	**g/mL)**	**Percent**	**Mean**	—	—	—	—	—	—	—	—	—	—	—	**N/A**	—	—	—	—	—	—	—	**N/A**	—	—
		**change**	**Median**	—	—	—	—	—	—	—	—	—	—	—	**N/A**	—	—	—	—	—	—	—	**N/A**	—	—
	**Total**	**Absolute**	**Mean**	—	—	—	—	—	—	—	—	—	—	—	**0.1**	—	—	—	—	—	—	—	**0.0**	—	—
	**sugars (g**	**change**	**Median**	—	—	—	—	—	—	—	—	—	—	—	**0.0**	—	—	—	—	—	—	—	**0.0**	—	—
	**per 100**	**Percent**	**Mean**	—	—	—	—	—	—	—	—	—	—	—	**0.7**	—	—	—	—	—	—	—	**-4.5**	—	—
	**g/mL)**	**change**	**Median**	—	—	—	—	—	—	—	—	—	—	—	**0.0**	—	—	—	—	—	—	—	**0.0**	—	—
	**Free**	**Absolute**	**Mean**	—	—	—	—	—	—	—	—	—	—	—	**0.0**	—	—	—	—	—	—	—	**0.0**	—	—
	**sugars (g**	**change**	**Median**	—	—	—	—	—	—	—	—	—	—	—	**0.0**	—	—	—	—	—	—	—	**0.0**	—	—
	**per 100**	**Percent**	**Mean**	—	—	—	—	—	—	—	—	—	—	—	**N/A**	—	—	—	—	—	—	—	**N/A**	—	—
	**g/mL)**	**change**	**Median**	—	—	—	—	—	—	—	—	—	—	—	**N/A**	—	—	—	—	—	—	—	**N/A**	—	—
**Meat, poultry,**	**Number of products**			**0**	**0**	**0**	**0**	**0**	**0**	**0**	**0**	**0**	**0**	**0**	**77**	**68**	**0**	**0**	**0**	**0**	**0**	**0**	**28**	**0**	**0**
**their products**	**HSR**	**Absolute**	**Mean**	—	—	—	—	—	—	—	—	—	—	—	**0.1**	**0.1**	—	—	—	—	—	—	**0.1**	—	—
**and**		**change**	**Median**	—	—	—	—	—	—	—	—	—	—	—	**0.0**	**0.0**	—	—	—	—	—	—	**0.0**	—	—
**substitutes (L)**		**Percent**	**Mean**	—	—	—	—	—	—	—	—	—	—	—	**6.0**	**11.9**	—	—	—	—	—	—	**4.6**	—	—
		**change**	**Median**	—	—	—	—	—	—	—	—	—	—	—	**0.0**	**0.0**	—	—	—	—	—	—	**0.0**	—	—
	**Calories**	**Absolute**	**Mean**	—	—	—	—	—	—	—	—	—	—	—	**1.0**	**-3.5**	—	—	—	—	—	—	**-1.9**	—	—
	**(kcal per**	**change**	**Median**	—	—	—	—	—	—	—	—	—	—	—	**0.0**	**0.0**	—	—	—	—	—	—	**0.0**	—	—
	**100 g/mL)**	**Percent**	**Mean**	—	—	—	—	—	—	—	—	—	—	—	**0.7**	**-1.4**	—	—	—	—	—	—	**-0.3**	—	—
		**change**	**Median**	—	—	—	—	—	—	—	—	—	—	—	**0.0**	**0.0**	—	—	—	—	—	—	**0.0**	—	—
	**Sodium**	**Absolute**	**Mean**	—	—	—	—	—	—	—	—	—	—	—	**-33.9**	**-55.2**	—	—	—	—	—	—	**-74.3**	—	—
	**(mg per**	**change**	**Median**	—	—	—	—	—	—	—	—	—	—	—	**0.0**	**0.0**	—	—	—	—	—	—	**-17.6**	—	—
	**100 g/mL)**	**Percent**	**Mean**	—	—	—	—	—	—	—	—	—	—	—	**-4.4**	**-6.3**	—	—	—	—	—	—	**-8.5**	—	—
		**change**	**Median**	—	—	—	—	—	—	—	—	—	—	—	**0.0**	**0.0**	—	—	—	—	—	—	**-2.8**	—	—
	**Saturated**	**Absolute**	**Mean**	—	—	—	—	—	—	—	—	—	—	—	**-0.1**	**-0.1**	—	—	—	—	—	—	**0.1**	—	—
	**fat (g per**	**change**	**Median**	—	—	—	—	—	—	—	—	—	—	—	**0.0**	**0.0**	—	—	—	—	—	—	**0.0**	—	—
	**100 g/mL)**	**Percent**	**Mean**	—	—	—	—	—	—	—	—	—	—	—	**3.2**	**-8.7**	—	—	—	—	—	—	**1.3**	—	—
		**change**	**Median**	—	—	—	—	—	—	—	—	—	—	—	**0.0**	**0.0**	—	—	—	—	—	—	**0.0**	—	—
	**Trans fat**	**Absolute**	**Mean**	—	—	—	—	—	—	—	—	—	—	—	**0.0**	**0.0**	—	—	—	—	—	—	**0.0**	—	—

				A. Lassonde	Agropur	Campbell Soup	Canada Bread	Canada Dry Mott's	Coca-Cola	Danone	General Mills	George Weston	Kellogg	Kraft Heinz	Loblaw	Maple Leaf Foods	Mondelēz	Nestlé	Ocean Spray	Parmalat	PepsiCo	Saputo	Sobeys	Sun-Rype	Unilever
	(g per 100	change	Median	—	—	—	—	—	—	—	—	—	—	—	0.0	0.0	—	—	—	—	—	—	0.0	—	—
	g/mL)	Percent	Mean	—	—	—	—	—	—	—	—	—	—	—	-14.4	-12.1	—	—	—	—	—	—	9.0	—	—
		change	Median	—	—	—	—	—	—	—	—	—	—	—	0.0	0.0	—	—	—	—	—	—	0.0	—	—
	Total	Absolute	Mean	—	—	—	—	—	—	—	—	—	—	—	0.1	-0.2	—	—	—	—	—	—	-0.3	—	—
	sugars (g	change	Median	—	—	—	—	—	—	—	—	—	—	—	0.0	0.0	—	—	—	—	—	—	0.0	—	—
	per 100	Percent	Mean	—	—	—	—	—	—	—	—	—	—	—	-0.6	-25.5	—	—	—	—	—	—	-30.6	—	—
	g/mL)	change	Median	—	—	—	—	—	—	—	—	—	—	—	0.0	0.0	—	—	—	—	—	—	0.0	—	—
	Free	Absolute	Mean	—	—	—	—	—	—	—	—	—	—	—	0.2	-0.3	—	—	—	—	—	—	-0.2	—	—
	sugars (g	change	Median	—	—	—	—	—	—	—	—	—	—	—	0.0	0.0	—	—	—	—	—	—	0.0	—	—
	per 100	Percent	Mean	—	—	—	—	—	—	—	—	—	—	—	26.1	-28.2	—	—	—	—	—	—	-25.3	—	—
	g/mL)	change	Median	—	—	—	—	—	—	—	—	—	—	—	0.0	-0.2	—	—	—	—	—	—	0.0	—	—
Miscellaneous	Number of products			0	0	0	0	1	0	0	23	0	1	9	40	1	2	3	0	0	16	0	18	0	1
(M)	HSR	Absolute	Mean	—	—	—	—	0.0	—	—	0.0	—	0.5	0.2	0.1	0.0	0.3	0.0	—	—	-0.1	—	0.1	—	0.0
		change	Median	—	—	—	—	0.0	—	—	0.0	—	0.5	0.0	0.0	0.0	0.3	0.0	—	—	0.0	—	0.0	—	0.0
		Percent	Mean	—	—	—	—	0.0	—	—	-2.0	—	25.0	38.9	16.3	0.0	12.5	0.0	—	—	-2.6	—	10.2	—	0.0
		change	Median	—	—	—	—	0.0	—	—	0.0	—	25.0	0.0	0.0	0.0	12.5	0.0	—	—	0.0	—	0.0	—	0.0
	Calories	Absolute	Mean	—	—	—	—	100.0	—	—	3.7	—	13.2	-5.5	0.4	0.0	-29.4	0.0	—	—	3.3	—	1.4	—	39.6
	(kcal per	change	Median	—	—	—	—	100.0	—	—	0.0	—	13.2	0.0	0.0	0.0	-29.4	0.0	—	—	0.0	—	0.0	—	39.6
	100 g/mL)	Percent	Mean	—	—	—	—	N/A	—	—	1.5	—	3.7	-1.0	0.4	0.0	-6.3	0.0	—	—	0.8	—	0.6	—	10.9
		change	Median	—	—	—	—	N/A	—	—	0.0	—	3.7	0.0	0.0	0.0	-6.3	0.0	—	—	0.0	—	0.0	—	10.9
	Sodium	Absolute	Mean	—	—	—	—	-2000.0	—	—	-216.5	—	-13.2	-36.4	-316.5	0.0	-14.7	0.0	—	—	-1.0	—	-153.4	—	-979.5
	(mg per	change	Median	—	—	—	—	-2000.0	—	—	-10.0	—	-13.2	0.0	0.0	0.0	-14.7	0.0	—	—	0.0	—	0.0	—	-979.5
	100 g/mL)	Percent	Mean	—	—	—	—	-6.7	—	—	-7.6	—	-2.1	-2.4	-5.7	0.0	-1.6	0.0	—	—	0.3	—	-13.6	—	-16.8
		change	Median	—	—	—	—	-6.7	—	—	-2.5	—	-2.1	0.0	0.0	0.0	-1.6	0.0	—	—	0.0	—	0.0	—	-16.8
	Saturated	Absolute	Mean	—	—	—	—	0.0	—	—	0.2	—	0.0	-1.1	0.1	6.3	-1.8	0.0	—	—	0.1	—	-0.1	—	1.4
	fat (g per	change	Median	—	—	—	—	0.0	—	—	0.0	—	0.0	0.0	0.0	6.3	-1.8	0.0	—	—	0.0	—	0.0	—	1.4
	100 g/mL)	Percent	Mean	—	—	—	—	N/A	—	—	10.8	—	N/A	-62.4	N/A	100.0	-80.0	0.0	—	—	10.1	—	27.8	—	77.4
		change	Median	—	—	—	—	N/A	—	—	5.2	—	N/A	-100.0	0.0	100.0	-80.0	0.0	—	—	0.0	—	0.0	—	77.4
	Trans fat	Absolute	Mean	—	—	—	—	0.0	—	—	-0.3	—	0.0	0.0	0.0	0.0	0.0	0.0	—	—	0.0	—	0.0	—	0.0
	(g per 100	change	Median	—	—	—	—	0.0	—	—	0.0	—	0.0	0.0	0.0	0.0	0.0	0.0	—	—	0.0	—	0.0	—	0.0
	g/mL)	Percent	Mean	—	—	—	—	N/A	—	—	N/A	—	N/A	-100.0	N/A	N/A	N/A	N/A	—	—	0.0	—	N/A	—	N/A
		change	Median	—	—	—	—	N/A	—	—	-100.0	—	N/A	-100.0	N/A	N/A	N/A	N/A	—	—	0.0	—	N/A	—	N/A
	Total	Absolute	Mean	—	—	—	—	0.0	—	—	0.5	—	0.4	-1.2	-0.2	0.0	0.0	0.0	—	—	0.7	—	0.3	—	14.1
	sugars (g	change	Median	—	—	—	—	0.0	—	—	0.0	—	0.4	0.0	0.0	0.0	0.0	0.0	—	—	0.0	—	0.0	—	14.1
	per 100	Percent	Mean	—	—	—	—	N/A	—	—	5.5	—	3.7	-26.9	-2.1	N/A	0.0	0.0	—	—	2.5	—	3.3	—	77.4
	g/mL)	change	Median	—	—	—	—	N/A	—	—	0.0	—	3.7	-3.8	0.0	N/A	0.0	0.0	—	—	0.0	—	0.6	—	77.4
	Free	Absolute	Mean	—	—	—	—	0.0	—	—	0.7	—	0.1	-0.7	0.0	0.0	0.0	0.2	—	—	1.4	—	0.4	—	13.3
	sugars (g	change	Median	—	—	—	—	0.0	—	—	0.4	—	0.1	0.0	0.0	0.0	0.0	0.2	—	—	0.4	—	0.2	—	13.3
	per 100	Percent	Mean	—	—	—	—	N/A	—	—	4.2	—	1.2	-23.6	N/A	N/A	0.0	1.6	—	—	N/A	—	N/A	—	73.3
	g/mL)	change	Median	—	—	—	—	N/A	—	—	2.9	—	1.2	-1.2	0.6	N/A	0.0	1.8	—	—	3.0	—	2.3	—	73.3
Baking/cooking	Number of products			0	0	0	0	0	0	0	16	0	1	9	30	1	2	0	0	0	16	0	15	0	0
ingredients,	HSR	Absolute	Mean	—	—	—	—	—	—	—	0.0	—	0.5	0.2	0.1	0.0	0.3	—	—	—	-0.1	—	0.1	—	—
e.g., baking		change	Median	—	—	—	—	—	—	—	0.0	—	0.5	0.0	0.0	0.0	0.3	—	—	—	0.0	—	0.0	—	—
powder, yeast,		Percent	Mean	—	—	—	—	—	—	—	1.3	—	25.0	38.9	15.0	0.0	12.5	—	—	—	-2.6	—	12.2	—	—
sprinkles, bread		change (%)	Median	—	—	—	—	—	—	—	0.0	—	25.0	0.0	0.0	0.0	12.5	—	—	—	0.0	—	0.0	—	—
crumbs, cocoa	Calories	Absolute	Mean	—	—	—	—	—	—	—	1.8	—	13.2	-5.5	1.4	0.0	-29.4	—	—	—	3.3	—	1.6	—	—
powder, etc.	(kcal per	change	Median	—	—	—	—	—	—	—	0.0	—	13.2	0.0	0.0	0.0	-29.4	—	—	—	0.0	—	0.0	—	—
(M1, M3, M5,	100 g/mL)	Percent	Mean	—	—	—	—	—	—	—	0.9	—	3.7	-1.0	0.9	0.0	-6.3	—	—	—	0.8	—	0.8	—	—
M8, M11,		change (%)	Median	—	—	—	—	—	—	—	0.0	—	3.7	0.0	0.0	0.0	-6.3	—	—	—	0.0	—	0.0	—	—
M12)	Sodium	Absolute	Mean	—	—	—	—	—	—	—	-25.9	—	-13.2	-36.4	-29.2	0.0	-14.7	—	—	—	-1.0	—	-184.1	—	—
	(mg per	change	Median	—	—	—	—	—	—	—	-9.2	—	-13.2	0.0	0.0	0.0	-14.7	—	—	—	0.0	—	-203.0	—	—
	100 g/mL)	Percent	Mean	—	—	—	—	—	—	—	-6.9	—	-2.1	-2.4	-5.5	0.0	-1.6	—	—	—	0.3	—	-16.3	—	—
		change (%)	Median	—	—	—	—	—	—	—	-2.2	—	-2.1	0.0	0.0	0.0	-1.6	—	—	—	0.0	—	-12.8	—	—
	Saturated	Absolute	Mean	—	—	—	—	—	—	—	0.3	—	0.0	-1.1	0.1	6.3	-1.8	—	—	—	0.1	—	-0.1	—	—
	fat (g per	change	Median	—	—	—	—	—	—	—	0.1	—	0.0	0.0	0.0	6.3	-1.8	—	—	—	0.0	—	0.0	—	—
	100 g/mL)	Percent	Mean	—	—	—	—	—	—	—	10.8	—	N/A	-62.4	N/A	100.0	-80.0	—	—	—	10.1	—	29.6	—	—
		change (%)	Median	—	—	—	—	—	—	—	5.2	—	N/A	-100.0	0.0	100.0	-80.0	—	—	—	0.0	—	0.0	—	—
	Trans fat (g	Absolute	Mean	—	—	—	—	—	—	—	-0.4	—	0.0	0.0	0.0	0.0	0.0	—	—	—	0.0	—	0.0	—	—
	per 100	change	Median	—	—	—	—	—	—	—	0.0	—	0.0	0.0	0.0	0.0	0.0	—	—	—	0.0	—	0.0	—	—
	g/mL)	Percent	Mean	—	—	—	—	—	—	—	N/A	—	N/A	-100.0	N/A	N/A	N/A	—	—	—	0.0	—	N/A	—	—
		change (%)	Median	—	—	—	—	—	—	—	-100.0	—	N/A	-100.0	N/A	N/A	N/A	—	—	—	0.0	—	N/A	—	—
	Total	Absolute	Mean	—	—	—	—	—	—	—	0.0	—	0.4	-1.2	-0.3	0.0	0.0	—	—	—	0.7	—	0.4	—	—
	sugars (g	change	Median	—	—	—	—	—	—	—	0.0	—	0.4	0.0	0.0	0.0	0.0	—	—	—	0.0	—	0.1	—	—
	per 100	Percent	Mean	—	—	—	—	—	—	—	-0.1	—	3.7	-26.9	-2.1	N/A	0.0	—	—	—	2.5	—	4.1	—	—
	g/mL)	change (%)	Median	—	—	—	—	—	—	—	0.0	—	3.7	-3.8	0.0	N/A	0.0	—	—	—	0.0	—	1.3	—	—
	Free sugars	Absolute	Mean	—	—	—	—	—	—	—	0.2	—	0.1	-0.7	0.0	0.0	0.0	—	—	—	1.4	—	0.6	—	—
	(g per 100	change	Median	—	—	—	—	—	—	—	0.5	—	0.1	0.0	0.0	0.0	0.0	—	—	—	0.4	—	0.2	—	—
	g/mL)	Percent	Mean	—	—	—	—	—	—	—	-3.6	—	1.2	-23.6	N/A	N/A	0.0	—	—	—	N/A	—	N/A	—	—
		change (%)	Median	—	—	—	—	—	—	—	2.6	—	1.2	-1.2	0.6	N/A	0.0	—	—	—	3.0	—	4.3	—	—
Seasoning salts	Number of products			0	0	0	0	1	0	0	7	0	0	0	10	0	0	3	0	0	0	0	3	0	1
and mixes (M9,	HSR	Absolute	Mean	—	—	—	—	0.0	—	—	-0.1	—	—	—	0.1	—	—	0.0	—	—	—	—	0.0	—	0.0
M10)		change	Median	—	—	—	—	0.0	—	—	0.0	—	—	—	0.0	—	—	0.0	—	—	—	—	0.0	—	0.0
		Percent	Mean	—	—	—	—	0.0	—	—	-9.5	—	—	—	20.0	—	—	0.0	—	—	—	—	0.0	—	0.0

				A. Lassonde	Agropur	Campbell Soup	Canada Bread	Canada Dry Mott's	Coca-Cola	Danone	General Mills	George Weston	Kellogg	Kraft Heinz	Loblaw	Maple Leaf Foods	Mondelēz	Nestlé	Ocean Spray	Parmalat	PepsiCo	Saputo	Sobeys	Sun-Rype	Unilever
		change (%)	Median	—	—	—	—	0.0	—	—	0.0	—	—	—	0.0	—	—	0.0	—	—	—	—	0.0	—	0.0
	Calories (kcal per 100 g/mL)	Absolute change	Mean	—	—	—	—	100.0	—	—	7.9	—	—	—	-2.9	—	—	0.0	—	—	—	—	0.0	—	39.6
			Median	—	—	—	—	100.0	—	—	0.0	—	—	—	0.0	—	—	0.0	—	—	—	—	0.0	—	39.6
		Percent change (%)	Mean	—	—	—	—	N/A	—	—	2.9	—	—	—	-1.4	—	—	0.0	—	—	—	—	0.0	—	10.9
			Median	—	—	—	—	N/A	—	—	0.0	—	—	—	0.0	—	—	0.0	—	—	—	—	0.0	—	10.9
	Sodium (mg per 100 g/mL)	Absolute change	Mean	—	—	—	—	-2000.0	—	—	-652.0	—	—	—	-1178.3	—	—	0.0	—	—	—	—	0.0	—	-979.5
			Median	—	—	—	—	-2000.0	—	—	-388.9	—	—	—	0.0	—	—	0.0	—	—	—	—	0.0	—	-979.5
		Percent change (%)	Mean	—	—	—	—	-6.7	—	—	-9.3	—	—	—	-6.6	—	—	0.0	—	—	—	—	0.0	—	-16.8
			Median	—	—	—	—	-6.7	—	—	-10.0	—	—	—	0.0	—	—	0.0	—	—	—	—	0.0	—	-16.8
	Saturated fat (g per 100 g/mL)	Absolute change	Mean	—	—	—	—	0.0	—	—	0.0	—	—	—	0.0	—	—	0.0	—	—	—	—	0.0	—	1.4
			Median	—	—	—	—	0.0	—	—	0.0	—	—	—	0.0	—	—	0.0	—	—	—	—	0.0	—	1.4
		Percent change (%)	Mean	—	—	—	—	N/A	—	—	N/A	—	—	—	0.0	—	—	0.0	—	—	—	—	0.0	—	77.4
			Median	—	—	—	—	N/A	—	—	N/A	—	—	—	0.0	—	—	0.0	—	—	—	—	0.0	—	77.4
	Trans fat (g per 100 g/mL)	Absolute change	Mean	—	—	—	—	0.0	—	—	0.0	—	—	—	0.0	—	—	0.0	—	—	—	—	0.0	—	0.0
			Median	—	—	—	—	0.0	—	—	0.0	—	—	—	0.0	—	—	0.0	—	—	—	—	0.0	—	0.0
		Percent change (%)	Mean	—	—	—	—	N/A	—	—	N/A	—	—	—	N/A	—	—	N/A	—	—	—	—	N/A	—	N/A
			Median	—	—	—	—	N/A	—	—	N/A	—	—	—	N/A	—	—	N/A	—	—	—	—	N/A	—	N/A
	Total sugars (g per 100 g/mL)	Absolute change	Mean	—	—	—	—	0.0	—	—	1.6	—	—	—	0.0	—	—	0.0	—	—	—	—	0.0	—	14.1
			Median	—	—	—	—	0.0	—	—	0.0	—	—	—	0.0	—	—	0.0	—	—	—	—	0.0	—	14.1
		Percent change (%)	Mean	—	—	—	—	N/A	—	—	50.0	—	—	—	N/A	—	—	0.0	—	—	—	—	0.0	—	77.4
			Median	—	—	—	—	N/A	—	—	50.0	—	—	—	N/A	—	—	0.0	—	—	—	—	0.0	—	77.4
	Free sugars (g per 100 g/mL)	Absolute change	Mean	—	—	—	—	0.0	—	—	1.9	—	—	—	0.0	—	—	0.2	—	—	—	—	-0.6	—	13.3
			Median	—	—	—	—	0.0	—	—	0.0	—	—	—	0.0	—	—	0.2	—	—	—	—	-0.9	—	13.3
		Percent change (%)	Mean	—	—	—	—	N/A	—	—	66.7	—	—	—	N/A	—	—	1.6	—	—	—	—	-4.3	—	73.3
			Median	—	—	—	—	N/A	—	—	66.7	—	—	—	N/A	—	—	1.8	—	—	—	—	-6.1	—	73.3
**Combination dishes (N)**	**Number of products**			**0**	**0**	**1**	**0**	**0**	**0**	**0**	**27**	**0**	**0**	**40**	**85**	**15**	**0**	**48**	**0**	**0**	**0**	**0**	**49**	**0**	**19**
	**HSR**	**Absolute change**	**Mean**	—	—	**0.0**	—	—	—	—	**0.1**	—	—	**0.0**	**0.1**	**-0.1**	—	**0.2**	—	—	—	—	**0.0**	—	**0.1**
			**Median**	—	—	**0.0**	—	—	—	—	**0.0**	—	—	**0.0**	**0.0**	**0.0**	—	**0.0**	—	—	—	—	**0.0**	—	**0.0**
		**Percent change**	**Mean**	—	—	**0.0**	—	—	—	—	**4.4**	—	—	**-0.3**	**3.7**	**-4.2**	—	**8.8**	—	—	—	—	**1.3**	—	**1.8**
			**Median**	—	—	**0.0**	—	—	—	—	**0.0**	—	—	**0.0**	**0.0**	**0.0**	—	**0.0**	—	—	—	—	**0.0**	—	**0.0**
	**Calories (kcal per 100 g/mL)**	**Absolute change**	**Mean**	—	—	**0.0**	—	—	—	—	**-0.6**	—	—	**-0.8**	**-7.5**	**-5.5**	—	**-7.9**	—	—	—	—	**-0.4**	—	**2.0**
			**Median**	—	—	**0.0**	—	—	—	—	**0.0**	—	—	**0.0**	**0.0**	**0.0**	—	**-7.8**	—	—	—	—	**0.0**	—	**5.6**
		**Percent change**	**Mean**	—	—	**0.0**	—	—	—	—	**-0.9**	—	—	**-0.2**	**-3.5**	**-1.6**	—	**-3.7**	—	—	—	—	**0.2**	—	**2.2**
			**Median**	—	—	**0.0**	—	—	—	—	**0.0**	—	—	**0.0**	**0.0**	**0.0**	—	**-3.8**	—	—	—	—	**0.0**	—	**5.3**
	**Sodium (mg per 100 g/mL)**	**Absolute change**	**Mean**	—	—	**0.0**	—	—	—	—	**-61.8**	—	—	**7.2**	**-18.7**	**-30.9**	—	**-22.7**	—	—	—	—	**-9.2**	—	**-7.5**
			**Median**	—	—	**0.0**	—	—	—	—	**-41.5**	—	—	**0.0**	**0.0**	**-30.1**	—	**-5.4**	—	—	—	—	**0.0**	—	**-6.0**
		**Percent change**	**Mean**	—	—	**0.0**	—	—	—	—	**-9.6**	—	—	**2.0**	**-1.0**	**-5.3**	—	**-3.6**	—	—	—	—	**-2.5**	—	**-2.9**
			**Median**	—	—	**0.0**	—	—	—	—	**-9.4**	—	—	**0.0**	**0.0**	**-6.1**	—	**-1.6**	—	—	—	—	**0.0**	—	**-2.2**
	**Saturated fat (g per 100 g/mL)**	**Absolute change**	**Mean**	—	—	**0.0**	—	—	—	—	**0.3**	—	—	**0.0**	**-0.2**	**0.2**	—	**-0.4**	—	—	—	—	**0.0**	—	**-0.1**
			**Median**	—	—	**0.0**	—	—	—	—	**0.1**	—	—	**0.0**	**0.0**	**0.0**	—	**-0.3**	—	—	—	—	**0.0**	—	**0.0**
		**Percent change**	**Mean**	—	—	**0.0**	—	—	—	—	**20.0**	—	—	**-4.2**	**-1.5**	**6.5**	—	**-11.9**	—	—	—	—	**11.2**	—	**-12.8**
			**Median**	—	—	**0.0**	—	—	—	—	**9.5**	—	—	**0.0**	**0.0**	**0.0**	—	**-12.3**	—	—	—	—	**0.0**	—	**0.0**
	**Trans fat (g per 100 g/mL)**	**Absolute change**	**Mean**	—	—	**0.0**	—	—	—	—	**-0.2**	—	—	**0.0**	**0.0**	**0.0**	—	**0.0**	—	—	—	—	**0.0**	—	**0.0**
			**Median**	—	—	**0.0**	—	—	—	—	**0.0**	—	—	**0.0**	**0.0**	**0.0**	—	**0.0**	—	—	—	—	**0.0**	—	**0.0**
		**Percent change**	**Mean**	—	—	**0.0**	—	—	—	—	**-74.3**	—	—	**N/A**	**N/A**	**N/A**	—	**N/A**	—	—	—	—	**2.5**	—	**N/A**
			**Median**	—	—	**0.0**	—	—	—	—	**-95.0**	—	—	**0.0**	**0.0**	**7.2**	—	**0.0**	—	—	—	—	**0.0**	—	**N/A**
	**Total sugars (g per 100 g/mL)**	**Absolute change**	**Mean**	—	—	**0.0**	—	—	—	—	**0.2**	—	—	**-0.2**	**0.5**	**1.2**	—	**-0.2**	—	—	—	—	**0.0**	—	**0.0**
			**Median**	—	—	**0.0**	—	—	—	—	**0.0**	—	—	**0.0**	**0.0**	**0.0**	—	**0.0**	—	—	—	—	**0.0**	—	**0.0**
		**Percent change**	**Mean**	—	—	**0.0**	—	—	—	—	**6.8**	—	—	**0.8**	**N/A**	**11.5**	—	**4.6**	—	—	—	—	**2.9**	—	**2.6**
			**Median**	—	—	**0.0**	—	—	—	—	**0.0**	—	—	**0.0**	**0.0**	**0.0**	—	**0.0**	—	—	—	—	**0.0**	—	**0.0**
	**Free sugars (g per 100 g/mL)**	**Absolute change**	**Mean**	—	—	**-0.4**	—	—	—	—	**0.4**	—	—	**0.0**	**0.3**	**1.2**	—	**-0.4**	—	—	—	—	**0.0**	—	**0.0**
			**Median**	—	—	**-0.4**	—	—	—	—	**0.1**	—	—	**0.0**	**0.0**	**1.3**	—	**-0.8**	—	—	—	—	**0.1**	—	**0.1**
		**Percent change**	**Mean**	—	—	**-46.7**	—	—	—	—	**N/A**	—	—	**-6.9**	**N/A**	**N/A**	—	**N/A**	—	—	—	—	**73.4**	—	**N/A**
			**Median**	—	—	**-46.7**	—	—	—	—	**14.5**	—	—	**6.6**	**4.9**	**19.8**	—	**-25.3**	—	—	—	—	**8.1**	—	**6.3**
**Nuts and seeds (O)**	**Number of products**			**0**	**0**	**0**	**0**	**0**	**0**	**0**	**0**	**0**	**0**	**8**	**26**	**0**	**0**	**0**	**0**	**0**	**0**	**0**	**10**	**0**	**0**
	**HSR**	**Absolute change**	**Mean**	—	—	—	—	—	—	—	—	—	—	**0.0**	**0.1**	—	—	—	—	—	—	—	**0.1**	—	—
			**Median**	—	—	—	—	—	—	—	—	—	—	**0.0**	**0.0**	—	—	—	—	—	—	—	**0.0**	—	—
		**Percent change**	**Mean**	—	—	—	—	—	—	—	—	—	—	**0.0**	**3.1**	—	—	—	—	—	—	—	**1.1**	—	—
			**Median**	—	—	—	—	—	—	—	—	—	—	**0.0**	**0.0**	—	—	—	—	—	—	—	**0.0**	—	—
	**Calories (kcal per 100 g/mL)**	**Absolute change**	**Mean**	—	—	—	—	—	—	—	—	—	—	**9.6**	**-21.9**	—	—	—	—	—	—	—	**0.0**	—	—
			**Median**	—	—	—	—	—	—	—	—	—	—	**0.0**	**0.0**	—	—	—	—	—	—	—	**0.0**	—	—
		**Percent change**	**Mean**	—	—	—	—	—	—	—	—	—	—	**1.8**	**-3.3**	—	—	—	—	—	—	—	**0.0**	—	—
			**Median**	—	—	—	—	—	—	—	—	—	—	**0.0**	**0.0**	—	—	—	—	—	—	—	**0.0**	—	—
	**Sodium (mg per 100 g/mL)**	**Absolute change**	**Mean**	—	—	—	—	—	—	—	—	—	—	**-4.8**	**-5.6**	—	—	—	—	—	—	—	**6.7**	—	—
			**Median**	—	—	—	—	—	—	—	—	—	—	**0.0**	**0.0**	—	—	—	—	—	—	—	**0.0**	—	—
		**Percent change**	**Mean**	—	—	—	—	—	—	—	—	—	—	**-1.7**	**-23.2**	—	—	—	—	—	—	—	**0.0**	—	—
			**Median**	—	—	—	—	—	—	—	—	—	—	**0.0**	**0.0**	—	—	—	—	—	—	—	**0.0**	—	—
	**Saturated fat (g per 100 g/mL)**	**Absolute change**	**Mean**	—	—	—	—	—	—	—	—	—	—	**0.0**	**-0.2**	—	—	—	—	—	—	—	**0.0**	—	—
			**Median**	—	—	—	—	—	—	—	—	—	—	**0.0**	**0.0**	—	—	—	—	—	—	—	**0.0**	—	—
		**Percent change**	**Mean**	—	—	—	—	—	—	—	—	—	—	**0.0**	**-2.9**	—	—	—	—	—	—	—	**0.0**	—	—
			**Median**	—	—	—	—	—	—	—	—	—	—	**0.0**	**0.0**	—	—	—	—	—	—	—	**0.0**	—	—
	**Trans fat (g per 100**	**Absolute change**	**Mean**	—	—	—	—	—	—	—	—	—	—	**0.0**	**0.0**	—	—	—	—	—	—	—	**0.0**	—	—
			**Median**	—	—	—	—	—	—	—	—	—	—	**0.0**	**0.0**	—	—	—	—	—	—	—	**0.0**	—	—

				A. Lassonde	Agropur	Campbell Soup	Canada Bread	Canada Dry Mott's	Coca-Cola	Danone	General Mills	George Weston	Kellogg	Kraft Heinz	Loblaw	Maple Leaf Foods	Mondelēz	Nestlé	Ocean Spray	Parmalat	PepsiCo	Saputo	Sobeys	Sun-Rype	Unilever
	**g/mL)**	**Percent**	**Mean**	—	—	—	—	—	—	—	—	—	—	**N/A**	**N/A**	—	—	—	—	—	—	—	**0.0**	—	—
		**change**	**Median**	—	—	—	—	—	—	—	—	—	—	**N/A**	**N/A**	—	—	—	—	—	—	—	**0.0**	—	—
	**Total**	**Absolute**	**Mean**	—	—	—	—	—	—	—	—	—	—	**0.0**	**0.0**	—	—	—	—	—	—	—	**0.0**	—	—
	**sugars (g**	**change**	**Median**	—	—	—	—	—	—	—	—	—	—	**0.0**	**0.0**	—	—	—	—	—	—	—	**0.0**	—	—
	**per 100**	**Percent**	**Mean**	—	—	—	—	—	—	—	—	—	—	**0.0**	**3.5**	—	—	—	—	—	—	—	**0.0**	—	—
	**g/mL)**	**change**	**Median**	—	—	—	—	—	—	—	—	—	—	**0.0**	**0.0**	—	—	—	—	—	—	—	**0.0**	—	—
	**Free**	**Absolute**	**Mean**	—	—	—	—	—	—	—	—	—	—	**-0.1**	**-0.1**	—	—	—	—	—	—	—	**-0.1**	—	—
	**sugars (g**	**change**	**Median**	—	—	—	—	—	—	—	—	—	—	**0.0**	**0.0**	—	—	—	—	—	—	—	**0.0**	—	—
	**per 100**	**Percent**	**Mean**	—	—	—	—	—	—	—	—	—	—	**-1.7**	**-31.4**	—	—	—	—	—	—	—	**-4.0**	—	—
	**g/mL)**	**change**	**Median**	—	—	—	—	—	—	—	—	—	—	**-2.0**	**-31.4**	—	—	—	—	—	—	—	**-4.0**	—	—
Nut butters,	Number of products			0	0	0	0	0	0	0	0	0	0	8	8	0	0	0	0	0	0	0	5	0	0
pastes and	HSR	Absolute	Mean	—	—	—	—	—	—	—	—	—	—	0.0	0.3	—	—	—	—	—	—	—	0.1	—	—
creams (O3)		change	Median	—	—	—	—	—	—	—	—	—	—	0.0	0.5	—	—	—	—	—	—	—	0.0	—	—
		Percent	Mean	—	—	—	—	—	—	—	—	—	—	0.0	8.7	—	—	—	—	—	—	—	2.2	—	—
		change (%)	Median	—	—	—	—	—	—	—	—	—	—	0.0	11.1	—	—	—	—	—	—	—	0.0	—	—
	Calories	Absolute	Mean	—	—	—	—	—	—	—	—	—	—	9.6	-61.3	—	—	—	—	—	—	—	0.0	—	—
	(kcal per	change	Median	—	—	—	—	—	—	—	—	—	—	0.0	-66.7	—	—	—	—	—	—	—	0.0	—	—
	100 g/mL)	Percent	Mean	—	—	—	—	—	—	—	—	—	—	1.8	-9.2	—	—	—	—	—	—	—	0.0	—	—
		change (%)	Median	—	—	—	—	—	—	—	—	—	—	0.0	-10.0	—	—	—	—	—	—	—	0.0	—	—
	Sodium	Absolute	Mean	—	—	—	—	—	—	—	—	—	—	-4.8	-19.3	—	—	—	—	—	—	—	13.3	—	—
	(mg per	change	Median	—	—	—	—	—	—	—	—	—	—	0.0	-10.7	—	—	—	—	—	—	—	0.0	—	—
	100 g/mL)	Percent	Mean	—	—	—	—	—	—	—	—	—	—	-1.7	-58.0	—	—	—	—	—	—	—	0.0	—	—
		change (%)	Median	—	—	—	—	—	—	—	—	—	—	0.0	-62.7	—	—	—	—	—	—	—	0.0	—	—
	Saturated	Absolute	Mean	—	—	—	—	—	—	—	—	—	—	0.0	-0.1	—	—	—	—	—	—	—	0.0	—	—
	fat (g per	change	Median	—	—	—	—	—	—	—	—	—	—	0.0	0.0	—	—	—	—	—	—	—	0.0	—	—
	100 g/mL)	Percent	Mean	—	—	—	—	—	—	—	—	—	—	0.0	-1.7	—	—	—	—	—	—	—	0.0	—	—
		change (%)	Median	—	—	—	—	—	—	—	—	—	—	0.0	0.0	—	—	—	—	—	—	—	0.0	—	—
	Trans fat (g	Absolute	Mean	—	—	—	—	—	—	—	—	—	—	0.0	0.0	—	—	—	—	—	—	—	0.0	—	—
	per 100	change	Median	—	—	—	—	—	—	—	—	—	—	0.0	0.0	—	—	—	—	—	—	—	0.0	—	—
	g/mL)	Percent	Mean	—	—	—	—	—	—	—	—	—	—	N/A	N/A	—	—	—	—	—	—	—	N/A	—	—
		change (%)	Median	—	—	—	—	—	—	—	—	—	—	N/A	N/A	—	—	—	—	—	—	—	N/A	—	—
	Total	Absolute	Mean	—	—	—	—	—	—	—	—	—	—	0.0	-0.1	—	—	—	—	—	—	—	0.0	—	—
	sugars (g	change	Median	—	—	—	—	—	—	—	—	—	—	0.0	0.0	—	—	—	—	—	—	—	0.0	—	—
	per 100	Percent	Mean	—	—	—	—	—	—	—	—	—	—	0.0	-1.7	—	—	—	—	—	—	—	0.0	—	—
	g/mL)	change (%)	Median	—	—	—	—	—	—	—	—	—	—	0.0	0.0	—	—	—	—	—	—	—	0.0	—	—
	Free sugars	Absolute	Mean	—	—	—	—	—	—	—	—	—	—	-0.1	-0.2	—	—	—	—	—	—	—	-0.1	—	—
	(g per 100	change	Median	—	—	—	—	—	—	—	—	—	—	0.0	0.0	—	—	—	—	—	—	—	0.0	—	—
	g/mL)	Percent	Mean	—	—	—	—	—	—	—	—	—	—	-1.7	-31.4	—	—	—	—	—	—	—	-4.0	—	—
		change (%)	Median	—	—	—	—	—	—	—	—	—	—	-2.0	-31.4	—	—	—	—	—	—	—	-4.0	—	—
Nuts, seeds and	Number of products			0	0	0	0	0	0	0	0	0	0	0	18	0	0	0	0	0	0	0	5	0	0
flours (O1, O4)	HSR	Absolute	Mean	—	—	—	—	—	—	—	—	—	—	—	0.0	—	—	—	—	—	—	—	0.0	—	—
		change	Median	—	—	—	—	—	—	—	—	—	—	—	0.0	—	—	—	—	—	—	—	0.0	—	—
		Percent	Mean	—	—	—	—	—	—	—	—	—	—	—	0.6	—	—	—	—	—	—	—	0.0	—	—
		change (%)	Median	—	—	—	—	—	—	—	—	—	—	—	0.0	—	—	—	—	—	—	—	0.0	—	—
	Calories	Absolute	Mean	—	—	—	—	—	—	—	—	—	—	—	-4.4	—	—	—	—	—	—	—	0.0	—	—
	(kcal per	change	Median	—	—	—	—	—	—	—	—	—	—	—	0.0	—	—	—	—	—	—	—	0.0	—	—
	100 g/mL)	Percent	Mean	—	—	—	—	—	—	—	—	—	—	—	-0.6	—	—	—	—	—	—	—	0.0	—	—
		change (%)	Median	—	—	—	—	—	—	—	—	—	—	—	0.0	—	—	—	—	—	—	—	0.0	—	—
	Sodium	Absolute	Mean	—	—	—	—	—	—	—	—	—	—	—	0.6	—	—	—	—	—	—	—	0.0	—	—
	(mg per	change	Median	—	—	—	—	—	—	—	—	—	—	—	0.0	—	—	—	—	—	—	—	0.0	—	—
	100 g/mL)	Percent	Mean	—	—	—	—	—	—	—	—	—	—	—	0.0	—	—	—	—	—	—	—	0.0	—	—
		change (%)	Median	—	—	—	—	—	—	—	—	—	—	—	0.0	—	—	—	—	—	—	—	0.0	—	—
	Saturated	Absolute	Mean	—	—	—	—	—	—	—	—	—	—	—	-0.2	—	—	—	—	—	—	—	0.0	—	—
	fat (g per	change	Median	—	—	—	—	—	—	—	—	—	—	—	0.0	—	—	—	—	—	—	—	0.0	—	—
	100 g/mL)	Percent	Mean	—	—	—	—	—	—	—	—	—	—	—	-3.4	—	—	—	—	—	—	—	0.0	—	—
		change (%)	Median	—	—	—	—	—	—	—	—	—	—	—	0.0	—	—	—	—	—	—	—	0.0	—	—
	Trans fat (g	Absolute	Mean	—	—	—	—	—	—	—	—	—	—	—	0.0	—	—	—	—	—	—	—	0.0	—	—
	per 100	change	Median	—	—	—	—	—	—	—	—	—	—	—	0.0	—	—	—	—	—	—	—	0.0	—	—
	g/mL)	Percent	Mean	—	—	—	—	—	—	—	—	—	—	—	N/A	—	—	—	—	—	—	—	0.0	—	—
		change (%)	Median	—	—	—	—	—	—	—	—	—	—	—	N/A	—	—	—	—	—	—	—	0.0	—	—
	Total	Absolute	Mean	—	—	—	—	—	—	—	—	—	—	—	0.1	—	—	—	—	—	—	—	0.0	—	—
	sugars (g	change	Median	—	—	—	—	—	—	—	—	—	—	—	0.0	—	—	—	—	—	—	—	0.0	—	—
	per 100	Percent	Mean	—	—	—	—	—	—	—	—	—	—	—	5.9	—	—	—	—	—	—	—	0.0	—	—
	g/mL)	change (%)	Median	—	—	—	—	—	—	—	—	—	—	—	0.0	—	—	—	—	—	—	—	0.0	—	—
	Free sugars	Absolute	Mean	—	—	—	—	—	—	—	—	—	—	—	0.0	—	—	—	—	—	—	—	0.0	—	—
	(g per 100	change	Median	—	—	—	—	—	—	—	—	—	—	—	0.0	—	—	—	—	—	—	—	0.0	—	—
	g/mL)	Percent	Mean	—	—	—	—	—	—	—	—	—	—	—	N/A	—	—	—	—	—	—	—	N/A	—	—
		change (%)	Median	—	—	—	—	—	—	—	—	—	—	—	N/A	—	—	—	—	—	—	—	N/A	—	—
**Potatoes (P)**	**Number of products**			**0**	**0**	**0**	**0**	**0**	**0**	**0**	**7**	**0**	**0**	**0**	**11**	**0**	**0**	**0**	**0**	**0**	**0**	**0**	**7**	**0**	**0**
	**HSR**	**Absolute**	**Mean**	—	—	—	—	—	—	—	**0.1**	—	—	—	**0.1**	—	—	—	—	—	—	—	**0.0**	—	—
		**change**	**Median**	—	—	—	—	—	—	—	**0.0**	—	—	—	**0.0**	—	—	—	—	—	—	—	**0.0**	—	—
		**Percent**	**Mean**	—	—	—	—	—	—	—	**4.8**	—	—	—	**2.4**	—	—	—	—	—	—	—	**0.0**	—	—
		**change**	**Median**	—	—	—	—	—	—	—	**0.0**	—	—	—	**0.0**	—	—	—	—	—	—	—	**0.0**	—	—

				A. Lassonde	Agropur	Campbell Soup	Canada Bread	Canada Dry Mott's	Coca-Cola	Danone	General Mills	George Weston	Kellogg	Kraft Heinz	Loblaw	Maple Leaf Foods	Mondelēz	Nestlé	Ocean Spray	Parmalat	PepsiCo	Saputo	Sobeys	Sun-Rype	Unilever
	Calories	Absolute	Mean	—	—	—	—	—	—	—	-5.3	—	—	—	0.6	—	—	—	—	—	—	—	1.7	—	—
	(kcal per	change	Median	—	—	—	—	—	—	—	0.0	—	—	—	0.0	—	—	—	—	—	—	—	0.0	—	—
	100 g/mL)	Percent	Mean	—	—	—	—	—	—	—	-1.4	—	—	—	19.1	—	—	—	—	—	—	—	5.2	—	—
		change	Median	—	—	—	—	—	—	—	0.0	—	—	—	0.0	—	—	—	—	—	—	—	0.0	—	—
	Sodium	Absolute	Mean	—	—	—	—	—	—	—	-4.8	—	—	—	-8.0	—	—	—	—	—	—	—	5.8	—	—
	(mg per	change	Median	—	—	—	—	—	—	—	0.0	—	—	—	0.0	—	—	—	—	—	—	—	0.0	—	—
	100 g/mL)	Percent	Mean	—	—	—	—	—	—	—	-0.3	—	—	—	18.4	—	—	—	—	—	—	—	5.2	—	—
		change	Median	—	—	—	—	—	—	—	0.0	—	—	—	0.0	—	—	—	—	—	—	—	0.0	—	—
	Saturated	Absolute	Mean	—	—	—	—	—	—	—	0.0	—	—	—	-0.1	—	—	—	—	—	—	—	0.0	—	—
	fat (g per	change	Median	—	—	—	—	—	—	—	0.0	—	—	—	0.0	—	—	—	—	—	—	—	0.0	—	—
	100 g/mL)	Percent	Mean	—	—	—	—	—	—	—	-66.7	—	—	—	-8.6	—	—	—	—	—	—	—	0.0	—	—
		change	Median	—	—	—	—	—	—	—	-100.0	—	—	—	0.0	—	—	—	—	—	—	—	0.0	—	—
	Trans fat	Absolute	Mean	—	—	—	—	—	—	—	0.0	—	—	—	0.0	—	—	—	—	—	—	—	0.0	—	—
	(g per 100	change	Median	—	—	—	—	—	—	—	0.0	—	—	—	0.0	—	—	—	—	—	—	—	0.0	—	—
	g/mL)	Percent	Mean	—	—	—	—	—	—	—	N/A	—	—	—	N/A	—	—	—	—	—	—	—	N/A	—	—
		change	Median	—	—	—	—	—	—	—	N/A	—	—	—	N/A	—	—	—	—	—	—	—	N/A	—	—
	Total	Absolute	Mean	—	—	—	—	—	—	—	0.0	—	—	—	-0.3	—	—	—	—	—	—	—	0.0	—	—
	sugars (g	change	Median	—	—	—	—	—	—	—	0.0	—	—	—	0.0	—	—	—	—	—	—	—	0.0	—	—
	per 100	Percent	Mean	—	—	—	—	—	—	—	0.0	—	—	—	-50.0	—	—	—	—	—	—	—	0.0	—	—
	g/mL)	change	Median	—	—	—	—	—	—	—	0.0	—	—	—	-50.0	—	—	—	—	—	—	—	0.0	—	—
	Free	Absolute	Mean	—	—	—	—	—	—	—	0.4	—	—	—	0.0	—	—	—	—	—	—	—	0.2	—	—
	sugars (g	change	Median	—	—	—	—	—	—	—	0.0	—	—	—	0.0	—	—	—	—	—	—	—	0.0	—	—
	per 100	Percent	Mean	—	—	—	—	—	—	—	N/A	—	—	—	N/A	—	—	—	—	—	—	—	N/A	—	—
	g/mL)	change	Median	—	—	—	—	—	—	—	N/A	—	—	—	N/A	—	—	—	—	—	—	—	N/A	—	—
Salads (Q)	Number of products			0	0	0	0	0	0	0	0	0	0	0	7	0	0	0	0	0	0	0	3	0	0
	HSR	Absolute	Mean	—	—	—	—	—	—	—	—	—	—	—	0.1	—	—	—	—	—	—	—	0.2	—	—
		change	Median	—	—	—	—	—	—	—	—	—	—	—	0.0	—	—	—	—	—	—	—	0.0	—	—
		Percent	Mean	—	—	—	—	—	—	—	—	—	—	—	1.6	—	—	—	—	—	—	—	4.8	—	—
		change	Median	—	—	—	—	—	—	—	—	—	—	—	0.0	—	—	—	—	—	—	—	0.0	—	—
	Calories	Absolute	Mean	—	—	—	—	—	—	—	—	—	—	—	0.3	—	—	—	—	—	—	—	0.0	—	—
	(kcal per	change	Median	—	—	—	—	—	—	—	—	—	—	—	0.0	—	—	—	—	—	—	—	0.0	—	—
	100 g/mL)	Percent	Mean	—	—	—	—	—	—	—	—	—	—	—	0.3	—	—	—	—	—	—	—	0.0	—	—
		change	Median	—	—	—	—	—	—	—	—	—	—	—	0.0	—	—	—	—	—	—	—	0.0	—	—
	Sodium	Absolute	Mean	—	—	—	—	—	—	—	—	—	—	—	-4.9	—	—	—	—	—	—	—	0.0	—	—
	(mg per	change	Median	—	—	—	—	—	—	—	—	—	—	—	0.0	—	—	—	—	—	—	—	0.0	—	—
	100 g/mL)	Percent	Mean	—	—	—	—	—	—	—	—	—	—	—	-1.5	—	—	—	—	—	—	—	0.0	—	—
		change	Median	—	—	—	—	—	—	—	—	—	—	—	0.0	—	—	—	—	—	—	—	0.0	—	—
	Saturated	Absolute	Mean	—	—	—	—	—	—	—	—	—	—	—	-0.1	—	—	—	—	—	—	—	0.0	—	—
	fat (g per	change	Median	—	—	—	—	—	—	—	—	—	—	—	0.0	—	—	—	—	—	—	—	0.0	—	—
	100 g/mL)	Percent	Mean	—	—	—	—	—	—	—	—	—	—	—	-9.2	—	—	—	—	—	—	—	0.0	—	—
		change	Median	—	—	—	—	—	—	—	—	—	—	—	0.0	—	—	—	—	—	—	—	0.0	—	—
	Trans fat	Absolute	Mean	—	—	—	—	—	—	—	—	—	—	—	0.0	—	—	—	—	—	—	—	0.0	—	—
	(g per 100	change	Median	—	—	—	—	—	—	—	—	—	—	—	0.0	—	—	—	—	—	—	—	0.0	—	—
	g/mL)	Percent	Mean	—	—	—	—	—	—	—	—	—	—	—	-50.0	—	—	—	—	—	—	—	0.0	—	—
		change	Median	—	—	—	—	—	—	—	—	—	—	—	-50.0	—	—	—	—	—	—	—	0.0	—	—
	Total	Absolute	Mean	—	—	—	—	—	—	—	—	—	—	—	-0.3	—	—	—	—	—	—	—	0.0	—	—
	sugars (g	change	Median	—	—	—	—	—	—	—	—	—	—	—	0.0	—	—	—	—	—	—	—	0.0	—	—
	per 100	Percent	Mean	—	—	—	—	—	—	—	—	—	—	—	-7.5	—	—	—	—	—	—	—	0.0	—	—
	g/mL)	change	Median	—	—	—	—	—	—	—	—	—	—	—	0.0	—	—	—	—	—	—	—	0.0	—	—
	Free	Absolute	Mean	—	—	—	—	—	—	—	—	—	—	—	-1.0	—	—	—	—	—	—	—	-1.2	—	—
	sugars (g	change	Median	—	—	—	—	—	—	—	—	—	—	—	-1.1	—	—	—	—	—	—	—	-1.3	—	—
	per 100	Percent	Mean	—	—	—	—	—	—	—	—	—	—	—	-28.6	—	—	—	—	—	—	—	-78.7	—	—
	g/mL)	change	Median	—	—	—	—	—	—	—	—	—	—	—	-8.0	—	—	—	—	—	—	—	-80.6	—	—
Sauces, dips,	Number of products			0	0	12	0	0	0	0	7	0	0	31	167	1	0	1	2	0	12	3	52	0	14
gravies and	HSR	Absolute	Mean	—	—	0.0	—	—	—	—	0.4	—	—	0.0	0.0	0.0	—	0.0	0.0	—	-0.1	0.0	0.0	—	0.0
condiments		change	Median	—	—	0.0	—	—	—	—	0.5	—	—	0.0	0.0	0.0	—	0.0	0.0	—	0.0	0.0	0.0	—	0.0
(R)		Percent	Mean	—	—	0.0	—	—	—	—	11.9	—	—	2.8	1.3	0.0	—	0.0	0.0	—	-2.1	0.0	-0.4	—	1.4
		change	Median	—	—	0.0	—	—	—	—	16.7	—	—	0.0	0.0	0.0	—	0.0	0.0	—	0.0	0.0	0.0	—	0.0
	Calories	Absolute	Mean	—	—	0.7	—	—	—	—	-7.1	—	—	-10.1	0.9	0.0	—	0.0	0.0	—	0.0	-32.3	0.4	—	-31.6
	(kcal per	change	Median	—	—	0.0	—	—	—	—	-10.0	—	—	0.0	0.0	0.0	—	0.0	0.0	—	0.0	-32.3	0.0	—	-10.9
	100 g/mL)	Percent	Mean	—	—	3.1	—	—	—	—	-15.7	—	—	-1.5	0.8	0.0	—	0.0	0.0	—	0.0	-16.7	0.6	—	-10.9
		change	Median	—	—	0.0	—	—	—	—	-20.0	—	—	0.0	0.0	0.0	—	0.0	0.0	—	0.0	-16.7	0.0	—	-2.9
	Sodium	Absolute	Mean	—	—	69.4	—	—	—	—	-182.9	—	—	-142.3	-46.9	0.0	—	0.0	0.0	—	-3.0	0.0	-118.7	—	-569.0
	(mg per	change	Median	—	—	0.0	—	—	—	—	-200.0	—	—	0.0	0.0	0.0	—	0.0	0.0	—	0.0	0.0	0.0	—	-649.5
	100 g/mL)	Percent	Mean	—	—	1.4	—	—	—	—	-22.0	—	—	-4.6	-4.9	0.0	—	0.0	0.0	—	-0.6	0.0	-0.9	—	-12.2
		change	Median	—	—	0.0	—	—	—	—	-21.7	—	—	0.0	0.0	0.0	—	0.0	0.0	—	0.0	0.0	0.0	—	-15.6
	Saturated	Absolute	Mean	—	—	0.0	—	—	—	—	0.0	—	—	-0.1	0.1	0.0	—	0.0	0.0	—	0.0	-0.5	-0.1	—	-1.3
	fat (g per	change	Median	—	—	0.0	—	—	—	—	0.0	—	—	0.0	0.0	0.0	—	0.0	0.0	—	0.0	0.0	0.0	—	0.0
	100 g/mL)	Percent	Mean	—	—	0.0	—	—	—	—	0.0	—	—	-25.0	8.8	N/A	—	N/A	N/A	—	0.0	-5.6	-7.4	—	-54.7
		change	Median	—	—	0.0	—	—	—	—	0.0	—	—	0.0	0.0	N/A	—	N/A	N/A	—	0.0	0.0	0.0	—	-100.0
	Trans fat	Absolute	Mean	—	—	0.0	—	—	—	—	0.0	—	—	0.0	0.0	0.0	—	0.0	0.0	—	0.0	-0.1	0.0	—	-0.2
	(g per 100	change	Median	—	—	0.0	—	—	—	—	0.0	—	—	0.0	0.0	0.0	—	0.0	0.0	—	0.0	0.0	0.0	—	0.0
	g/mL)	Percent	Mean	—	—	N/A	—	—	—	—	0.0	—	—	0.0	-0.8	N/A	—	N/A	N/A	—	N/A	-100.0	-2.8	—	-75.8

				A. Lassonde	Agropur	Campbell Soup	Canada Bread	Canada Dry Mott's	Coca-Cola	Danone	General Mills	George Weston	Kellogg	Kraft Heinz	Loblaw	Maple Leaf Foods	Mondelēz	Nestlé	Ocean Spray	Parmalat	PepsiCo	Saputo	Sobeys	Sun-Rype	Unilever
		change	Median	—	—	N/A	—	—	—	—	0.0	—	—	0.0	0.0	N/A	—	N/A	N/A	—	N/A	-100.0	0.0	—	-75.8
	Total	Absolute	Mean	—	—	0.0	—	—	—	—	-0.6	—	—	-0.5	0.5	0.0	—	0.0	0.0	—	0.0	1.1	0.5	—	-1.3
	sugars (g	change	Median	—	—	0.0	—	—	—	—	0.0	—	—	0.0	0.0	0.0	—	0.0	0.0	—	0.0	0.0	0.0	—	-0.1
	per 100	Percent	Mean	—	—	0.0	—	—	—	—	-9.5	—	—	-0.5	0.1	0.0	—	0.0	0.0	—	0.0	33.3	-2.2	—	-17.0
	g/mL)	change	Median	—	—	0.0	—	—	—	—	0.0	—	—	0.0	0.0	0.0	—	0.0	0.0	—	0.0	0.0	0.0	—	-2.9
	Free	Absolute	Mean	—	—	-0.6	—	—	—	—	-0.7	—	—	-0.7	0.1	0.0	—	0.0	0.0	—	-0.1	-1.5	0.4	—	-1.3
	sugars (g	change	Median	—	—	0.0	—	—	—	—	-0.4	—	—	0.0	0.0	0.0	—	0.0	0.0	—	0.0	-0.6	0.0	—	0.0
	per 100	Percent	Mean	—	—	-10.7	—	—	—	—	-49.7	—	—	-4.5	-15.2	0.0	—	0.0	0.0	—	-70.4	-100.0	-13.7	—	-16.4
	g/mL)	change	Median	—	—	-10.4	—	—	—	—	-52.2	—	—	0.0	0.0	0.0	—	0.0	0.0	—	-70.4	-100.0	0.0	—	-5.4
Snacks (S)	Number of products			0	0	0	0	0	0	0	3	0	6	0	63	5	2	0	0	0	69	0	48	0	0
	HSR	Absolute	Mean	—	—	—	—	—	—	—	-0.5	—	-0.1	—	0.1	0.1	0.0	—	—	—	0.0	—	0.0	—	—
		change	Median	—	—	—	—	—	—	—	-0.5	—	0.0	—	0.0	0.0	0.0	—	—	—	0.0	—	0.0	—	—
		Percent	Mean	—	—	—	—	—	—	—	-12.5	—	0.0	—	4.0	6.7	0.0	—	—	—	2.5	—	-0.9	—	—
		change	Median	—	—	—	—	—	—	—	-12.5	—	0.0	—	0.0	0.0	0.0	—	—	—	0.0	—	0.0	—	—
	Calories	Absolute	Mean	—	—	—	—	—	—	—	-1.1	—	16.7	—	-7.3	-4.0	0.0	—	—	—	2.4	—	-2.1	—	—
	(kcal per	change	Median	—	—	—	—	—	—	—	0.0	—	0.0	—	0.0	0.0	0.0	—	—	—	0.0	—	0.0	—	—
	100 g/mL)	Percent	Mean	—	—	—	—	—	—	—	-0.3	—	4.2	—	-1.4	-1.7	0.0	—	—	—	0.5	—	-0.3	—	—
		change	Median	—	—	—	—	—	—	—	0.0	—	0.0	—	0.0	0.0	0.0	—	—	—	0.0	—	0.0	—	—
	Sodium	Absolute	Mean	—	—	—	—	—	—	—	0.0	—	14.3	—	-27.4	-36.0	0.0	—	—	—	-37.6	—	-12.6	—	—
	(mg per	change	Median	—	—	—	—	—	—	—	0.0	—	0.0	—	0.0	0.0	0.0	—	—	—	0.0	—	0.0	—	—
	100 g/mL)	Percent	Mean	—	—	—	—	—	—	—	0.0	—	8.3	—	-5.6	-3.5	0.0	—	—	—	-2.1	—	-0.7	—	—
		change	Median	—	—	—	—	—	—	—	0.0	—	0.0	—	0.0	0.0	0.0	—	—	—	0.0	—	0.0	—	—
	Saturated	Absolute	Mean	—	—	—	—	—	—	—	-0.4	—	0.2	—	-0.2	0.2	0.0	—	—	—	0.0	—	0.3	—	—
	fat (g per	change	Median	—	—	—	—	—	—	—	0.0	—	0.0	—	0.0	0.0	0.0	—	—	—	0.0	—	0.0	—	—
	100 g/mL)	Percent	Mean	—	—	—	—	—	—	—	-16.7	—	22.2	—	-2.8	5.0	0.0	—	—	—	2.5	—	5.0	—	—
		change	Median	—	—	—	—	—	—	—	0.0	—	0.0	—	0.0	0.0	0.0	—	—	—	0.0	—	0.0	—	—
	Trans fat	Absolute	Mean	—	—	—	—	—	—	—	0.0	—	0.0	—	0.0	0.0	0.0	—	—	—	0.0	—	0.0	—	—
	(g per 100	change	Median	—	—	—	—	—	—	—	0.0	—	0.0	—	0.0	0.0	0.0	—	—	—	0.0	—	0.0	—	—
	g/mL)	Percent	Mean	—	—	—	—	—	—	—	N/A	—	N/A	—	-6.0	0.0	0.0	—	—	—	-2.0	—	-16.7	—	—
		change	Median	—	—	—	—	—	—	—	N/A	—	N/A	—	0.0	0.0	0.0	—	—	—	0.0	—	0.0	—	—
	Total	Absolute	Mean	—	—	—	—	—	—	—	0.8	—	-0.4	—	-0.1	-0.4	0.0	—	—	—	-0.1	—	0.5	—	—
	sugars (g	change	Median	—	—	—	—	—	—	—	0.0	—	0.0	—	0.0	0.0	0.0	—	—	—	0.0	—	0.0	—	—
	per 100	Percent	Mean	—	—	—	—	—	—	—	11.1	—	-10.0	—	1.3	-25.0	0.0	—	—	—	-6.2	—	25.3	—	—
	g/mL)	change	Median	—	—	—	—	—	—	—	0.0	—	0.0	—	0.0	-25.0	0.0	—	—	—	0.0	—	0.0	—	—
	Free	Absolute	Mean	—	—	—	—	—	—	—	0.7	—	0.5	—	0.1	-0.4	-0.2	—	—	—	0.0	—	1.4	—	—
	sugars (g	change	Median	—	—	—	—	—	—	—	-0.1	—	0.2	—	0.0	0.0	-0.2	—	—	—	0.0	—	0.0	—	—
	per 100	Percent	Mean	—	—	—	—	—	—	—	9.5	—	30.5	—	16.9	-25.0	-4.8	—	—	—	10.5	—	92.4	—	—
	g/mL)	change	Median	—	—	—	—	—	—	—	-2.4	—	19.6	—	4.5	-25.0	-4.8	—	—	—	14.0	—	33.0	—	—
Soups (T)	Number of products			0	0	79	0	0	0	0	0	0	0	0	43	0	0	0	0	0	0	0	22	0	45
	HSR	Absolute	Mean	—	—	0.0	—	—	—	—	—	—	—	—	0.0	—	—	—	—	—	—	—	0.0	—	0.0
		change	Median	—	—	0.0	—	—	—	—	—	—	—	—	0.0	—	—	—	—	—	—	—	0.0	—	0.0
		Percent	Mean	—	—	-0.6	—	—	—	—	—	—	—	—	0.6	—	—	—	—	—	—	—	-0.6	—	0.8
		change	Median	—	—	0.0	—	—	—	—	—	—	—	—	0.0	—	—	—	—	—	—	—	0.0	—	0.0
	Calories	Absolute	Mean	—	—	0.6	—	—	—	—	—	—	—	—	-1.4	—	—	—	—	—	—	—	3.0	—	13.1
	(kcal per	change	Median	—	—	0.0	—	—	—	—	—	—	—	—	0.0	—	—	—	—	—	—	—	0.0	—	0.0
	100 g/mL)	Percent	Mean	—	—	1.2	—	—	—	—	—	—	—	—	-1.6	—	—	—	—	—	—	—	18.8	—	8.8
		change	Median	—	—	0.0	—	—	—	—	—	—	—	—	0.0	—	—	—	—	—	—	—	0.0	—	0.0
	Sodium	Absolute	Mean	—	—	15.2	—	—	—	—	—	—	—	—	-101.7	—	—	—	—	—	—	—	21.1	—	-21.4
	(mg per	change	Median	—	—	0.0	—	—	—	—	—	—	—	—	0.0	—	—	—	—	—	—	—	0.0	—	-105.3
	100 g/mL)	Percent	Mean	—	—	5.2	—	—	—	—	—	—	—	—	5.5	—	—	—	—	—	—	—	5.2	—	-1.2
		change	Median	—	—	0.0	—	—	—	—	—	—	—	—	0.0	—	—	—	—	—	—	—	0.0	—	-1.7
	Saturated	Absolute	Mean	—	—	0.0	—	—	—	—	—	—	—	—	0.0	—	—	—	—	—	—	—	0.1	—	0.2
	fat (g per	change	Median	—	—	0.0	—	—	—	—	—	—	—	—	0.0	—	—	—	—	—	—	—	0.0	—	0.0
	100 g/mL)	Percent	Mean	—	—	16.5	—	—	—	—	—	—	—	—	1.2	—	—	—	—	—	—	—	22.9	—	-4.1
		change	Median	—	—	0.0	—	—	—	—	—	—	—	—	0.0	—	—	—	—	—	—	—	0.0	—	0.0
	Trans fat	Absolute	Mean	—	—	0.0	—	—	—	—	—	—	—	—	0.0	—	—	—	—	—	—	—	0.0	—	-0.1
	(g per 100	change	Median	—	—	0.0	—	—	—	—	—	—	—	—	0.0	—	—	—	—	—	—	—	0.0	—	0.0
	g/mL)	Percent	Mean	—	—	0.0	—	—	—	—	—	—	—	—	-8.3	—	—	—	—	—	—	—	N/A	—	-100.0
		change	Median	—	—	0.0	—	—	—	—	—	—	—	—	0.0	—	—	—	—	—	—	—	N/A	—	-100.0
	Total	Absolute	Mean	—	—	0.0	—	—	—	—	—	—	—	—	0.0	—	—	—	—	—	—	—	0.3	—	3.0
	sugars (g	change	Median	—	—	0.0	—	—	—	—	—	—	—	—	0.0	—	—	—	—	—	—	—	0.0	—	0.0
	per 100	Percent	Mean	—	—	-0.4	—	—	—	—	—	—	—	—	-2.9	—	—	—	—	—	—	—	-3.0	—	7.0
	g/mL)	change	Median	—	—	0.0	—	—	—	—	—	—	—	—	0.0	—	—	—	—	—	—	—	0.0	—	0.0
	Free	Absolute	Mean	—	—	0.1	—	—	—	—	—	—	—	—	-0.2	—	—	—	—	—	—	—	0.4	—	-0.4
	sugars (g	change	Median	—	—	0.0	—	—	—	—	—	—	—	—	0.0	—	—	—	—	—	—	—	0.0	—	0.0
	per 100	Percent	Mean	—	—	7.3	—	—	—	—	—	—	—	—	-8.5	—	—	—	—	—	—	—	28.4	—	-65.7
	g/mL)	change	Median	—	—	1.5	—	—	—	—	—	—	—	—	0.0	—	—	—	—	—	—	—	41.5	—	-88.6
Sugars and sweets (U)	Number of products			0	0	0	0	2	0	0	6	0	0	5	72	0	23	18	0	0	3	0	24	8	0
	HSR	Absolute	Mean	—	—	—	—	-0.3	—	—	0.2	—	—	0.3	0.0	—	0.0	0.0	—	—	0.0	—	0.0	0.0	—
		change	Median	—	—	—	—	-0.3	—	—	0.0	—	—	0.0	0.0	—	0.0	0.0	—	—	0.0	—	0.0	0.0	—
		Percent	Mean	—	—	—	—	-16.7	—	—	13.9	—	—	60.0	-1.9	—	-3.6	-1.9	—	—	0.0	—	1.0	0.0	—
		change	Median	—	—	—	—	-16.7	—	—	0.0	—	—	0.0	0.0	—	0.0	0.0	—	—	0.0	—	0.0	0.0	—
	Calories	Absolute	Mean	—	—	—	—	0.0	—	—	-8.6	—	—	-9.4	4.3	—	-10.3	0.6	—	—	0.0	—	11.9	0.0	—

				A. Lassonde	Agropur	Campbell Soup	Canada Bread	Canada Dry Mott's	Coca-Cola	Danone	General Mills	George Weston	Kellogg	Kraft Heinz	Loblaw	Maple Leaf Foods	Mondelēz	Nestlé	Ocean Spray	Parmalat	PepsiCo	Saputo	Sobeys	Sun-Rype	Unilever
	**(kcal per**	**change**	**Median**	—	—	—	—	**0.0**	—	—	**0.0**	—	—	**0.0**	**0.0**	—	**-5.5**	**0.0**	—	—	**0.0**	—	**0.0**	**0.0**	—
	**100 g/mL)**	**Percent**	**Mean**	—	—	—	—	**0.0**	—	—	**-2.4**	—	—	**-1.5**	**2.6**	—	**-2.0**	**0.2**	—	—	**0.0**	—	**3.4**	**0.0**	—
		**change**	**Median**	—	—	—	—	**0.0**	—	—	**0.0**	—	—	**0.0**	**0.0**	—	**-1.0**	**0.0**	—	—	**0.0**	—	**0.0**	**0.0**	—
	**Sodium**	**Absolute**	**Mean**	—	—	—	—	**16.7**	—	—	**16.7**	—	—	**-15.0**	**1.0**	—	**10.6**	**18.8**	—	—	**3.7**	—	**0.8**	**0.0**	—
	**(mg per**	**change**	**Median**	—	—	—	—	**16.7**	—	—	**20.3**	—	—	**0.0**	**0.0**	—	**3.2**	**0.0**	—	—	**0.0**	—	**0.0**	**0.0**	—
	**100 g/mL)**	**Percent**	**Mean**	—	—	—	—	**25.0**	—	—	**7.8**	—	—	**-33.3**	**4.4**	—	**9.7**	**64.6**	—	—	**0.0**	—	**-3.4**	**0.0**	—
		**change**	**Median**	—	—	—	—	**25.0**	—	—	**10.6**	—	—	**0.0**	**0.0**	—	**13.2**	**0.0**	—	—	**0.0**	—	**0.0**	**0.0**	—
	**Saturated**	**Absolute**	**Mean**	—	—	—	—	**0.0**	—	—	**0.5**	—	—	**-0.5**	**0.1**	—	**0.3**	**-0.2**	—	—	**0.0**	—	**-0.1**	**0.0**	—
	**fat (g per**	**change**	**Median**	—	—	—	—	**0.0**	—	—	**0.5**	—	—	**0.0**	**0.0**	—	**0.0**	**0.0**	—	—	**0.0**	—	**0.0**	**0.0**	—
	**100 g/mL)**	**Percent**	**Mean**	—	—	—	—	**N/A**	—	—	**33.3**	—	—	**2.1**	**2.5**	—	**2.9**	**-1.2**	—	—	**N/A**	—	**-12.5**	**N/A**	—
		**change**	**Median**	—	—	—	—	**N/A**	—	—	**25.0**	—	—	**0.0**	**0.0**	—	**0.0**	**0.0**	—	—	**N/A**	—	**-12.5**	**N/A**	—
	**Trans fat**	**Absolute**	**Mean**	—	—	—	—	**0.0**	—	—	**-1.4**	—	—	**0.0**	**0.0**	—	**0.0**	**0.0**	—	—	**0.0**	—	**0.0**	**0.0**	—
	**(g per 100**	**change**	**Median**	—	—	—	—	**0.0**	—	—	**-1.6**	—	—	**0.0**	**0.0**	—	**0.0**	**0.0**	—	—	**0.0**	—	**0.0**	**0.0**	—
	**g/mL)**	**Percent**	**Mean**	—	—	—	—	**N/A**	—	—	**-100.0**	—	—	**N/A**	**0.0**	—	**-7.5**	**30.1**	—	—	**N/A**	—	**N/A**	**N/A**	—
		**change**	**Median**	—	—	—	—	**N/A**	—	—	**-100.0**	—	—	**N/A**	**0.0**	—	**0.0**	**0.0**	—	—	**N/A**	—	**N/A**	**N/A**	—
	**Total**	**Absolute**	**Mean**	—	—	—	—	**10.0**	—	—	**-7.6**	—	—	**-1.0**	**1.5**	—	**1.9**	**-0.4**	—	—	**0.0**	—	**1.3**	**0.0**	—
	**sugars (g**	**change**	**Median**	—	—	—	—	**10.0**	—	—	**-4.3**	—	—	**0.0**	**0.0**	—	**1.2**	**0.0**	—	—	**0.0**	—	**0.0**	**0.0**	—
	**per 100**	**Percent**	**Mean**	—	—	—	—	**15.0**	—	—	**-14.9**	—	—	**-1.7**	**3.7**	—	**3.6**	**-0.4**	—	—	**0.0**	—	**2.6**	**0.0**	—
	**g/mL)**	**change**	**Median**	—	—	—	—	**15.0**	—	—	**-9.1**	—	—	**0.0**	**0.0**	—	**2.2**	**0.0**	—	—	**0.0**	—	**0.0**	**0.0**	—
	**Free**	**Absolute**	**Mean**	—	—	—	—	**-1.8**	—	—	**-29.0**	—	—	**-1.5**	**-4.8**	—	**1.4**	**-1.0**	—	—	**0.0**	—	**-1.4**	**37.4**	—
	**sugars (g**	**change**	**Median**	—	—	—	—	**-1.8**	—	—	**-27.7**	—	—	**-0.9**	**-0.7**	—	**0.3**	**-0.8**	—	—	**0.0**	—	**0.0**	**36.3**	—
	**per 100**	**Percent**	**Mean**	—	—	—	—	**-0.6**	—	—	**-60.3**	—	—	**-3.7**	**-11.0**	—	**2.5**	**-1.4**	—	—	**0.0**	—	**-2.1**	**135.2**	—
	**g/mL)**	**change**	**Median**	—	—	—	—	**-0.6**	—	—	**-61.0**	—	—	**-3.4**	**-1.2**	—	**0.7**	**-1.5**	—	—	**0.0**	—	**0.0**	**111.7**	—
Confectionary	Number of products			0	0	0	0	0	0	0	6	0	0	5	20	0	23	14	0	0	0	0	10	8	0
(U1, U3, U4,	HSR	Absolute	Mean	—	—	—	—	—	—	—	0.2	—	—	0.3	-0.1	—	0.0	0.0	—	—	—	—	0.1	0.0	—
U10, U11)		change	Median	—	—	—	—	—	—	—	0.0	—	—	0.0	0.0	—	0.0	0.0	—	—	—	—	0.0	0.0	—
		Percent	Mean	—	—	—	—	—	—	—	13.9	—	—	60.0	-4.2	—	-3.6	-2.4	—	—	—	—	10.0	0.0	—
		change (%)	Median	—	—	—	—	—	—	—	0.0	—	—	0.0	0.0	—	0.0	0.0	—	—	—	—	0.0	0.0	—
	Calories	Absolute	Mean	—	—	—	—	—	—	—	-8.6	—	—	-9.4	-2.3	—	-10.3	0.7	—	—	—	—	1.6	0.0	—
	(kcal per	change	Median	—	—	—	—	—	—	—	0.0	—	—	0.0	0.0	—	-5.5	0.0	—	—	—	—	0.0	0.0	—
	100 g/mL)	Percent	Mean	—	—	—	—	—	—	—	-2.4	—	—	-1.5	-0.4	—	-2.0	0.2	—	—	—	—	0.5	0.0	—
		change (%)	Median	—	—	—	—	—	—	—	0.0	—	—	0.0	0.0	—	-1.0	0.0	—	—	—	—	0.0	0.0	—
	Sodium	Absolute	Mean	—	—	—	—	—	—	—	16.7	—	—	-15.0	-1.3	—	10.6	15.3	—	—	—	—	-2.4	0.0	—
	(mg per	change	Median	—	—	—	—	—	—	—	20.3	—	—	0.0	0.0	—	3.2	0.0	—	—	—	—	0.0	0.0	—
	100 g/mL)	Percent	Mean	—	—	—	—	—	—	—	7.8	—	—	-33.3	-2.2	—	9.7	71.3	—	—	—	—	-11.1	0.0	—
		change (%)	Median	—	—	—	—	—	—	—	10.6	—	—	0.0	0.0	—	13.2	0.0	—	—	—	—	0.0	0.0	—
	Saturated	Absolute	Mean	—	—	—	—	—	—	—	0.5	—	—	-0.5	0.2	—	0.3	-0.3	—	—	—	—	0.0	0.0	—
	fat (g per	change	Median	—	—	—	—	—	—	—	0.5	—	—	0.0	0.0	—	0.0	0.0	—	—	—	—	0.0	0.0	—
	100 g/mL)	Percent	Mean	—	—	—	—	—	—	—	33.3	—	—	2.1	1.1	—	2.9	-1.2	—	—	—	—	0.0	N/A	—
		change (%)	Median	—	—	—	—	—	—	—	25.0	—	—	0.0	0.0	—	0.0	0.0	—	—	—	—	0.0	N/A	—
	Trans fat (g	Absolute	Mean	—	—	—	—	—	—	—	-1.4	—	—	0.0	0.0	—	0.0	0.1	—	—	—	—	0.0	0.0	—
	per 100	change	Median	—	—	—	—	—	—	—	-1.6	—	—	0.0	0.0	—	0.0	0.0	—	—	—	—	0.0	0.0	—
	g/mL)	Percent	Mean	—	—	—	—	—	—	—	-100.0	—	—	N/A	0.0	—	-7.5	30.1	—	—	—	—	N/A	N/A	—
		change (%)	Median	—	—	—	—	—	—	—	-100.0	—	—	N/A	0.0	—	0.0	0.0	—	—	—	—	N/A	N/A	—
	Total	Absolute	Mean	—	—	—	—	—	—	—	-7.6	—	—	-1.0	0.2	—	1.9	-0.1	—	—	—	—	-0.7	0.0	—
	sugars (g	change	Median	—	—	—	—	—	—	—	-4.3	—	—	0.0	0.0	—	1.2	0.0	—	—	—	—	0.0	0.0	—
	per 100	Percent	Mean	—	—	—	—	—	—	—	-14.9	—	—	-1.7	0.4	—	3.6	-0.1	—	—	—	—	0.1	0.0	—
	g/mL)	change (%)	Median	—	—	—	—	—	—	—	-9.1	—	—	0.0	0.0	—	2.2	0.0	—	—	—	—	0.0	0.0	—
	Free sugars	Absolute	Mean	—	—	—	—	—	—	—	-29.0	—	—	-1.5	1.7	—	1.4	-0.8	—	—	—	—	-0.7	37.4	—
	(g per 100	change	Median	—	—	—	—	—	—	—	-27.7	—	—	-0.9	-0.7	—	0.3	-0.7	—	—	—	—	0.0	36.3	—
	g/mL)	Percent	Mean	—	—	—	—	—	—	—	-60.3	—	—	-3.7	-1.4	—	2.5	-1.4	—	—	—	—	0.1	135.2	—
		change (%)	Median	—	—	—	—	—	—	—	-61.0	—	—	-3.4	-1.2	—	0.7	-1.1	—	—	—	—	0.0	111.7	—
Sugars and	Number of products			0	0	0	0	2	0	0	0	0	0	0	52	0	0	4	0	0	3	0	14	0	0
syrups (U8,	HSR	Absolute	Mean	—	—	—	—	-0.3	—	—	—	—	—	—	0.0	—	—	0.0	—	—	0.0	—	-0.1	—	—
U9, U12, U14,		change	Median	—	—	—	—	-0.3	—	—	—	—	—	—	0.0	—	—	0.0	—	—	0.0	—	0.0	—	—
U15)		Percent	Mean	—	—	—	—	-16.7	—	—	—	—	—	—	-1.0	—	—	0.0	—	—	0.0	—	-5.4	—	—
		change (%)	Median	—	—	—	—	-16.7	—	—	—	—	—	—	0.0	—	—	0.0	—	—	0.0	—	0.0	—	—
	Calories	Absolute	Mean	—	—	—	—	0.0	—	—	—	—	—	—	6.9	—	—	0.0	—	—	0.0	—	19.2	—	—
	(kcal per	change	Median	—	—	—	—	0.0	—	—	—	—	—	—	0.0	—	—	-16.7	—	—	0.0	—	8.3	—	—
	100 g/mL)	Percent	Mean	—	—	—	—	0.0	—	—	—	—	—	—	3.8	—	—	0.0	—	—	0.0	—	5.5	—	—
		change (%)	Median	—	—	—	—	0.0	—	—	—	—	—	—	0.0	—	—	-4.8	—	—	0.0	—	2.4	—	—
	Sodium	Absolute	Mean	—	—	—	—	16.7	—	—	—	—	—	—	1.8	—	—	31.3	—	—	3.7	—	3.1	—	—
	(mg per	change	Median	—	—	—	—	16.7	—	—	—	—	—	—	0.0	—	—	41.7	—	—	0.0	—	0.0	—	—
	100 g/mL)	Percent	Mean	—	—	—	—	25.0	—	—	—	—	—	—	9.7	—	—	33.3	—	—	0.0	—	8.1	—	—
		change (%)	Median	—	—	—	—	25.0	—	—	—	—	—	—	0.0	—	—	33.3	—	—	0.0	—	4.2	—	—
	Saturated	Absolute	Mean	—	—	—	—	0.0	—	—	—	—	—	—	0.0	—	—	0.0	—	—	0.0	—	-0.2	—	—
	fat (g per	change	Median	—	—	—	—	0.0	—	—	—	—	—	—	0.0	—	—	0.0	—	—	0.0	—	0.0	—	—
	100 g/mL)	Percent	Mean	—	—	—	—	N/A	—	—	—	—	—	—	26.3	—	—	N/A	—	—	N/A	—	-25.0	—	—
		change (%)	Median	—	—	—	—	N/A	—	—	—	—	—	—	26.3	—	—	N/A	—	—	N/A	—	-25.0	—	—
	Trans fat (g	Absolute	Mean	—	—	—	—	0.0	—	—	—	—	—	—	0.0	—	—	0.0	—	—	0.0	—	0.0	—	—
	per 100	change	Median	—	—	—	—	0.0	—	—	—	—	—	—	0.0	—	—	0.0	—	—	0.0	—	0.0	—	—
	g/mL)	Percent	Mean	—	—	—	—	N/A	—	—	—	—	—	—	N/A	—	—	N/A	—	—	N/A	—	N/A	—	—
		change (%)	Median	—	—	—	—	N/A	—	—	—	—	—	—	N/A	—	—	N/A	—	—	N/A	—	N/A	—	—

				A. Lassonde	Agropur	Campbell Soup	Canada Bread	Canada Dry Mott's	Coca-Cola	Danone	General Mills	George Weston	Kellogg	Kraft Heinz	Loblaw	Maple Leaf Foods	Mondelēz	Nestlé	Ocean Spray	Parmalat	PepsiCo	Saputo	Sobeys	Sun-Rype	Unilever
	Total sugars (g per 100 g/mL)	Absolute change	Mean	—	—	—	—	10.0	—	—	—	—	—	—	2.1	—	—	-1.7	—	—	0.0	—	2.8	—	—
			Median	—	—	—	—	10.0	—	—	—	—	—	—	0.0	—	—	-6.7	—	—	0.0	—	0.0	—	—
		Percent change (%)	Mean	—	—	—	—	15.0	—	—	—	—	—	—	5.0	—	—	-1.4	—	—	0.0	—	4.4	—	—
			Median	—	—	—	—	15.0	—	—	—	—	—	—	0.0	—	—	-8.3	—	—	0.0	—	0.0	—	—
	Free sugars (g per 100 g/mL)	Absolute change	Mean	—	—	—	—	-1.8	—	—	—	—	—	—	-7.4	—	—	-1.7	—	—	0.0	—	-2.0	—	—
			Median	—	—	—	—	-1.8	—	—	—	—	—	—	-3.2	—	—	-6.7	—	—	0.0	—	0.0	—	—
		Percent change (%)	Mean	—	—	—	—	-0.6	—	—	—	—	—	—	-14.6	—	—	-1.4	—	—	0.0	—	-3.6	—	—
			Median	—	—	—	—	-0.6	—	—	—	—	—	—	-6.4	—	—	-8.3	—	—	0.0	—	0.0	—	—
**Vegetables (V)**	**Number of products**			**1**	**0**	**5**	**0**	**5**	**0**	**0**	**4**	**0**	**0**	**1**	**108**	**0**	**0**	**0**	**0**	**0**	**0**	**0**	**68**	**0**	**1**
	**HSR**	**Absolute change**	**Mean**	**0.5**	—	**0.0**	—	**0.0**	—	—	**0.0**	—	—	**0.0**	**0.0**	—	—	—	—	—	—	—	**0.0**	—	**0.0**
			**Median**	**0.5**	—	**0.0**	—	**0.0**	—	—	**0.0**	—	—	**0.0**	**0.0**	—	—	—	—	—	—	—	**0.0**	—	**0.0**
		**Percent change**	**Mean**	**25.0**	—	**0.0**	—	**0.0**	—	—	**0.0**	—	—	**0.0**	**2.4**	—	—	—	—	—	—	—	**0.1**	—	**0.0**
			**Median**	**25.0**	—	**0.0**	—	**0.0**	—	—	**0.0**	—	—	**0.0**	**0.0**	—	—	—	—	—	—	—	**0.0**	—	**0.0**
	**Calories (kcal per 100 g/mL)**	**Absolute change**	**Mean**	**0.7**	—	**0.0**	—	**0.0**	—	—	**0.0**	—	—	**0.0**	**0.1**	—	—	—	—	—	—	—	**-0.1**	—	**-16.7**
			**Median**	**0.7**	—	**0.0**	—	**0.0**	—	—	**0.0**	—	—	**0.0**	**0.0**	—	—	—	—	—	—	—	**0.0**	—	**-16.7**
		**Percent change**	**Mean**	**2.9**	—	**0.0**	—	**0.0**	—	—	**0.0**	—	—	**0.0**	**0.9**	—	—	—	—	—	—	—	**-0.3**	—	**-100.0**
			**Median**	**2.9**	—	**0.0**	—	**0.0**	—	—	**0.0**	—	—	**0.0**	**0.0**	—	—	—	—	—	—	—	**0.0**	—	**-100.0**
	**Sodium (mg per 100 g/mL)**	**Absolute change**	**Mean**	**-15.3**	—	**0.0**	—	**0.0**	—	—	**0.0**	—	—	**0.0**	**-50.6**	—	—	—	—	—	—	—	**-17.4**	—	**-233.3**
			**Median**	**-15.3**	—	**0.0**	—	**0.0**	—	—	**0.0**	—	—	**0.0**	**0.0**	—	—	—	—	—	—	—	**0.0**	—	**-233.3**
		**Percent change**	**Mean**	**-8.4**	—	**0.0**	—	**0.0**	—	—	**0.0**	—	—	**0.0**	**3.7**	—	—	—	—	—	—	—	**-6.2**	—	**-30.4**
			**Median**	**-8.4**	—	**0.0**	—	**0.0**	—	—	**0.0**	—	—	**0.0**	**0.0**	—	—	—	—	—	—	—	**0.0**	—	**-30.4**
	**Saturated fat (g per 100 g/mL)**	**Absolute change**	**Mean**	**0.0**	—	**0.0**	—	**0.0**	—	—	**0.0**	—	—	**0.0**	**0.0**	—	—	—	—	—	—	—	**0.0**	—	**0.0**
			**Median**	**0.0**	—	**0.0**	—	**0.0**	—	—	**0.0**	—	—	**0.0**	**0.0**	—	—	—	—	—	—	—	**0.0**	—	**0.0**
		**Percent change**	**Mean**	**N/A**	—	**-100.0**	—	**N/A**	—	—	**N/A**	—	—	**N/A**	**2.8**	—	—	—	—	—	—	—	**0.0**	—	**N/A**
			**Median**	**N/A**	—	**-100.0**	—	**N/A**	—	—	**N/A**	—	—	**N/A**	**0.0**	—	—	—	—	—	—	—	**0.0**	—	**N/A**
	**Trans fat (g per 100 g/mL)**	**Absolute change**	**Mean**	**0.0**	—	**0.0**	—	**0.0**	—	—	**0.0**	—	—	**0.0**	**0.0**	—	—	—	—	—	—	—	**0.0**	—	**0.0**
			**Median**	**0.0**	—	**0.0**	—	**0.0**	—	—	**0.0**	—	—	**0.0**	**0.0**	—	—	—	—	—	—	—	**0.0**	—	**0.0**
		**Percent change**	**Mean**	**N/A**	—	**N/A**	—	**N/A**	—	—	**N/A**	—	—	**N/A**	**N/A**	—	—	—	—	—	—	—	**N/A**	—	**N/A**
			**Median**	**N/A**	—	**N/A**	—	**N/A**	—	—	**N/A**	—	—	**N/A**	**N/A**	—	—	—	—	—	—	—	**N/A**	—	**N/A**
	**Total sugars (g per 100 g/mL)**	**Absolute change**	**Mean**	**0.1**	—	**-0.3**	—	**0.0**	—	—	**0.0**	—	—	**0.0**	**-0.3**	—	—	—	—	—	—	—	**0.1**	—	**0.0**
			**Median**	**0.1**	—	**0.0**	—	**0.0**	—	—	**0.0**	—	—	**0.0**	**0.0**	—	—	—	—	—	—	—	**0.0**	—	**0.0**
		**Percent change**	**Mean**	**2.9**	—	**-8.1**	—	**0.0**	—	—	**0.0**	—	—	**0.0**	**-3.7**	—	—	—	—	—	—	—	**5.5**	—	**N/A**
			**Median**	**2.9**	—	**0.0**	—	**0.0**	—	—	**0.0**	—	—	**0.0**	**0.0**	—	—	—	—	—	—	—	**0.0**	—	**N/A**
	**Free sugars (g per 100 g/mL)**	**Absolute change**	**Mean**	**0.0**	—	**-0.5**	—	**-1.3**	—	—	**0.0**	—	—	**0.0**	**-0.3**	—	—	—	—	—	—	—	**-0.1**	—	**0.0**
			**Median**	**0.0**	—	**0.0**	—	**-1.5**	—	—	**0.0**	—	—	**0.0**	**0.0**	—	—	—	—	—	—	—	**0.0**	—	**0.0**
		**Percent change**	**Mean**	**N/A**	—	**-100.0**	—	**-100.0**	—	—	**N/A**	—	—	**N/A**	**-0.4**	—	—	—	—	—	—	—	**-0.1**	—	**N/A**
			**Median**	**N/A**	—	**-100.0**	—	**-100.0**	—	—	**N/A**	—	—	**N/A**	**-5.2**	—	—	—	—	—	—	—	**-4.0**	—	**N/A**
Vegetable juice and vegetable drink (V7)	Number of products			1	0	4	0	5	0	0	0	0	0	1	6	0	0	0	0	0	0	0	5	0	0
	HSR	Absolute change	Mean	0.5	—	0.0	—	0.0	—	—	—	—	—	0.0	0.3	—	—	—	—	—	—	—	-0.2	—	—
			Median	0.5	—	0.0	—	0.0	—	—	—	—	—	0.0	0.5	—	—	—	—	—	—	—	0.0	—	—
		Percent change (%)	Mean	25.0	—	0.0	—	0.0	—	—	—	—	—	0.0	27.8	—	—	—	—	—	—	—	-13.3	—	—
			Median	25.0	—	0.0	—	0.0	—	—	—	—	—	0.0	33.3	—	—	—	—	—	—	—	0.0	—	—
	Calories (kcal per 100 g/mL)	Absolute change	Mean	0.7	—	0.0	—	0.0	—	—	—	—	—	0.0	0.0	—	—	—	—	—	—	—	1.2	—	—
			Median	0.7	—	0.0	—	0.0	—	—	—	—	—	0.0	0.0	—	—	—	—	—	—	—	2.0	—	—
		Percent change (%)	Mean	2.9	—	0.0	—	0.0	—	—	—	—	—	0.0	0.6	—	—	—	—	—	—	—	5.6	—	—
			Median	2.9	—	0.0	—	0.0	—	—	—	—	—	0.0	0.0	—	—	—	—	—	—	—	11.1	—	—
	Sodium (mg per 100 g/mL)	Absolute change	Mean	-15.3	—	0.0	—	0.0	—	—	—	—	—	0.0	-43.0	—	—	—	—	—	—	—	15.2	—	—
			Median	-15.3	—	0.0	—	0.0	—	—	—	—	—	0.0	-17.0	—	—	—	—	—	—	—	4.0	—	—
		Percent change (%)	Mean	-8.4	—	0.0	—	0.0	—	—	—	—	—	0.0	-15.5	—	—	—	—	—	—	—	4.5	—	—
			Median	-8.4	—	0.0	—	0.0	—	—	—	—	—	0.0	-12.0	—	—	—	—	—	—	—	1.7	—	—
	Saturated fat (g per 100 g/mL)	Absolute change	Mean	0.0	—	0.0	—	0.0	—	—	—	—	—	0.0	0.0	—	—	—	—	—	—	—	0.0	—	—
			Median	0.0	—	0.0	—	0.0	—	—	—	—	—	0.0	0.0	—	—	—	—	—	—	—	0.0	—	—
		Percent change (%)	Mean	N/A	—	-100.0	—	N/A	—	—	—	—	—	N/A	N/A	—	—	—	—	—	—	—	N/A	—	—
			Median	N/A	—	-100.0	—	N/A	—	—	—	—	—	N/A	N/A	—	—	—	—	—	—	—	N/A	—	—
	Trans fat (g per 100 g/mL)	Absolute change	Mean	0.0	—	0.0	—	0.0	—	—	—	—	—	0.0	0.0	—	—	—	—	—	—	—	0.0	—	—
			Median	0.0	—	0.0	—	0.0	—	—	—	—	—	0.0	0.0	—	—	—	—	—	—	—	0.0	—	—
		Percent change (%)	Mean	N/A	—	N/A	—	N/A	—	—	—	—	—	N/A	N/A	—	—	—	—	—	—	—	N/A	—	—
			Median	N/A	—	N/A	—	N/A	—	—	—	—	—	N/A	N/A	—	—	—	—	—	—	—	N/A	—	—
	Total sugars (g per 100 g/mL)	Absolute change	Mean	0.1	—	-0.4	—	0.0	—	—	—	—	—	0.0	0.0	—	—	—	—	—	—	—	0.5	—	—
			Median	0.1	—	-0.4	—	0.0	—	—	—	—	—	0.0	0.0	—	—	—	—	—	—	—	0.8	—	—
		Percent change (%)	Mean	2.9	—	-10.1	—	0.0	—	—	—	—	—	0.0	0.6	—	—	—	—	—	—	—	17.7	—	—
			Median	2.9	—	-9.1	—	0.0	—	—	—	—	—	0.0	0.0	—	—	—	—	—	—	—	33.3	—	—
	Free sugars (g per 100 g/mL)	Absolute change	Mean	0.0	—	-0.6	—	-1.3	—	—	—	—	—	0.0	-0.3	—	—	—	—	—	—	—	-0.6	—	—
			Median	0.0	—	-0.3	—	-1.5	—	—	—	—	—	0.0	-0.1	—	—	—	—	—	—	—	-0.6	—	—
		Percent change (%)	Mean	N/A	—	-100.0	—	-100.0	—	—	—	—	—	N/A	-100.0	—	—	—	—	—	—	—	-100.0	—	—
			Median	N/A	—	-100.0	—	-100.0	—	—	—	—	—	N/A	-100.0	—	—	—	—	—	—	—	-100.0	—	—
Vegetables with sauce, pastes (V2, V9, V10, V11, V12)	Number of products			0	0	0	0	0	0	0	0	0	0	0	27	0	0	0	0	0	0	0	12	0	1
	HSR	Absolute change	Mean	—	—	—	—	—	—	—	—	—	—	—	0.1	—	—	—	—	—	—	—	0.0	—	0.0
			Median	—	—	—	—	—	—	—	—	—	—	—	0.0	—	—	—	—	—	—	—	0.0	—	0.0
		Percent change (%)	Mean	—	—	—	—	—	—	—	—	—	—	—	4.4	—	—	—	—	—	—	—	0.0	—	0.0
			Median	—	—	—	—	—	—	—	—	—	—	—	0.0	—	—	—	—	—	—	—	0.0	—	0.0
	Calories (kcal per	Absolute change	Mean	—	—	—	—	—	—	—	—	—	—	—	-0.1	—	—	—	—	—	—	—	-0.3	—	-16.7
			Median	—	—	—	—	—	—	—	—	—	—	—	0.0	—	—	—	—	—	—	—	0.0	—	-16.7

				A. Lassonde	Agropur	Campbell Soup	Canada Bread	Canada Dry Mott's	Coca-Cola	Danone	General Mills	George Weston	Kellogg	Kraft Heinz	Loblaw	Maple Leaf Foods	Mondelēz	Nestlé	Ocean Spray	Parmalat	PepsiCo	Saputo	Sobeys	Sun-Rype	Unilever
	100 g/mL)	Percent	Mean	—	—	—	—	—	—	—	—	—	—	—	1.0	—	—	—	—	—	—	—	-1.0	—	-100.0
		change (%)	Median	—	—	—	—	—	—	—	—	—	—	—	0.0	—	—	—	—	—	—	—	0.0	—	-100.0
	Sodium	Absolute	Mean	—	—	—	—	—	—	—	—	—	—	—	-53.1	—	—	—	—	—	—	—	-6.7	—	-233.3
	(mg per	change	Median	—	—	—	—	—	—	—	—	—	—	—	0.0	—	—	—	—	—	—	—	0.0	—	-233.3
	100 g/mL)	Percent	Mean	—	—	—	—	—	—	—	—	—	—	—	-6.0	—	—	—	—	—	—	—	8.0	—	-30.4
		change (%)	Median	—	—	—	—	—	—	—	—	—	—	—	0.0	—	—	—	—	—	—	—	0.0	—	-30.4
	Saturated	Absolute	Mean	—	—	—	—	—	—	—	—	—	—	—	0.0	—	—	—	—	—	—	—	0.0	—	0.0
	fat (g per	change	Median	—	—	—	—	—	—	—	—	—	—	—	0.0	—	—	—	—	—	—	—	0.0	—	0.0
	100 g/mL)	Percent	Mean	—	—	—	—	—	—	—	—	—	—	—	18.4	—	—	—	—	—	—	—	N/A	—	N/A
		change (%)	Median	—	—	—	—	—	—	—	—	—	—	—	18.4	—	—	—	—	—	—	—	N/A	—	N/A
	Trans fat (g	Absolute	Mean	—	—	—	—	—	—	—	—	—	—	—	0.0	—	—	—	—	—	—	—	0.0	—	0.0
	per 100	change	Median	—	—	—	—	—	—	—	—	—	—	—	0.0	—	—	—	—	—	—	—	0.0	—	0.0
	g/mL)	Percent	Mean	—	—	—	—	—	—	—	—	—	—	—	N/A	—	—	—	—	—	—	—	N/A	—	N/A
		change (%)	Median	—	—	—	—	—	—	—	—	—	—	—	N/A	—	—	—	—	—	—	—	N/A	—	N/A
	Total	Absolute	Mean	—	—	—	—	—	—	—	—	—	—	—	-1.2	—	—	—	—	—	—	—	0.1	—	0.0
	sugars (g	change	Median	—	—	—	—	—	—	—	—	—	—	—	0.0	—	—	—	—	—	—	—	0.0	—	0.0
	per 100	Percent	Mean	—	—	—	—	—	—	—	—	—	—	—	-19.2	—	—	—	—	—	—	—	-5.6	—	N/A
	g/mL)	change (%)	Median	—	—	—	—	—	—	—	—	—	—	—	0.0	—	—	—	—	—	—	—	0.0	—	N/A
	Free sugars	Absolute	Mean	—	—	—	—	—	—	—	—	—	—	—	-1.2	—	—	—	—	—	—	—	-0.5	—	0.0
	(g per 100	change	Median	—	—	—	—	—	—	—	—	—	—	—	0.0	—	—	—	—	—	—	—	-0.4	—	0.0
	g/mL)	Percent	Mean	—	—	—	—	—	—	—	—	—	—	—	-16.6	—	—	—	—	—	—	—	-4.2	—	N/A
		change (%)	Median	—	—	—	—	—	—	—	—	—	—	—	-6.6	—	—	—	—	—	—	—	-3.7	—	N/A
Vegetables	Number of products			0	0	1	0	0	0	0	4	0	0	0	75	0	0	0	0	0	0	0	51	0	0
without sauce	HSR	Absolute	Mean	—	—	0.0	—	—	—	—	0.0	—	—	—	0.0	—	—	—	—	—	—	—	0.1	—	—
(V1, V3, V4,		change	Median	—	—	0.0	—	—	—	—	0.0	—	—	—	0.0	—	—	—	—	—	—	—	0.0	—	—
V5, V8)		Percent	Mean	—	—	0.0	—	—	—	—	0.0	—	—	—	-0.4	—	—	—	—	—	—	—	1.4	—	—
		change (%)	Median	—	—	0.0	—	—	—	—	0.0	—	—	—	0.0	—	—	—	—	—	—	—	0.0	—	—
	Calories	Absolute	Mean	—	—	0.0	—	—	—	—	0.0	—	—	—	0.2	—	—	—	—	—	—	—	-0.2	—	—
	(kcal per	change	Median	—	—	0.0	—	—	—	—	0.0	—	—	—	0.0	—	—	—	—	—	—	—	0.0	—	—
	100 g/mL)	Percent	Mean	—	—	0.0	—	—	—	—	0.0	—	—	—	0.9	—	—	—	—	—	—	—	-0.7	—	—
		change (%)	Median	—	—	0.0	—	—	—	—	0.0	—	—	—	0.0	—	—	—	—	—	—	—	0.0	—	—
	Sodium	Absolute	Mean	—	—	0.0	—	—	—	—	0.0	—	—	—	-50.4	—	—	—	—	—	—	—	-23.1	—	—
	(mg per	change	Median	—	—	0.0	—	—	—	—	0.0	—	—	—	0.0	—	—	—	—	—	—	—	0.0	—	—
	100 g/mL)	Percent	Mean	—	—	0.0	—	—	—	—	0.0	—	—	—	9.0	—	—	—	—	—	—	—	-11.3	—	—
		change (%)	Median	—	—	0.0	—	—	—	—	0.0	—	—	—	0.0	—	—	—	—	—	—	—	0.0	—	—
	Saturated	Absolute	Mean	—	—	0.0	—	—	—	—	0.0	—	—	—	0.1	—	—	—	—	—	—	—	0.0	—	—
	fat (g per	change	Median	—	—	0.0	—	—	—	—	0.0	—	—	—	0.0	—	—	—	—	—	—	—	0.0	—	—
	100 g/mL)	Percent	Mean	—	—	N/A	—	—	—	—	N/A	—	—	—	1.0	—	—	—	—	—	—	—	0.0	—	—
		change (%)	Median	—	—	N/A	—	—	—	—	N/A	—	—	—	0.0	—	—	—	—	—	—	—	0.0	—	—
	Trans fat (g	Absolute	Mean	—	—	0.0	—	—	—	—	0.0	—	—	—	0.0	—	—	—	—	—	—	—	0.0	—	—
	per 100	change	Median	—	—	0.0	—	—	—	—	0.0	—	—	—	0.0	—	—	—	—	—	—	—	0.0	—	—
	g/mL)	Percent	Mean	—	—	N/A	—	—	—	—	N/A	—	—	—	N/A	—	—	—	—	—	—	—	N/A	—	—
		change (%)	Median	—	—	N/A	—	—	—	—	N/A	—	—	—	N/A	—	—	—	—	—	—	—	N/A	—	—
	Total	Absolute	Mean	—	—	0.0	—	—	—	—	0.0	—	—	—	0.0	—	—	—	—	—	—	—	0.0	—	—
	sugars (g	change	Median	—	—	0.0	—	—	—	—	0.0	—	—	—	0.0	—	—	—	—	—	—	—	0.0	—	—
	per 100	Percent	Mean	—	—	0.0	—	—	—	—	0.0	—	—	—	1.0	—	—	—	—	—	—	—	6.4	—	—
	g/mL)	change (%)	Median	—	—	0.0	—	—	—	—	0.0	—	—	—	0.0	—	—	—	—	—	—	—	0.0	—	—
	Free sugars	Absolute	Mean	—	—	0.0	—	—	—	—	0.0	—	—	—	0.0	—	—	—	—	—	—	—	0.0	—	—
	(g per 100	change	Median	—	—	0.0	—	—	—	—	0.0	—	—	—	0.0	—	—	—	—	—	—	—	0.0	—	—
	g/mL)	Percent	Mean	—	—	N/A	—	—	—	—	N/A	—	—	—	33.8	—	—	—	—	—	—	—	40.6	—	—
		change (%)	Median	—	—	N/A	—	—	—	—	N/A	—	—	—	9.6	—	—	—	—	—	—	—	6.5	—	—

[1]Food categories are based on those defined in Health Canada's Table of Reference Amounts for Foods (TRA), listed in brackets. Values at the TRA major food category level are shown in bold. TRA minor food categories that were combined are indicated in brackets. If none of the sampled products fell into that food category, it was omitted from the table. N/A indicates that percentage change could not be determined when the mean and/or median nutrient value per 100 g/mL in 2013 was 0 g/mL.

Supplementary Table 4. Mean Health Star Ratings and median amounts of calories, sodium, saturated fat, trans fat, total sugars and free sugars per 100 g (or mL) in products offered by each company in 2013, presented overall and by food category. [1]

Category	Measure	Statistic	A. Lassonde	Agropur	Campbell Soup	Canada Bread	Canada Dry Mott's	Coca-Cola	Danone	General Mills	George Weston	Kellogg	Kraft Heinz	Loblaw	Maple Leaf Foods	Mondelēz	Nestlé	Ocean Spray	Parmalat	PepsiCo	Saputo	Sobeys	Sun-Rype	Unilever
Total product portfolio	Number of products		55	99	197	101	50	83	63	300	104	129	612	2260	178	45	277	32	72	267	78	1248	40	200
	Health Star Rating	Mean	2.3	3.6	3.1	3.5	2.3	2.3	3.9	2.9	3.3	2.9	2.2	2.9	2.3	1.2	2.4	2.5	3.3	2.7	2.3	2.8	2.6	2.8
		SD	1.1	1.4	0.8	0.7	1.3	1.4	0.9	1.3	0.9	1.1	1.1	1.2	1.1	0.9	1.0	1.0	1.3	1.1	1.6	1.2	1.0	0.8
		≥3.5 (n)	7	61	126	81	17	17	35	126	72	44	136	963	54	1	70	4	35	55	25	521	7	53
		≥3.5 (%)	12.7	61.6	64.0	80.2	34.0	20.5	55.6	42.0	69.2	34.1	22.2	42.6	30.3	2.2	25.3	12.5	48.6	20.6	32.1	41.7	17.5	26.5
	Calories per 100 g/mL	Median	44.0	100.0	48.0	253.3	44.4	45.1	90.0	238.0	266.7	392.9	266.7	233.3	228.6	482.8	136.0	52.0	112.0	409.1	233.3	217.9	52.0	108.6
		IQR	4.0	231.7	32.0	27.5	28.8	16.5	46.4	297.6	48.9	57.2	324.0	301.3	168.5	142.9	147.0	24.0	320.0	330.2	347.1	286.7	276.3	174.1
	Sodium per 100 g/mL	Median	10.0	66.7	256.0	395.3	14.8	7.5	45.0	271.4	449.1	400.0	500.0	274.8	811.8	76.9	78.9	14.0	60.0	400.0	298.9	262.0	11.0	260.0
		IQR	8.0	437.8	128.0	164.4	29.4	10.0	10.7	401.1	187.9	294.1	616.7	493.3	352.6	161.9	194.3	12.8	616.7	603.7	386.6	503.0	25.9	388.5
	Saturated fat per 100	Median	0.0	1.7	0.2	0.7	0.0	0.0	1.0	1.4	0.9	1.4	2.6	1.2	4.9	11.7	2.8	0.0	2.0	1.9	6.3	1.0	0.0	0.8
		IQR	0.0	9.2	0.6	1.0	0.0	0.0	1.4	3.6	1.0	3.0	6.2	5.0	7.2	13.3	5.0	0.0	14.1	3.5	11.4	4.3	0.0	3.6
	Trans fat per 100	Median	0.0	0.1	0.0	0.0	0.0	0.0	0.0	0.0	0.0	0.0	0.0	0.0	0.0	0.0	0.1	0.0	0.0	0.0	0.1	0.0	0.0	0.0
		IQR	0.0	0.3	0.0	0.0	0.0	0.0	0.0	0.2	0.0	0.0	0.2	0.1	0.2	0.2	0.2	0.0	1.0	0.0	0.3	0.1	0.0	0.1
	Total sugars per 100	Median	10.0	4.4	1.6	3.0	9.9	10.0	10.8	10.0	3.6	20.0	6.7	4.0	1.1	52.1	11.2	13.2	4.8	5.7	10.6	4.8	12.4	3.0
		IQR	0.8	10.8	4.4	2.7	6.9	3.8	7.0	20.9	2.9	20.0	15.6	14.0	3.0	13.8	14.7	6.1	12.0	17.7	27.9	14.3	64.1	12.8
	Free sugars per 100	Median	10.0	0.0	0.6	1.2	4.5	10.0	7.2	6.7	2.1	18.4	4.5	1.0	0.0	52.1	8.1	12.8	0.0	4.0	6.3	1.8	12.4	1.2
		IQR	0.8	8.1	3.7	2.8	10.6	3.8	7.9	20.8	3.0	23.3	14.0	11.6	2.5	13.8	12.8	10.8	8.9	18.1	30.2	12.3	20.6	9.0
Bakery products (A)	Number of products		0	2	23	78	0	0	0	60	103	68	130	308	0	11	1	0	0	53	27	194	2	0
	Health Star Rating	Mean	—	3.3	2.0	3.5	—	—	—	2.1	3.3	2.6	1.8	2.0	—	2.0	0.5	—	—	2.4	0.9	2.1	3.5	—
		SD	—	1.1	0.8	0.7	—	—	—	0.8	0.9	1.0	0.9	1.0	—	1.2	N/A	—	—	0.9	0.4	0.9	0.0	—
	Calories per 100 g/mL	Median	—	386.4	450.0	253.3	—	—	—	400.0	266.7	400.0	462.9	428.6	—	480.0	526.3	—	—	428.6	449.0	392.9	366.7	—
		IQR	—	22.7	50.0	23.7	—	—	—	121.0	48.7	54.5	61.2	111.9	—	60.5	0.0	—	—	33.3	31.9	119.0	0.0	—
	Sodium per 100 g/mL	Median	—	454.5	650.0	416.6	—	—	—	341.1	450.0	381.7	482.8	382.4	—	250.0	78.9	—	—	269.2	359.4	335.3	58.3	—
		IQR	—	45.5	362.5	161.2	—	—	—	243.1	184.0	224.6	442.3	357.1	—	83.1	0.0	—	—	214.3	130.8	288.4	25.0	—
	Saturated fat per 100	Median	—	3.4	2.5	0.7	—	—	—	5.5	0.9	2.4	5.0	4.0	—	4.0	18.4	—	—	2.4	12.2	3.8	1.7	—
		IQR	—	1.1	3.0	0.3	—	—	—	3.6	1.0	2.7	5.1	6.5	—	9.2	0.0	—	—	2.9	6.8	6.0	1.7	—
	Trans fat per 100	Median	—	0.0	0.0	0.0	—	—	—	0.0	0.0	0.0	0.0	0.0	—	0.0	0.0	—	—	0.0	0.2	0.0	0.0	—
		IQR	—	0.0	0.0	0.0	—	—	—	0.6	0.0	0.0	0.0	0.2	—	0.4	0.0	—	—	0.0	0.1	0.2	0.0	—
	Total sugars per 100	Median	—	4.5	7.1	2.9	—	—	—	29.2	3.6	25.5	17.6	21.7	—	24.1	36.8	—	—	22.9	43.8	22.9	55.0	—
		IQR	—	0.0	25.8	3.0	—	—	—	18.8	2.8	22.2	27.1	26.8	—	17.7	0.0	—	—	17.4	18.3	21.9	1.7	—
	Free sugars per 100	Median	—	3.2	6.8	1.6	—	—	—	24.4	2.1	22.9	17.3	19.3	—	24.1	36.8	—	—	21.4	37.9	20.2	51.5	—
		IQR	—	0.0	25.8	2.7	—	—	—	22.1	3.0	23.6	27.6	25.5	—	19.2	0.0	—	—	20.8	21.9	23.1	1.5	—
Baked goods, pastries and other sweetened bakery products (A4, A5, A6, A7, A8, A9, A10, A14, A15, A17, A22, A23)	Number of products		0	0	7	6	0	0	0	20	6	23	67	167	0	9	1	0	0	4	25	111	0	0
	Health Star Rating	Mean	—	—	1.1	1.8	—	—	—	1.6	1.6	2.1	1.5	1.6	—	1.6	0.5	—	—	3.1	0.8	1.8	—	—
		SD	—	—	0.6	0.3	—	—	—	0.6	0.2	0.7	0.8	0.8	—	0.9	N/A	—	—	0.3	0.4	0.8	—	—
	Calories per 100 g/mL	Median	—	—	500.0	476.2	—	—	—	381.8	353.8	400.0	464.3	415.4	—	480.0	526.3	—	—	238.1	449.0	384.6	—	—
		IQR	—	—	38.6	53.6	—	—	—	130.4	22.8	128.6	48.5	124.3	—	82.8	0.0	—	—	5.1	35.7	128.6	—	—
	Sodium per 100 g/mL	Median	—	—	200.0	301.9	—	—	—	336.3	410.4	500.0	323.5	300.0	—	240.0	78.9	—	—	442.9	359.4	274.3	—	—
		IQR	—	—	158.0	37.6	—	—	—	174.6	162.0	208.6	186.7	220.0	—	93.1	0.0	—	—	14.1	142.9	202.3	—	—
	Saturated fat per 100 g/mL	Median	—	—	9.6	11.9	—	—	—	5.6	4.9	3.0	6.7	6.9	—	6.0	18.4	—	—	1.0	12.2	6.4	—	—
		IQR	—	—	7.8	1.3	—	—	—	0.9	2.2	1.9	6.0	7.9	—	10.6	0.0	—	—	0.1	5.5	6.4	—	—
	Trans fat per 100 g/mL (g)	Median	—	—	0.0	0.0	—	—	—	0.4	0.1	0.0	0.0	0.1	—	0.0	0.0	—	—	0.1	0.2	0.2	—	—
		IQR	—	—	0.0	0.2	—	—	—	1.3	0.2	0.0	0.0	0.3	—	0.4	0.0	—	—	0.0	0.1	0.3	—	—
	Total sugars per 100 g/mL	Median	—	—	35.0	1.0	—	—	—	22.2	38.1	28.0	31.3	28.3	—	24.1	36.8	—	—	9.0	44.4	25.0	—	—
		IQR	—	—	12.2	4.1	—	—	—	20.2	15.9	19.1	8.8	12.0	—	17.3	0.0	—	—	1.4	17.0	13.4	—	—
	Free sugars per 100 g/mL	Median	—	—	35.0	0.6	—	—	—	21.1	33.0	28.0	31.3	26.8	—	24.1	36.8	—	—	3.5	40.5	22.6	—	—
		IQR	—	—	12.2	3.5	—	—	—	25.9	14.6	24.2	8.8	13.7	—	19.3	0.0	—	—	1.5	22.3	16.0	—	—
Bread (A1, A2, A3, A24)	Number of products		0	0	0	72	0	0	0	11	89	0	0	40	0	0	0	0	0	0	0	25	0	0
	Health Star Rating	Mean	—	—	—	3.7	—	—	—	1.8	3.6	—	—	3.0	—	—	—	—	—	—	—	3.1	—	—
		SD	—	—	—	0.5	—	—	—	0.7	0.7	—	—	1.0	—	—	—	—	—	—	—	0.7	—	—
	Calories per 100 g/mL	Median	—	—	—	251.7	—	—	—	310.3	261.9	—	—	285.7	—	—	—	—	—	—	—	279.4	—	—
		IQR	—	—	—	19.4	—	—	—	66.3	39.9	—	—	99.6	—	—	—	—	—	—	—	73.2	—	—
	Sodium per 100 g/mL	Median	—	—	—	437.4	—	—	—	785.7	442.9	—	—	400.0	—	—	—	—	—	—	—	535.2	—	—
		IQR	—	—	—	160.4	—	—	—	156.9	174.6	—	—	190.1	—	—	—	—	—	—	—	148.5	—	—
	Saturated fat per 100 g/mL	Median	—	—	—	0.7	—	—	—	4.4	0.8	—	—	0.9	—	—	—	—	—	—	—	1.0	—	—
		IQR	—	—	—	0.2	—	—	—	4.8	0.5	—	—	2.8	—	—	—	—	—	—	—	1.5	—	—
	Trans fat per 100 g/mL (g)	Median	—	—	—	0.0	—	—	—	0.9	0.0	—	—	0.0	—	—	—	—	—	—	—	0.0	—	—
		IQR	—	—	—	0.0	—	—	—	0.6	0.0	—	—	0.0	—	—	—	—	—	—	—	0.0	—	—
	Total sugars per 100 g/mL	Median	—	—	—	3.1	—	—	—	6.2	3.5	—	—	4.8	—	—	—	—	—	—	—	4.3	—	—
		IQR	—	—	—	3.1	—	—	—	6.1	2.4	—	—	8.3	—	—	—	—	—	—	—	2.7	—	—
	Free sugars per 100 g/mL	Median	—	—	—	1.7	—	—	—	5.5	2.1	—	—	4.0	—	—	—	—	—	—	—	2.9	—	—
		IQR	—	—	—	2.4	—	—	—	6.2	2.2	—	—	6.2	—	—	—	—	—	—	—	2.8	—	—
Crackers, croutons and rice cakes (A11, A12, A13, A16, A21, A25)	Number of products		0	2	16	0	0	0	0	2	8	14	63	81	0	2	0	0	0	18	0	31	0	0
	Health Star Rating	Mean	—	3.3	2.3	—	—	—	—	2.0	2.0	2.9	2.1	2.3	—	3.8	—	—	—	2.1	—	2.2	—	—
		SD	—	1.1	0.5	—	—	—	—	0.0	0.5	1.1	1.0	0.8	—	1.1	—	—	—	0.8	—	0.8	—	—
	Calories per 100 g/mL	Median	—	386.4	450.0	—	—	—	—	478.8	400.0	400.0	450.0	450.0	—	444.4	—	—	—	450.0	—	428.6	—	—
		IQR	—	22.7	0.0	—	—	—	—	2.6	12.5	44.6	71.4	56.3	—	0.0	—	—	—	47.7	—	28.6	—	—
	Sodium per 100 g/mL	Median	—	454.5	800.0	—	—	—	—	427.2	1000.0	725.0	800.0	700.0	—	416.7	—	—	—	762.5	—	800.0	—	—
		IQR	—	45.5	220.8	—	—	—	—	1.3	337.5	200.8	425.0	316.7	—	166.7	—	—	—	688.6	—	622.1	—	—
	Saturated fat per 100 g/mL	Median	—	3.4	2.3	—	—	—	—	9.4	2.3	1.8	2.6	2.0	—	2.2	—	—	—	1.5	—	1.5	—	—
		IQR	—	1.1	0.5	—	—	—	—	0.1	0.6	1.5	2.9	3.5	—	0.0	—	—	—	1.3	—	2.1	—	—

			A. Lassonde	Agropur	Campbell Soup	Canada Bread	Canada Dry Mott's	Coca-Cola	Danone	General Mills	George Weston	Kellogg	Kraft Heinz	Loblaw	Maple Leaf Foods	Mondelēz	Nestlé	Ocean Spray	Parmalat	PepsiCo	Saputo	Sobeys	Sun-Rype	Unilever
	Trans fat per	Median	—	0.0	0.0	—	—	—	—	0.2	0.0	0.0	0.0	0.0	—	0.0	—	—	—	0.0	—	0.0	—	—
	100 g/mL (g)	IQR	—	0.0	0.0	—	—	—	—	0.2	0.0	0.0	0.0	0.0	—	0.0	—	—	—	0.0	—	0.0	—	—
	Total sugars	Median	—	4.5	4.2	—	—	—	—	0.0	0.0	5.0	5.0	4.8	—	0.0	—	—	—	5.0	—	5.0	—	—
	per 100 g/mL	IQR	—	0.0	9.1	—	—	—	—	0.0	8.8	1.6	9.8	7.1	—	0.0	—	—	—	19.5	—	8.7	—	—
	Free sugars	Median	—	3.2	3.8	—	—	—	—	0.0	0.0	4.5	4.5	3.1	—	0.0	—	—	—	4.5	—	4.5	—	—
	per 100 g/mL	IQR	—	0.0	8.6	—	—	—	—	0.0	4.8	1.5	9.3	6.8	—	0.0	—	—	—	22.7	—	8.3	—	—
Grain-based,	Number of products		0	0	0	0	0	0	0	27	0	31	0	20	0	0	0	0	0	31	2	27	2	0
protein and	Health Star	Mean	—	—	—	—	—	—	—	2.6	—	2.7	—	2.2	—	—	—	—	—	2.5	1.8	2.4	3.5	—
energy bars	Rating	SD	—	—	—	—	—	—	—	0.8	—	1.0	—	0.5	—	—	—	—	—	0.9	0.4	0.8	0.0	—
(A18, A19,	Calories per	Median	—	—	—	—	—	—	—	437.5	—	407.4	—	428.6	—	—	—	—	—	428.6	450.1	423.1	366.7	—
A20)	100 g/mL	IQR	—	—	—	—	—	—	—	76.0	—	53.7	—	36.1	—	—	—	—	—	18.4	8.2	78.0	0.0	—
	Sodium per	Median	—	—	—	—	—	—	—	271.4	—	270.3	—	200.0	—	—	—	—	—	242.9	389.5	269.2	58.3	—
	100 g/mL	IQR	—	—	—	—	—	—	—	152.9	—	103.9	—	99.1	—	—	—	—	—	54.9	110.5	128.7	25.0	—
	Saturated fat	Median	—	—	—	—	—	—	—	4.3	—	1.4	—	3.6	—	—	—	—	—	3.8	3.9	2.2	1.7	—
	per 100 g/mL	IQR	—	—	—	—	—	—	—	4.5	—	3.5	—	4.8	—	—	—	—	—	4.3	1.9	3.9	1.7	—
	Trans fat per	Median	—	—	—	—	—	—	—	0.0	—	0.0	—	0.0	—	—	—	—	—	0.0	0.0	0.0	0.0	—
	100 g/mL (g)	IQR	—	—	—	—	—	—	—	0.0	—	0.0	—	0.0	—	—	—	—	—	0.0	0.0	0.0	0.0	—
	Total sugars	Median	—	—	—	—	—	—	—	34.3	—	30.0	—	34.5	—	—	—	—	—	25.7	32.7	28.9	55.0	—
	per 100 g/mL	IQR	—	—	—	—	—	—	—	4.5	—	12.0	—	7.5	—	—	—	—	—	8.9	4.8	8.2	1.7	—
	Free sugars	Median	—	—	—	—	—	—	—	31.7	—	28.0	—	30.0	—	—	—	—	—	22.4	30.5	25.0	51.5	—
	per 100 g/mL	IQR	—	—	—	—	—	—	—	7.1	—	8.5	—	6.4	—	—	—	—	—	7.6	4.5	7.1	1.5	—
**Beverages (B)**	Number of products		1	0	1	0	16	28	0	0	0	0	44	49	0	0	42	0	0	33	1	45	0	10
	Health Star	Mean	1.5	—	2.0	—	1.3	1.7	—	—	—	—	1.7	1.5	—	—	1.7	—	—	1.7	1.0	1.4	—	1.9
	Rating	SD	N/A	—	N/A	—	0.5	0.8	—	—	—	—	0.4	0.6	—	—	0.6	—	—	0.8	N/A	0.5	—	0.3
	Calories per	Median	36.0	—	76.0	—	34.9	21.1	—	—	—	—	2.0	32.0	—	—	23.4	—	—	24.0	44.0	40.0	—	0.0
	100 g/mL	IQR	0.0	—	0.0	—	39.4	31.2	—	—	—	—	39.0	44.0	—	—	44.4	—	—	28.0	0.0	48.0	—	0.0
	Sodium per	Median	2.0	—	42.0	—	18.0	26.1	—	—	—	—	6.0	6.0	—	—	1.6	—	—	31.0	4.0	6.0	—	0.0
	100 g/mL	IQR	0.0	—	0.0	—	11.6	29.9	—	—	—	—	29.4	2.4	—	—	18.2	—	—	29.6	0.0	4.0	—	23.8
	Saturated	Median	0.0	—	0.6	—	0.0	0.0	—	—	—	—	0.0	0.0	—	—	0.0	—	—	0.0	0.0	0.0	—	0.0
	fat per 100	IQR	0.0	—	0.0	—	0.0	0.0	—	—	—	—	0.2	0.0	—	—	0.6	—	—	0.0	0.0	0.0	—	0.0
	Trans fat	Median	0.0	—	0.0	—	0.0	0.0	—	—	—	—	0.0	0.0	—	—	0.0	—	—	0.0	0.0	0.0	—	0.0
	per 100	IQR	0.0	—	0.0	—	0.0	0.0	—	—	—	—	0.0	0.0	—	—	0.0	—	—	0.0	0.0	0.0	—	0.0
	Total sugars	Median	8.8	—	12.8	—	9.0	5.6	—	—	—	—	0.0	6.8	—	—	4.6	—	—	5.9	10.4	10.0	—	0.0
	per 100	IQR	0.0	—	0.0	—	10.1	8.1	—	—	—	—	4.0	11.2	—	—	9.4	—	—	7.2	0.0	12.0	—	0.0
	Free sugars	Median	8.8	—	12.8	—	9.0	5.6	—	—	—	—	0.0	6.8	—	—	4.4	—	—	4.5	10.4	9.6	—	0.0
	per 100	IQR	0.0	—	0.0	—	10.1	8.1	—	—	—	—	3.6	11.2	—	—	9.0	—	—	4.2	0.0	12.0	—	0.0
Carbonated	Number of products		1	0	1	0	16	28	0	0	0	0	33	44	0	0	28	0	0	33	1	43	0	4
and non-	Health Star	Mean	1.5	—	2.0	—	1.3	1.7	—	—	—	—	1.7	1.6	—	—	1.8	—	—	1.7	1.0	1.4	—	1.6
carbonated	Rating	SD	N/A	—	N/A	—	0.5	0.8	—	—	—	—	0.5	0.6	—	—	0.4	—	—	0.8	N/A	0.5	—	0.5
beverages (B1)	Calories per	Median	36.0	—	76.0	—	34.9	21.1	—	—	—	—	2.0	28.2	—	—	1.5	—	—	24.0	44.0	38.1	—	10.6
	100 g/mL	IQR	0.0	—	0.0	—	39.4	31.2	—	—	—	—	38.9	44.0	—	—	28.7	—	—	28.0	0.0	44.5	—	24.3
	Sodium per	Median	2.0	—	42.0	—	18.0	26.1	—	—	—	—	3.0	6.0	—	—	0.0	—	—	31.0	4.0	6.0	—	25.5
	100 g/mL	IQR	0.0	—	0.0	—	11.6	29.9	—	—	—	—	6.0	2.8	—	—	1.7	—	—	29.6	0.0	2.2	—	3.7
	Saturated fat	Median	0.0	—	0.6	—	0.0	0.0	—	—	—	—	0.0	0.0	—	—	0.0	—	—	0.0	0.0	0.0	—	0.0
	per 100 g/mL	IQR	0.0	—	0.0	—	0.0	0.0	—	—	—	—	0.0	0.0	—	—	0.0	—	—	0.0	0.0	0.0	—	0.0
	Trans fat per	Median	0.0	—	0.0	—	0.0	0.0	—	—	—	—	0.0	0.0	—	—	0.0	—	—	0.0	0.0	0.0	—	0.0
	100 g/mL (g)	IQR	0.0	—	0.0	—	0.0	0.0	—	—	—	—	0.0	0.0	—	—	0.0	—	—	0.0	0.0	0.0	—	0.0
	Total sugars	Median	8.8	—	12.8	—	9.0	5.6	—	—	—	—	0.0	6.4	—	—	0.0	—	—	5.9	10.4	9.6	—	2.8
	per 100 g/mL	IQR	0.0	—	0.0	—	10.1	8.1	—	—	—	—	10.0	11.2	—	—	7.2	—	—	7.2	0.0	12.1	—	6.5
	Free sugars	Median	8.8	—	12.8	—	9.0	5.6	—	—	—	—	0.0	6.4	—	—	0.0	—	—	4.5	10.4	9.6	—	2.8
	per 100 g/mL	IQR	0.0	—	0.0	—	10.1	8.1	—	—	—	—	10.0	11.2	—	—	7.2	—	—	4.2	0.0	12.1	—	6.5
Coffee, tea and	Number of products		0	0	0	0	0	0	0	0	0	0	11	5	0	0	14	0	0	0	0	2	0	6
hot chocolate	Health Star	Mean	—	—	—	—	—	—	—	—	—	—	1.6	1.1	—	—	1.4	—	—	—	—	1.0	—	2.0
(B3-B5)	Rating	SD	—	—	—	—	—	—	—	—	—	—	0.3	0.7	—	—	0.7	—	—	—	—	0.0	—	0.0
	Calories per	Median	—	—	—	—	—	—	—	—	—	—	27.1	57.1	—	—	41.7	—	—	—	—	68.6	—	0.0
	100 g/mL	IQR	—	—	—	—	—	—	—	—	—	—	20.3	36.3	—	—	40.6	—	—	—	—	0.0	—	0.0
	Sodium per	Median	—	—	—	—	—	—	—	—	—	—	46.9	60.0	—	—	33.3	—	—	—	—	60.0	—	0.0
	100 g/mL	IQR	—	—	—	—	—	—	—	—	—	—	11.3	34.1	—	—	37.7	—	—	—	—	25.7	—	0.0
	Saturated fat	Median	—	—	—	—	—	—	—	—	—	—	1.1	0.9	—	—	1.0	—	—	—	—	0.9	—	0.0
	per 100 g/mL	IQR	—	—	—	—	—	—	—	—	—	—	0.4	0.0	—	—	1.2	—	—	—	—	0.0	—	0.0
	Trans fat per	Median	—	—	—	—	—	—	—	—	—	—	0.1	0.0	—	—	0.0	—	—	—	—	0.0	—	0.0
	100 g/mL (g)	IQR	—	—	—	—	—	—	—	—	—	—	0.0	0.0	—	—	0.0	—	—	—	—	0.0	—	0.0
	Total sugars	Median	—	—	—	—	—	—	—	—	—	—	1.1	9.7	—	—	6.0	—	—	—	—	11.1	—	0.0
	per 100 g/mL	IQR	—	—	—	—	—	—	—	—	—	—	2.6	7.7	—	—	6.6	—	—	—	—	0.3	—	0.0
	Free sugars	Median	—	—	—	—	—	—	—	—	—	—	1.1	8.1	—	—	6.0	—	—	—	—	9.5	—	0.0
	per 100 g/mL	IQR	—	—	—	—	—	—	—	—	—	—	2.6	6.0	—	—	6.2	—	—	—	—	0.3	—	0.0
**Cereals and**	Number of products		0	0	1	13	0	0	0	21	1	45	5	141	0	0	1	0	0	33	0	54	0	9
**other grains**	Health Star	Mean	—	—	2.0	3.6	—	—	—	2.8	4.0	3.5	2.0	4.0	—	—	1.5	—	—	3.3	—	3.5	—	2.1
**(C)**	Rating	SD	—	—	N/A	0.2	—	—	—	1.0	N/A	1.1	0.7	0.7	—	—	N/A	—	—	1.2	—	0.9	—	0.2
	Calories per	Median	—	—	400.0	283.3	—	—	—	392.9	460.0	375.0	366.7	361.7	—	—	400.0	—	—	394.7	—	364.7	—	393.9
	100 g/mL	IQR	—	—	0.0	30.0	—	—	—	34.5	0.0	39.1	0.0	43.9	—	—	0.0	—	—	11.1	—	17.8	—	26.3
	Sodium per	Median	—	—	1166.7	320.0	—	—	—	586.2	70.0	388.9	1400.0	11.1	—	—	1480.0	—	—	441.9	—	21.7	—	1000.0
	100 g/mL	IQR	—	—	0.0	100.0	—	—	—	271.8	0.0	333.3	500.0	280.0	—	—	0.0	—	—	407.1	—	505.9	—	89.1

			A. Lassonde	Agropur	Campbell Soup	Canada Bread	Canada Dry Mott's	Coca-Cola	Danone	General Mills	George Weston	Kellogg	Kraft Heinz	Loblaw	Maple Leaf Foods	Mondelēz	Nestlé	Ocean Spray	Parmalat	PepsiCo	Saputo	Sobeys	Sun-Rype	Unilever
	**Saturated fat per 100**	**Median**	—	—	**1.0**	**2.0**	—	—	—	**0.7**	**2.0**	**0.0**	**0.7**	**0.4**	—	—	**6.0**	—	—	**1.0**	—	**0.2**	—	**0.0**
		**IQR**	—	—	**0.0**	**1.0**	—	—	—	**1.0**	**0.0**	**0.7**	**0.7**	**0.9**	—	—	**0.0**	—	—	**0.9**	—	**0.7**	—	**0.8**
	**Trans fat per 100**	**Median**	—	—	**0.7**	**0.1**	—	—	—	**0.0**	**0.0**	**0.0**	**0.7**	**0.0**	—	—	**0.0**	—	—	**0.0**	—	**0.0**	—	**0.0**
		**IQR**	—	—	**0.0**	**0.1**	—	—	—	**0.0**	**0.0**	**0.0**	**0.7**	**0.0**	—	—	**0.0**	—	—	**0.0**	—	**0.0**	—	**0.0**
	**Total sugars per 100**	**Median**	—	—	**3.3**	**3.0**	—	—	—	**29.4**	**22.0**	**21.8**	**10.0**	**2.4**	—	—	**2.0**	—	—	**20.0**	—	**3.5**	—	**2.6**
		**IQR**	—	—	**0.0**	**3.0**	—	—	—	**11.0**	**0.0**	**7.5**	**3.3**	**6.7**	—	—	**0.0**	—	—	**23.9**	—	**5.8**	—	**0.4**
	**Free sugars per 100**	**Median**	—	—	**0.0**	**0.0**	—	—	—	**25.3**	**17.4**	**18.5**	**6.0**	**0.0**	—	—	**0.0**	—	—	**17.6**	—	**0.0**	—	**0.0**
		**IQR**	—	—	**0.0**	**0.0**	—	—	—	**11.6**	**0.0**	**10.1**	**3.7**	**0.8**	—	—	**0.0**	—	—	**24.7**	—	**2.3**	—	**0.0**
Breakfast cereals, hot and ready-to-eat (C1-C4)	Number of products		0	0	0	0	0	0	0	20	1	45	0	41	0	0	0	0	0	30	0	16	0	0
	Health Star Rating	Mean	—	—	—	—	—	—	—	2.8	4.0	3.5	—	3.8	—	—	—	—	—	3.3	—	3.1	—	—
		SD	—	—	—	—	—	—	—	1.0	N/A	1.1	—	0.8	—	—	—	—	—	1.2	—	1.0	—	—
	Calories per 100 g/mL	Median	—	—	—	—	—	—	—	392.9	460.0	375.0	—	400.0	—	—	—	—	—	397.7	—	381.8	—	—
		IQR	—	—	—	—	—	—	—	32.2	0.0	39.1	—	5.3	—	—	—	—	—	10.1	—	28.2	—	—
	Sodium per 100 g/mL	Median	—	—	—	—	—	—	—	569.0	70.0	388.9	—	200.0	—	—	—	—	—	441.9	—	490.1	—	—
		IQR	—	—	—	—	—	—	—	269.7	0.0	333.3	—	345.5	—	—	—	—	—	386.1	—	319.0	—	—
	Saturated fat per 100 g/mL	Median	—	—	—	—	—	—	—	0.8	2.0	0.0	—	1.1	—	—	—	—	—	1.2	—	0.0	—	—
		IQR	—	—	—	—	—	—	—	1.0	0.0	0.7	—	0.9	—	—	—	—	—	1.9	—	0.8	—	—
	Trans fat per 100 g/mL (g)	Median	—	—	—	—	—	—	—	0.0	0.0	0.0	—	0.0	—	—	—	—	—	0.0	—	0.0	—	—
		IQR	—	—	—	—	—	—	—	0.0	0.0	0.0	—	0.0	—	—	—	—	—	0.0	—	0.0	—	—
	Total sugars per 100 g/mL	Median	—	—	—	—	—	—	—	29.5	22.0	21.8	—	20.0	—	—	—	—	—	22.2	—	19.1	—	—
		IQR	—	—	—	—	—	—	—	9.7	0.0	7.5	—	17.0	—	—	—	—	—	14.0	—	21.7	—	—
	Free sugars per 100 g/mL	Median	—	—	—	—	—	—	—	25.3	17.4	18.5	—	15.3	—	—	—	—	—	19.7	—	17.4	—	—
		IQR	—	—	—	—	—	—	—	10.2	0.0	10.1	—	19.5	—	—	—	—	—	14.8	—	24.4	—	—
Grains (e.g., rice, barley), including flavoured (C7)	Number of products		0	0	0	0	0	0	0	1	0	0	0	30	0	0	1	0	0	2	0	13	0	8
	Health Star Rating	Mean	—	—	—	—	—	—	—	2.0	—	—	—	4.0	—	—	1.5	—	—	1.8	—	3.3	—	2.0
		SD	—	—	—	—	—	—	—	N/A	—	—	—	0.6	—	—	N/A	—	—	0.4	—	0.8	—	0.0
	Calories per 100 g/mL	Median	—	—	—	—	—	—	—	351.9	—	—	—	353.0	—	—	400.0	—	—	342.9	—	359.0	—	394.3
		IQR	—	—	—	—	—	—	—	0.0	—	—	—	25.6	—	—	0.0	—	—	0.0	—	10.3	—	20.7
	Sodium per 100 g/mL	Median	—	—	—	—	—	—	—	870.4	—	—	—	0.0	—	—	1480.0	—	—	1757.1	—	0.0	—	1012.8
		IQR	—	—	—	—	—	—	—	0.0	—	—	—	131.1	—	—	0.0	—	—	228.6	—	815.8	—	86.6
	Saturated fat per 100 g/mL	Median	—	—	—	—	—	—	—	0.4	—	—	—	0.0	—	—	6.0	—	—	0.1	—	0.0	—	0.3
		IQR	—	—	—	—	—	—	—	0.0	—	—	—	0.4	—	—	0.0	—	—	0.0	—	0.6	—	0.9
	Trans fat per 100 g/mL (g)	Median	—	—	—	—	—	—	—	0.0	—	—	—	0.0	—	—	0.0	—	—	0.0	—	0.0	—	0.0
		IQR	—	—	—	—	—	—	—	0.0	—	—	—	0.0	—	—	0.0	—	—	0.0	—	0.0	—	0.0
	Total sugars per 100 g/mL	Median	—	—	—	—	—	—	—	3.7	—	—	—	0.0	—	—	2.0	—	—	4.3	—	0.0	—	2.6
		IQR	—	—	—	—	—	—	—	0.0	—	—	—	1.1	—	—	0.0	—	—	1.4	—	2.9	—	0.3
	Free sugars per 100 g/mL	Median	—	—	—	—	—	—	—	0.0	—	—	—	0.0	—	—	0.0	—	—	0.7	—	0.0	—	0.0
		IQR	—	—	—	—	—	—	—	0.0	—	—	—	0.0	—	—	0.0	—	—	0.7	—	0.0	—	0.0
Other (C5, C6, C10, C11)	Number of products		0	0	1	0	0	0	0	0	0	0	5	10	0	0	0	0	0	1	0	2	0	1
	Health Star Rating	Mean	—	—	2.0	—	—	—	—	—	—	—	2.0	3.7	—	—	—	—	—	5.0	—	2.0	—	2.5
		SD	—	—	N/A	—	—	—	—	—	—	—	0.7	1.6	—	—	—	—	—	N/A	—	0.7	—	N/A
	Calories per 100 g/mL	Median	—	—	400.0	—	—	—	—	—	—	—	366.7	358.3	—	—	—	—	—	333.3	—	366.7	—	357.1
		IQR	—	—	0.0	—	—	—	—	—	—	—	0.0	54.2	—	—	—	—	—	0.0	—	0.0	—	0.0
	Sodium per 100 g/mL	Median	—	—	1166.7	—	—	—	—	—	—	—	1400.0	0.0	—	—	—	—	—	16.7	—	1466.7	—	28.6
		IQR	—	—	0.0	—	—	—	—	—	—	—	500.0	25.0	—	—	—	—	—	0.0	—	100.0	—	0.0
	Saturated fat per 100 g/mL	Median	—	—	1.0	—	—	—	—	—	—	—	0.7	0.0	—	—	—	—	—	0.7	—	0.0	—	0.0
		IQR	—	—	0.0	—	—	—	—	—	—	—	0.7	1.3	—	—	—	—	—	0.0	—	0.0	—	0.0
	Trans fat per 100 g/mL (g)	Median	—	—	0.7	—	—	—	—	—	—	—	0.7	0.0	—	—	—	—	—	0.0	—	0.0	—	0.0
		IQR	—	—	0.0	—	—	—	—	—	—	—	0.7	0.0	—	—	—	—	—	0.0	—	0.0	—	0.0
	Total sugars per 100 g/mL	Median	—	—	3.3	—	—	—	—	—	—	—	10.0	0.0	—	—	—	—	—	3.3	—	6.7	—	28.6
		IQR	—	—	0.0	—	—	—	—	—	—	—	3.3	3.8	—	—	—	—	—	0.0	—	0.0	—	0.0
	Free sugars per 100 g/mL	Median	—	—	0.0	—	—	—	—	—	—	—	6.0	0.0	—	—	—	—	—	0.0	—	2.3	—	27.1
		IQR	—	—	0.0	—	—	—	—	—	—	—	3.7	0.0	—	—	—	—	—	0.0	—	0.0	—	0.0
Pastas, including filled pastas, without sauce (C8)	Number of products		0	0	0	13	0	0	0	0	0	0	0	60	0	0	0	0	0	0	0	23	0	0
	Health Star Rating	Mean	—	—	—	3.6	—	—	—	—	—	—	—	4.2	—	—	—	—	—	—	—	4.1	—	—
		SD	—	—	—	0.2	—	—	—	—	—	—	—	0.5	—	—	—	—	—	—	—	0.5	—	—
	Calories per 100 g/mL	Median	—	—	—	283.3	—	—	—	—	—	—	—	360.0	—	—	—	—	—	—	—	364.7	—	—
		IQR	—	—	—	30.0	—	—	—	—	—	—	—	15.0	—	—	—	—	—	—	—	69.7	—	—
	Sodium per 100 g/mL	Median	—	—	—	320.0	—	—	—	—	—	—	—	5.9	—	—	—	—	—	—	—	0.0	—	—
		IQR	—	—	—	100.0	—	—	—	—	—	—	—	21.6	—	—	—	—	—	—	—	25.6	—	—
	Saturated fat per 100 g/mL	Median	—	—	—	2.0	—	—	—	—	—	—	—	0.4	—	—	—	—	—	—	—	0.2	—	—
		IQR	—	—	—	1.0	—	—	—	—	—	—	—	0.2	—	—	—	—	—	—	—	0.2	—	—
	Trans fat per 100 g/mL (g)	Median	—	—	—	0.1	—	—	—	—	—	—	—	0.0	—	—	—	—	—	—	—	0.0	—	—
		IQR	—	—	—	0.1	—	—	—	—	—	—	—	0.0	—	—	—	—	—	—	—	0.0	—	—
	Total sugars per 100 g/mL	Median	—	—	—	3.0	—	—	—	—	—	—	—	2.4	—	—	—	—	—	—	—	3.5	—	—
		IQR	—	—	—	3.0	—	—	—	—	—	—	—	0.9	—	—	—	—	—	—	—	1.8	—	—
	Free sugars per 100 g/mL	Median	—	—	—	0.0	—	—	—	—	—	—	—	0.0	—	—	—	—	—	—	—	0.0	—	—
		IQR	—	—	—	0.0	—	—	—	—	—	—	—	0.0	—	—	—	—	—	—	—	0.0	—	—
**Dairy products and substitutes (D)**	**Number of products**		**0**	**97**	**1**	**0**	**0**	**0**	**63**	**77**	**0**	**3**	**93**	**151**	**2**	**0**	**18**	**0**	**62**	**0**	**40**	**78**	**0**	**0**
	**Health Star Rating**	**Mean**	—	**3.6**	**4.5**	—	—	—	**3.9**	**3.8**	—	**4.5**	**2.3**	**3.1**	**2.8**	—	**2.0**	—	**3.5**	—	**3.5**	**2.8**	—	—
		**SD**	—	**1.4**	**N/A**	—	—	—	**0.9**	**1.4**	—	**0.0**	**1.4**	**1.6**	**0.4**	—	**0.8**	—	**1.2**	—	**1.3**	**1.5**	—	—
	**Calories per 100 g/mL**	**Median**	—	**100.0**	**86.7**	—	—	—	**90.0**	**80.0**	—	**64.2**	**285.7**	**285.7**	**87.5**	—	**150.0**	—	**104.0**	—	**96.9**	**333.3**	—	—
		**IQR**	—	**233.3**	**0.0**	—	—	—	**46.4**	**42.9**	—	**0.0**	**128.6**	**281.7**	**12.5**	—	**148.6**	—	**201.0**	—	**174.5**	**149.1**	—	—

			A. Lassonde	Agropur	Campbell Soup	Canada Bread	Canada Dry Mott's	Coca-Cola	Danone	General Mills	George Weston	Kellogg	Kraft Heinz	Loblaw	Maple Leaf Foods	Mondelēz	Nestlé	Ocean Spray	Parmalat	PepsiCo	Saputo	Sobeys	Sun-Rype	Unilever
	**Sodium per 100 g/mL**	**Median**	—	**66.7**	**84.4**	—	—	—	**45.0**	**42.9**	—	**74.3**	**700.0**	**535.7**	**37.5**	—	**116.7**	—	**55.1**	—	**94.6**	**607.7**	—	—
		**IQR**	—	**421.7**	**0.0**	—	—	—	**10.7**	**15.0**	—	**1.7**	**544.3**	**638.6**	**4.2**	—	**165.1**	—	**616.7**	—	**533.0**	**287.5**	—	—
	**Saturated fat per 100**	**Median**	—	**1.7**	**0.8**	—	—	—	**1.0**	**1.0**	—	**0.2**	**13.3**	**11.9**	**7.5**	—	**2.7**	—	**1.5**	—	**1.2**	**15.0**	—	—
		**IQR**	—	**9.2**	**0.0**	—	—	—	**1.4**	**1.7**	—	**0.0**	**5.8**	**15.3**	**0.8**	—	**8.4**	—	**10.6**	—	**9.8**	**8.4**	—	—
	**Trans fat per 100**	**Median**	—	**0.1**	**0.1**	—	—	—	**0.0**	**0.0**	—	**0.0**	**0.7**	**0.7**	**0.2**	—	**0.0**	—	**0.0**	—	**0.0**	**0.7**	—	—
		**IQR**	—	**0.3**	**0.0**	—	—	—	**0.0**	**0.0**	—	**0.0**	**0.3**	**1.0**	**0.0**	—	**0.0**	—	**0.5**	—	**0.7**	**0.6**	—	—
	**Total sugars per 100**	**Median**	—	**4.4**	**10.9**	—	—	—	**10.8**	**10.0**	—	**6.4**	**4.4**	**0.0**	**4.2**	—	**13.3**	—	**6.6**	—	**4.8**	**0.0**	—	—
		**IQR**	—	**11.0**	**0.0**	—	—	—	**7.0**	**8.0**	—	**0.0**	**6.7**	**6.7**	**0.8**	—	**23.3**	—	**12.6**	—	**10.8**	**4.4**	—	—
	**Free sugars per 100**	**Median**	—	**0.0**	**7.8**	—	—	—	**7.2**	**7.6**	—	**3.2**	**0.0**	**0.0**	**4.2**	—	**10.4**	—	**4.6**	—	**0.0**	**0.0**	—	—
		**IQR**	—	**8.3**	**0.0**	—	—	—	**7.9**	**8.8**	—	**0.0**	**0.0**	**0.9**	**0.8**	—	**27.8**	—	**9.4**	—	**6.3**	**0.0**	—	—
Cheese (D1-D5)	Number of products		0	36	0	0	0	0	0	5	0	0	87	101	0	0	0	0	23	0	17	67	0	0
	Health Star Rating	Mean	—	3.4	—	—	—	—	—	3.2	—	—	2.3	2.8	—	—	—	—	3.2	—	3.7	3.0	—	—
		SD	—	1.7	—	—	—	—	—	1.7	—	—	1.5	1.6	—	—	—	—	1.4	—	1.6	1.5	—	—
	Calories per 100 g/mL	Median	—	333.3	—	—	—	—	—	145.5	—	—	300.0	333.3	—	—	—	—	333.3	—	266.7	333.3	—	—
		IQR	—	141.7	—	—	—	—	—	109.1	—	—	100.0	114.3	—	—	—	—	147.6	—	215.9	126.4	—	—
	Sodium per 100 g/mL	Median	—	666.7	—	—	—	—	—	309.1	—	—	700.0	666.7	—	—	—	—	666.7	—	733.3	666.7	—	—
		IQR	—	333.3	—	—	—	—	—	348.3	—	—	602.4	264.3	—	—	—	—	470.0	—	512.7	250.0	—	—
	Saturated fat per 100 g/mL	Median	—	15.0	—	—	—	—	—	6.4	—	—	13.3	16.7	—	—	—	—	16.7	—	11.7	16.7	—	—
		IQR	—	13.3	—	—	—	—	—	9.6	—	—	6.0	8.3	—	—	—	—	11.0	—	14.2	7.2	—	—
	Trans fat per 100 g/mL (g)	Median	—	0.3	—	—	—	—	—	0.0	—	—	0.7	1.0	—	—	—	—	1.0	—	0.7	0.7	—	—
		IQR	—	0.4	—	—	—	—	—	0.4	—	—	0.3	0.3	—	—	—	—	0.6	—	0.7	0.3	—	—
	Total sugars per 100 g/mL	Median	—	0.0	—	—	—	—	—	3.0	—	—	0.0	0.0	—	—	—	—	0.0	—	0.0	0.0	—	—
		IQR	—	0.1	—	—	—	—	—	1.5	—	—	6.7	0.0	—	—	—	—	2.4	—	4.4	3.3	—	—
	Free sugars per 100 g/mL	Median	—	0.0	—	—	—	—	—	0.0	—	—	0.0	0.0	—	—	—	—	0.0	—	0.0	0.0	—	—
		IQR	—	0.0	—	—	—	—	—	0.0	—	—	0.0	0.0	—	—	—	—	1.9	—	0.0	0.0	—	—
Cream and cream substitutes (D6, D7, D8, D14)	Number of products		0	10	0	0	0	0	0	4	0	0	6	8	2	0	11	0	1	0	6	6	0	0
	Health Star Rating	Mean	—	2.4	—	—	—	—	—	1.3	—	—	2.3	2.1	2.8	—	1.8	—	3.5	—	2.3	2.0	—	—
		SD	—	0.5	—	—	—	—	—	1.2	—	—	1.1	1.3	0.4	—	0.8	—	N/A	—	0.8	1.2	—	—
	Calories per 100 g/mL	Median	—	166.7	—	—	—	—	—	233.3	—	—	127.8	121.4	87.5	—	233.3	—	66.7	—	200.0	121.4	—	—
		IQR	—	91.7	—	—	—	—	—	179.2	—	—	133.5	113.2	12.5	—	66.7	—	0.0	—	75.0	69.0	—	—
	Sodium per 100 g/mL	Median	—	66.7	—	—	—	—	—	41.7	—	—	5.6	89.3	37.5	—	200.0	—	100.0	—	66.7	64.3	—	—
		IQR	—	0.0	—	—	—	—	—	16.7	—	—	28.6	39.6	4.2	—	83.3	—	0.0	—	76.7	97.0	—	—
	Saturated fat per 100 g/mL	Median	—	5.0	—	—	—	—	—	12.5	—	—	5.0	4.6	7.5	—	10.0	—	1.3	—	6.7	4.7	—	—
		IQR	—	5.8	—	—	—	—	—	12.1	—	—	10.1	7.9	0.8	—	7.3	—	0.0	—	5.0	4.2	—	—
	Trans fat per 100 g/mL (g)	Median	—	0.3	—	—	—	—	—	0.0	—	—	0.0	0.0	0.2	—	0.0	—	0.0	—	0.3	0.0	—	—
		IQR	—	0.7	—	—	—	—	—	0.3	—	—	0.0	0.0	0.0	—	0.0	—	0.0	—	0.7	0.0	—	—
	Total sugars per 100 g/mL	Median	—	6.7	—	—	—	—	—	3.3	—	—	8.0	5.8	4.2	—	33.3	—	6.7	—	6.7	4.4	—	—
		IQR	—	10.0	—	—	—	—	—	0.8	—	—	15.5	3.2	0.8	—	20.0	—	0.0	—	20.0	2.5	—	—
	Free sugars per 100 g/mL	Median	—	0.0	—	—	—	—	—	0.0	—	—	8.0	2.2	4.2	—	33.3	—	3.3	—	3.3	2.0	—	—
		IQR	—	13.0	—	—	—	—	—	0.0	—	—	15.5	5.5	0.8	—	20.0	—	0.0	—	22.5	4.0	—	—
Milk and milk alternatives (D10, D11)	Number of products		0	18	0	0	0	0	0	3	0	0	0	15	0	0	4	0	3	0	12	5	0	0
	Health Star Rating	Mean	—	4.6	—	—	—	—	—	4.2	—	—	—	3.1	—	—	1.9	—	4.7	—	4.3	2.4	—	—
		SD	—	0.5	—	—	—	—	—	0.3	—	—	—	1.4	—	—	0.8	—	0.3	—	0.5	1.8	—	—
	Calories per 100 g/mL	Median	—	50.0	—	—	—	—	—	48.0	—	—	—	52.0	—	—	80.0	—	44.0	—	56.0	80.0	—	—
		IQR	—	23.0	—	—	—	—	—	10.0	—	—	—	58.7	—	—	13.8	—	8.0	—	14.6	66.7	—	—
	Sodium per 100 g/mL	Median	—	46.0	—	—	—	—	—	40.0	—	—	—	48.0	—	—	57.3	—	48.0	—	59.0	166.7	—	—
		IQR	—	26.0	—	—	—	—	—	0.0	—	—	—	50.5	—	—	15.0	—	1.0	—	28.2	102.0	—	—
	Saturated fat per 100 g/mL	Median	—	0.6	—	—	—	—	—	1.2	—	—	—	0.6	—	—	1.7	—	0.6	—	0.6	1.2	—	—
		IQR	—	0.5	—	—	—	—	—	0.7	—	—	—	0.9	—	—	0.6	—	0.6	—	0.2	3.1	—	—
	Trans fat per 100 g/mL (g)	Median	—	0.0	—	—	—	—	—	0.0	—	—	—	0.0	—	—	0.0	—	0.0	—	0.0	0.0	—	—
		IQR	—	0.1	—	—	—	—	—	0.0	—	—	—	0.0	—	—	0.0	—	0.0	—	0.0	0.0	—	—
	Total sugars per 100 g/mL	Median	—	4.4	—	—	—	—	—	4.4	—	—	—	5.2	—	—	10.6	—	4.8	—	6.4	6.7	—	—
		IQR	—	3.4	—	—	—	—	—	0.0	—	—	—	3.3	—	—	3.0	—	0.2	—	6.0	4.5	—	—
	Free sugars per 100 g/mL	Median	—	0.0	—	—	—	—	—	0.0	—	—	—	0.0	—	—	6.1	—	0.0	—	1.5	3.8	—	—
		IQR	—	3.2	—	—	—	—	—	0.0	—	—	—	3.4	—	—	3.6	—	0.0	—	6.3	6.7	—	—
Yogurt, yogurt drinks and shakes (D12, D13, D15)	Number of products		0	33	1	0	0	0	63	65	0	3	0	27	0	0	3	0	35	0	5	0	0	0
	Health Star Rating	Mean	—	3.7	4.5	—	—	—	3.9	4.0	—	4.5	—	4.4	—	—	3.0	—	3.7	—	2.6	—	—	—
		SD	—	1.2	N/A	—	—	—	0.9	1.2	—	0.0	—	0.8	—	—	0.5	—	1.0	—	0.2	—	—	—
	Calories per 100 g/mL	Median	—	83.3	86.7	—	—	—	90.0	80.0	—	64.2	—	91.4	—	—	84.7	—	90.0	—	98.5	—	—	—
		IQR	—	27.1	0.0	—	—	—	46.4	32.9	—	0.0	—	32.4	—	—	4.2	—	22.0	—	3.1	—	—	—
	Sodium per 100 g/mL	Median	—	48.6	84.4	—	—	—	45.0	44.0	—	74.3	—	50.0	—	—	63.6	—	50.0	—	76.9	—	—	—
		IQR	—	5.0	0.0	—	—	—	10.7	10.0	—	1.7	—	5.2	—	—	6.4	—	8.5	—	9.2	—	—	—
	Saturated fat per 100 g/mL	Median	—	1.0	0.8	—	—	—	1.0	1.0	—	0.2	—	0.0	—	—	1.3	—	1.0	—	1.1	—	—	—
		IQR	—	1.4	0.0	—	—	—	1.4	1.7	—	0.0	—	1.1	—	—	0.0	—	1.7	—	0.0	—	—	—
	Trans fat per 100 g/mL (g)	Median	—	0.0	0.1	—	—	—	0.0	0.0	—	0.0	—	0.0	—	—	0.0	—	0.0	—	0.0	—	—	—
		IQR	—	0.1	0.0	—	—	—	0.0	0.0	—	0.0	—	0.0	—	—	0.0	—	0.1	—	0.0	—	—	—
	Total sugars per 100 g/mL	Median	—	11.7	10.9	—	—	—	10.8	11.0	—	6.4	—	9.6	—	—	13.1	—	12.0	—	14.2	—	—	—
		IQR	—	2.2	0.0	—	—	—	7.0	8.0	—	0.0	—	7.8	—	—	0.8	—	6.3	—	0.3	—	—	—
	Free sugars per 100 g/mL	Median	—	8.5	7.8	—	—	—	7.2	8.6	—	3.2	—	6.5	—	—	10.1	—	9.0	—	11.2	—	—	—
		IQR	—	2.9	0.0	—	—	—	7.9	9.0	—	0.0	—	7.5	—	—	1.6	—	7.4	—	0.3	—	—	—
**Desserts (E)**	**Number of products**		**0**	**0**	**0**	**0**	**1**	**0**	**0**	**0**	**0**	**0**	**44**	**98**	**0**	**0**	**98**	**0**	**0**	**0**	**0**	**84**	**0**	**61**
	**Health Star Rating**	**Mean**	—	—	—	—	**3.5**	—	—	—	—	—	—	**3.4**	**2.8**	—	—	**2.5**	—	—	—	—	**2.8**	—	**2.5**
		**SD**	—	—	—	—	**N/A**	—	—	—	—	—	—	**0.2**	**0.5**	—	—	**0.7**	—	—	—	—	**0.6**	—	**0.6**

			A. Lassonde	Agropur	Campbell Soup	Canada Bread	Canada Dry Mott's	Coca-Cola	Danone	General Mills	George Weston	Kellogg	Kraft Heinz	Loblaw	Maple Leaf Foods	Mondelēz	Nestlé	Ocean Spray	Parmalat	PepsiCo	Saputo	Sobeys	Sun-Rype	Unilever
	Calories per	Median	—	—	—	—	75.5	—	—	—	—	—	54.8	112.3	—	—	136.0	—	—	—	—	111.1	—	128.0
	100 g/mL	IQR	—	—	—	—	0.0	—	—	—	—	—	38.9	57.9	—	—	116.2	—	—	—	—	56.0	—	95.8
	Sodium per	Median	—	—	—	—	18.9	—	—	—	—	—	87.9	47.3	—	—	52.0	—	—	—	—	50.0	—	44.0
	100 g/mL	IQR	—	—	—	—	0.0	—	—	—	—	—	120.2	46.3	—	—	31.5	—	—	—	—	28.5	—	26.5
	Saturated	Median	—	—	—	—	0.0	—	—	—	—	—	0.0	1.7	—	—	3.8	—	—	—	—	1.9	—	3.6
	fat per 100	IQR	—	—	—	—	0.0	—	—	—	—	—	0.7	4.2	—	—	6.4	—	—	—	—	3.8	—	6.0
	Trans fat	Median	—	—	—	—	0.0	—	—	—	—	—	0.0	0.1	—	—	0.2	—	—	—	—	0.1	—	0.1
	per 100	IQR	—	—	—	—	0.0	—	—	—	—	—	0.0	0.2	—	—	0.2	—	—	—	—	0.2	—	0.2
	Total sugars	Median	—	—	—	—	15.1	—	—	—	—	—	13.0	13.7	—	—	15.3	—	—	—	—	13.6	—	16.0
	per 100	IQR	—	—	—	—	0.0	—	—	—	—	—	14.5	4.8	—	—	5.7	—	—	—	—	4.8	—	8.2
	Free sugars	Median	—	—	—	—	15.1	—	—	—	—	—	11.7	11.6	—	—	12.1	—	—	—	—	11.4	—	13.0
	per 100	IQR	—	—	—	—	0.0	—	—	—	—	—	13.0	5.4	—	—	7.2	—	—	—	—	6.2	—	10.0
Dessert	Number of products		0	0	0	0	0	0	0	10	0	0	4	14	0	0	0	0	0	0	0	8	0	0
toppings and	Health Star	Mean	—	—	—	—	—	—	—	1.1	—	—	2.1	2.1	—	—	—	—	—	—	—	2.1	—	—
fillings (F)	Rating	SD	—	—	—	—	—	—	—	0.4	—	—	0.5	0.7	—	—	—	—	—	—	—	1.0	—	—
	Calories per	Median	—	—	—	—	—	—	—	405.3	—	—	320.5	308.1	—	—	—	—	—	—	—	284.8	—	—
	100 g/mL	IQR	—	—	—	—	—	—	—	54.0	—	—	27.6	223.5	—	—	—	—	—	—	—	285.2	—	—
	Sodium per	Median	—	—	—	—	—	—	—	199.8	—	—	91.7	176.9	—	—	—	—	—	—	—	170.2	—	—
	100 g/mL	IQR	—	—	—	—	—	—	—	54.5	—	—	22.4	171.5	—	—	—	—	—	—	—	178.1	—	—
	Saturated	Median	—	—	—	—	—	—	—	5.4	—	—	1.5	1.4	—	—	—	—	—	—	—	0.0	—	—
	fat per 100	IQR	—	—	—	—	—	—	—	3.2	—	—	0.7	4.3	—	—	—	—	—	—	—	4.3	—	—
	Trans fat	Median	—	—	—	—	—	—	—	6.1	—	—	0.0	0.0	—	—	—	—	—	—	—	0.0	—	—
	per 100	IQR	—	—	—	—	—	—	—	2.0	—	—	0.1	0.0	—	—	—	—	—	—	—	0.0	—	—
	Total sugars	Median	—	—	—	—	—	—	—	58.0	—	—	40.0	41.9	—	—	—	—	—	—	—	44.8	—	—
	per 100	IQR	—	—	—	—	—	—	—	5.3	—	—	15.6	20.3	—	—	—	—	—	—	—	31.1	—	—
	Free sugars	Median	—	—	—	—	—	—	—	58.0	—	—	40.0	41.9	—	—	—	—	—	—	—	44.8	—	—
	per 100	IQR	—	—	—	—	—	—	—	5.3	—	—	15.6	20.3	—	—	—	—	—	—	—	31.1	—	—
Eggs and egg	Number of products		0	0	0	0	0	0	0	0	0	0	0	7	0	0	0	0	0	0	0	6	0	0
substitutes	Health Star	Mean	—	—	—	—	—	—	—	—	—	—	—	3.9	—	—	—	—	—	—	—	3.9	—	—
(G)	Rating	SD	—	—	—	—	—	—	—	—	—	—	—	0.2	—	—	—	—	—	—	—	0.2	—	—
	Calories per	Median	—	—	—	—	—	—	—	—	—	—	—	137.9	—	—	—	—	—	—	—	132.1	—	—
	100 g/mL	IQR	—	—	—	—	—	—	—	—	—	—	—	5.9	—	—	—	—	—	—	—	4.4	—	—
	Sodium per	Median	—	—	—	—	—	—	—	—	—	—	—	129.3	—	—	—	—	—	—	—	126.0	—	—
	100 g/mL	IQR	—	—	—	—	—	—	—	—	—	—	—	6.7	—	—	—	—	—	—	—	7.5	—	—
	Saturated	Median	—	—	—	—	—	—	—	—	—	—	—	3.3	—	—	—	—	—	—	—	2.8	—	—
	fat per 100	IQR	—	—	—	—	—	—	—	—	—	—	—	0.6	—	—	—	—	—	—	—	0.3	—	—
	Trans fat	Median	—	—	—	—	—	—	—	—	—	—	—	0.0	—	—	—	—	—	—	—	0.0	—	—
	per 100	IQR	—	—	—	—	—	—	—	—	—	—	—	0.0	—	—	—	—	—	—	—	0.0	—	—
	Total sugars	Median	—	—	—	—	—	—	—	—	—	—	—	0.0	—	—	—	—	—	—	—	0.0	—	—
	per 100	IQR	—	—	—	—	—	—	—	—	—	—	—	0.0	—	—	—	—	—	—	—	0.0	—	—
	Free sugars	Median	—	—	—	—	—	—	—	—	—	—	—	0.0	—	—	—	—	—	—	—	0.0	—	—
	per 100	IQR	—	—	—	—	—	—	—	—	—	—	—	0.0	—	—	—	—	—	—	—	0.0	—	—
Fats and oils	Number of products		0	0	0	1	0	0	0	0	0	0	101	105	1	0	0	0	9	0	0	56	0	16
(H)	Health Star	Mean	—	—	—	1.0	—	—	—	—	—	—	1.8	2.5	1.5	—	—	—	1.8	—	—	2.2	—	3.0
	Rating	SD	—	—	—	N/A	—	—	—	—	—	—	0.5	1.3	N/A	—	—	—	1.4	—	—	1.2	—	1.3
	Calories per	Median	—	—	—	900.0	—	—	—	—	—	—	300.0	666.7	400.0	—	—	—	700.0	—	—	500.0	—	633.3
	100 g/mL	IQR	—	—	—	0.0	—	—	—	—	—	—	200.0	466.7	0.0	—	—	—	100.0	—	—	416.7	—	354.2
	Sodium per	Median	—	—	—	0.0	—	—	—	—	—	—	900.0	600.0	1066.7	—	—	—	600.0	—	—	658.3	—	666.7
	100 g/mL	IQR	—	—	—	0.0	—	—	—	—	—	—	366.7	766.7	0.0	—	—	—	100.0	—	—	808.3	—	100.0
	Saturated	Median	—	—	—	40.0	—	—	—	—	—	—	2.7	6.7	3.3	—	—	—	35.0	—	—	6.7	—	10.0
	fat per 100	IQR	—	—	—	0.0	—	—	—	—	—	—	1.3	11.7	0.0	—	—	—	35.0	—	—	11.8	—	5.0
	Trans fat	Median	—	—	—	1.0	—	—	—	—	—	—	0.0	0.0	0.0	—	—	—	2.0	—	—	0.0	—	0.0
	per 100	IQR	—	—	—	0.0	—	—	—	—	—	—	0.0	0.7	0.0	—	—	—	2.0	—	—	0.0	—	0.0
	Total sugars	Median	—	—	—	0.0	—	—	—	—	—	—	6.7	0.0	0.0	—	—	—	0.0	—	—	0.0	—	0.0
	per 100	IQR	—	—	—	0.0	—	—	—	—	—	—	13.3	6.7	0.0	—	—	—	0.0	—	—	6.7	—	0.0
	Free sugars	Median	—	—	—	0.0	—	—	—	—	—	—	6.0	0.0	0.0	—	—	—	0.0	—	—	0.0	—	0.0
	per 100	IQR	—	—	—	0.0	—	—	—	—	—	—	13.3	6.0	0.0	—	—	—	0.0	—	—	6.0	—	0.0
Butter,	Number of products		0	0	0	1	0	0	0	0	0	0	0	19	0	0	0	0	9	0	0	12	0	12
margarine,	Health Star	Mean	—	—	—	1.0	—	—	—	—	—	—	—	2.1	—	—	—	—	1.8	—	—	2.0	—	3.5
shortening,	Rating	SD	—	—	—	N/A	—	—	—	—	—	—	—	1.4	—	—	—	—	1.4	—	—	1.2	—	1.0
lard, etc. (H1)	Calories per	Median	—	—	—	900.0	—	—	—	—	—	—	—	700.0	—	—	—	—	700.0	—	—	700.0	—	650.0
	100 g/mL	IQR	—	—	—	0.0	—	—	—	—	—	—	—	0.0	—	—	—	—	100.0	—	—	0.0	—	350.0
	Sodium per	Median	—	—	—	0.0	—	—	—	—	—	—	—	600.0	—	—	—	—	600.0	—	—	600.0	—	600.0
	100 g/mL	IQR	—	—	—	0.0	—	—	—	—	—	—	—	650.0	—	—	—	—	100.0	—	—	612.5	—	100.0
	Saturated fat	Median	—	—	—	40.0	—	—	—	—	—	—	—	15.0	—	—	—	—	35.0	—	—	22.5	—	10.0
	per 100 g/mL	IQR	—	—	—	0.0	—	—	—	—	—	—	—	40.0	—	—	—	—	35.0	—	—	32.5	—	1.3
	Trans fat per	Median	—	—	—	1.0	—	—	—	—	—	—	—	1.0	—	—	—	—	2.0	—	—	1.5	—	0.0
	100 g/mL (g)	IQR	—	—	—	0.0	—	—	—	—	—	—	—	3.0	—	—	—	—	2.0	—	—	3.0	—	0.3
	Total sugars	Median	—	—	—	0.0	—	—	—	—	—	—	—	0.0	—	—	—	—	0.0	—	—	0.0	—	0.0
	per 100 g/mL	IQR	—	—	—	0.0	—	—	—	—	—	—	—	0.0	—	—	—	—	0.0	—	—	0.0	—	0.0
	Free sugars	Median	—	—	—	0.0	—	—	—	—	—	—	—	0.0	—	—	—	—	0.0	—	—	0.0	—	0.0
	per 100 g/mL	IQR	—	—	—	0.0	—	—	—	—	—	—	—	0.0	—	—	—	—	0.0	—	—	0.0	—	0.0
Mayonnaise	Number of products		0	0	0	0	0	0	0	0	0	0	101	50	1	0	0	0	0	0	0	31	0	4

			A. Lassonde	Agropur	Campbell Soup	Canada Bread	Canada Dry Mott's	Coca-Cola	Danone	General Mills	George Weston	Kellogg	Kraft Heinz	Loblaw	Maple Leaf Foods	Mondelēz	Nestlé	Ocean Spray	Parmalat	PepsiCo	Saputo	Sobeys	Sun-Rype	Unilever
and salad dressings (H4, H5)	Health Star Rating	Mean	—	—	—	—	—	—	—	—	—	—	1.8	1.6	1.5	—	—	—	—	—	—	1.6	—	1.5
		SD	—	—	—	—	—	—	—	—	—	—	0.5	0.5	N/A	—	—	—	—	—	—	0.5	—	0.6
	Calories per 100 g/mL	Median	—	—	—	—	—	—	—	—	—	—	300.0	333.3	400.0	—	—	—	—	—	—	400.0	—	500.0
		IQR	—	—	—	—	—	—	—	—	—	—	200.0	220.8	0.0	—	—	—	—	—	—	216.7	—	350.0
	Sodium per 100 g/mL	Median	—	—	—	—	—	—	—	—	—	—	900.0	800.0	1066.7	—	—	—	—	—	—	800.0	—	750.0
		IQR	—	—	—	—	—	—	—	—	—	—	366.7	300.0	0.0	—	—	—	—	—	—	316.7	—	91.7
	Saturated fat per 100 g/mL	Median	—	—	—	—	—	—	—	—	—	—	2.7	3.3	3.3	—	—	—	—	—	—	3.3	—	4.7
		IQR	—	—	—	—	—	—	—	—	—	—	1.3	4.0	0.0	—	—	—	—	—	—	4.7	—	4.2
	Trans fat per 100 g/mL (g)	Median	—	—	—	—	—	—	—	—	—	—	0.0	0.0	0.0	—	—	—	—	—	—	0.0	—	0.0
		IQR	—	—	—	—	—	—	—	—	—	—	0.0	0.7	0.0	—	—	—	—	—	—	0.0	—	0.0
	Total sugars per 100 g/mL	Median	—	—	—	—	—	—	—	—	—	—	6.7	6.7	0.0	—	—	—	—	—	—	6.7	—	0.0
		IQR	—	—	—	—	—	—	—	—	—	—	13.3	6.7	0.0	—	—	—	—	—	—	13.3	—	0.0
	Free sugars per 100 g/mL	Median	—	—	—	—	—	—	—	—	—	—	6.0	6.3	0.0	—	—	—	—	—	—	6.0	—	0.0
		IQR	—	—	—	—	—	—	—	—	—	—	13.3	13.3	0.0	—	—	—	—	—	—	12.7	—	0.0
Oils (H2, H6)	Number of products		0	0	0	0	0	0	0	0	0	0	0	36	0	0	0	0	0	0	0	13	0	0
	Health Star Rating	Mean	—	—	—	—	—	—	—	—	—	—	—	3.9	—	—	—	—	—	—	—	4.0	—	—
		SD	—	—	—	—	—	—	—	—	—	—	—	0.6	—	—	—	—	—	—	—	0.8	—	—
	Calories per 100 g/mL	Median	—	—	—	—	—	—	—	—	—	—	—	800.0	—	—	—	—	—	—	—	800.0	—	—
		IQR	—	—	—	—	—	—	—	—	—	—	—	0.0	—	—	—	—	—	—	—	0.0	—	—
	Sodium per 100 g/mL	Median	—	—	—	—	—	—	—	—	—	—	—	0.0	—	—	—	—	—	—	—	0.0	—	—
		IQR	—	—	—	—	—	—	—	—	—	—	—	0.0	—	—	—	—	—	—	—	0.0	—	—
	Saturated fat per 100 g/mL	Median	—	—	—	—	—	—	—	—	—	—	—	12.5	—	—	—	—	—	—	—	15.0	—	—
		IQR	—	—	—	—	—	—	—	—	—	—	—	5.0	—	—	—	—	—	—	—	10.0	—	—
	Trans fat per 100 g/mL (g)	Median	—	—	—	—	—	—	—	—	—	—	—	0.0	—	—	—	—	—	—	—	0.0	—	—
		IQR	—	—	—	—	—	—	—	—	—	—	—	0.0	—	—	—	—	—	—	—	0.0	—	—
	Total sugars per 100 g/mL	Median	—	—	—	—	—	—	—	—	—	—	—	0.0	—	—	—	—	—	—	—	0.0	—	—
		IQR	—	—	—	—	—	—	—	—	—	—	—	0.0	—	—	—	—	—	—	—	0.0	—	—
	Free sugars per 100 g/mL	Median	—	—	—	—	—	—	—	—	—	—	—	0.0	—	—	—	—	—	—	—	0.0	—	—
		IQR	—	—	—	—	—	—	—	—	—	—	—	0.0	—	—	—	—	—	—	—	0.0	—	—
**Marine and fresh water animals (I)**	**Number of products**		**0**	**0**	**0**	**0**	**0**	**0**	**0**	**0**	**0**	**0**	**0**	**64**	**0**	**0**	**0**	**0**	**0**	**0**	**0**	**35**	**0**	**0**
	**Health Star Rating**	**Mean**	—	—	—	—	—	—	—	—	—	—	—	**3.9**	—	—	—	—	—	—	—	**3.8**	—	—
		**SD**	—	—	—	—	—	—	—	—	—	—	—	**0.6**	—	—	—	—	—	—	—	**0.6**	—	—
	**Calories per 100 g/mL**	**Median**	—	—	—	—	—	—	—	—	—	—	—	**105.3**	—	—	—	—	—	—	—	**100.0**	—	—
		**IQR**	—	—	—	—	—	—	—	—	—	—	—	**82.5**	—	—	—	—	—	—	—	**94.2**	—	—
	**Sodium per 100 g/mL**	**Median**	—	—	—	—	—	—	—	—	—	—	—	**297.9**	—	—	—	—	—	—	—	**330.0**	—	—
		**IQR**	—	—	—	—	—	—	—	—	—	—	—	**290.1**	—	—	—	—	—	—	—	**294.9**	—	—
	**Saturated fat per 100**	**Median**	—	—	—	—	—	—	—	—	—	—	—	**0.6**	—	—	—	—	—	—	—	**0.2**	—	—
		**IQR**	—	—	—	—	—	—	—	—	—	—	—	**1.2**	—	—	—	—	—	—	—	**1.1**	—	—
	**Trans fat per 100**	**Median**	—	—	—	—	—	—	—	—	—	—	—	**0.0**	—	—	—	—	—	—	—	**0.0**	—	—
		**IQR**	—	—	—	—	—	—	—	—	—	—	—	**0.0**	—	—	—	—	—	—	—	**0.0**	—	—
	**Total sugars per 100**	**Median**	—	—	—	—	—	—	—	—	—	—	—	**0.0**	—	—	—	—	—	—	—	**0.0**	—	—
		**IQR**	—	—	—	—	—	—	—	—	—	—	—	**0.7**	—	—	—	—	—	—	—	**3.8**	—	—
	**Free sugars per 100**	**Median**	—	—	—	—	—	—	—	—	—	—	—	**0.0**	—	—	—	—	—	—	—	**0.0**	—	—
		**IQR**	—	—	—	—	—	—	—	—	—	—	—	**0.0**	—	—	—	—	—	—	—	**3.6**	—	—
**Fruit and fruit juices (J)**	**Number of products**		**52**	**0**	**20**	**0**	**25**	**55**	**0**	**0**	**0**	**0**	**8**	**155**	**0**	**0**	**0**	**30**	**0**	**23**	**5**	**120**	**25**	**0**
	**Health Star Rating**	**Mean**	**2.3**	—	**2.0**	—	**3.2**	**2.6**	—	—	—	—	**1.4**	**3.0**	—	—	—	**2.5**	—	**4.2**	**1.5**	**2.8**	**2.6**	—
		**SD**	**1.1**	—	**0.6**	—	**1.1**	**1.6**	—	—	—	—	**0.5**	**1.3**	—	—	—	**1.0**	—	**1.4**	**0.5**	**1.3**	**1.3**	—
	**Calories per 100 g/mL**	**Median**	**44.0**	—	**52.0**	—	**45.0**	**48.0**	—	—	—	—	**38.9**	**52.0**	—	—	—	**50.0**	—	**44.0**	**44.0**	**52.0**	**48.0**	—
		**IQR**	**4.0**	—	**10.0**	—	**19.6**	**3.5**	—	—	—	—	**25.4**	**26.4**	—	—	—	**37.0**	—	**4.0**	**20.0**	**22.4**	**2.0**	—
	**Sodium per 100 g/mL**	**Median**	**9.0**	—	**16.0**	—	**0.0**	**2.0**	—	—	—	—	**8.3**	**6.0**	—	—	—	**14.0**	—	**0.0**	**10.0**	**6.7**	**10.0**	—
		**IQR**	**7.5**	—	**22.0**	—	**13.5**	**5.5**	—	—	—	—	**5.3**	**12.0**	—	—	—	**13.6**	—	**4.0**	**58.0**	**14.0**	**2.0**	—
	**Saturated fat per 100**	**Median**	**0.0**	—	**0.0**	—	**0.0**	**0.0**	—	—	—	—	**0.0**	**0.0**	—	—	—	**0.0**	—	**0.0**	**0.0**	**0.0**	**0.0**	—
		**IQR**	**0.0**	—	**0.0**	—	**0.0**	**0.0**	—	—	—	—	**0.0**	**0.0**	—	—	—	**0.0**	—	**0.0**	**0.0**	**0.0**	**0.0**	—
	**Trans fat per 100**	**Median**	**0.0**	—	**0.0**	—	**0.0**	**0.0**	—	—	—	—	**0.0**	**0.0**	—	—	—	**0.0**	—	**0.0**	**0.0**	**0.0**	**0.0**	—
		**IQR**	**0.0**	—	**0.0**	—	**0.0**	**0.0**	—	—	—	—	**0.0**	**0.0**	—	—	—	**0.0**	—	**0.0**	**0.0**	**0.0**	**0.0**	—
	**Total sugars per 100**	**Median**	**10.0**	—	**10.6**	—	**9.9**	**10.8**	—	—	—	—	**9.4**	**10.4**	—	—	—	**12.8**	—	**9.2**	**10.4**	**11.6**	**12.0**	—
		**IQR**	**0.4**	—	**3.3**	—	**6.7**	**2.0**	—	—	—	—	**6.7**	**6.2**	—	—	—	**9.1**	—	**1.8**	**4.8**	**5.1**	**1.2**	—
	**Free sugars per 100**	**Median**	**10.0**	—	**10.6**	—	**0.0**	**10.8**	—	—	—	—	**9.4**	**9.2**	—	—	—	**12.2**	—	**9.2**	**10.4**	**10.0**	**12.0**	—
		**IQR**	**0.4**	—	**3.3**	—	**11.2**	**2.0**	—	—	—	—	**6.7**	**11.1**	—	—	—	**10.4**	—	**1.8**	**4.8**	**6.3**	**1.2**	—
Fruit juices, nectars and fruit drinks (J11)	Number of products		52	0	20	0	8	55	0	0	0	0	8	82	0	0	0	25	0	23	5	68	25	0
	Health Star Rating	Mean	2.3	—	2.0	—	1.8	2.6	—	—	—	—	1.4	2.3	—	—	—	2.5	—	4.2	1.5	2.1	2.6	—
		SD	1.1	—	0.6	—	0.7	1.6	—	—	—	—	0.5	1.2	—	—	—	1.1	—	1.4	0.5	1.2	1.3	—
	Calories per 100 g/mL	Median	44.0	—	52.0	—	64.0	48.0	—	—	—	—	38.9	48.0	—	—	—	48.0	—	44.0	44.0	48.0	48.0	—
		IQR	4.0	—	10.0	—	41.0	3.5	—	—	—	—	25.4	11.0	—	—	—	40.0	—	4.0	20.0	12.0	2.0	—
	Sodium per 100 g/mL	Median	9.0	—	16.0	—	17.0	2.0	—	—	—	—	8.3	8.0	—	—	—	16.0	—	0.0	10.0	7.5	10.0	—
		IQR	7.5	—	22.0	—	34.2	5.5	—	—	—	—	5.3	8.0	—	—	—	10.0	—	4.0	58.0	12.0	2.0	—
	Saturated fat per 100 g/mL	Median	0.0	—	0.0	—	0.0	0.0	—	—	—	—	0.0	0.0	—	—	—	0.0	—	0.0	0.0	0.0	0.0	—
		IQR	0.0	—	0.0	—	0.0	0.0	—	—	—	—	0.0	0.0	—	—	—	0.0	—	0.0	0.0	0.0	0.0	—
	Trans fat per 100 g/mL (g)	Median	0.0	—	0.0	—	0.0	0.0	—	—	—	—	0.0	0.0	—	—	—	0.0	—	0.0	0.0	0.0	0.0	—
		IQR	0.0	—	0.0	—	0.0	0.0	—	—	—	—	0.0	0.0	—	—	—	0.0	—	0.0	0.0	0.0	0.0	—
	Total sugars per 100 g/mL	Median	10.0	—	10.6	—	14.2	10.8	—	—	—	—	9.4	9.6	—	—	—	11.6	—	9.2	10.4	11.1	12.0	—
		IQR	0.4	—	3.3	—	8.7	2.0	—	—	—	—	6.7	2.4	—	—	—	9.2	—	1.8	4.8	2.4	1.2	—
	Free sugars	Median	10.0	—	10.6	—	14.2	10.8	—	—	—	—	9.4	9.6	—	—	—	11.6	—	9.2	10.4	11.1	12.0	—

			A. Lassonde	Agropur	Campbell Soup	Canada Bread	Canada Dry Mott's	Coca-Cola	Danone	General Mills	George Weston	Kellogg	Kraft Heinz	Loblaw	Maple Leaf Foods	Mondelēz	Nestlé	Ocean Spray	Parmalat	PepsiCo	Saputo	Sobeys	Sun-Rype	Unilever
	per 100 g/mL	IQR	0.4	—	3.3	—	8.7	2.0	—	—	—	—	6.7	2.4	—	—	—	9.2	—	1.8	4.8	2.4	1.2	—
Fruit, frozen or	Number of products		0	0	0	0	0	0	0	0	0	0	0	36	0	0	0	0	0	0	0	24	0	0
canned, coated	Health Star	Mean	—	—	—	—	—	—	—	—	—	—	—	4.1	—	—	—	—	—	—	—	4.0	—	—
or uncoated	Rating	SD	—	—	—	—	—	—	—	—	—	—	—	0.6	—	—	—	—	—	—	—	0.6	—	—
(J1-J5)	Calories per	Median	—	—	—	—	—	—	—	—	—	—	—	57.1	—	—	—	—	—	—	—	60.0	—	—
	100 g/mL	IQR	—	—	—	—	—	—	—	—	—	—	—	23.8	—	—	—	—	—	—	—	11.0	—	—
	Sodium per	Median	—	—	—	—	—	—	—	—	—	—	—	0.0	—	—	—	—	—	—	—	6.7	—	—
	100 g/mL	IQR	—	—	—	—	—	—	—	—	—	—	—	4.0	—	—	—	—	—	—	—	8.5	—	—
	Saturated fat	Median	—	—	—	—	—	—	—	—	—	—	—	0.0	—	—	—	—	—	—	—	0.0	—	—
	per 100 g/mL	IQR	—	—	—	—	—	—	—	—	—	—	—	0.0	—	—	—	—	—	—	—	0.0	—	—
	Trans fat per	Median	—	—	—	—	—	—	—	—	—	—	—	0.0	—	—	—	—	—	—	—	0.0	—	—
	100 g/mL (g)	IQR	—	—	—	—	—	—	—	—	—	—	—	0.0	—	—	—	—	—	—	—	0.0	—	—
	Total sugars	Median	—	—	—	—	—	—	—	—	—	—	—	9.0	—	—	—	—	—	—	—	12.7	—	—
	per 100 g/mL	IQR	—	—	—	—	—	—	—	—	—	—	—	8.4	—	—	—	—	—	—	—	4.3	—	—
	Free sugars	Median	—	—	—	—	—	—	—	—	—	—	—	0.0	—	—	—	—	—	—	—	6.1	—	—
	per 100 g/mL	IQR	—	—	—	—	—	—	—	—	—	—	—	9.4	—	—	—	—	—	—	—	9.6	—	—
Other, e.g.,	Number of products		0	0	0	0	17	0	0	0	0	0	0	37	0	0	0	5	0	0	0	28	0	0
apple sauce,	Health Star	Mean	—	—	—	—	3.9	—	—	—	—	—	—	3.4	—	—	—	2.7	—	—	—	3.4	—	—
dried or	Rating	SD	—	—	—	—	0.3	—	—	—	—	—	—	1.0	—	—	—	0.4	—	—	—	0.8	—	—
candied fruit,	Calories per	Median	—	—	—	—	45.0	—	—	—	—	—	—	275.0	—	—	—	350.0	—	—	—	290.0	—	—
fruit relishes	100 g/mL	IQR	—	—	—	—	9.0	—	—	—	—	—	—	245.0	—	—	—	25.0	—	—	—	252.3	—	—
(J6, J7, J8, J9,	Sodium per	Median	—	—	—	—	0.0	—	—	—	—	—	—	16.7	—	—	—	0.0	—	—	—	0.0	—	—
J10, J12)	100 g/mL	IQR	—	—	—	—	0.0	—	—	—	—	—	—	20.0	—	—	—	0.0	—	—	—	10.6	—	—
	Saturated fat	Median	—	—	—	—	0.0	—	—	—	—	—	—	0.0	—	—	—	0.0	—	—	—	0.0	—	—
	per 100 g/mL	IQR	—	—	—	—	0.0	—	—	—	—	—	—	0.0	—	—	—	0.0	—	—	—	0.0	—	—
	Trans fat per	Median	—	—	—	—	0.0	—	—	—	—	—	—	0.0	—	—	—	0.0	—	—	—	0.0	—	—
	100 g/mL (g)	IQR	—	—	—	—	0.0	—	—	—	—	—	—	0.0	—	—	—	0.0	—	—	—	0.0	—	—
	Total sugars	Median	—	—	—	—	9.9	—	—	—	—	—	—	37.5	—	—	—	65.0	—	—	—	23.8	—	—
	per 100 g/mL	IQR	—	—	—	—	0.9	—	—	—	—	—	—	47.3	—	—	—	0.0	—	—	—	30.0	—	—
	Free sugars	Median	—	—	—	—	0.0	—	—	—	—	—	—	0.0	—	—	—	32.0	—	—	—	0.0	—	—
	per 100 g/mL	IQR	—	—	—	—	0.0	—	—	—	—	—	—	7.5	—	—	—	0.0	—	—	—	6.7	—	—
**Legumes,**	**Number of products**		**0**	**0**	**0**	**0**	**0**	**0**	**0**	**1**	**0**	**0**	**2**	**43**	**0**	**0**	**0**	**0**	**0**	**0**	**0**	**12**	**0**	**0**
**includes tofu**	**Health Star**	**Mean**	—	—	—	—	—	—	—	**5.0**	—	—	**4.0**	**4.8**	—	—	—	—	—	—	—	**4.7**	—	—
**and tempeh**	**Rating**	**SD**	—	—	—	—	—	—	—	**N/A**	—	—	**0.0**	**0.3**	—	—	—	—	—	—	—	**0.3**	—	—
**(K)**	**Calories per**	**Median**	—	—	—	—	—	—	—	**110.0**	—	—	**76.0**	**100.0**	—	—	—	—	—	—	—	**90.0**	—	—
	**100 g/mL**	**IQR**	—	—	—	—	—	—	—	**0.0**	—	—	**4.0**	**266.0**	—	—	—	—	—	—	—	**23.4**	—	—
	**Sodium per**	**Median**	—	—	—	—	—	—	—	**5.0**	—	—	**148.0**	**5.2**	—	—	—	—	—	—	—	**118.0**	—	—
	**100 g/mL**	**IQR**	—	—	—	—	—	—	—	**0.0**	—	—	**4.0**	**80.0**	—	—	—	—	—	—	—	**144.0**	—	—
	**Saturated**	**Median**	—	—	—	—	—	—	—	**0.0**	—	—	**0.1**	**0.2**	—	—	—	—	—	—	—	**0.2**	—	—
	**fat per 100**	**IQR**	—	—	—	—	—	—	—	**0.0**	—	—	**0.0**	**0.2**	—	—	—	—	—	—	—	**0.1**	—	—
	**Trans fat**	**Median**	—	—	—	—	—	—	—	**0.0**	—	—	**0.0**	**0.0**	—	—	—	—	—	—	—	**0.0**	—	—
	**per 100**	**IQR**	—	—	—	—	—	—	—	**0.0**	—	—	**0.0**	**0.0**	—	—	—	—	—	—	—	**0.0**	—	—
	**Total sugars**	**Median**	—	—	—	—	—	—	—	**3.0**	—	—	**0.4**	**0.8**	—	—	—	—	—	—	—	**0.4**	—	—
	**per 100**	**IQR**	—	—	—	—	—	—	—	**0.0**	—	—	**0.4**	**1.7**	—	—	—	—	—	—	—	**0.5**	—	—
	**Free sugars**	**Median**	—	—	—	—	—	—	—	**0.0**	—	—	**0.0**	**0.0**	—	—	—	—	—	—	—	**0.0**	—	—
	**per 100**	**IQR**	—	—	—	—	—	—	—	**0.0**	—	—	**0.0**	**0.0**	—	—	—	—	—	—	—	**0.0**	—	—
**Meat,**	**Number of products**		**0**	**0**	**0**	**0**	**0**	**0**	**0**	**0**	**0**	**0**	**0**	**169**	**138**	**0**	**0**	**0**	**0**	**0**	**0**	**81**	**0**	**0**
**poultry, their**	**Health Star**	**Mean**	—	—	—	—	—	—	—	—	—	—	—	**2.6**	**2.3**	—	—	—	—	—	—	**2.8**	—	—
**products and**	**Rating**	**SD**	—	—	—	—	—	—	—	—	—	—	—	**1.2**	**1.1**	—	—	—	—	—	—	**1.0**	—	—
**substitutes**	**Calories per**	**Median**	—	—	—	—	—	—	—	—	—	—	—	**218.3**	**212.9**	—	—	—	—	—	—	**200.0**	—	—
**(L)**	**100 g/mL**	**IQR**	—	—	—	—	—	—	—	—	—	—	—	**109.3**	**165.1**	—	—	—	—	—	—	**124.4**	—	—
	**Sodium per**	**Median**	—	—	—	—	—	—	—	—	—	—	—	**490.0**	**834.6**	—	—	—	—	—	—	**592.6**	—	—
	**100 g/mL**	**IQR**	—	—	—	—	—	—	—	—	—	—	—	**457.6**	**278.4**	—	—	—	—	—	—	**428.8**	—	—
	**Saturated**	**Median**	—	—	—	—	—	—	—	—	—	—	—	**4.4**	**4.6**	—	—	—	—	—	—	**3.3**	—	—
	**fat per 100**	**IQR**	—	—	—	—	—	—	—	—	—	—	—	**7.1**	**8.0**	—	—	—	—	—	—	**6.6**	—	—
	**Trans fat**	**Median**	—	—	—	—	—	—	—	—	—	—	—	**0.1**	**0.0**	—	—	—	—	—	—	**0.0**	—	—
	**per 100**	**IQR**	—	—	—	—	—	—	—	—	—	—	—	**0.3**	**0.1**	—	—	—	—	—	—	**0.2**	—	—
	**Total sugars**	**Median**	—	—	—	—	—	—	—	—	—	—	—	**0.0**	**0.0**	—	—	—	—	—	—	**0.0**	—	—
	**per 100**	**IQR**	—	—	—	—	—	—	—	—	—	—	—	**1.9**	**1.8**	—	—	—	—	—	—	**1.8**	—	—
	**Free sugars**	**Median**	—	—	—	—	—	—	—	—	—	—	—	**0.0**	**0.0**	—	—	—	—	—	—	**0.0**	—	—
	**per 100**	**IQR**	—	—	—	—	—	—	—	—	—	—	—	**1.7**	**1.6**	—	—	—	—	—	—	**1.7**	—	—
**Miscellaneous**	**Number of products**		**0**	**0**	**0**	**0**	**1**	**0**	**0**	**28**	**0**	**1**	**17**	**66**	**2**	**0**	**3**	**0**	**0**	**17**	**0**	**21**	**0**	**2**
**(M)**	**Health Star**	**Mean**	—	—	—	—	**0.5**	—	—	**1.6**	—	**2.0**	**0.9**	**2.1**	**0.5**	—	**0.8**	—	—	**2.5**	—	**2.0**	—	**0.5**
	**Rating**	**SD**	—	—	—	—	**N/A**	—	—	**0.8**	—	**N/A**	**0.7**	**1.4**	**0.0**	—	**0.3**	—	—	**0.6**	—	**1.0**	—	**0.0**
	**Calories per**	**Median**	—	—	—	—	**0.0**	—	—	**301.2**	—	**357.1**	**375.0**	**335.6**	**437.5**	—	**294.1**	—	—	**254.2**	—	**291.1**	—	**381.8**
	**100 g/mL**	**IQR**	—	—	—	—	**0.0**	—	—	**49.3**	—	**0.0**	**38.5**	**240.8**	**0.0**	—	**8.2**	—	—	**100.4**	—	**118.0**	—	**18.2**
	**Sodium per**	**Median**	—	—	—	—	**30000.0**	—	—	**382.7**	—	**642.9**	**2428.6**	**471.4**	**2250.0**	—	**8470.6**	—	—	**393.4**	—	**599.0**	—	**6109.1**
	**100 g/mL**	**IQR**	—	—	—	—	**0.0**	—	—	**2999.9**	—	**0.0**	**1309.5**	**4376.1**	**125.0**	—	**967.3**	—	—	**107.0**	—	**2684.6**	—	**290.9**
	**Saturated**	**Median**	—	—	—	—	**0.0**	—	—	**2.1**	—	**0.0**	**3.1**	**0.4**	**9.4**	—	**0.0**	—	—	**0.8**	—	**1.9**	—	**1.9**
	**fat per 100**	**IQR**	—	—	—	—	**0.0**	—	—	**2.5**	—	**0.0**	**1.3**	**3.8**	**3.1**	—	**0.6**	—	—	**1.9**	—	**2.1**	—	**0.1**
	**Trans fat**	**Median**	—	—	—	—	**0.0**	—	—	**0.0**	—	**0.0**	**0.0**	**0.0**	**0.0**	—	**0.0**	—	—	**0.0**	—	**0.0**	—	**0.0**
	**per 100**	**IQR**	—	—	—	—	**0.0**	—	—	**0.3**	—	**0.0**	**0.0**	**0.0**	**0.0**	—	**0.0**	—	—	**0.0**	—	**0.0**	—	**0.0**
	**Total sugars**	**Median**	—	—	—	—	**0.0**	—	—	**19.1**	—	**10.7**	**0.0**	**0.0**	**0.0**	—	**11.8**	—	—	**22.0**	—	**14.3**	—	**19.1**

			A. Lassonde	Agropur	Campbell Soup	Canada Bread	Canada Dry Mott's	Coca-Cola	Danone	General Mills	George Weston	Kellogg	Kraft Heinz	Loblaw	Maple Leaf Foods	Mondelēz	Nestlé	Ocean Spray	Parmalat	PepsiCo	Saputo	Sobeys	Sun-Rype	Unilever
	**per 100**	**IQR**	—	—	—	—	0.0	—	—	12.1	—	0.0	8.3	17.6	0.0	—	5.2	—	—	8.9	—	16.1	—	0.9
	**Free sugars**	**Median**	—	—	—	—	0.0	—	—	18.2	—	10.0	0.0	0.0	0.0	—	10.6	—	—	20.8	—	14.3	—	19.1
	**per 100**	**IQR**	—	—	—	—	0.0	—	—	13.2	—	0.0	7.5	14.7	0.0	—	5.3	—	—	9.0	—	19.4	—	0.9
Baking/cooking ingredients, e.g., baking powder, yeast, sprinkles, bread crumbs, cocoa powder, etc. (M1, M3, M5, M8, M11, M12)	Number of products		0	0	0	0	0	0	0	20	0	1	17	42	2	0	0	0	0	17	0	18	0	0
	Health Star	Mean	—	—	—	—	—	—	—	2.0	—	2.0	0.9	2.3	0.5	—	—	—	—	2.5	—	2.1	—	—
	Rating	SD	—	—	—	—	—	—	—	0.6	—	N/A	0.7	1.1	0.0	—	—	—	—	0.6	—	1.0	—	—
	Calories per	Median	—	—	—	—	—	—	—	294.7	—	357.1	375.0	369.8	437.5	—	—	—	—	254.2	—	290.3	—	—
	100 g/mL	IQR	—	—	—	—	—	—	—	101.7	—	0.0	38.5	203.1	0.0	—	—	—	—	100.4	—	131.7	—	—
	Sodium per	Median	—	—	—	—	—	—	—	353.1	—	642.9	2428.6	379.6	2250.0	—	—	—	—	393.4	—	554.8	—	—
	100 g/mL	IQR	—	—	—	—	—	—	—	53.1	—	0.0	1309.5	435.2	125.0	—	—	—	—	107.0	—	1495.2	—	—
	Saturated fat	Median	—	—	—	—	—	—	—	2.1	—	0.0	3.1	2.3	9.4	—	—	—	—	0.8	—	2.3	—	—
	per 100 g/mL	IQR	—	—	—	—	—	—	—	2.6	—	0.0	1.3	5.9	3.1	—	—	—	—	1.9	—	2.6	—	—
	Trans fat per	Median	—	—	—	—	—	—	—	0.0	—	0.0	0.0	0.0	0.0	—	—	—	—	0.0	—	0.0	—	—
	100 g/mL (g)	IQR	—	—	—	—	—	—	—	0.5	—	0.0	0.0	0.0	0.0	—	—	—	—	0.0	—	0.0	—	—
	Total sugars	Median	—	—	—	—	—	—	—	20.0	—	10.7	0.0	8.3	0.0	—	—	—	—	22.0	—	14.6	—	—
	per 100 g/mL	IQR	—	—	—	—	—	—	—	14.6	—	0.0	8.3	23.7	0.0	—	—	—	—	8.9	—	17.5	—	—
	Free sugars	Median	—	—	—	—	—	—	—	19.1	—	10.0	0.0	6.0	0.0	—	—	—	—	20.8	—	14.2	—	—
	per 100 g/mL	IQR	—	—	—	—	—	—	—	15.5	—	0.0	7.5	23.7	0.0	—	—	—	—	9.0	—	20.2	—	—
Seasoning salts and mixes (M9, M10)	Number of products		0	0	0	0	1	0	0	8	0	0	0	24	0	0	3	0	0	0	0	3	0	2
	Health Star	Mean	—	—	—	—	0.5	—	—	0.7	—	—	—	1.9	—	—	0.8	—	—	—	—	1.3	—	0.5
	Rating	SD	—	—	—	—	N/A	—	—	0.4	—	—	—	1.7	—	—	0.3	—	—	—	—	0.8	—	0.0
	Calories per	Median	—	—	—	—	0.0	—	—	333.3	—	—	—	0.0	—	—	294.1	—	—	—	—	307.7	—	381.8
	100 g/mL	IQR	—	—	—	—	0.0	—	—	6.4	—	—	—	375.0	—	—	8.2	—	—	—	—	35.7	—	18.2
	Sodium per	Median	—	—	—	—	30000.0	—	—	5500.0	—	—	—	5437.5	—	—	8470.6	—	—	—	—	5538.5	—	6109.1
	100 g/mL	IQR	—	—	—	—	0.0	—	—	1305.6	—	—	—	14000.0	—	—	967.3	—	—	—	—	1571.4	—	290.9
	Saturated fat	Median	—	—	—	—	0.0	—	—	0.0	—	—	—	0.0	—	—	0.0	—	—	—	—	0.0	—	1.9
	per 100 g/mL	IQR	—	—	—	—	0.0	—	—	0.0	—	—	—	0.0	—	—	0.6	—	—	—	—	0.7	—	0.1
	Trans fat per	Median	—	—	—	—	0.0	—	—	0.0	—	—	—	0.0	—	—	0.0	—	—	—	—	0.0	—	0.0
	100 g/mL (g)	IQR	—	—	—	—	0.0	—	—	0.0	—	—	—	0.0	—	—	0.0	—	—	—	—	0.0	—	0.0
	Total sugars	Median	—	—	—	—	0.0	—	—	0.0	—	—	—	0.0	—	—	11.8	—	—	—	—	14.3	—	19.1
	per 100 g/mL	IQR	—	—	—	—	0.0	—	—	2.8	—	—	—	0.0	—	—	5.2	—	—	—	—	0.5	—	0.9
	Free sugars	Median	—	—	—	—	0.0	—	—	0.0	—	—	—	0.0	—	—	10.6	—	—	—	—	14.3	—	19.1
	per 100 g/mL	IQR	—	—	—	—	0.0	—	—	2.5	—	—	—	0.0	—	—	5.3	—	—	—	—	0.5	—	0.9
**Combination dishes (N)**	**Number of products**		0	0	1	3	0	0	0	38	0	3	66	202	26	0	83	0	0	0	0	91	0	21
	**Health Star**	**Mean**	—	—	4.0	3.5	—	—	—	3.0	—	3.0	3.5	3.2	2.8	—	3.3	—	—	—	—	3.4	—	3.4
	**Rating**	**SD**	—	—	N/A	0.0	—	—	—	0.7	—	0.0	0.4	0.7	0.8	—	0.6	—	—	—	—	0.4	—	0.2
	**Calories per**	**Median**	—	—	96.0	222.2	—	—	—	173.0	—	186.3	118.4	163.9	253.6	—	195.3	—	—	—	—	158.3	—	109.1
	**100 g/mL**	**IQR**	—	—	0.0	11.1	—	—	—	148.7	—	14.0	60.6	134.4	131.8	—	120.3	—	—	—	—	109.8	—	9.1
	**Sodium per**	**Median**	—	—	260.0	333.3	—	—	—	471.2	—	706.9	278.6	312.3	456.7	—	364.0	—	—	—	—	323.3	—	244.0
	**100 g/mL**	**IQR**	—	—	0.0	27.8	—	—	—	407.5	—	89.4	58.4	205.5	208.9	—	270.7	—	—	—	—	166.1	—	39.8
	**Saturated**	**Median**	—	—	1.4	3.3	—	—	—	1.5	—	3.4	0.8	1.9	3.5	—	2.6	—	—	—	—	1.3	—	0.7
	**fat per 100**	**IQR**	—	—	0.0	0.6	—	—	—	1.8	—	0.1	0.8	3.7	4.3	—	2.8	—	—	—	—	2.2	—	0.6
	**Trans fat**	**Median**	—	—	0.2	0.1	—	—	—	0.1	—	0.1	0.0	0.1	0.1	—	0.1	—	—	—	—	0.1	—	0.0
	**per 100**	**IQR**	—	—	0.0	0.0	—	—	—	0.2	—	0.0	0.1	0.2	0.3	—	0.2	—	—	—	—	0.1	—	0.0
	**Total sugars**	**Median**	—	—	2.8	4.4	—	—	—	2.4	—	2.9	4.2	2.4	6.8	—	2.9	—	—	—	—	2.7	—	3.0
	**per 100**	**IQR**	—	—	0.0	1.7	—	—	—	1.5	—	0.3	3.0	2.0	6.6	—	2.8	—	—	—	—	2.2	—	0.6
	**Free sugars**	**Median**	—	—	1.0	0.0	—	—	—	0.6	—	2.2	1.5	1.0	5.2	—	2.1	—	—	—	—	1.3	—	1.2
	**per 100**	**IQR**	—	—	0.0	1.4	—	—	—	1.4	—	0.3	2.9	2.1	6.3	—	2.9	—	—	—	—	2.5	—	1.3
**Nuts and seeds (O)**	**Number of products**		0	0	0	0	0	0	0	0	0	0	11	29	0	0	0	0	0	0	0	12	0	5
	**Health Star**	**Mean**	—	—	—	—	—	—	—	—	—	—	3.0	4.4	—	—	—	—	—	—	—	4.1	—	2.1
	**Rating**	**SD**	—	—	—	—	—	—	—	—	—	—	1.2	0.9	—	—	—	—	—	—	—	1.3	—	0.2
	**Calories per**	**Median**	—	—	—	—	—	—	—	—	—	—	538.5	666.7	—	—	—	—	—	—	—	666.7	—	600.0
	**100 g/mL**	**IQR**	—	—	—	—	—	—	—	—	—	—	66.7	66.7	—	—	—	—	—	—	—	30.0	—	66.7
	**Sodium per**	**Median**	—	—	—	—	—	—	—	—	—	—	366.7	0.0	—	—	—	—	—	—	—	0.0	—	433.3
	**100 g/mL**	**IQR**	—	—	—	—	—	—	—	—	—	—	233.3	16.7	—	—	—	—	—	—	—	96.7	—	33.3
	**Saturated**	**Median**	—	—	—	—	—	—	—	—	—	—	6.7	6.3	—	—	—	—	—	—	—	6.8	—	10.0
	**fat per 100**	**IQR**	—	—	—	—	—	—	—	—	—	—	2.2	3.3	—	—	—	—	—	—	—	2.6	—	0.0
	**Trans fat**	**Median**	—	—	—	—	—	—	—	—	—	—	0.0	0.0	—	—	—	—	—	—	—	0.0	—	0.0
	**per 100**	**IQR**	—	—	—	—	—	—	—	—	—	—	0.0	0.0	—	—	—	—	—	—	—	0.0	—	0.0
	**Total sugars**	**Median**	—	—	—	—	—	—	—	—	—	—	6.7	3.3	—	—	—	—	—	—	—	5.3	—	6.7
	**per 100**	**IQR**	—	—	—	—	—	—	—	—	—	—	10.0	3.9	—	—	—	—	—	—	—	2.7	—	0.0
	**Free sugars**	**Median**	—	—	—	—	—	—	—	—	—	—	2.0	0.0	—	—	—	—	—	—	—	0.0	—	2.0
	**per 100**	**IQR**	—	—	—	—	—	—	—	—	—	—	11.7	0.0	—	—	—	—	—	—	—	0.5	—	0.0
Nut butters, pastes and creams (O2, O3)	Number of products		0	0	0	0	0	0	0	0	0	0	11	9	0	0	0	0	0	0	0	6	0	5
	Health Star	Mean	—	—	—	—	—	—	—	—	—	—	3.0	3.8	—	—	—	—	—	—	—	3.4	—	2.1
	Rating	SD	—	—	—	—	—	—	—	—	—	—	1.2	1.3	—	—	—	—	—	—	—	1.6	—	0.2
	Calories per	Median	—	—	—	—	—	—	—	—	—	—	538.5	666.7	—	—	—	—	—	—	—	666.7	—	600.0
	100 g/mL	IQR	—	—	—	—	—	—	—	—	—	—	66.7	0.0	—	—	—	—	—	—	—	0.0	—	66.7
	Sodium per	Median	—	—	—	—	—	—	—	—	—	—	366.7	21.4	—	—	—	—	—	—	—	133.3	—	433.3
	100 g/mL	IQR	—	—	—	—	—	—	—	—	—	—	233.3	321.4	—	—	—	—	—	—	—	291.7	—	33.3
	Saturated fat	Median	—	—	—	—	—	—	—	—	—	—	6.7	6.7	—	—	—	—	—	—	—	6.7	—	10.0
	per 100 g/mL	IQR	—	—	—	—	—	—	—	—	—	—	2.2	0.5	—	—	—	—	—	—	—	2.5	—	0.0
	Trans fat per	Median	—	—	—	—	—	—	—	—	—	—	0.0	0.0	—	—	—	—	—	—	—	0.0	—	0.0

			A. Lassonde	Agropur	Campbell Soup	Canada Bread	Canada Dry Mott's	Coca-Cola	Danone	General Mills	George Weston	Kellogg	Kraft Heinz	Loblaw	Maple Leaf Foods	Mondelēz	Nestlé	Ocean Spray	Parmalat	PepsiCo	Saputo	Sobeys	Sun-Rype	Unilever
	100 g/mL (g)	IQR	—	—	—	—	—	—	—	—	—	—	0.0	0.0	—	—	—	—	—	—	—	0.0	—	0.0
	Total sugars	Median	—	—	—	—	—	—	—	—	—	—	6.7	7.1	—	—	—	—	—	—	—	6.7	—	6.7
	per 100 g/mL	IQR	—	—	—	—	—	—	—	—	—	—	10.0	0.5	—	—	—	—	—	—	—	5.0	—	0.0
	Free sugars	Median	—	—	—	—	—	—	—	—	—	—	2.0	0.0	—	—	—	—	—	—	—	1.0	—	2.0
	per 100 g/mL	IQR	—	—	—	—	—	—	—	—	—	—	11.7	2.9	—	—	—	—	—	—	—	7.5	—	0.0
Nuts, seeds and flours (O1, O4)	Number of products		0	0	0	0	0	0	0	0	0	0	0	20	0	0	0	0	0	0	0	6	0	0
	Health Star	Mean	—	—	—	—	—	—	—	—	—	—	—	4.7	—	—	—	—	—	—	—	4.8	—	—
	Rating	SD	—	—	—	—	—	—	—	—	—	—	—	0.4	—	—	—	—	—	—	—	0.3	—	—
	Calories per	Median	—	—	—	—	—	—	—	—	—	—	—	636.7	—	—	—	—	—	—	—	660.0	—	—
	100 g/mL	IQR	—	—	—	—	—	—	—	—	—	—	—	66.7	—	—	—	—	—	—	—	70.0	—	—
	Sodium per	Median	—	—	—	—	—	—	—	—	—	—	—	0.0	—	—	—	—	—	—	—	0.0	—	—
	100 g/mL	IQR	—	—	—	—	—	—	—	—	—	—	—	13.5	—	—	—	—	—	—	—	0.0	—	—
	Saturated fat	Median	—	—	—	—	—	—	—	—	—	—	—	5.6	—	—	—	—	—	—	—	7.5	—	—
	per 100 g/mL	IQR	—	—	—	—	—	—	—	—	—	—	—	3.1	—	—	—	—	—	—	—	3.3	—	—
	Trans fat per	Median	—	—	—	—	—	—	—	—	—	—	—	0.0	—	—	—	—	—	—	—	0.0	—	—
	100 g/mL (g)	IQR	—	—	—	—	—	—	—	—	—	—	—	0.0	—	—	—	—	—	—	—	0.2	—	—
	Total sugars	Median	—	—	—	—	—	—	—	—	—	—	—	3.3	—	—	—	—	—	—	—	4.0	—	—
	per 100 g/mL	IQR	—	—	—	—	—	—	—	—	—	—	—	1.0	—	—	—	—	—	—	—	1.5	—	—
	Free sugars	Median	—	—	—	—	—	—	—	—	—	—	—	0.0	—	—	—	—	—	—	—	0.0	—	—
	per 100 g/mL	IQR	—	—	—	—	—	—	—	—	—	—	—	0.0	—	—	—	—	—	—	—	0.0	—	—
**Potatoes (P)**	**Number of products**		**0**	**0**	**0**	**0**	**0**	**0**	**0**	**8**	**0**	**0**	**0**	**12**	**0**	**0**	**0**	**0**	**0**	**0**	**0**	**16**	**0**	**4**
	**Health Star**	**Mean**	—	—	—	—	—	—	—	**3.3**	—	—	—	**3.9**	—	—	—	—	—	—	—	**3.8**	—	**3.5**
	**Rating**	**SD**	—	—	—	—	—	—	—	**0.3**	—	—	—	**0.3**	—	—	—	—	—	—	—	**0.5**	—	**0.0**
	**Calories per**	**Median**	—	—	—	—	—	—	—	**100.2**	—	—	—	**117.8**	—	—	—	—	—	—	—	**112.4**	—	**100.6**
	**100 g/mL**	**IQR**	—	—	—	—	—	—	—	**12.9**	—	—	—	**61.1**	—	—	—	—	—	—	—	**32.4**	—	**1.7**
	**Sodium per**	**Median**	—	—	—	—	—	—	—	**331.6**	—	—	—	**200.0**	—	—	—	—	—	—	—	**229.8**	—	**214.6**
	**100 g/mL**	**IQR**	—	—	—	—	—	—	—	**51.7**	—	—	—	**174.4**	—	—	—	—	—	—	—	**208.9**	—	**115.1**
	**Saturated**	**Median**	—	—	—	—	—	—	—	**0.7**	—	—	—	**0.5**	—	—	—	—	—	—	—	**0.7**	—	**0.6**
	**fat per 100**	**IQR**	—	—	—	—	—	—	—	**0.4**	—	—	—	**0.8**	—	—	—	—	—	—	—	**1.0**	—	**0.0**
	**Trans fat**	**Median**	—	—	—	—	—	—	—	**0.0**	—	—	—	**0.0**	—	—	—	—	—	—	—	**0.0**	—	**0.0**
	**per 100**	**IQR**	—	—	—	—	—	—	—	**0.0**	—	—	—	**0.0**	—	—	—	—	—	—	—	**0.0**	—	**0.0**
	**Total sugars**	**Median**	—	—	—	—	—	—	—	**1.2**	—	—	—	**0.0**	—	—	—	—	—	—	—	**0.7**	—	**2.2**
	**per 100**	**IQR**	—	—	—	—	—	—	—	**0.6**	—	—	—	**0.3**	—	—	—	—	—	—	—	**2.8**	—	**2.0**
	**Free sugars**	**Median**	—	—	—	—	—	—	—	**0.0**	—	—	—	**0.0**	—	—	—	—	—	—	—	**0.0**	—	**1.2**
	**per 100**	**IQR**	—	—	—	—	—	—	—	**0.3**	—	—	—	**0.0**	—	—	—	—	—	—	—	**1.2**	—	**2.0**
**Salads (Q)**	**Number of products**		**0**	**0**	**0**	**0**	**0**	**0**	**0**	**0**	**0**	**0**	**1**	**8**	**0**	**0**	**0**	**0**	**0**	**0**	**0**	**16**	**0**	**0**
	**Health Star**	**Mean**	—	—	—	—	—	—	—	—	—	—	**3.0**	**3.5**	—	—	—	—	—	—	—	**3.7**	—	—
	**Rating**	**SD**	—	—	—	—	—	—	—	—	—	—	**N/A**	**0.7**	—	—	—	—	—	—	—	**0.6**	—	—
	**Calories per**	**Median**	—	—	—	—	—	—	—	—	—	—	**191.8**	**141.9**	—	—	—	—	—	—	—	**145.0**	—	—
	**100 g/mL**	**IQR**	—	—	—	—	—	—	—	—	—	—	**0.0**	**16.2**	—	—	—	—	—	—	—	**45.5**	—	—
	**Sodium per**	**Median**	—	—	—	—	—	—	—	—	—	—	**520.5**	**263.8**	—	—	—	—	—	—	—	**257.1**	—	—
	**100 g/mL**	**IQR**	—	—	—	—	—	—	—	—	—	—	**0.0**	**61.4**	—	—	—	—	—	—	—	**132.9**	—	—
	**Saturated**	**Median**	—	—	—	—	—	—	—	—	—	—	**0.7**	**1.3**	—	—	—	—	—	—	—	**1.0**	—	—
	**fat per 100**	**IQR**	—	—	—	—	—	—	—	—	—	—	**0.0**	**1.0**	—	—	—	—	—	—	—	**1.1**	—	—
	**Trans fat**	**Median**	—	—	—	—	—	—	—	—	—	—	**0.0**	**0.0**	—	—	—	—	—	—	—	**0.0**	—	—
	**per 100**	**IQR**	—	—	—	—	—	—	—	—	—	—	**0.0**	**0.0**	—	—	—	—	—	—	—	**0.0**	—	—
	**Total sugars**	**Median**	—	—	—	—	—	—	—	—	—	—	**3.4**	**4.7**	—	—	—	—	—	—	—	**5.9**	—	—
	**per 100**	**IQR**	—	—	—	—	—	—	—	—	—	—	**0.0**	**5.0**	—	—	—	—	—	—	—	**7.0**	—	—
	**Free sugars**	**Median**	—	—	—	—	—	—	—	—	—	—	**0.0**	**3.9**	—	—	—	—	—	—	—	**4.9**	—	—
	**per 100**	**IQR**	—	—	—	—	—	—	—	—	—	—	**0.0**	**5.1**	—	—	—	—	—	—	—	**7.1**	—	—
**Sauces, dips, gravies and condiments (R)**	**Number of products**		**0**	**0**	**15**	**6**	**0**	**0**	**0**	**10**	**0**	**0**	**66**	**221**	**1**	**0**	**3**	**2**	**1**	**13**	**5**	**82**	**0**	**25**
	**Health Star**	**Mean**	—	—	**3.0**	**2.8**	—	—	—	**3.0**	—	—	**2.1**	**2.7**	**1.0**	—	**2.5**	**2.5**	**3.0**	**3.2**	**1.5**	**2.6**	—	**2.8**
	**Rating**	**SD**	—	—	**0.2**	**1.2**	—	—	—	**0.2**	—	—	**0.7**	**0.8**	**N/A**	—	**0.9**	**0.0**	**N/A**	**0.7**	**0.0**	**0.8**	—	**0.7**
	**Calories per**	**Median**	—	—	**41.7**	**140.0**	—	—	—	**50.0**	—	—	**140.6**	**116.7**	**300.0**	—	**66.7**	**200.0**	**133.3**	**181.8**	**193.5**	**133.3**	—	**83.3**
	**100 g/mL**	**IQR**	—	—	**16.3**	**165.0**	—	—	—	**10.0**	—	—	**100.0**	**136.0**	**0.0**	—	**50.0**	**0.0**	**0.0**	**216.7**	**32.3**	**148.5**	—	**77.3**
	**Sodium per**	**Median**	—	—	**466.7**	**445.0**	—	—	—	**880.0**	—	—	**800.0**	**500.0**	**1700.0**	—	**9200.0**	**16.7**	**233.3**	**625.0**	**548.4**	**566.7**	—	**566.7**
	**100 g/mL**	**IQR**	—	—	**258.3**	**180.0**	—	—	—	**245.7**	—	—	**569.8**	**556.0**	**0.0**	—	**4350.0**	**0.0**	**0.0**	**402.4**	**0.0**	**766.5**	—	**150.0**
	**Saturated**	**Median**	—	—	**0.0**	**3.0**	—	—	—	**0.0**	—	—	**0.0**	**0.0**	**0.0**	—	**0.0**	**0.0**	**6.7**	**1.6**	**9.7**	**0.0**	—	**0.3**
	**fat per 100**	**IQR**	—	—	**0.6**	**4.1**	—	—	—	**0.0**	—	—	**1.7**	**1.3**	**0.0**	—	**0.0**	**0.0**	**0.0**	**3.6**	**1.6**	**0.8**	—	**1.3**
	**Trans fat**	**Median**	—	—	**0.0**	**0.1**	—	—	—	**0.0**	—	—	**0.0**	**0.0**	**0.0**	—	**0.0**	**0.0**	**0.0**	**0.0**	**0.0**	**0.0**	—	**0.0**
	**per 100**	**IQR**	—	—	**0.0**	**0.1**	—	—	—	**0.0**	—	—	**0.0**	**0.0**	**0.0**	—	**0.0**	**0.0**	**0.0**	**0.0**	**0.0**	**0.0**	—	**0.0**
	**Total sugars**	**Median**	—	—	**1.6**	**4.0**	—	—	—	**6.0**	—	—	**13.3**	**6.4**	**40.0**	—	**6.7**	**40.0**	**6.7**	**0.0**	**3.2**	**5.0**	—	**3.3**
	**per 100**	**IQR**	—	—	**6.7**	**1.5**	—	—	—	**2.5**	—	—	**23.3**	**20.1**	**0.0**	—	**16.7**	**0.0**	**0.0**	**3.3**	**3.2**	**24.6**	—	**5.0**
	**Free sugars**	**Median**	—	—	**0.0**	**2.0**	—	—	—	**2.2**	—	—	**13.3**	**3.3**	**40.0**	—	**6.7**	**40.0**	**4.0**	**0.0**	**0.6**	**2.1**	—	**1.0**
	**per 100**	**IQR**	—	—	**1.0**	**1.6**	—	—	—	**3.2**	—	—	**25.2**	**20.0**	**0.0**	—	**14.3**	**0.0**	**0.0**	**0.0**	**3.9**	**21.0**	—	**4.3**
**Snacks (S)**	**Number of products**		**0**	**0**	**0**	**0**	**0**	**0**	**0**	**3**	**0**	**9**	**6**	**103**	**8**	**1**	**0**	**0**	**0**	**92**	**0**	**75**	**0**	**0**
	**Health Star**	**Mean**	—	—	—	—	—	—	—	**2.8**	—	**2.0**	**0.9**	**2.8**	**0.9**	**2.0**	—	—	—	**2.5**	—	**2.9**	—	—
	**Rating**	**SD**	—	—	—	—	—	—	—	**2.0**	—	**0.5**	**0.7**	**1.0**	**0.4**	**N/A**	—	—	—	**0.6**	—	**1.0**	—	—
	**Calories per**	**Median**	—	—	—	—	—	—	—	**488.4**	—	**500.0**	**447.3**	**520.0**	**300.0**	**440.0**	—	—	—	**520.0**	—	**540.0**	—	—
	**100 g/mL**	**IQR**	—	—	—	—	—	—	—	**15.8**	—	**35.7**	**18.5**	**80.0**	**50.0**	**0.0**	—	—	—	**40.0**	—	**110.0**	—	—
	**Sodium per**	**Median**	—	—	—	—	—	—	—	**279.1**	—	**607.1**	**727.3**	**500.0**	**1180.0**	**1060.0**	—	—	—	**680.0**	—	**480.0**	—	—
	**100 g/mL**	**IQR**	—	—	—	—	—	—	—	**390.5**	—	**142.9**	**226.8**	**430.0**	**770.0**	**0.0**	—	—	—	**420.0**	—	**348.5**	—	—
	**Saturated**	**Median**	—	—	—	—	—	—	—	**2.3**	—	**7.1**	**7.6**	**3.0**	**8.5**	**3.0**	—	—	—	**3.0**	—	**4.0**	—	—

			A. Lassonde	Agropur	Campbell Soup	Canada Bread	Canada Dry Mott's	Coca-Cola	Danone	General Mills	George Weston	Kellogg	Kraft Heinz	Loblaw	Maple Leaf Foods	Mondelēz	Nestlé	Ocean Spray	Parmalat	PepsiCo	Saputo	Sobeys	Sun-Rype	Unilever
	fat per 100	IQR	—	—	—	—	—	—	—	11.8	—	1.8	8.5	3.0	2.5	0.0	—	—	—	1.0	—	5.0	—	—
	Trans fat	Median	—	—	—	—	—	—	—	0.0	—	0.0	0.0	0.0	0.2	0.0	—	—	—	0.0	—	0.0	—	—
	per 100	IQR	—	—	—	—	—	—	—	0.0	—	0.0	0.0	0.0	0.3	0.0	—	—	—	0.0	—	0.1	—	—
	Total sugars	Median	—	—	—	—	—	—	—	4.7	—	3.6	31.8	4.0	0.0	6.0	—	—	—	2.0	—	4.0	—	—
	per 100	IQR	—	—	—	—	—	—	—	1.5	—	0.0	38.8	4.0	0.9	0.0	—	—	—	2.1	—	4.0	—	—
	Free sugars	Median	—	—	—	—	—	—	—	3.7	—	2.5	31.4	1.0	0.0	4.8	—	—	—	1.0	—	0.0	—	—
	per 100	IQR	—	—	—	—	—	—	—	1.7	—	0.0	39.9	4.0	0.9	0.0	—	—	—	3.0	—	3.0	—	—
Soups (T)	Number of products		0	0	127	0	0	0	0	0	0	0	1	54	0	0	0	0	0	0	0	32	0	45
	Health Star	Mean	—	—	3.5	—	—	—	—	—	—	—	3.5	3.3	—	—	—	—	—	—	—	3.4	—	3.2
	Rating	SD	—	—	0.2	—	—	—	—	—	—	—	N/A	0.3	—	—	—	—	—	—	—	0.3	—	0.3
	Calories per	Median	—	—	44.0	—	—	—	—	—	—	—	28.0	40.0	—	—	—	—	—	—	—	36.0	—	10.0
	100 g/mL	IQR	—	—	29.1	—	—	—	—	—	—	—	0.0	55.2	—	—	—	—	—	—	—	59.0	—	22.9
	Sodium per	Median	—	—	256.0	—	—	—	—	—	—	—	320.0	277.0	—	—	—	—	—	—	—	234.0	—	288.0
	100 g/mL	IQR	—	—	80.9	—	—	—	—	—	—	—	0.0	99.9	—	—	—	—	—	—	—	29.0	—	126.0
	Saturated	Median	—	—	0.2	—	—	—	—	—	—	—	0.0	0.2	—	—	—	—	—	—	—	0.2	—	0.0
	fat per 100	IQR	—	—	0.4	—	—	—	—	—	—	—	0.0	0.6	—	—	—	—	—	—	—	0.4	—	0.2
	Trans fat	Median	—	—	0.0	—	—	—	—	—	—	—	0.0	0.0	—	—	—	—	—	—	—	0.0	—	0.0
	per 100	IQR	—	—	0.0	—	—	—	—	—	—	—	0.0	0.0	—	—	—	—	—	—	—	0.0	—	0.0
	Total sugars	Median	—	—	1.2	—	—	—	—	—	—	—	0.4	0.4	—	—	—	—	—	—	—	0.4	—	0.4
	per 100	IQR	—	—	1.3	—	—	—	—	—	—	—	0.0	1.5	—	—	—	—	—	—	—	0.9	—	0.8
	Free sugars	Median	—	—	0.5	—	—	—	—	—	—	—	0.2	0.3	—	—	—	—	—	—	—	0.0	—	0.0
	per 100	IQR	—	—	1.4	—	—	—	—	—	—	—	0.0	0.8	—	—	—	—	—	—	—	0.8	—	0.6
Sugars and sweets (U)	Number of products		0	0	0	0	2	0	0	11	0	0	12	116	0	33	28	0	0	3	0	38	13	1
	Health Star	Mean	—	—	—	—	1.3	—	—	1.6	—	—	0.7	1.6	—	0.9	0.7	—	—	2.0	—	1.7	2.5	0.5
	Rating	SD	—	—	—	—	0.4	—	—	0.2	—	—	0.5	0.7	—	0.5	0.4	—	—	0.5	—	0.6	0.0	N/A
	Calories per	Median	—	—	—	—	333.3	—	—	363.6	—	—	513.9	333.3	—	500.0	512.7	—	—	355.6	—	333.3	324.3	700.0
	100 g/mL	IQR	—	—	—	—	0.0	—	—	6.5	—	—	44.5	216.4	—	161.6	112.6	—	—	111.1	—	31.8	32.8	0.0
	Sodium per	Median	—	—	—	—	66.7	—	—	173.9	—	—	86.3	30.6	—	69.8	84.3	—	—	33.3	—	18.3	35.7	200.0
	100 g/mL	IQR	—	—	—	—	0.0	—	—	100.6	—	—	142.1	66.9	—	33.3	42.9	—	—	22.2	—	46.9	73.8	0.0
	Saturated	Median	—	—	—	—	0.0	—	—	1.7	—	—	16.5	0.0	—	14.0	15.0	—	—	0.0	—	0.0	0.0	20.0
	fat per 100	IQR	—	—	—	—	0.0	—	—	1.9	—	—	5.1	13.5	—	16.7	9.2	—	—	0.0	—	0.0	0.0	0.0
	Trans fat	Median	—	—	—	—	0.0	—	—	1.3	—	—	0.1	0.0	—	0.2	0.0	—	—	0.0	—	0.0	0.0	0.0
	per 100	IQR	—	—	—	—	0.0	—	—	1.9	—	—	0.2	0.0	—	0.2	0.0	—	—	0.0	—	0.0	0.0	0.0
	Total sugars	Median	—	—	—	—	76.7	—	—	50.0	—	—	53.9	53.3	—	54.0	56.2	—	—	53.3	—	60.0	78.4	50.0
	per 100	IQR	—	—	—	—	10.0	—	—	6.8	—	—	11.9	21.0	—	10.5	13.3	—	—	11.1	—	14.2	2.9	0.0
	Free sugars	Median	—	—	—	—	76.7	—	—	50.0	—	—	53.9	53.3	—	54.0	56.2	—	—	53.3	—	60.0	32.2	50.0
	per 100	IQR	—	—	—	—	10.0	—	—	6.8	—	—	11.9	24.8	—	10.5	13.3	—	—	11.1	—	14.2	9.3	0.0
Confectionary (U1, U3, U4, U10, U11)	Number of products		0	0	0	0	0	0	0	11	0	0	12	48	0	33	23	0	0	0	0	17	13	0
	Health Star	Mean	—	—	—	—	—	—	—	1.6	—	—	0.7	1.2	—	0.9	0.6	—	—	—	—	1.5	2.5	—
	Rating	SD	—	—	—	—	—	—	—	0.2	—	—	0.5	0.7	—	0.5	0.3	—	—	—	—	0.4	0.0	—
	Calories per	Median	—	—	—	—	—	—	—	363.6	—	—	513.9	533.3	—	500.0	520.0	—	—	—	—	325.0	324.3	—
	100 g/mL	IQR	—	—	—	—	—	—	—	6.5	—	—	44.5	190.0	—	161.6	49.0	—	—	—	—	67.5	32.8	—
	Sodium per	Median	—	—	—	—	—	—	—	173.9	—	—	86.3	62.5	—	69.8	83.3	—	—	—	—	25.0	35.7	—
	100 g/mL	IQR	—	—	—	—	—	—	—	100.6	—	—	142.1	88.1	—	33.3	23.0	—	—	—	—	47.0	73.8	—
	Saturated fat	Median	—	—	—	—	—	—	—	1.7	—	—	16.5	15.0	—	14.0	15.6	—	—	—	—	0.0	0.0	—
	per 100 g/mL	IQR	—	—	—	—	—	—	—	1.9	—	—	5.1	17.5	—	16.7	3.3	—	—	—	—	0.0	0.0	—
	Trans fat per	Median	—	—	—	—	—	—	—	1.3	—	—	0.1	0.0	—	0.2	0.0	—	—	—	—	0.0	0.0	—
	100 g/mL (g)	IQR	—	—	—	—	—	—	—	1.9	—	—	0.2	0.1	—	0.2	0.1	—	—	—	—	0.0	0.0	—
	Total sugars	Median	—	—	—	—	—	—	—	50.0	—	—	53.9	48.8	—	54.0	52.5	—	—	—	—	60.7	78.4	—
	per 100 g/mL	IQR	—	—	—	—	—	—	—	6.8	—	—	11.9	18.2	—	10.5	10.3	—	—	—	—	17.5	2.9	—
	Free sugars	Median	—	—	—	—	—	—	—	50.0	—	—	53.9	47.5	—	54.0	52.5	—	—	—	—	60.7	32.2	—
	per 100 g/mL	IQR	—	—	—	—	—	—	—	6.8	—	—	11.9	18.0	—	10.5	10.3	—	—	—	—	17.5	9.3	—
Sugars and syrups (U8, U9, U12, U14, U15)	Number of products		0	0	0	0	2	0	0	0	0	0	0	68	0	0	5	0	0	3	0	21	0	1
	Health Star	Mean	—	—	—	—	1.3	—	—	—	—	—	—	1.9	—	—	1.3	—	—	2.0	—	1.8	—	0.5
	Rating	SD	—	—	—	—	0.4	—	—	—	—	—	—	0.6	—	—	0.3	—	—	0.5	—	0.6	—	N/A
	Calories per	Median	—	—	—	—	333.3	—	—	—	—	—	—	333.3	—	—	350.0	—	—	355.6	—	333.3	—	700.0
	100 g/mL	IQR	—	—	—	—	0.0	—	—	—	—	—	—	100.0	—	—	0.0	—	—	111.1	—	16.7	—	0.0
	Sodium per	Median	—	—	—	—	66.7	—	—	—	—	—	—	0.0	—	—	125.0	—	—	33.3	—	0.0	—	200.0
	100 g/mL	IQR	—	—	—	—	0.0	—	—	—	—	—	—	33.3	—	—	0.0	—	—	22.2	—	8.3	—	0.0
	Saturated fat	Median	—	—	—	—	0.0	—	—	—	—	—	—	0.0	—	—	0.0	—	—	0.0	—	0.0	—	20.0
	per 100 g/mL	IQR	—	—	—	—	0.0	—	—	—	—	—	—	0.0	—	—	0.0	—	—	0.0	—	0.0	—	0.0
	Trans fat per	Median	—	—	—	—	0.0	—	—	—	—	—	—	0.0	—	—	0.0	—	—	0.0	—	0.0	—	0.0
	100 g/mL (g)	IQR	—	—	—	—	0.0	—	—	—	—	—	—	0.0	—	—	0.0	—	—	0.0	—	0.0	—	0.0
	Total sugars	Median	—	—	—	—	76.7	—	—	—	—	—	—	60.0	—	—	60.0	—	—	53.3	—	60.0	—	50.0
	per 100 g/mL	IQR	—	—	—	—	10.0	—	—	—	—	—	—	23.3	—	—	20.0	—	—	11.1	—	21.7	—	0.0
	Free sugars	Median	—	—	—	—	76.7	—	—	—	—	—	—	60.0	—	—	60.0	—	—	53.3	—	60.0	—	50.0
	per 100 g/mL	IQR	—	—	—	—	10.0	—	—	—	—	—	—	23.3	—	—	20.0	—	—	11.1	—	21.7	—	0.0
Vegetables (V)	Number of products		2	0	8	0	5	0	0	33	0	0	1	145	0	0	0	0	0	0	0	92	0	1
	Health Star	Mean	1.8	—	3.4	—	1.7	—	—	4.2	—	—	5.0	3.7	—	—	—	—	—	—	—	4.0	—	3.5
	Rating	SD	0.4	—	1.4	—	0.6	—	—	0.5	—	—	N/A	1.0	—	—	—	—	—	—	—	1.0	—	N/A
	Calories per	Median	23.7	—	26.0	—	24.0	—	—	40.0	—	—	20.0	35.3	—	—	—	—	—	—	—	29.4	—	16.7
	100 g/mL	IQR	0.3	—	18.2	—	0.0	—	—	37.0	—	—	0.0	80.0	—	—	—	—	—	—	—	62.4	—	0.0
	Sodium per	Median	183.7	—	76.5	—	248.0	—	—	150.0	—	—	192.0	233.3	—	—	—	—	—	—	—	180.0	—	766.7

			A. Lassonde	Agropur	Campbell Soup	Canada Bread	Canada Dry Mott's	Coca-Cola	Danone	General Mills	George Weston	Kellogg	Kraft Heinz	Loblaw	Maple Leaf Foods	Mondelēz	Nestlé	Ocean Spray	Parmalat	PepsiCo	Saputo	Sobeys	Sun-Rype	Unilever
	**100 g/mL**	**IQR**	**0.3**	—	**37.9**	—	**76.0**	—	—	**186.0**	—	—	**0.0**	**729.4**	—	—	—	—	—	—	—	**352.4**	—	**0.0**
	**Saturated fat per 100**	**Median**	**0.0**	—	**0.0**	—	**0.0**	—	—	**0.0**	—	—	**0.0**	**0.0**	—	—	—	—	—	—	—	**0.0**	—	**0.0**
		**IQR**	**0.0**	—	**0.0**	—	**0.0**	—	—	**0.0**	—	—	**0.0**	**0.0**	—	—	—	—	—	—	—	**0.0**	—	**0.0**
	**Trans fat per 100**	**Median**	**0.0**	—	**0.0**	—	**0.0**	—	—	**0.0**	—	—	**0.0**	**0.0**	—	—	—	—	—	—	—	**0.0**	—	**0.0**
		**IQR**	**0.0**	—	**0.0**	—	**0.0**	—	—	**0.0**	—	—	**0.0**	**0.0**	—	—	—	—	—	—	—	**0.0**	—	**0.0**
	**Total sugars per 100**	**Median**	**2.4**	—	**4.8**	—	**4.0**	—	—	**3.2**	—	—	**2.4**	**1.6**	—	—	—	—	—	—	—	**2.0**	—	**0.0**
		**IQR**	**0.0**	—	**2.0**	—	**0.4**	—	—	**2.0**	—	—	**0.0**	**3.5**	—	—	—	—	—	—	—	**3.2**	—	**0.0**
	**Free sugars per 100**	**Median**	**0.0**	—	**0.0**	—	**1.5**	—	—	**0.0**	—	—	**0.0**	**0.0**	—	—	—	—	—	—	—	**0.0**	—	**0.0**
		**IQR**	**0.0**	—	**0.9**	—	**0.4**	—	—	**2.5**	—	—	**0.0**	**0.0**	—	—	—	—	—	—	—	**0.0**	—	**0.0**
Vegetable juice and vegetable drink (V7)	Number of products		2	0	5	0	5	0	0	0	0	0	1	7	0	0	0	0	0	0	0	5	0	0
	Health Star Rating	Mean	1.8	—	2.7	—	1.7	—	—	—	—	—	5.0	2.5	—	—	—	—	—	—	—	2.3	—	—
		SD	0.4	—	1.4	—	0.6	—	—	—	—	—	N/A	1.8	—	—	—	—	—	—	—	1.5	—	—
	Calories per 100 g/mL	Median	23.7	—	24.0	—	24.0	—	—	—	—	—	20.0	24.0	—	—	—	—	—	—	—	24.0	—	—
		IQR	0.3	—	4.0	—	0.0	—	—	—	—	—	0.0	5.0	—	—	—	—	—	—	—	4.0	—	—
	Sodium per 100 g/mL	Median	183.7	—	68.0	—	248.0	—	—	—	—	—	192.0	192.0	—	—	—	—	—	—	—	240.0	—	—
		IQR	0.3	—	126.0	—	76.0	—	—	—	—	—	0.0	112.0	—	—	—	—	—	—	—	68.0	—	—
	Saturated fat per 100 g/mL	Median	0.0	—	0.0	—	0.0	—	—	—	—	—	0.0	0.0	—	—	—	—	—	—	—	0.0	—	—
		IQR	0.0	—	0.0	—	0.0	—	—	—	—	—	0.0	0.0	—	—	—	—	—	—	—	0.0	—	—
	Trans fat per 100 g/mL (g)	Median	0.0	—	0.0	—	0.0	—	—	—	—	—	0.0	0.0	—	—	—	—	—	—	—	0.0	—	—
		IQR	0.0	—	0.0	—	0.0	—	—	—	—	—	0.0	0.0	—	—	—	—	—	—	—	0.0	—	—
	Total sugars per 100 g/mL	Median	2.4	—	4.0	—	4.0	—	—	—	—	—	2.4	2.8	—	—	—	—	—	—	—	3.2	—	—
		IQR	0.0	—	0.8	—	0.4	—	—	—	—	—	0.0	0.4	—	—	—	—	—	—	—	0.8	—	—
	Free sugars per 100 g/mL	Median	0.0	—	0.6	—	1.5	—	—	—	—	—	0.0	0.0	—	—	—	—	—	—	—	0.6	—	—
		IQR	0.0	—	1.5	—	0.4	—	—	—	—	—	0.0	0.4	—	—	—	—	—	—	—	0.6	—	—
Vegetables with sauce, pastes (V2, V9, V10, V11, V12)	Number of products		0	0	0	0	0	0	0	6	0	0	0	35	0	0	0	0	0	0	0	13	0	1
	Health Star Rating	Mean	—	—	—	—	—	—	—	3.8	—	—	—	3.3	—	—	—	—	—	—	—	3.1	—	3.5
		SD	—	—	—	—	—	—	—	0.3	—	—	—	0.7	—	—	—	—	—	—	—	0.7	—	N/A
	Calories per 100 g/mL	Median	—	—	—	—	—	—	—	78.1	—	—	—	53.2	—	—	—	—	—	—	—	83.3	—	16.7
		IQR	—	—	—	—	—	—	—	36.6	—	—	—	78.9	—	—	—	—	—	—	—	83.3	—	0.0
	Sodium per 100 g/mL	Median	—	—	—	—	—	—	—	231.0	—	—	—	571.4	—	—	—	—	—	—	—	500.0	—	766.7
		IQR	—	—	—	—	—	—	—	40.0	—	—	—	559.5	—	—	—	—	—	—	—	400.0	—	0.0
	Saturated fat per 100 g/mL	Median	—	—	—	—	—	—	—	0.7	—	—	—	0.0	—	—	—	—	—	—	—	0.0	—	0.0
		IQR	—	—	—	—	—	—	—	0.7	—	—	—	0.0	—	—	—	—	—	—	—	0.0	—	0.0
	Trans fat per 100 g/mL (g)	Median	—	—	—	—	—	—	—	0.0	—	—	—	0.0	—	—	—	—	—	—	—	0.0	—	0.0
		IQR	—	—	—	—	—	—	—	0.0	—	—	—	0.0	—	—	—	—	—	—	—	0.0	—	0.0
	Total sugars per 100 g/mL	Median	—	—	—	—	—	—	—	3.1	—	—	—	5.0	—	—	—	—	—	—	—	10.0	—	0.0
		IQR	—	—	—	—	—	—	—	1.4	—	—	—	16.7	—	—	—	—	—	—	—	20.0	—	0.0
	Free sugars per 100 g/mL	Median	—	—	—	—	—	—	—	1.4	—	—	—	0.0	—	—	—	—	—	—	—	0.0	—	0.0
		IQR	—	—	—	—	—	—	—	1.7	—	—	—	16.7	—	—	—	—	—	—	—	20.0	—	0.0
Vegetables without sauce (V1, V3, V4, V5, V8)	Number of products		0	0	3	0	0	0	0	27	0	0	0	103	0	0	0	0	0	0	0	74	0	0
	Health Star Rating	Mean	—	—	4.5	—	—	—	—	4.3	—	—	—	3.9	—	—	—	—	—	—	—	4.2	—	—
		SD	—	—	0.0	—	—	—	—	0.5	—	—	—	0.8	—	—	—	—	—	—	—	0.8	—	—
	Calories per 100 g/mL	Median	—	—	41.2	—	—	—	—	36.0	—	—	—	35.3	—	—	—	—	—	—	—	29.8	—	—
		IQR	—	—	0.0	—	—	—	—	24.0	—	—	—	109.2	—	—	—	—	—	—	—	38.7	—	—
	Sodium per 100 g/mL	Median	—	—	76.5	—	—	—	—	88.0	—	—	—	184.0	—	—	—	—	—	—	—	124.0	—	—
		IQR	—	—	0.0	—	—	—	—	167.5	—	—	—	820.6	—	—	—	—	—	—	—	238.4	—	—
	Saturated fat per 100 g/mL	Median	—	—	0.0	—	—	—	—	0.0	—	—	—	0.0	—	—	—	—	—	—	—	0.0	—	—
		IQR	—	—	0.0	—	—	—	—	0.0	—	—	—	0.2	—	—	—	—	—	—	—	0.0	—	—
	Trans fat per 100 g/mL (g)	Median	—	—	0.0	—	—	—	—	0.0	—	—	—	0.0	—	—	—	—	—	—	—	0.0	—	—
		IQR	—	—	0.0	—	—	—	—	0.0	—	—	—	0.0	—	—	—	—	—	—	—	0.0	—	—
	Total sugars per 100 g/mL	Median	—	—	5.9	—	—	—	—	3.2	—	—	—	1.2	—	—	—	—	—	—	—	1.6	—	—
		IQR	—	—	0.0	—	—	—	—	2.0	—	—	—	3.2	—	—	—	—	—	—	—	2.4	—	—
	Free sugars per 100 g/mL	Median	—	—	0.0	—	—	—	—	0.0	—	—	—	0.0	—	—	—	—	—	—	—	0.0	—	—
		IQR	—	—	0.0	—	—	—	—	2.0	—	—	—	0.0	—	—	—	—	—	—	—	0.0	—	—

[1]Food categories are based on those defined in Health Canada's Table of Reference Amounts for Foods (TRA), listed in brackets. Values at the TRA major food category level are shown in bold. TRA minor food categories that were combined are indicated in brackets. If none of the sampled products fell into that food category, it was omitted from the table.

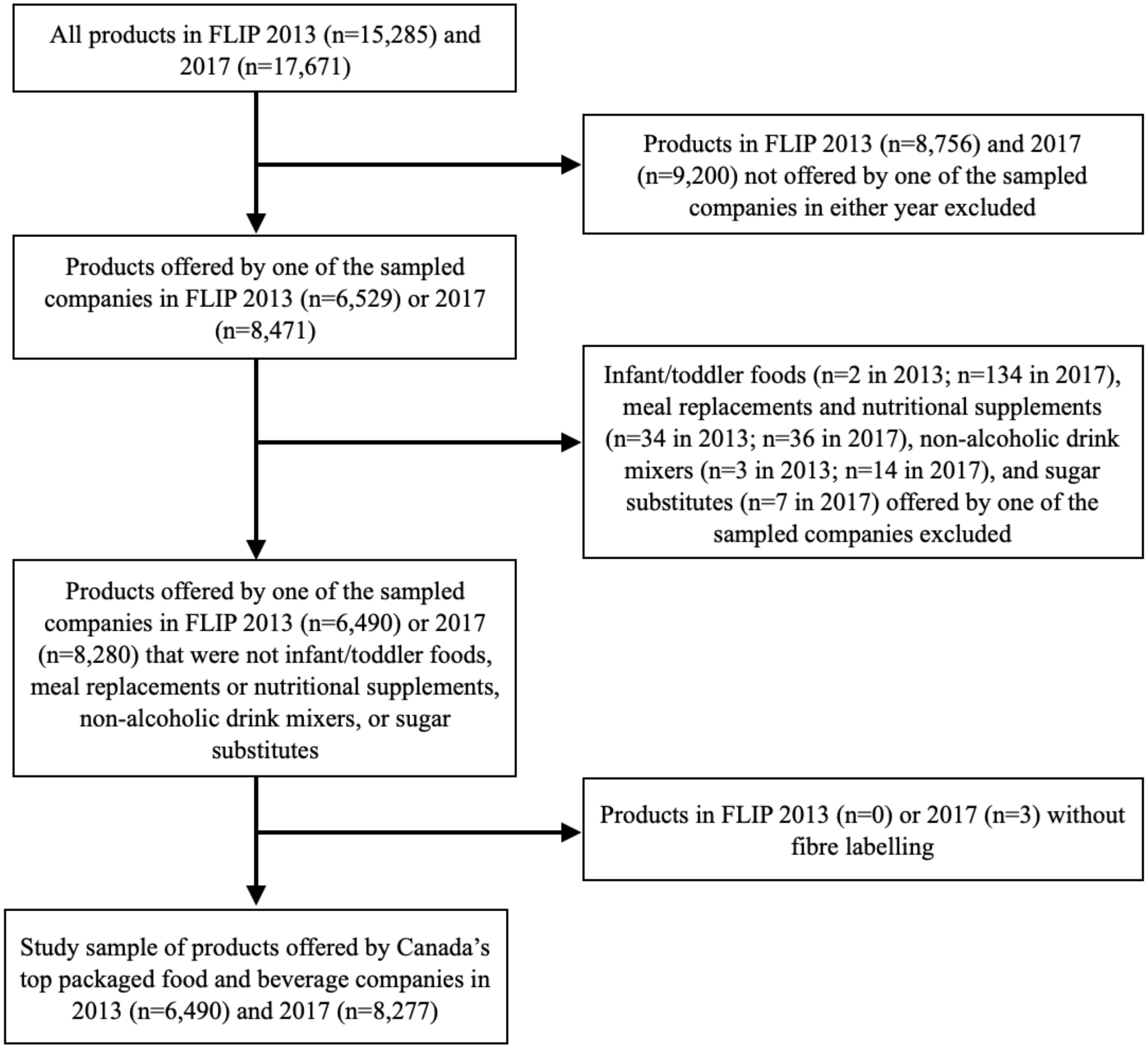

Supplementary Figure 1. The approach used to derive the sample of products examined in this study.
