# Supplementary material for: The relationship between voluntary product (re) formulation commitments and changes in the nutritional quality of products offered by the top packaged food and beverage companies in Canada from 2013 to 2017
Source: BMC Public Health. 2022 Feb 10;22:271. doi: 10.1186/s12889-022-12683-2 (PMC8832833; doi:10.1186/s12889-022-12683-2)
Supplement: Supplementary file 2 — Additional file 2:. Evaluating the Validity, Internal Consistency and Inter-rater Reliability of the Food Company Reformulation (FCR) Scoring Tool. [file 12889_2022_12683_MOESM2_ESM.pdf]

Evaluating the Validity, Internal Consistency and Inter-rater Reliability of the Food Company Reformulation (FCR) Scoring Tool*

This document accompanies the following manuscript:

Vergeer L, Ahmed M, Vanderlee L, Mulligan C, Weippert M, Franco-Arellano B, Dickinson K, Bernstein JT, Labonté MÈ, L'Abbé MR. The relationship between voluntary product (re)formulation commitments and changes in the nutritional quality of products offered by the top packaged food and beverage companies in Canada from 2013-2017.

The development and application of the FCR tool is described in detail here:

Vergeer L, Vanderlee L, Sacks G, Robinson E, Mackay S, Young L, Mulligan C, L'Abbé MR. The Development and Application of a Tool for Quantifying the Strength of Voluntary Actions and Commitments of Major Canadian Food Companies to Improve the Nutritional Quality of Their Products. Curr Dev Nutr. 2020 Sep 22;4(10):nzaa151. doi: 10.1093/cdn/nzaa151.

*This document and any analyses within it were completed by Dr. Laura Vergeer, with guidance and feedback provided by Drs. Lana Vanderlee, Mary L'Abbé and Valerie Tarasuk.

In developing and applying a new scoring tool or methodology, it is important to establish the validity, internal consistency and reliability of the tool. Validity is a measure of the extent that the scores derived from a tool represent the intended concept. Internal consistency measures the consistency of scores or responses across the items of a multiple-item measure or tool; since all items should be reflective of the same underlying construct, scores on individual items should be correlated with one another[1]. Reliability refers to the consistency of the items or measures within the tool and between raters[1]. This document describes our approach to assessing the validity and reliability of the Food Company Reformulation (FCR) scoring tool.

Unweighted versus weighted FCR tool scores

The FCR total score is derived from companies' nutrient/component sub-scores (energy/portion sizes, sodium, saturated fat, *trans* fat, and sugars for packaged food companies; and only energy/portion sizes and sugars for beverage companies), and their scores for the "additional" indicators (out of 5), which assess more general product (re)formulation policies that are not nutrient-specific (e.g., support of government or WHO product formulation policies, use of a government-endorsed NP model, participating in industry initiatives). The sub-score denominators differ by nutrient/component, with scores out of 32 for sodium, out of 30 for energy/portion sizes and saturated fat and sugars, and out of 28 for *trans* fat. The original FCR scores were unweighted, meaning that some nutrient/component scores inadvertently contributed more to a company's FCR total score, simply based on the denominator for that nutrient (with sodium contributing the most and *trans* fat contributing the least). We therefore decided to examine the effect of equally weighting all nutrients/components on companies' FCR scores. Each nutrient/component was weighted the same, with the additional indicators assigned a lower weight, based on their weighting within the original total scores (3.3% of the total score for packaged food companies and 7.8% for beverage companies). Weighted total scores that excluded the additional indicators were also calculated to examine how this might change the ranking of companies, as the weighting of the additional indicators was somewhat arbitrary.

Within each nutrient/component of the FCR tool, there are 6 domains (reduction/commitment, timebound, breadth, magnitude of reduction, national/global applicability, and transparency). The total number of indicators within a domain ranges from 2 to 8. Consequently, domains with

a larger number of indicators (e.g., magnitude of reduction, breadth) may inadvertently exert greater influence on companies' nutrient/component scores than domains with fewer indicators (e.g., reduction/commitment, timebound, national/global applicability). Similar to as described above, we therefore calculated new 'weighted' scores, such that each of the 6 domains in the nutrient sections of the tool were weighted the same. Weighted scores were determined by calculating each company's score as a proportion of the denominator for that domain (i.e., resulting in a score out of 1) and summed to generate an absolute weighted score out of 6.

Face and content validity

Face validity is the extent to which a tool or method appears at first glance or "on its face" to measure the intended construct[1]. Face validity of the FCR scoring tool was established by consulting with 6 researchers with expertise in private sector nutrition policy, and with extensive knowledge of recommendations concerning product (re)formulation (according to governments, the WHO and other public health authorities). The tool was initially developed by the first author (Vergeer) based on the BIA-Obesity and ATNI tools for evaluating food companies' nutrition policies and commitments, and a review of relevant peer-reviewed articles, grey literature and WHO reports. Experts provided feedback on the tool in several iterations from 2019-2020, and it was modified based on their comments.

Content validity is similar to face validity in that it assesses the degree to which a tool or method captures the complete range of meaning for the concept being measured; however, it is typically evaluated more formally, such as by assessing the alignment of the tool with current scientific literature, or with an accepted standard (when available)[2,3]. Development of the FCR tool in consultation with experts and after a review of existing methodologies and recommendations concerning product (re)formulation policy ensured that the final version of the tool included key nutrients/components of public health concern (addressed in government and WHO recommendations)[4], and criteria commonly used to develop and/or evaluate food companies' commitments related to product (re)formulation (e.g., baseline and target years, breadth across brands and food categories, magnitude of reduction targeted)[5-8], thereby establishing content validity. Additionally, the binary indicators in the FCR tool were developed based on SMART

criteria, which are commonly used and recommended by the WHO, FAO and other global public health authorities in setting nutrition targets[9-15].

Construct/convergent validity

Without a gold standard against which to compare the tool or methodology in question (i.e., concurrent/criterion validity), construct/convergent validity can be evaluated by examining the alignment of the tool with existing methods[16]. Convergent validity is a sub-type of construct validity and can be assessed using correlation coefficients to determine whether a test of a concept is strongly correlated with other tests intended to measure similar concepts[17]. As mentioned previously, the FCR tool was developed based on the BIA-Obesity and ATNI tools[8,18], neither of which have been sufficiently validated to be considered “gold standards”. Examining the extent that these tools align in assessing companies’ product (re)formulation actions/commitments was thus used to establish construct/convergent validity of the FCR tool.

The FCR total scores of the 22 packaged food and beverage companies in this sample were compared to the BIA-Obesity Product Formulation (FORM) domain scores of the same 22 companies (**Supplementary Figure D-1**)[19]. Scores according to both the BIA-Obesity and FCR tools were based on the same policy data (implemented as of December 31, 2017) for the Canadian market, facilitating comparisons of scores between tools. BIA-Obesity FORM scores were compared to both the original FCR total scores, as well as the weighted total scores. Spearman’s rank correlation was used to examine the association between FCR total scores and BIA-Obesity FORM scores. Scores based on the FCR tool were not compared to the ATNI since the latter tool has not been applied in Canada. For all correlations in the validation and internal consistency evaluations, statistical significance was defined as $p<0.05$.

The ranking of companies was similar based on their FCR total scores and BIA-Obesity FORM scores, irrespective of whether the original or weighted FCR scores were used (Supplementary Figure D-1). There were strong positive correlations between BIA-Obesity FORM and FCR original total scores ($r_s=0.91$, $p<0.001$), and between BIA-Obesity scores and weighted FCR scores with additional indicators included ($r_s=0.92$, $p<0.001$) and when additional indicators were excluded ($r_s=0.91$, $p<0.001$). When companies with total scores of 0 for the FCR tool and

BIA-Obesity FORM domain were excluded (to examine if correlations may be zero-inflated), positive correlations of BIA-Obesity scores with FCR original total scores (r_s=0.79, p<0.001), weighted total scores with additional indicators (r_s=0.83, p<0.001) and weighted total scores without additional indicators (r_s=0.82, p<0.001) were weaker but still significant.

The overall similarity in the ranking of companies between the BIA-Obesity and FCR tools is not surprising, given that both tools were designed to evaluate the quality of food companies' reported actions and commitments to reduce energy/portion sizes, sodium, saturated fat, *trans* fat and sugars in their products, based on SMART criteria. Both tools also include indicators related to a company's participation in industry initiatives and positions on government or WHO recommendations related to product (re)formulation. Moreover, like the FCR tool, BIA-Obesity was developed based on existing scientific evidence, government and WHO recommendations, and through extensive consultation with experts.

Nonetheless, several companies ranked differently between tools; these discrepancies can largely be attributed to differences in the measures underlying the tools. For example, the FCR tool awards a full 28 points (out of the total 155 points) to companies reporting that they have already eliminated industrially produced *trans* fats from their product portfolio, whereas the maximum *trans* fat score in the BIA-Obesity tool is 10 (out of 95), a much lower proportion of the total score. Therefore, companies such as Mondelēz who reported having eliminated industrially produced *trans* fats from their global product portfolio ranked better in terms of their FCR total scores compared with their BIA-Obesity FORM scores. Similarly, companies reporting both recent actions and commitments tended to score higher based on the FCR tool, which includes separate sets of indicators for recent actions and ongoing commitments (for each nutrient/component). The BIA-Obesity does not distinguish between recent actions and commitments, disadvantaging companies that report both. Conversely, companies with very weak or no recent actions and/or commitments are penalized more based on the FCR tool than the BIA-Obesity. Overall, compared with the FORM domain of the BIA-Obesity tool, the FCR tool lends less weight to whether a company: participates in industry initiatives concerning product (re)formulation; has a position on government product (re)formulation policy; and/or has established a NP model or nutrition criteria. In comparison, reporting recent actions or

commitments in relation to SMART characteristics (i.e., baseline/target years, breadth across the product portfolio, meaningfulness/significance of the targeted magnitude of reduction, national/global applicability, and transparency/reporting of progress) carry more weight with the FCR tool since they are separated into individual indicators for each nutrient, and for recent actions versus commitments. The BIA-Obesity tool combines multiple SMART characteristics into a single indicator for each nutrient/component.

Overall, the strong positive correlation between FCR total scores and BIA-Obesity FORM scores, and the generally similar ranking of companies between tools, contributes to establishing construct/convergent validity of the FCR tool.

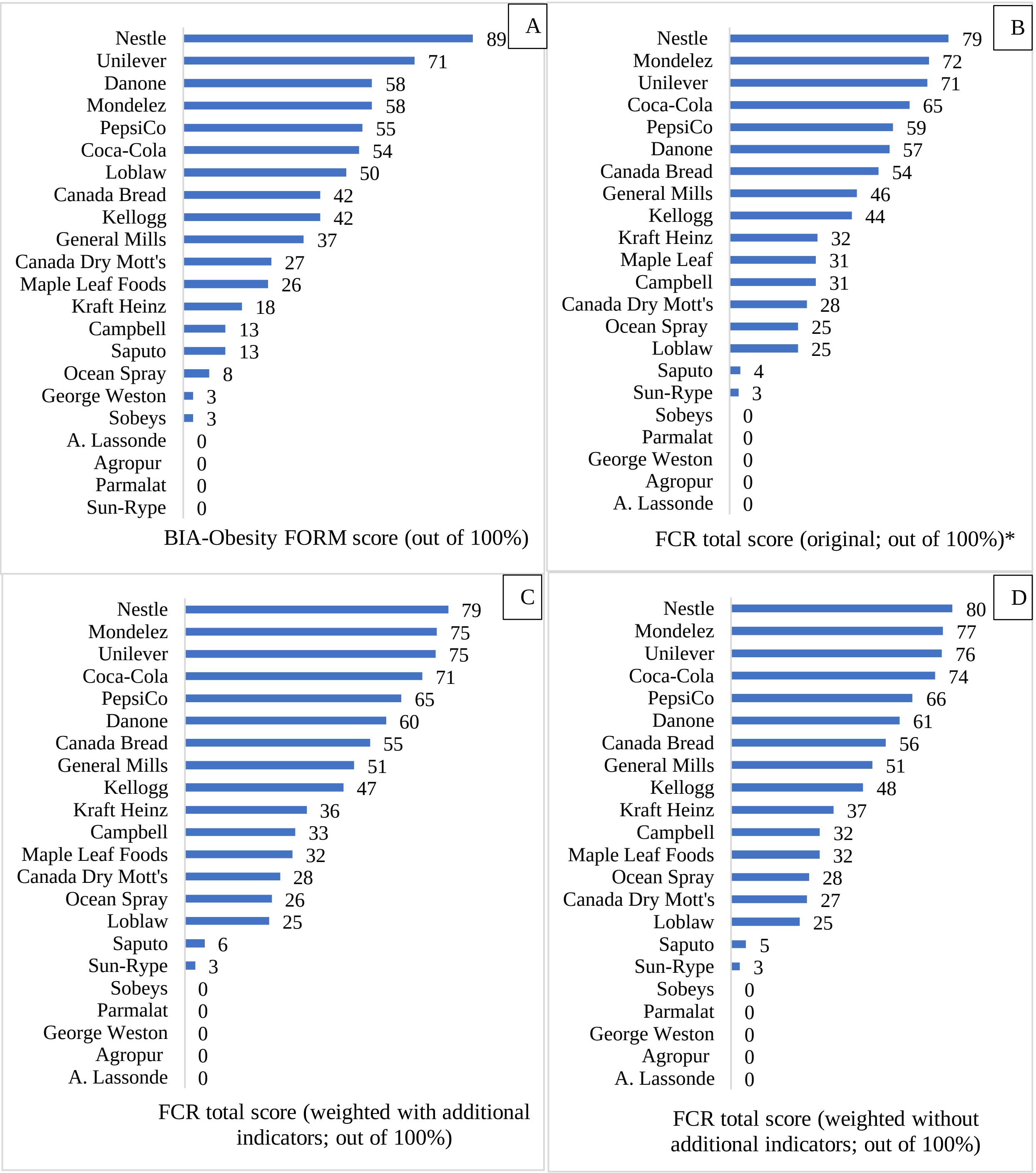

Supplementary Figure D-1. A comparison of companies' scores according to the product formulation ("FORM") domain of the BIA-Obesity tool (A) and the FCR tool (out of 100% for all). FCR tool scores are presented as original total scores (B), weighted total scores with the additional indicators both included (C) and excluded (D).

*The original FCR total scores were out of 65 for the four companies only or primarily offering beverages (A. Lassonde, Canada Dry Mott's, Coca-Cola, and Ocean Spray) and out of 155 for all other companies; scores were been converted to percentages to facilitate comparisons with the BIA-Obesity tool.

Internal consistency

The internal consistency of the FCR tool was examined using companies' scores for sodium since it was the most commonly targeted nutrient and companies scored the best in terms of sodium reduction, on average, resulting in a greater quantity and broader scale of data available for the analyses. Internal consistency was not examined based on scores for multiple nutrients because the same indicators were applied to each nutrient. It was therefore assumed that the internal consistency of the sodium domain would be similar to that of other nutrients/components evaluated by the tool (i.e., energy/portion sizes, saturated fat, *trans* fat, and sugars).

Plots and Spearman's rank correlation coefficients were used to examine whether certain domains (e.g., timebound, breadth, magnitude of reduction, transparency) or indicators (e.g., SOD1.1/7.1) were exerting significantly more or less influence on companies' FCR sodium scores, compared with other domains and indicators in the tool. Companies only or primarily offering beverages were not assigned FCR sodium scores (since sodium contents are typically zero or negligible in these products) and were thus excluded from this assessment of internal consistency (n=4), resulting in the inclusion of 18 companies. Because each nutrient section of the FCR tool includes an identical set of indicators for both reported recent actions and commitments, scores for each domain were combined. For example, the "magnitude of reduction" domain for sodium includes 4 indicators for recent actions (i.e., SOD4.1, SOD4.2, SOD4.3, SOD4.4) and the same set of 4 indicators for commitments (i.e., SOD10.1, SOD10.2, SOD10.3, SOD10.4). SOD4.1 and SOD10.1 assess whether the company specifies the magnitude of reduction that has been achieved (for recent actions) or will be achieved (for commitments); SOD4.2 and SOD10.2, SOD4.3 and SOD10.3, and SOD4.4 and SOD10.4 are paired similarly. Accordingly, for the purposes of the internal consistency evaluation, each company's score for the magnitude of reduction domain was out of 8 (the maximum possible score for sodium remained 32). Both the original and weighted FCR scores for each company were used in the internal consistency analyses (see section above for details about weighting).

Plots were used to examine the relationship between companies' total scores for each of the 6 domains and their total scores for sodium (**Supplementary Figures D-2 and D-3**). Sets of plots were created using both the original unweighted scores and with the new weighted scores. Based

on Supplementary Figures D-2 and D-3, most domains were positively correlated with FCR sodium scores, suggesting that companies scoring higher within each domain generally receive higher sodium scores overall. It is worth noting, however, that the reduction/commitment domain is only out of 2, with 1 binary indicator assessing whether the company reported a recent reduction in sodium and 1 indicator assessing whether they reported a commitment. Therefore, the ability to assess the strength of the correlation of between this domain and sodium total scores (and with other domain scores in subsequent analyses) was limited.

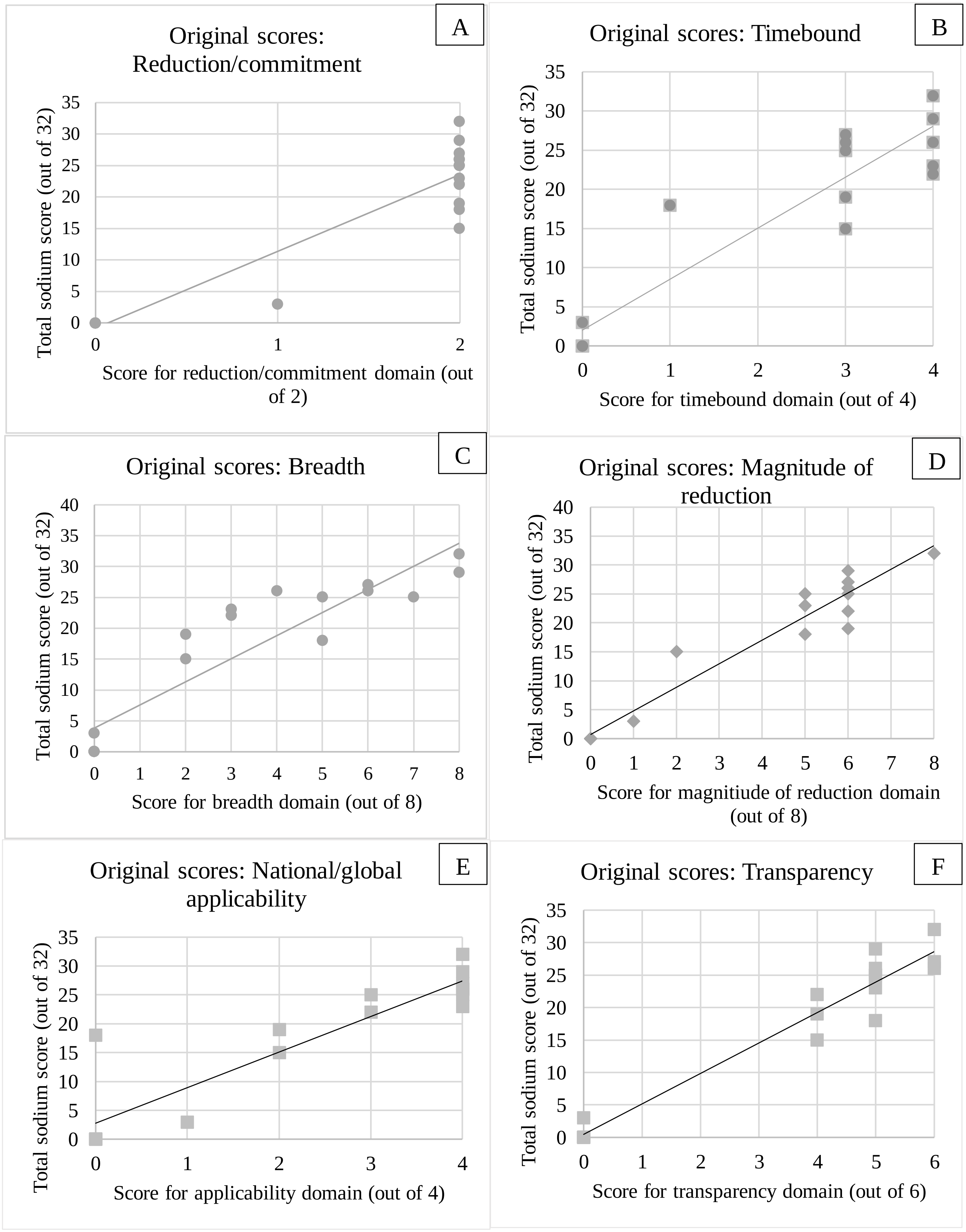

Supplementary Figure D-2. Correlations between companies' original (unweighted) total scores for sodium and their scores for the reduction/commitment (A), timebound (B), breadth (C), magnitude of reduction (D), national/global applicability (E), and transparency (F) domains.

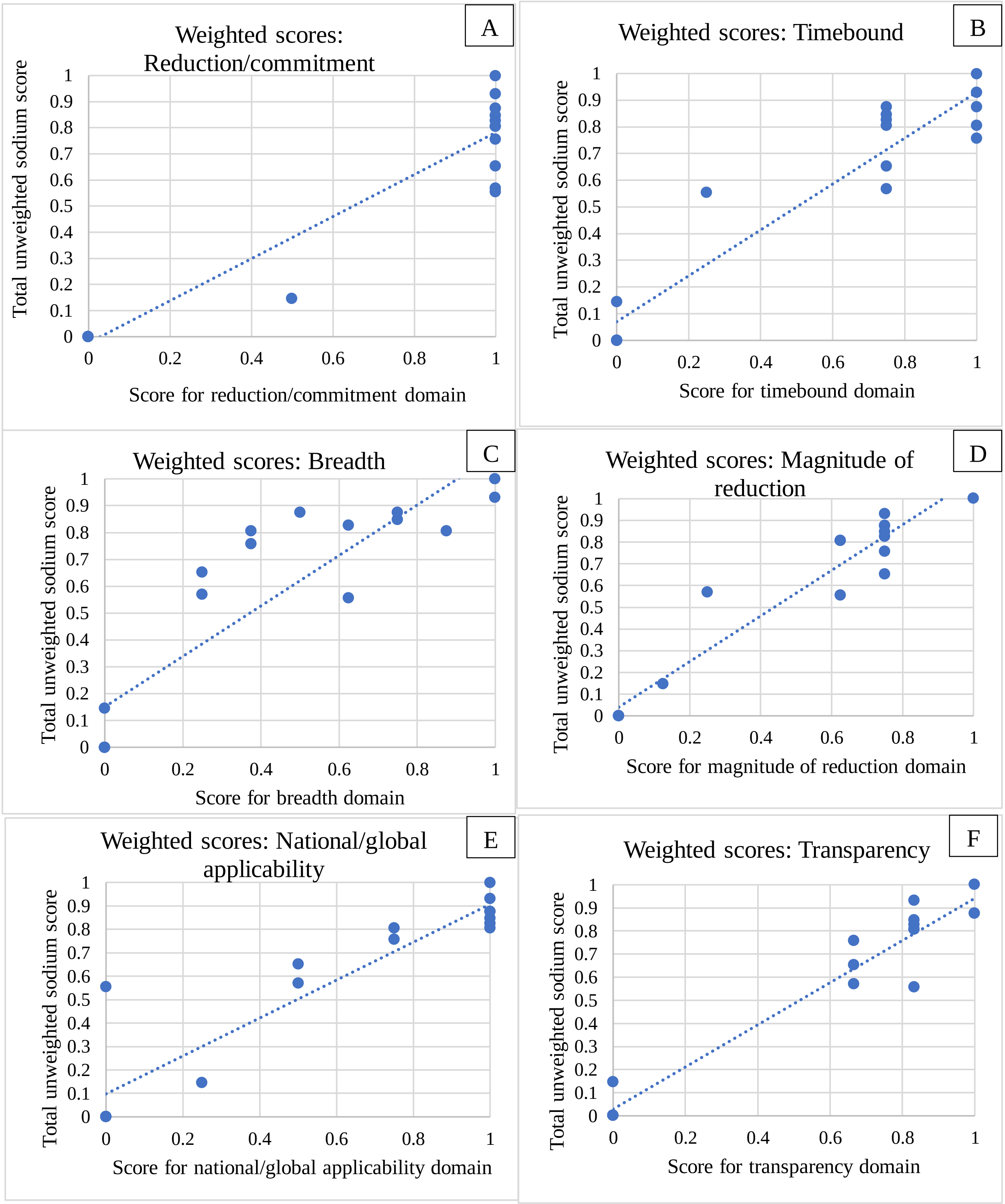

Supplementary Figure D-3. Correlations between companies' weighted total scores for sodium and their scores for the reduction/commitment (A), timebound (B), breadth (C), magnitude of reduction (D), national/global applicability (E), and transparency (F) domains.

Stepwise testing of the influence of individual indicators on correlations

To further explore these relationships, Spearman's rank correlations were conducted between companies' scores for each domain and their total sodium scores. Initial correlations included all indicators and then pairs of identical indicators were excluded one at a time to examine whether one type of indicator was considerably more or less correlated with sodium scores than others (e.g., SOD2.1 and SOD8.1 were both removed at once), similar to a backward elimination regression approach. Sodium score denominators were reduced accordingly as indicators were removed. All correlations were conducted both with all 18 companies included, and excluding companies with sodium scores of 0 (n=5) to determine whether any of the correlations were zero-inflated (companies receiving a score of 0 for a particular indicator or domain but a total score >0 remained in the analysis). Additionally, based on the plots in Supplementary Figures D-2 and D-3, there was reason to suspect potential outlier scores within some of the domains, which may inflate or deflate the strength of correlations between domains and total scores. Boxplots were used to examine the distribution of total sodium scores for each score within the 6 domains of the FCR tool and identify outlier scores (**Supplementary Figure D-4**). Outliers were identified for the timebound (Saputo), breadth (Saputo), national/global applicability (Canada Bread, Nestlé) and transparency domains (Canada Bread, Saputo). Sensitivity analyses examined whether excluding these companies changed the strength of the correlations.

Results of the correlations are presented in **Supplementary Table D-1**. Overall, there were strong positive correlations between each of the 6 domains and sodium scores, and most of these correlations were maintained when the 5 companies with sodium scores of 0 were excluded. In the recent reduction/commitment domain, there was a relatively strong positive correlation when both indicators were included but the relationship was no longer significant when companies with total sodium scores of 0 were excluded. Similar results were observed when only the reduction or commitment indicators were included (SOD1.1 and SOD7.1, respectively).

For the timebound domain, there were strong positive correlations when all indicators were included. The strength of the correlation was reduced when companies with total sodium scores of 0 were excluded but statistical significance was maintained. Reporting a baseline year (SOD2.1 and SOD8.1) was more strongly correlated with higher sodium scores than reporting a

target year (SOD2.2 and SOD8.2). When outliers and companies with sodium scores of 0 were excluded, the correlation between the timebound domain (with all indicators included) and sodium scores was weakened and no longer statistically significant.

There were strong positive correlations between breadth scores and total sodium scores, even when companies with sodium scores of 0 were removed. Excluding the SOD3.2/9.2 (reduction/commitment applies to all relevant products in the company's portfolio) and SOD3.1/9.1 (reduction/commitment applies to specified brands or food categories) indicators reduced the strength of the positive correlation the most, although all correlations remained strong, irrespective of which indicators were excluded. Correlations also remained strong and statistically significant when companies with sodium scores of 0 were excluded, and when outliers were omitted.

In the magnitude of reduction domain, there were strong positive correlations with sodium scores, regardless of whether companies with sodium scores of 0 were included. Excluding the SOD4.3/10.3 indicators (reduction/commitment reportedly aligns with Health Canada's national sodium reduction targets[20]) reduced the strength of the positive correlation the most. The strength of the correlation between domain scores and sodium scores was also reduced when the SOD4.2/10.2 indicators were excluded (magnitude of reduction is reported in a meaningful and measurable way).

National/global applicability scores were strongly correlated with sodium scores, even when companies with sodium scores of 0 were excluded. Correlations were of similar strength when only the national (SOD5.1/11.2) and global applicability indicators (SOD5.2/11.2) indicators were included, and all correlations were statistically significant, even when outliers were excluded.

Lastly, in the transparency domain, there were strong correlations between transparency scores and sodium scores, irrespective of whether companies with sodium scores of 0 were excluded. Excluding the SOD6.3/12.3 indicators (regular annual/biannual reporting of progress) reduced the strength of the positive correlation the most; all correlations retained statistical significance.

However, excluding outliers and companies with sodium scores of 0 resulted in considerably weaker correlations that were no longer significant when the SOD6.2/12.2 (availability and consolidation of publicly available information concerning sodium reduction) and SOD6.3/12.3 (regular reporting of progress) variables were excluded, suggesting that these variables within the transparency domain are particularly influential in determining companies' sodium scores.

Overall, in most cases, excluding individual pairs of indicators increased or reduced the strength of correlations slightly; however, most correlations maintained statistical significance, even when companies with sodium scores of 0 were excluded. With the exception of the reduction/commitment domain (which is unique since it only included two indicators, resulting in possible scores of only 0, 1 or 2), no domains stood out as being uncorrelated or weakly correlated with sodium scores, which suggests that nearly all domains and indicators are influencing companies' sodium scores. Scores for the reduction/commitment domain were not significantly correlated with sodium scores when companies with sodium scores of 0 were excluded, suggesting that scores in this domain do not affect overall sodium scores. This is perhaps unsurprising given that the indicators in this domain simply assess whether a company reports any type of recent action or commitment concerning sodium reduction. A company could therefore report a very weak recent action or commitment and score well in this domain, but score poorly in all other domains, resulting in a low total score for sodium. Additionally, excluding certain indicators from a domain more greatly reduced the strength of the correlation between domain and sodium scores than other indicators, indicating that the former indicators have more influence on companies' sodium scores than other indicators within that domain (e.g., SOD2.1/8.1, SOD 3.1/9.1, SOD3.2/9.2, SOD4.2/10.2, SOD4.3/10.3, SOD6.2/12.2, SOD6.3/12.3).

Supplementary Table D-1. Spearman's rank correlations between companies' score for each domain of the sodium section of the FCR scoring tool and their total sodium scores, presented for the inclusion of all indicators in the FCR scoring tool, and with the inclusion of selected indicators in each domain. Correlations are shown for all 18 companies in the sample (A) and for a subsample that excluded outliers (B). Results are also presented for both the inclusion of all companies (C), and exclusion of companies with total sodium scores of 0 (D) to account for potential zero-inflation of correlations.

**Inclusion of all indicators in the FCR scoring tool**								
			**Reduction/commitment[1]**	**Timebound[2]**	**Breadth[3]**	**Magnitude of reduction[4]**	**National/global applicability[5]**	**Transparency[6]**
**All companies (A)**	**Scores of 0 included (C; n=18)**	**r_s**	0.84	0.84	0.94	0.92	0.93	0.93
		**p-value**	**<0.001**	**<0.001**	**<0.001**	**<0.001**	**<0.001**	**<0.001**
	**Scores of 0 excluded (D; n=13)**	**r_s**	0.46	0.59	0.85	0.78	0.86	0.82
		**p-value**	0.11	**0.03**	**<0.001**	**0.002**	**<0.001**	**<0.001**
**Outliers excluded[7] (B)**	**Scores of 0 included (C)**	**n**	N/A	17	17	N/A	16	16
		**r_s**	N/A	0.82	0.93	N/A	**0.95**	**0.95**
		**p-value**	N/A	**<0.001**	**<0.001**	N/A	**<0.001**	**<0.001**
	**Scores of 0 excluded (D)**	**n**	N/A	12	12	N/A	11	11
		**r_s**	N/A	0.47	0.81	N/A	0.86	0.84
		**p-value**	N/A	0.13	**0.001**	N/A	**<0.001**	**0.001**
**Inclusion of selected indicators only (as indicated in brackets below)**								
			**Reduction/commitment (SOD 1.1 only)[1]**	**Timebound (SOD 2.1 and 8.1 only)[2]**	**Breadth (SOD 3.1,3.2,3.3,9.1,9.2 and 9.3 only)[3]**	**Magnitude of reduction (SOD 4.1, 4.2, 4.3, 10.1, 10.2 and 10.3 only)[4]**	**National/global applicability (SOD 5.1 and 11.1 only)[5]**	**Transparency (SOD 6.1, 6.2, 12.1 and 12.2 only)[6]**
**All companies (A)**	**Scores of 0 included (C; n=18)**	**r_s**	0.83	0.87	0.93	0.95	0.90	0.85
		**p-value**	**<0.001**	**<0.001**	**<0.001**	**<0.001**	**<0.001**	**<0.001**
	**Scores of 0 excluded (D; n=13)**	**r_s**	0.46	0.69	0.84	0.87	0.78	0.56
		**p-value**	0.11	**0.009**	**<0.001**	**<0.001**	**0.001**	**0.045**
**Outliers excluded[7] (B)**	**Scores of 0 included (C)**	**n**	N/A	17	17	N/A	16	16
		**r_s**	N/A	0.85	0.93	N/A	0.93	0.87
		**p-value**	N/A	**<0.001**	**<0.001**	N/A	**<0.001**	**<0.001**
		**n**	N/A	12	12	N/A	11	11

	**Scores of 0 excluded (D)**	**r_s**	N/A	0.59	0.80	N/A	0.78	0.17
		**p-value**	N/A	**0.04**	**0.002**	N/A	**0.004**	0.61
**Inclusion of selected indicators only (as indicated in brackets below)**								
			**Reduction/commitment (SOD 7.1 only)[1]**	**Timebound (SOD 2.2 and 8.2 only)[2]**	**Breadth (SOD 3.1, 3.2, 3.4, 9.1, 9.2 and 9.4 only)[3]**	**Magnitude of reduction (SOD 4.1, 4.2, 4.4, 10.1, 10.2, 10.4)[4]**	**National/global applicability (SOD 5.2 and 11.2)[5]**	**Transparency (SOD 6.1, 6.3, 12.1 and 12.3 only)[6]**
**All companies (A)**	**Scores of 0 included (C; n=18)**	**r_s**	0.79	0.77	0.96	0.84	0.91	0.91
		**p-value**	**<0.001**	**<0.001**	**<0.001**	**<0.001**	**<0.001**	**<0.001**
	**Scores of 0 excluded (D; n=13)**	**r_s**	N/A (all values were 1's)	0.37	0.91	0.6	0.83	0.79
		**p-value**	N/A (all values were 1's)	0.21	**<0.001**	**0.03**	**<0.001**	**0.001**
**Outliers excluded[7] (B)**	**Scores of 0 included (C)**	**n**	N/A	17	17	N/A	16	16
		**r_s**	N/A	0.73	0.96	N/A	0.93	0.92
		**p-value**	N/A	**<0.001**	**<0.001**	N/A	**<0.001**	**<0.001**
	**Scores of 0 excluded (D)**	**n**	N/A	12	12	N/A	11	11
		**r_s**	N/A	0.17	0.89	N/A	0.82	0.56
		**p-value**	N/A	0.59	**<0.001**	N/A	**0.002**	0.07
**Inclusion of selected indicators only (as indicated in brackets below)**								
			**Reduction/commitment[1]**	**Timebound[2]**	**Breadth (SOD 3.1, 3.3, 3.4, 9.1, 9.3 and 9.4 only)[3]**	**Magnitude of reduction (SOD 4.1, 4.3, 4.4, 10.1, 10.3 and 10.4 only)[4]**	**National/global applicability[5]**	**Transparency (SOD 6.2, 6.3, 12.2 and 12.3 only)[6]**
**All companies (A)**	**Scores of 0 included (C; n=18)**	**r_s**	N/A	N/A	0.91	0.87	N/A	0.93
		**p-value**	N/A	N/A	**<0.001**	**<0.001**	N/A	**<0.001**
	**Scores of 0 excluded (D; n=13)**	**r_s**	N/A	N/A	0.79	0.63	N/A	0.82
		**p-value**	N/A	N/A	**0.001**	**0.02**	N/A	**<0.001**
**Outliers excluded[7] (B)**	**Scores of 0 included (C)**	**n**	N/A	N/A	17	N/A	N/A	16
		**r_s**	N/A	N/A	0.91	N/A	N/A	0.95
		**p-value**	N/A	N/A	**<0.001**	N/A	N/A	**<0.001**
	**Scores of 0 excluded (D)**	**n**	N/A	N/A	12	N/A	N/A	11
		**r_s**	N/A	N/A	0.74	N/A	N/A	0.84

		**p-value**	N/A	N/A	**0.006**	N/A	N/A	**0.001**
**Inclusion of selected indicators only (as indicated in brackets below)**								
			**Reduction/commitment[1]**	**Timebound[2]**	**Breadth (SOD 3.2, 3.3, 3.4, 9.2, 9.3 and 9.4 only)[3]**	**Magnitude of reduction (SOD 4.2, 4.3, 4.4, 10.2, 10.3 and 10.4 only)[4]**	**National/global applicability[5]**	**Transparency[6]**
**All companies (A)**	**Scores of 0 included (C; n=18)**	**r_s**	N/A	N/A	0.9	0.92	N/A	N/A
		**p-value**	N/A	N/A	**<0.001**	**<0.001**	N/A	N/A
	**Scores of 0 excluded (D; n=13)**	**r_s**	N/A	N/A	0.77	0.78	N/A	N/A
		**p-value**	N/A	N/A	**0.002**	**0.002**	N/A	N/A
**Outliers excluded[7] (B)**	**Scores of 0 included (C)**	**n**	N/A	N/A	17	N/A	N/A	N/A
		**r_s**	N/A	N/A	0.90	N/A	N/A	N/A
		**p-value**	N/A	N/A	**<0.001**	N/A	N/A	N/A
	**Scores of 0 excluded (D)**	**n**	N/A	N/A	12	N/A	N/A	N/A
		**r_s**	N/A	N/A	0.71	N/A	N/A	N/A
		**p-value**	N/A	N/A	**0.01**	N/A	N/A	N/A

[1]SOD1.1 and SOD7.1 assess whether the company reports a recent reduction or commitment to reduce sodium, respectively.
[2]SOD2.1 and SOD8.1 assess whether the companies' recent reduction or commitment, respectively, are reported in the context of a baseline year (i.e., starting point). SOD2.2 and SOD8.2. assess whether the companies' recent reduction or commitment, respectively, are reported in the context of a target year (i.e., end point).
[3]SOD3.1 and SOD9.1 assess whether the reduction or commitment, respectively, are applied to all relevant products in the company's product portfolio. SOD3.2 and SOD9.2 assess whether the company specifies which brands or food categories were or would be included in the reduction or commitment, respectively. SOD3.3 and SOD9.3 assess whether the company specifies the sales volume or proportion of total sales that were or would be reduced in sodium, respectively. SOD3.4 and SOD9.4 assess whether the company discloses the number and/or percentage of products that have been or will be reduced in sodium, respectively.
[4]SOD4.1 and SOD10.1 assess whether the company specifies that magnitude of reduction that has been or will be achieved, respectively. SOD4.2 and SOD10.2 assess whether the magnitude of reduction included in the reduction or commitment, respectively, is stated in a measurable and meaningful way (i.e., with a denominator). SOD4.3 and SOD10.3 assess whether the company's recent reduction or commitment to reduce sodium, respectively, reportedly aligns with national voluntary sodium reduction targets. SOD4.4 and SOD10.4 assess whether the company's recent reduction or commitment to reduction sodium, respectively, reportedly align with published policy objectives or nutrient targets of global public health organizations (e.g., WHO).
[5]SOD5.1 and SOD11.1 assess whether the reduction or commitment, respectively, is applicable to the country of interest. SOD5.2 and SOD11.2 assess whether the reduction or commitment, respectively, has been or will be applied consistently in all countries in which the company operates.
[6]SOD6.1 and SOD12.1 assess whether the reduction or commitment, respectively, is publicly reported. SOD6.2 and SOD12.2 assess whether publicly reported information about the reduction or commitment, respectively, is consolidated and easy to locate. SOD6.3 and SOD12.3 assess whether the company has reported or commits to report their reformulation progress on a regular basis.
[7]Outliers were identified from boxplots of the distribution of total sodium scores for each score within the 6 domains of the FCR tool (Supplementary Figure D-4). Outliers were identified in the timebound (Saputo), breadth (Saputo), national/global applicability (Canada Bread, Nestlé) and transparency domains (Canada Bread, Saputo).

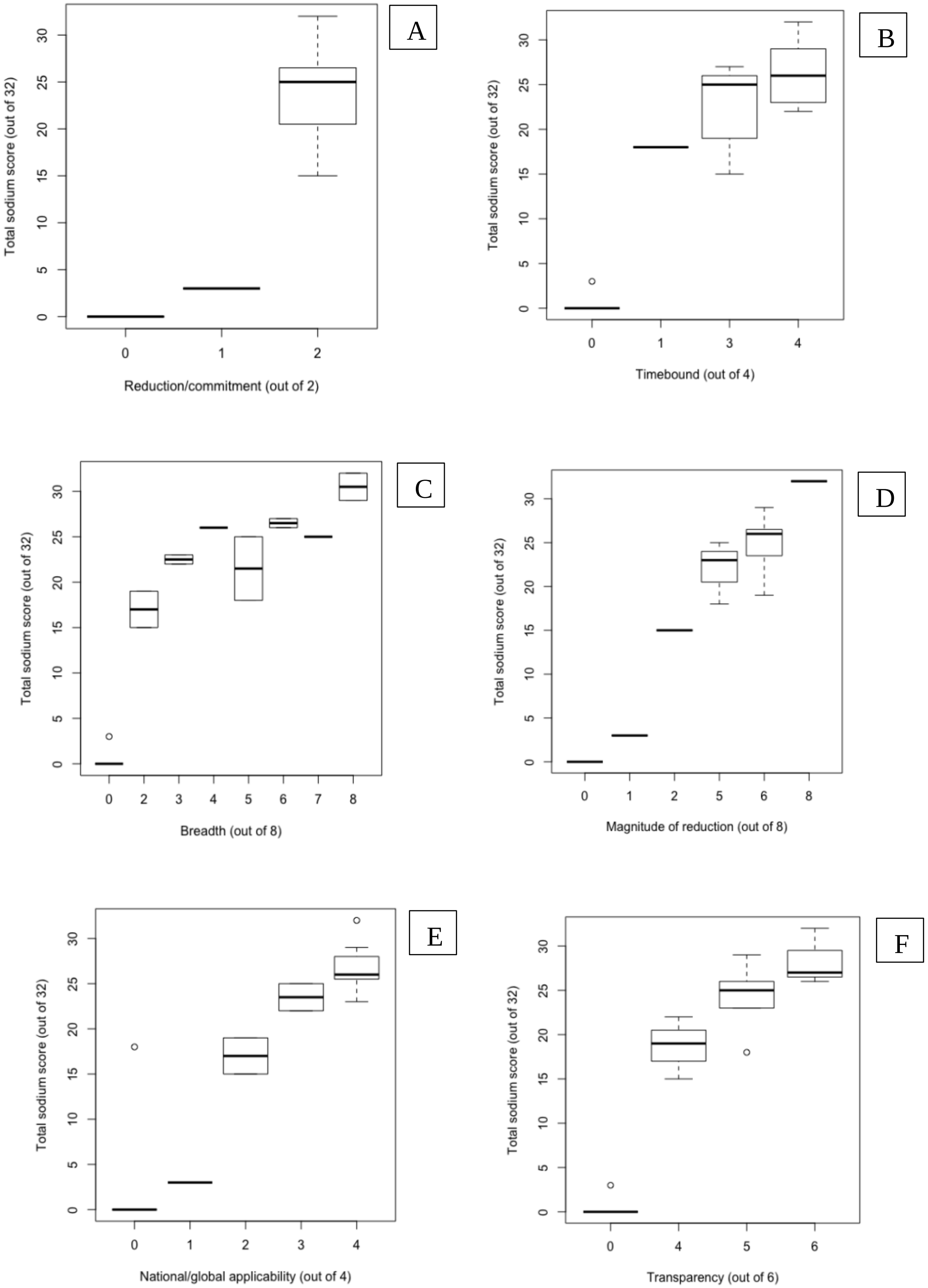

Supplementary Figure D-4. Boxplots of the distribution of total sodium scores for each score within the 6 domains of the FCR tool: reduction/commitment (A); timebound (B); breadth (C); magnitude of reduction (D); national/global applicability (E); and transparency (F).

Examining correlations between domains

A correlation matrix was constructed to examine correlations between different domains of the FCR tool (**Supplementary Table D-2**). When all companies were included, statistically significant positive correlations were observed between all domains. When companies with total sodium scores of 0 were excluded, the strength of the correlations were reduced overall and correlations between the following domains were no longer significant: reduction/commitment and timebound (p=0.051); reduction/commitment and national/global applicability (0.44); timebound and breadth (p=0.13); and breadth and national/global applicability (p=0.07). There are a couple of possible explanations of these findings. First, as described previously, all companies who report any recent reduction in sodium and/or commitment to reduce sodium receive points in the reduction/commitment domain; a company may therefore score high in this domain but low in others if their reported recent reduction or commitment is relatively weak. Conversely, many companies scored relatively poorly in the breadth domain, which includes four indicators concerning the extent that companies report their recent reduction or commitment included all relevant brands and/or food categories. Nonetheless, domains were positively correlated overall, irrespective of whether companies with sodium scores of 0 were included, suggesting internal consistency of the 6 domains within the FCR tool.

Supplementary Figure D-5 displays companies' weighted sodium scores, with a breakdown by score for each of the 6 domains, demonstrating which domains each company scored higher in relative to others. Overall, nearly all companies received full points for the reduction/commitment domain, indicating that they reported both recent actions and ongoing commitments to reduce sodium in their products as of the end of 2017. Conversely, breadth (of the reduction/commitment across a company's product portfolio) was the lowest-scoring domain, on average. This suggests that the breadth domain was particularly important in separating the higher-scoring companies from those with lower scores, as evidenced by the top-scoring companies, Nestlé and Mondelēz, receiving full points for the breadth domain.

Supplementary Table D-2. Spearman's rank correlations between companies' scores for different domains of the sodium section of the FCR scoring tool.

All companies (n=18)												
	**Reduction/commitment (out of 2)**		**Timebound (out of 4)**		**Breadth (out of 8)**		**Magnitude of reduction (out of 8)**		**National/global applicability (out of 4)**		**Transparency (out of 6)**	
	**Coefficient**	**p-value**	**Coefficient**	**p-value**	**Coefficient**	**p-value**	**Coefficient**	**p-value**	**Coefficient**	**p-value**	**Coefficient**	**p-value**
**Reduction/commitment (out of 2)**	N/A	N/A	0.89	**<0.001**	0.79	**<0.001**	0.91	**<0.001**	0.82	**<0.001**	0.94	**<0.001**
**Timebound (out of 4)**	0.89	**<0.001**	N/A	N/A	0.75	**<0.001**	0.89	**<0.001**	0.92	**<0.001**	0.90	**<0.001**
**Breadth (out of 8)**	0.79	**<0.001**	0.75	**<0.001**	N/A	N/A	0.87	**<0.001**	0.78	**<0.001**	0.87	**<0.001**
**Magnitude of reduction (out of 8)**	0.91	**<0.001**	0.89	**<0.001**	0.87	**<0.001**	N/A	N/A	0.86	**<0.001**	0.94	**<0.001**
**National/global applicability (out of 4)**	0.82	**<0.001**	0.92	**<0.001**	0.78	**<0.001**	0.86	**<0.001**	N/A	N/A	0.86	**<0.001**
**Transparency (out of 6)**	0.94	**<0.001**	0.90	**<0.001**	0.87	**<0.001**	0.94	**<0.001**	0.86	**<0.001**	N/A	N/A
**Excluding companies with total scores of 0 for sodium (n=13)**												
	**Reduction/commitment (out of 2)**		**Timebound (out of 4)**		**Breadth (out of 8)**		**Magnitude of reduction (out of 8)**		**National/global applicability (out of 4)**		**Transparency (out of 6)**	
	**Coefficient**	**p-value**	**Coefficient**	**p-value**	**Coefficient**	**p-value**	**Coefficient**	**p-value**	**Coefficient**	**p-value**	**Coefficient**	**p-value**
**Reduction/commitment (out of 2)**	N/A	N/A	0.74	**0.004**	0.55	0.051	0.69	**0.009**	0.44	0.13	0.89	**<0.001**
**Timebound (out of 4)**	0.74	**0.004**	N/A	N/A	0.44	0.13	0.67	**0.01**	0.80	**<0.001**	0.70	**0.008**
**Breadth (out of 8)**	0.55	0.051	0.44	0.13	N/A	N/A	0.71	**0.007**	0.52	0.07	0.73	**0.005**
**Magnitude of reduction (out of 8)**	0.69	**0.009**	0.67	**0.01**	0.71	**0.007**	N/A	N/A	0.60	**0.03**	0.79	**0.001**
**National/global applicability (out of 4)**	0.44	0.13	0.80	**<0.001**	0.52	0.07	0.60	**0.03**	N/A	N/A	0.59	**0.03**
**Transparency (out of 6)**	0.89	**<0.001**	0.7	**0.008**	0.73	**0.005**	0.79	**0.001**	0.59	**0.03**	N/A	N/A

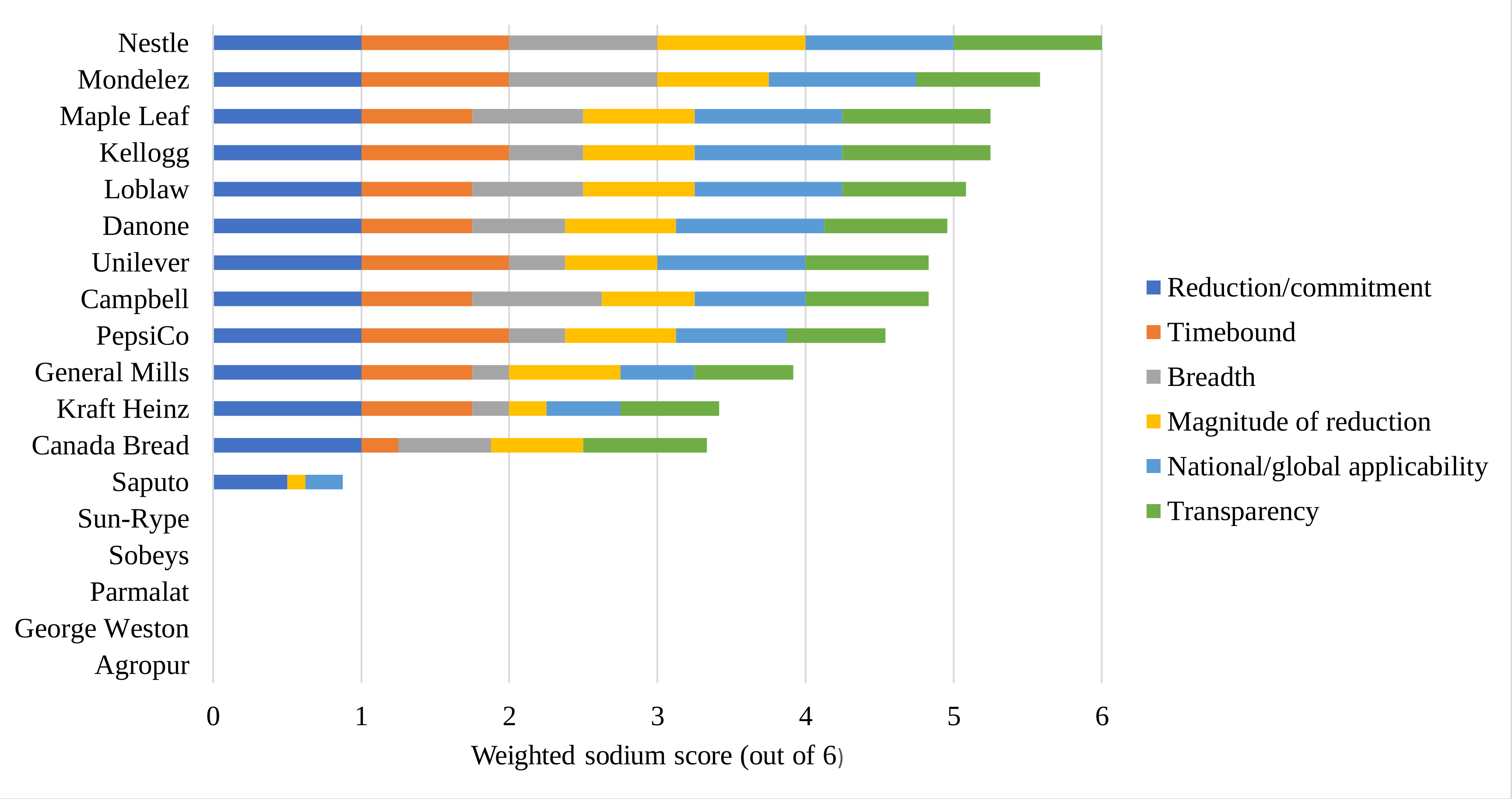

Supplementary Figure D-5. FCR tool sodium scores by company, with equal weights assigned to each of the 6 domains constituting the score (reduction/commitment, timebound, breadth, magnitude of reduction, national/global applicability, and transparency).

A comparison of companies' original and weighted sodium scores is provided in **Supplementary Figure D-6**. Overall, companies ranked similarly, irrespective of which scores were used, particularly among the highest-scoring companies. There was some difference in the ranking of companies with lower or average scores between the original and weighted scores. Specifically, when the weighted scores were used instead of the original scores, Kellogg moved ahead of Loblaw, Danone moved ahead of Unilever, Unilever moved ahead and became tied with Campbell, and Kraft Heinz moved ahead of Canada Bread.

Supplementary Figures D-7-18 demonstrate the effect of eliminating one of the six domains of the FCR tool on companies' rankings based on their sodium scores, using both the original and weighted scores. For example, we examined how the ranking of companies would change if the "magnitude of reduction" domain was eliminated and companies' scores were adjusted accordingly. In general, most differences were apparent for companies with mid-range sodium scores, and for domains with larger numbers of indicators (e.g., breadth, magnitude of reduction), with less change in the rankings of the highest-scoring companies. These figures are useful in further understanding how the different domains of the FCR tool influence companies' scores, and how the rankings of companies would differ if each one was not excluded from the sodium scores. Specific observations related to each comparison are presented to the right of each figure (**Supplementary Figures D-7-18**).

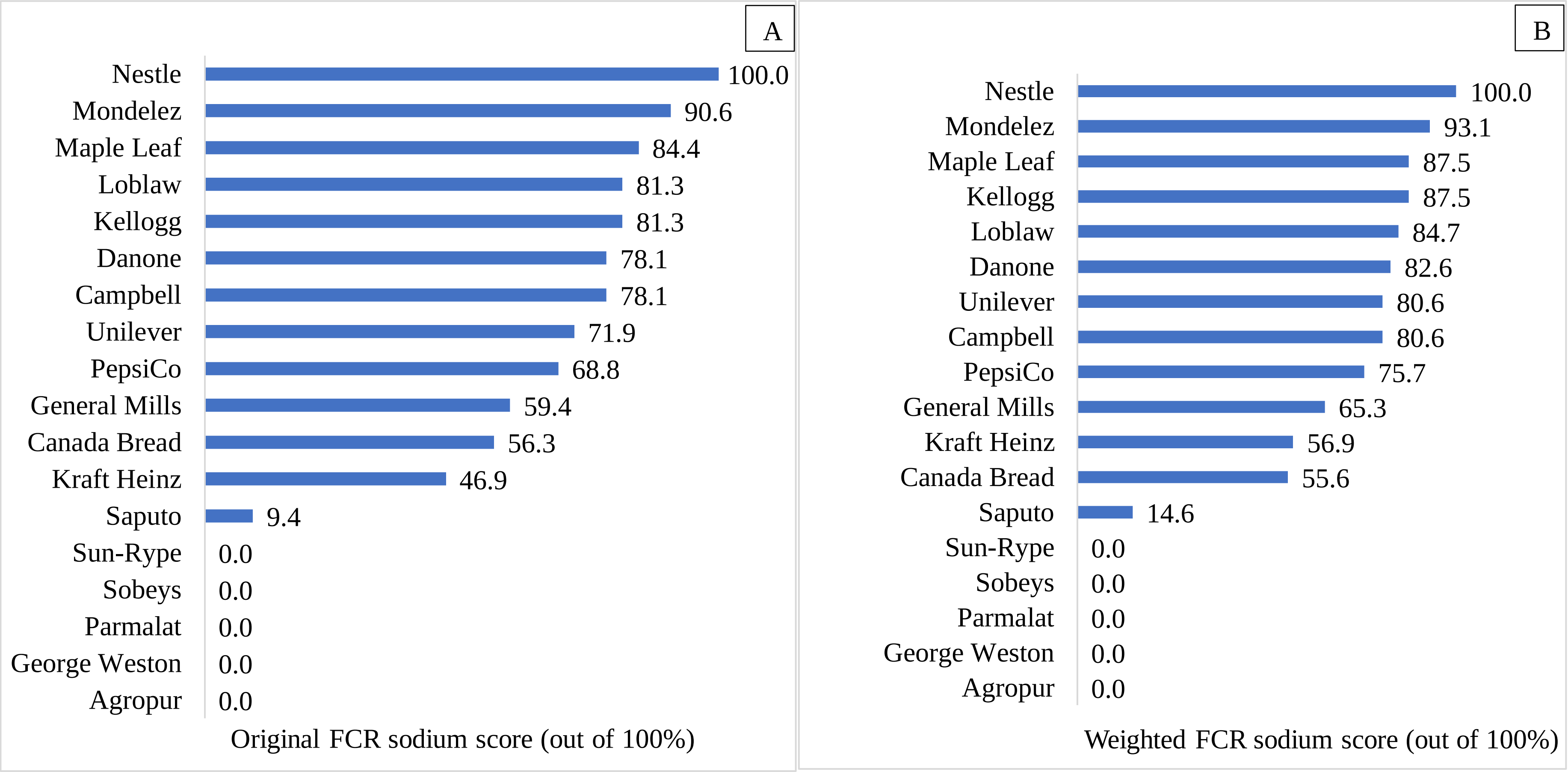

Supplementary Figure D-6. A comparison of companies' original (unweighted) sodium scores versus their weighted sodium scores according to the FCR scoring tool.

Supplementary Figure D-7. Original sodium scores vs. recent actions/commitments domain removed (SOD1.1, 7.1).

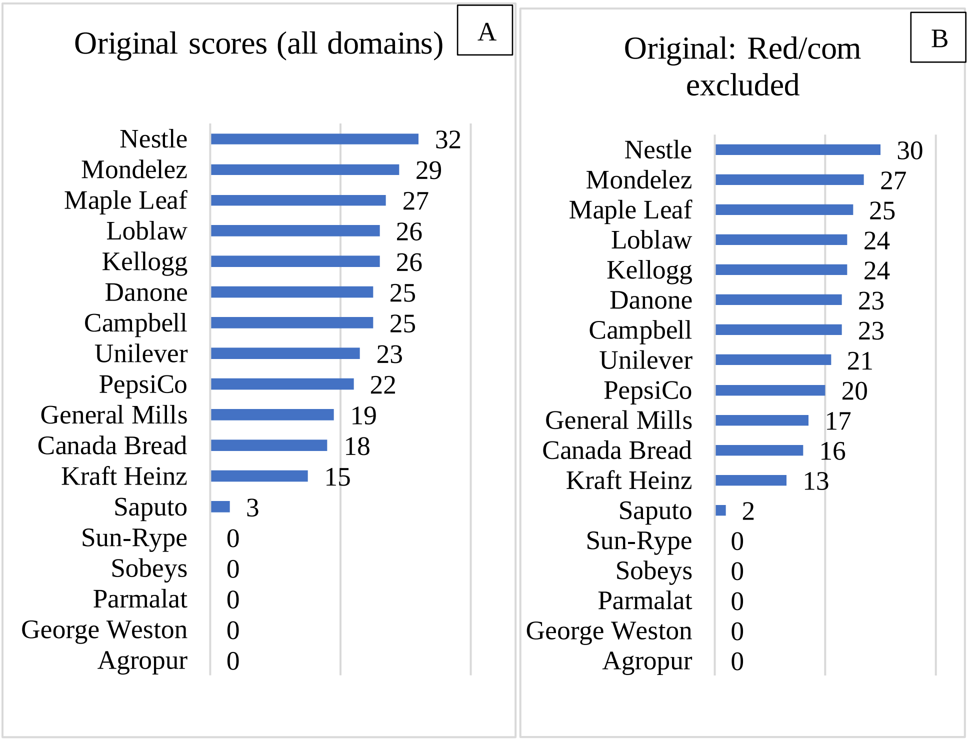

Removing the recent action/commitment domain did not change the ranking of companies

Supplementary Figure D-8. Weighted sodium scores vs. recent actions/commitments domain removed (SOD1.1, 7.1).

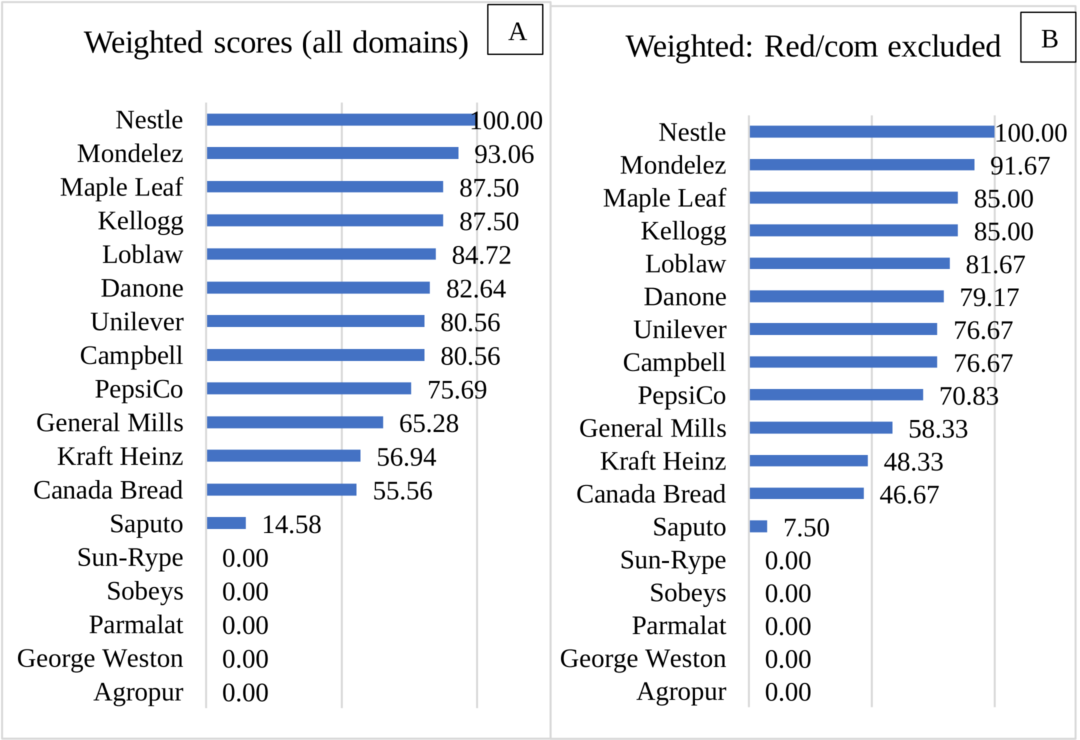

Removing recent actions and commitments domain did not change the ranking of companies when the scores were weighted equally.

Supplementary Figure D-9. <u>Original</u> sodium scores vs. timebound domain removed (SOD2.1, 2.2, 8.1, 8.2).

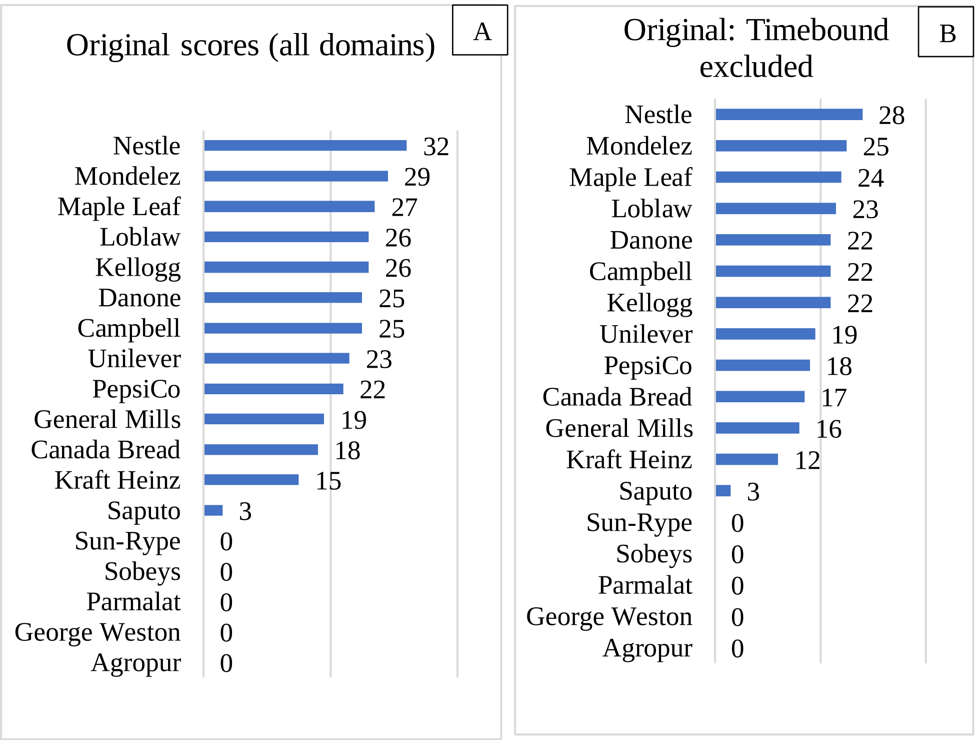

Removing the timebound domain caused in Kellogg to move ahead and become tied with Danone and Campbell.

Canada Bread moved ahead of General Mills.

Supplementary Figure D-10. <u>Weighted</u> sodium scores vs. timebound domain removed (SOD2.1, 2.2, 8.1, 8.2).

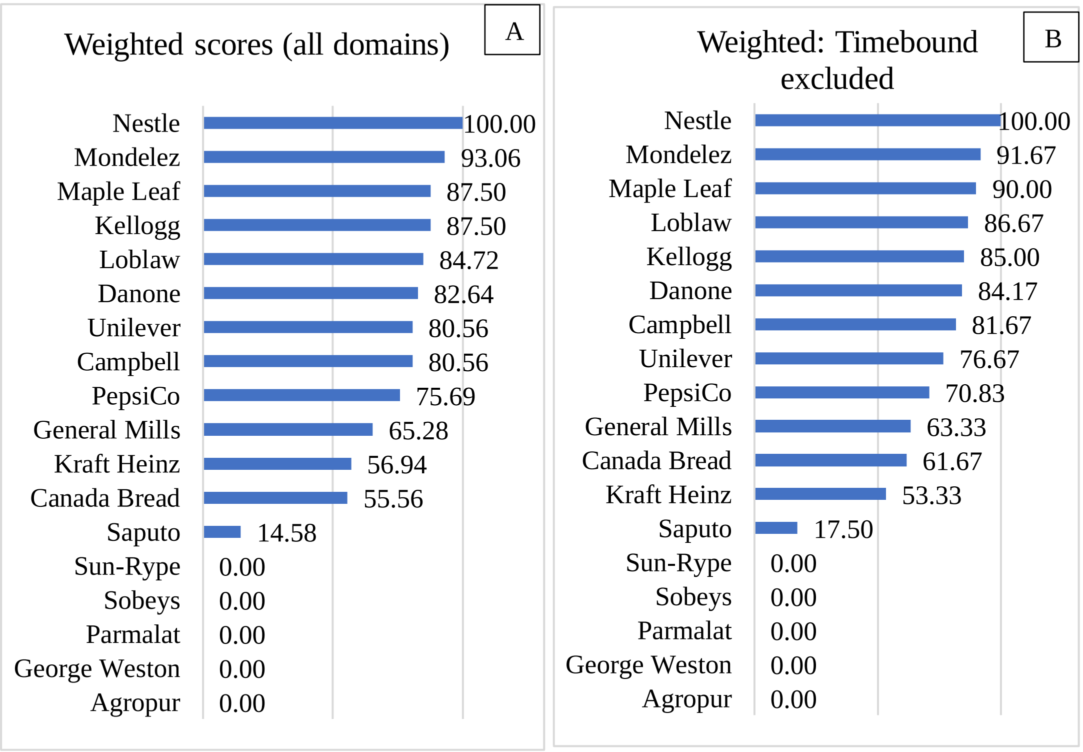

Removing the timebound domain caused Loblaw to move ahead of Kellogg.

Campbell moved ahead of Unilever instead of being tied.

Supplementary Figure D-11. Original sodium scores vs. breadth domain removed (SOD3.1, 3.2, 3.3, 3.4, 9.1, 9.2, 9.3, 9.4).

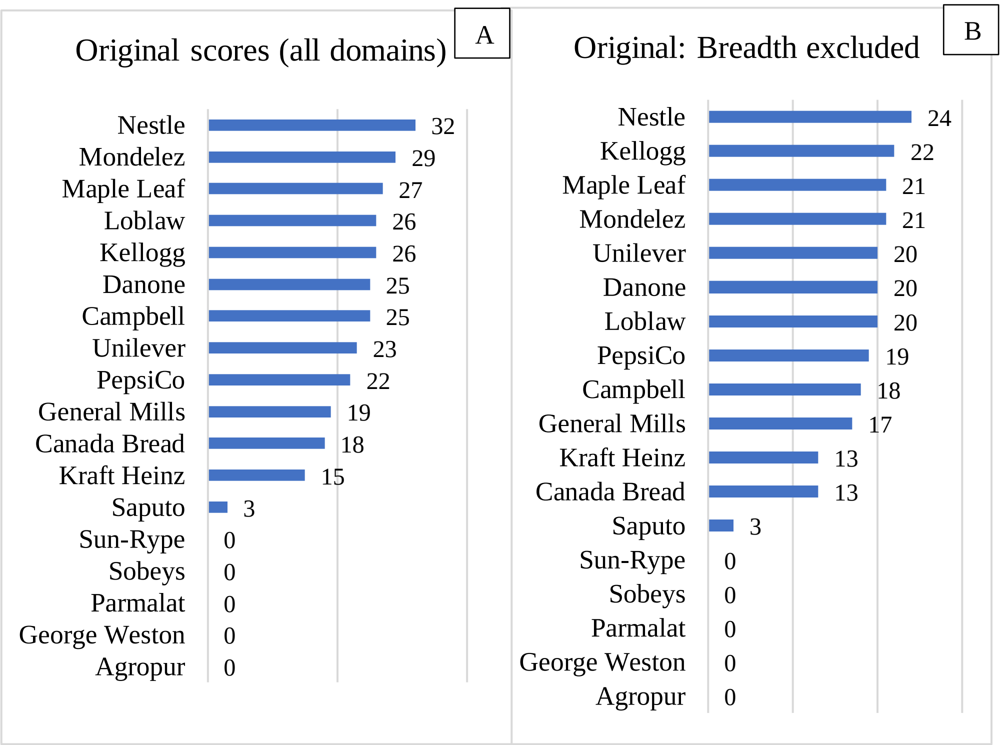

Removing the breadth domain caused Kellogg to move ahead and become ranked 2nd. Mondelez became tied with Maple Leaf. Unilever moved ahead of Danone, Loblaw and Campbell. Loblaw's ranking fell, and became tied with Unilever and Danone. Kraft Heinz moved ahead of Canada Bread.

Supplementary Figure D-12. Weighted sodium scores vs. breadth domain removed (SOD3.1, 3.2, 3.3, 3.4, 9.1, 9.2, 9.3, 9.4).

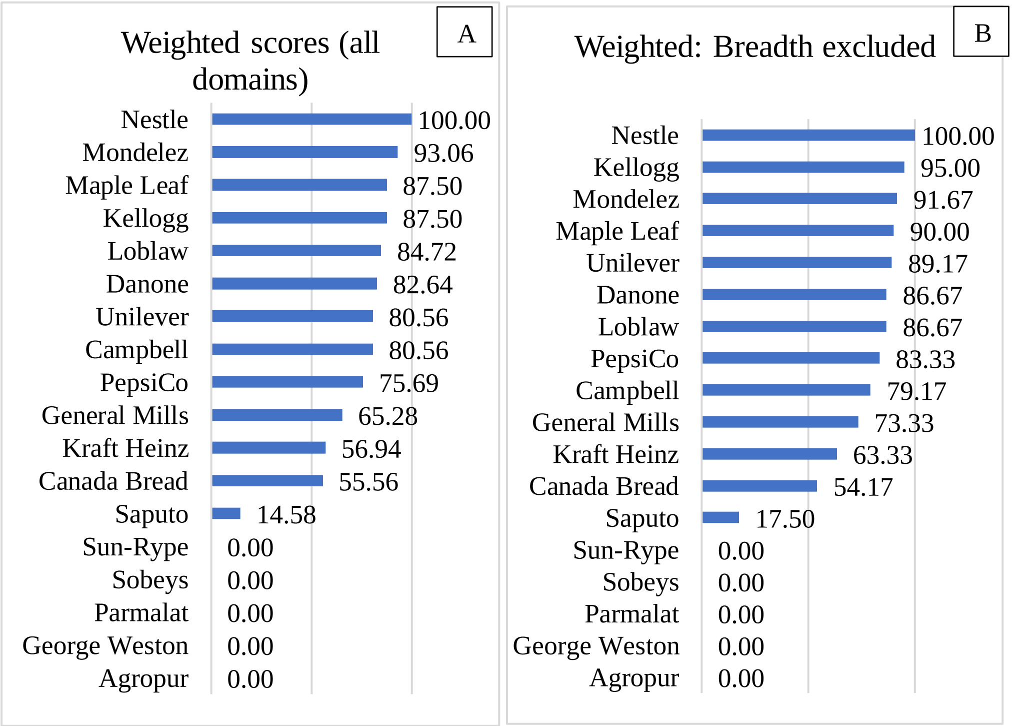

Removing the breadth domain caused Kellogg to move ahead of Mondelez and Maple Leaf.

Loblaw fell behind Unilever and became tied with Danone.

PepsiCo moved ahead of Campbell.

Supplementary Figure D-13. Original sodium scores vs. magnitude of reduction domain removed (SOD4.1, 4.2, 4.3, 4.4, 10.1, 10.2, 10.3, 10.4).

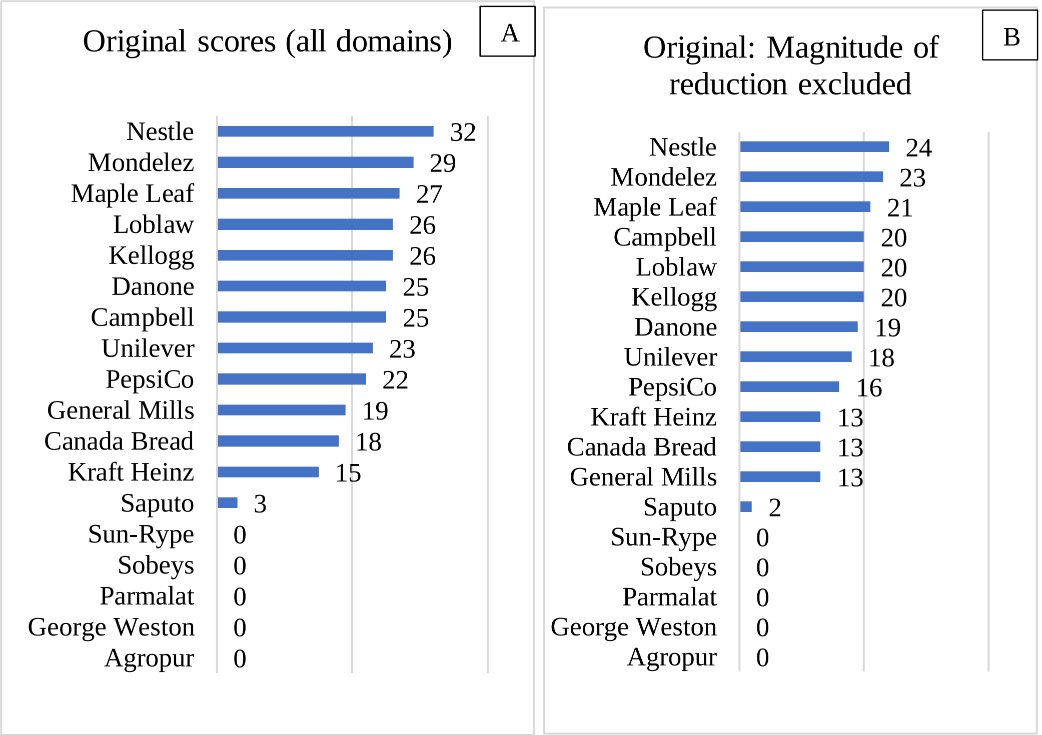

Removing the magnitude of reduction domain caused Campbell to move ahead and become tied with Loblaw and Kellogg. Kraft Heinz, Canada Bread and General Mills became tied.

Supplementary Figure D-14. Weighted sodium scores vs. magnitude of reduction domain removed (SOD4.1, 4.2, 4.3, 4.4, 10.1, 10.2, 10.3, 10.4).

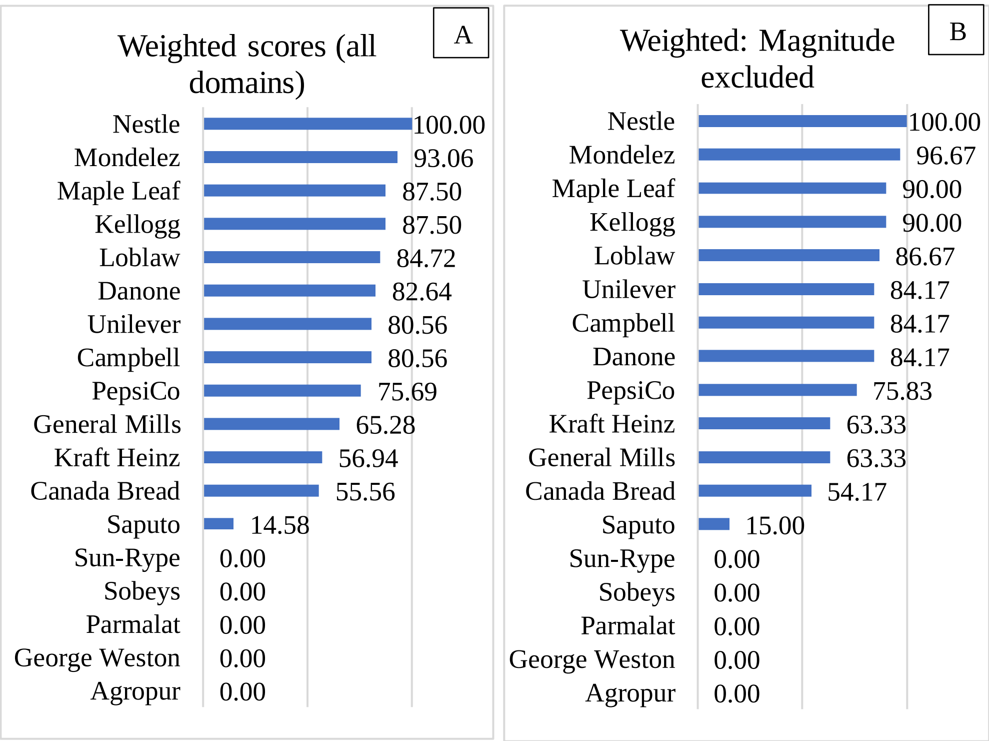

Removing the magnitude of reduction domain caused Unilever and Campbell to move ahead and become tied with Danone.

Kraft Heinz moved up and became tied with General Mills.

Supplementary Figure D-15. Original sodium scores vs. national/global applicability domain removed (SOD5.1, 5.2, 11.1, 11.2).

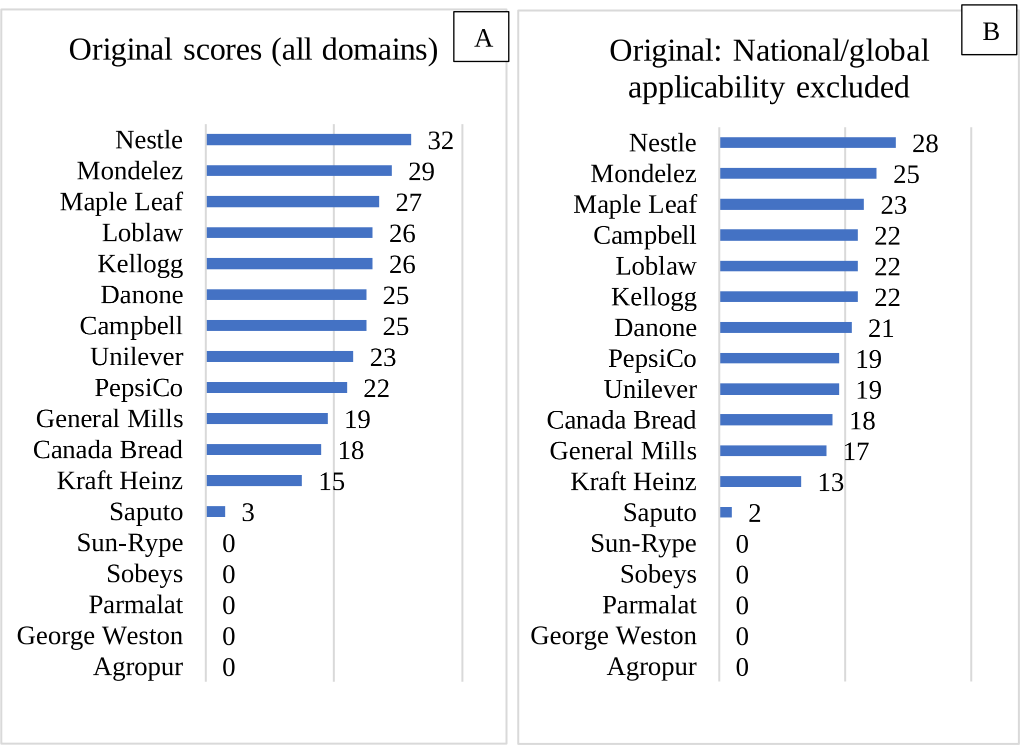

Removing the national/global applicability domain caused Campbell to move ahead and become tied with Loblaw and Kellogg.

PepsiCo moved ahead and became tied with Unilever.

Canada Bread moved ahead of General Mills.

Supplementary Figure D-16. Weighted sodium scores vs. national/global applicability domain removed (SOD5.1, 5.2, 11.1, 11.2).

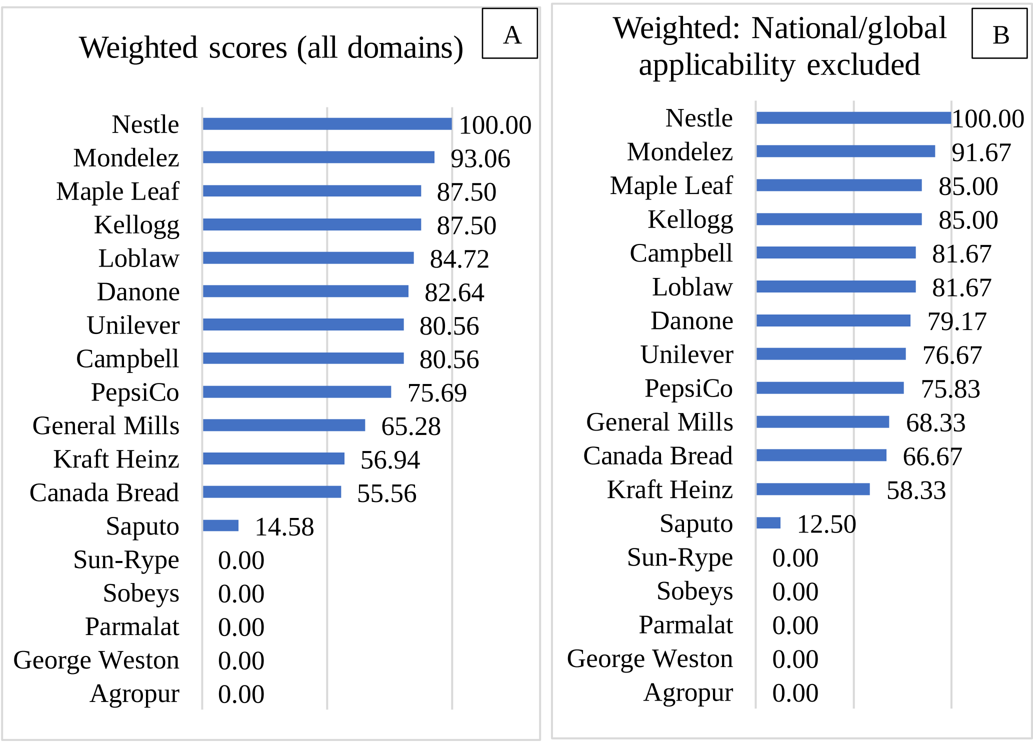

Removing the applicability domain caused Campbell to move ahead and become tied with Loblaw.

Supplementary Figure D-17. Original sodium scores vs. transparency domain removed (SOD6.1, 6.2, 6.3, 12.1, 12.2, 12.3).

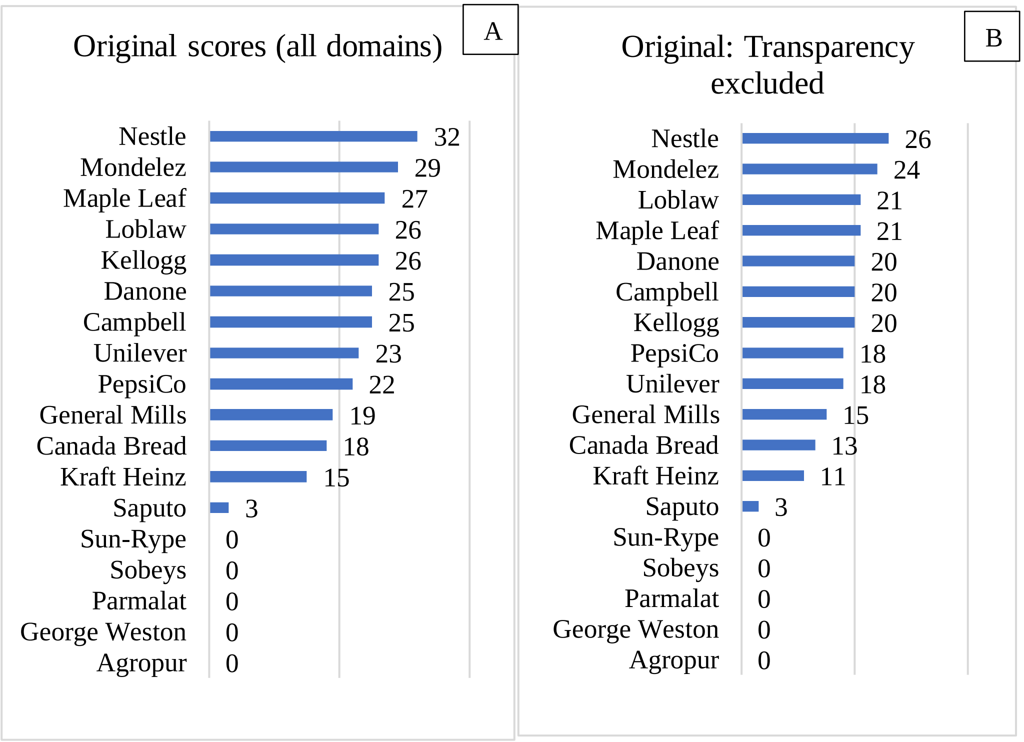

Removing transparency domain caused Loblaw to move ahead and become tied with Maple Leaf.

Danone and Campbell became tied with Kellogg.

PepsiCo moved ahead and became tied with Unilever.

Supplementary Figure D-18. Weighted sodium scores vs. transparency domain removed (SOD6.1, 6.2, 6.3, 12.1, 12.2, 12.3).

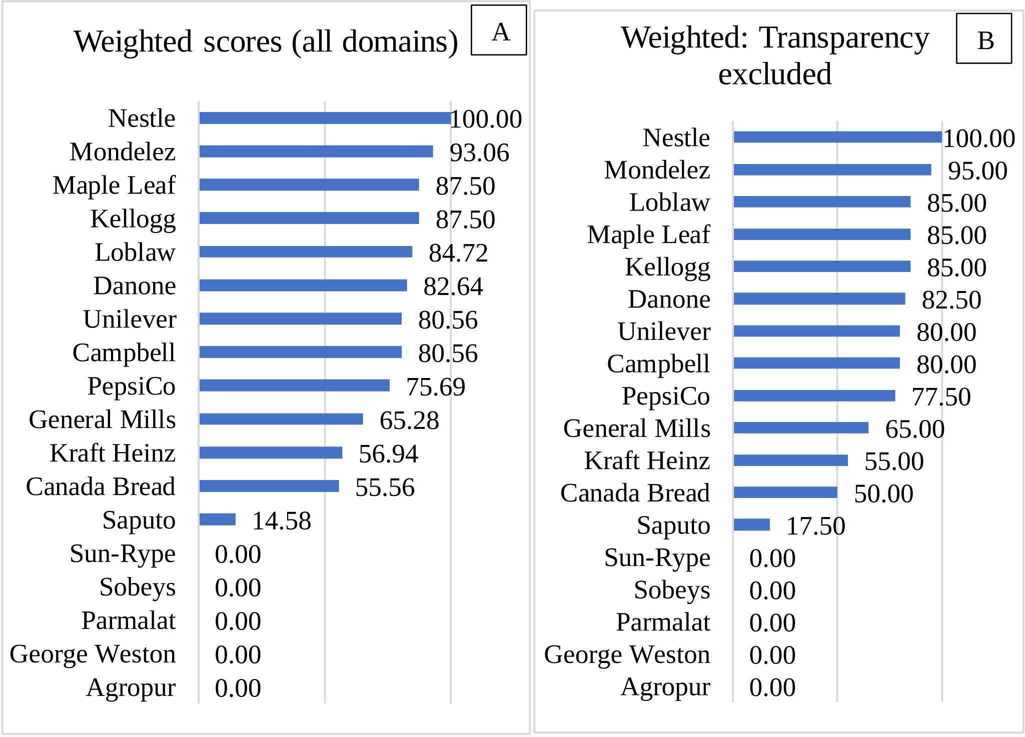

Removing the transparency domain caused Loblaw to move ahead and become tied with Maple Leaf and Kellogg.

Application of FCR tool scores in this study

In this study, we examined the relationship between companies' FCR tool scores and changes in the nutritional quality of their products between 2013 and 2017 through the use of generalized estimating equations (GEEs). Based on our examination of the internal consistency of the FCR tool, which included examining the effect of assigning equal weighting to the 6 domains of the sodium section of the tool, we decided to use weighted scores in the GEE analysis so that each nutrient/component and domain were treated equally in companies' final scores. Equally weighted scores provide a more equitable evaluation of companies' recent actions or commitments, rather than weighting them arbitrarily based on the number of indicators in each domain (which range from 2 to 8). Total scores were also weighted, assigning equal weight to each of the 5 nutrients/components for packaged food companies or 2 nutrients/components for beverage companies (which were not evaluated in terms of sodium, saturated fat or *trans* fat). The 5 additional indicators were excluded from our analysis in this study to simplify the weighting process and because they are not directly related to the reformulation of any one nutrient or component. However, sensitivity analyses examined the impact of including the additional indicators (with weightings of 3.3% of the total score for packaged food companies and 7.8% for beverage companies, based on their weighting in the original total scores), and no differences in the GEE results were observed.

Internal consistency testing indicated that, aside from the reduction/commitment domain, most domains or indicators appeared to have moderate or strong influence on companies' final FCR sodium scores. Nonetheless, given that excluding the reduction/commitment domain did not change the overall ranking of companies' scores (Supplementary Figures D-7 and D-8), we elected to retain it in our analyses. The domains and indicators included in the FCR tool were selected based on a review of public health recommendations concerning product (re)formulation, existing methodologies/tools for assessing companies' reported actions and commitments concerning nutrition, and through consultation with global experts in private sector nutrition policy. In light of this and the results of the content/face validity, construct/convergent validity and internal consistency testing presented in this chapter, we decided to keep all of the domains and indicators that were originally included in the FCR tool in our analyses in this study.

Sensitivity analyses also examined whether our results would change if rather than using companies' total FCR scores for sodium, we used their scores for each of the 6 individual domains (recent reduction commitment; timebound; breadth; magnitude of reduction; national/global applicability; and transparency). Six separate GEE models were constructed for each domain score (as the continuous predictor of interest), with year and TRA category as additional independent variables, an interaction term between the "domain score" and "year" variables and sodium per 100 g/mL as the dependent variable (as was done in the original analyses). These results were similar those of our original analysis, with the interaction term between domain score and year found not to be statistically significant ($p \geq 0.05$), indicating there was no difference in the change in sodium contents over time between products offered by companies with higher versus lower scores for each sodium domain. Therefore, all six domains were retained in the final GEE analyses for this study.

Inter-rater reliability

Inter-rater reliability is the extent to which the judgements of different observers are consistent. In the context of the FCR scoring tool, inter-rater reliability measured the extent to which scores assigned for all companies assigned independently by two researchers were in alignment[1]. FCR scores for all 22 companies were assigned independently and inter-rater-reliability was assessed using Gwet's AC1 statistic, which has been shown to provide a more stable measure of inter-rater reliability than the commonly used Cohen's Kappa[21]. Inter-rater reliability was high, with a Gwet's AC1 inter-rater reliability coefficient of 0.88 (95% CI: 0.87, 0.89), indicating that the FCR tool can be applied consistently. This is critical if the tool is to be applied repeatedly over time by different researchers in various countries.

References

1. Price PC, Jhangiani R, Chiang I-CA. Reliability and Validity of Measurement. BCcampus; 2015:chap Chapter 5: Psychological Measurement. Accessed May 11, 2021. https://opentextbc.ca/researchmethods/
2. Townsend MS. Where is the science? What will it take to show that nutrient profiling systems work? *Am J Clin Nutr*. Apr 2010;91(4):1109S-1115S. doi:10.3945/ajcn.2010.28450F
3. Poon T, Labonté M, Mulligan C, Ahmed M, Dickinson KM, L'Abbé MR. Comparison of nutrient profiling models for assessing the nutritional quality of foods: a validation study. *Br J Nutr*. Sep 2018;120(5):567-582. doi:10.1017/S0007114518001575
4. World Health Organization. Healthy diet. 2020. Accessed May 11, 2021. https://www.who.int/news-room/fact-sheets/detail/healthy-diet
5. Access to Nutrition Index. *Global Index 2018 - Corporate Profile Methodology*. 2017. Accessed May 11, 2021. https://accesstonutrition.org/index/global-index-2018/methodology/
6. Access to Nutrition Foundation. Access to Nutrition Index: U.S. Spotlight Index 2018. 2018. Accessed May 11, 2021. https://accesstonutrition.org/app/uploads/2020/02/Spotlight_Index_US-Index_Full_Report_2018.pdf
7. Access to Nutrition Foundation. Access to Nutrition Initiative: India Spotlight Index 2020. 2020. Accessed May 11, 2021. https://accesstonutrition.org/index/india-spotlight-2020/
8. Sacks G, Vanderlee L, Robinson E, et al. BIA-Obesity (Business Impact Assessment - Obesity and population-level nutrition): A tool and process to assess food company policies and commitments related to obesity prevention and population nutrition at the national level *Obesity Reviews*. 2019;(Future Directions in Obesity Prevention):1-12. 9. World Health Organization. Global Targets 2025. Accessed January 13,, 2021. https://www.who.int/nutrition/global-target-2025/en/
10. World Health Organization. Comprehensive implementation plan on maternal, infant and young child nutrition. 2014. Accessed May 11, 2021. https://www.who.int/nutrition/publications/CIP_document/en/
11. United Nations. Sustainable Development Goals: Goal 2: Zero Hunger. Accessed May 11, 2021. https://www.un.org/sustainabledevelopment/hunger/
12. World Health Organization, Food and Agriculture Organization of the United Nations. Strengthening nutrition action: a resource guide for countries based on the policy recommendations of the Second International Conference on Nutrition (ICN2). 2018. Accessed May 11, 2021. https://apps.who.int/nutrition/publications/strengthening-nutrition-action/en/index.html
13. Nutrition for Growth (N4G). Commitment-Making Guide. 2020. Accessed May 11, 2021. https://nutritionforgrowth.org/wp-content/uploads/2020/06/Nutrition-for-Growth-2020-Commitment-Guide-Updated-March-2020-1.pdf
14. NCD Alliance, World Cancer Research Fund International. Ambitious, SMART commitments to address NCDs, overweight and obesity (Extended Version). Accessed May 11, 2021. https://www.wcrf.org/int/policy/our-publications/ambitious-smart-commitments-address-ncds-overweight-and-obesity

15. Food and Agriculture Organization of the United Nations, World Health Organization. Second International Conference on Nutrition Conference Outcome Document: Framework for Action. 2014. Accessed May 11, 2021. http://www.fao.org/3/a-mm215e.pdf

16. Cooper SL, Pelly FE, Lowe JB. Construct and criterion-related validation of nutrient profiling models: A systematic review of the literature. *Appetite*. May 2016;100:26-40. doi:10.1016/j.appet.2016.02.001
17. Trochim WMK. Convergent & Discriminant Validity. Conjoint.ly. Accessed May 11, 2021. https://conjointly.com/kb/convergent-and-discriminant-validity/
18. Access to Nutrition Foundation. Access to Nutrition Initiative Methodology. Accessed May 11, 2021. https://accesstonutrition.org/methodology/
19. Vanderlee L, Vergeer L, Sacks G, Robinson E, L'Abbé M. Food and beverage manufacturers in Canada: policies and commitments to improve the food environment. University of Toronto. 2019. Accessed May 11 2021. http://labbelab.utoronto.ca/bia-obesity-canada-2019/
20. Health Canada. Guidance for the Food Industry on Reducing Sodium in Processed Foods. 2012. 2012. Accessed May 11, 2021. https://www.canada.ca/content/dam/hc-sc/migration/hc-sc/fn-an/alt_formats/pdf/legislation/guide-ld/2012-sodium-reduction-indust-eng.pdf
21. Wongpakaran N, Wongpakaran T, Wedding D, Gwet KL. A comparison of Cohen's Kappa and Gwet's AC1 when calculating inter-rater reliability coefficients: a study conducted with personality disorder samples. *BMC Med Res Methodol*. Apr 2013;13:61. doi:10.1186/1471-2288-13-61.
